# Dynamic transcriptomic analysis of Ischemic Injury in a Porcine Pre-Clinical Model mimicking Donors Deceased after Circulatory Death

**DOI:** 10.1038/s41598-018-24282-6

**Published:** 2018-04-13

**Authors:** Sebastien Giraud, Clara Steichen, Geraldine Allain, Pierre Couturier, Delphine Labourdette, Sophie Lamarre, Virginie Ameteau, Solenne Tillet, Patrick Hannaert, Raphael Thuillier, Thierry Hauet

**Affiliations:** 1 Inserm U1082 IRTOMIT, Poitiers, F-86000 France; 2 Université de Poitiers, Faculté de Médecine et de Pharmacie, Poitiers, F-86000 France; 3 CHU Poitiers, Service de Biochimie, Poitiers, F-86000 France; 4 CHU Poitiers, Service de chirurgie cardio-thoracique, Poitiers, 86000 France; 5 MOPICT, IBiSA plateforme ‘Experimental Surgery and Transplantation’, Domaine du Magneraud, Surgères, F-17700 France; 6 LISBP, Université de Toulouse, CNRS, INRA, INSA, Toulouse, F- 31077 France; 7 FHU SUPORT ‘SUrvival oPtimization in ORgan Transplantation’, Poitiers, F-86000 France

## Abstract

Due to organ shortage, clinicians are prone to consider alternative type of organ donors among them donors deceased after circulatory death (DCD). However, especially using these organs which are more prone to graft dysfunction, there is a need to better understand mechanistic events ocuring during ischemia phase and leading to ischemia/reperfusion injuries (IRI). The aim of this study is to provide a dynamic transcriptomic analysis of preclinical porcine model kidneys subjected to ischemic stress mimicking DCD donor. We compared cortex and corticomedullary junction (CMJ) tissues from porcine kidneys submitted to 60 min warm ischemia (WI) followed by 0, 6 or 24 hours of cold storage in University of Wisconsin solution versus control non-ischemic kidneys (n = 5 per group). 29 cortex genes and 113 CMJ genes were significantly up or down-regulated after WI versus healthy kidneys, and up to 400 genes were regulated after WI followed by 6 or 24 hours of cold storage (p < 0.05). Functionnal enrichment analysis (home selected gene kinetic classification, Gene-ontology-biological processes and Gene-ontology-molecular-function) revealed relevant genes implication during WI and cold storage. We uncovered targets which we will further validate as biomarkers and new therapeutic targets to optimize graft kidney quality before transplantation and improve whole transplantation outcome.

## Introduction

Transplantation remains the only efficient therapeutic option for end-stage renal diseases. However, this success led to a worldwide organ shortage, and only 30% of patients on the waiting list have access to an organ. This situation, combined with unavoidable demographic change in donor population, has led to the growing use of organs coming from marginal or “extended-criteria” donors, including deceased after circulatory death donors (DCD)^1^. However, organs coming from marginal donors are more prone to develop ischemia injuries, harmful during reperfusion for organ quality and outcome. Importantly, ischemia-reperfusion (IR) injuries (IRI) are correlated with delayed graft function rate and primary non function rate^1^. Hence the need to consider new strategies to improve organ preservation quality. Indeed, from cardiac death of the donor, through organ procurement and its cold storage until graft revascularistion in the recipient, ischemic injuries lay the groundwork for secondary lesions resulting in worsening the outcome. In transplantation, severe reperfusion injuries after transplantation are mostly caused by the initiation of ischemic lesions. However, the molecular mechanisms underlying severe ischemia are currently not fully determined and this knowledge gap limits the current effort to design better approach for organ preservation. While several solutions using technological or pharmacological improvements have been tested^2^, the lack of specific targets as well as the reduced number of available biomarkers are slowing down the development and transfer to the clinic. In addition, IRI- focusing studies mostly rely on the use of small animal models where the application of ischemia protocols similar to those used in clinic is challenging and/or studies are focused on a single pathway in a hypothesis-driven fashion. We propose here an open ended approach based on microarray technology to understand IRI occurring in DCD-like kidney submitted to warm-ischemia (WI) followed by cold ischemia (CS), using a pre-clinical porcine model with the main advantage that porcine and human kidneys are extremely similar in size, structure and function^3^. The specific objective of this study is to evaluate gene expression profile of kidney submitted to ischemic injury similarly to what is observed in clinic with kidneys coming from DCD donors i.e submitted to WI followed by cold ischemia. Indeed, we investigated differential gene expression patterns in kidneys after a period of WI followed or not by a cold storage (CS) of 6 h (WI + CS6h) or 24 h (WI + CS24h) versus control non-ischemic kidneys (Ctl) in a reproducible model. Identified gene expression profiles were submitted to functional enrichment analysis and a comprehensive bibliographic review was performed to understand the role of each marker in the biological serie of events occurring during ischemia. Our aim is to determine specific inhibited/activated genes which could become pharmacological target and define markers useful to evaluate organs before transplantation.

## Results

### Ischemia impact on global gene expression profile

**Figure 1:**
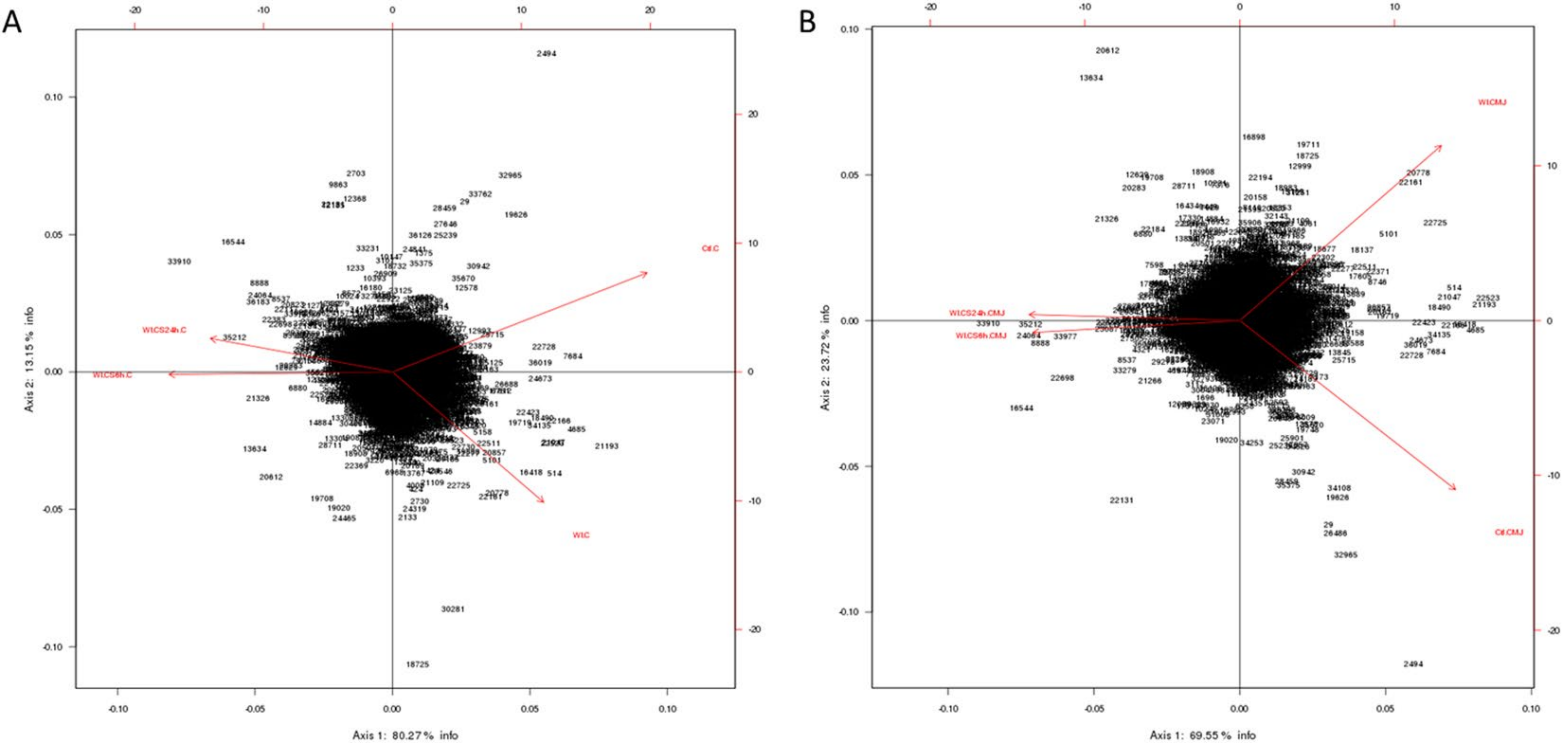
Principal Component Analysis (PCA) plots of the integrated gene expression data matrix for all analyzed genes, for (**A**) cortex genes and (**B**) CMJ genes. Numbers in parentheses represent the percentage of total variance explained by the first and second PC.

From microarray datasets, we generated heatmaps to depict mRNA expression between our groups and we submitted our data to Principal Component Analysis (PCA). Numbers in parentheses represent the percentage of total variance explained by the first and second principal components, explaining respectively 80.27% and 13.15% of the variability of our results for cortex (C) genes (Fig. 1A), and 59.55 and 23.77% of the variability of our results for CMJ genes (Fig. 1B), showing that our experimental groups have well distanced transcriptomic profiles. Moreover, for both cortex and CMJ genes, PCA shows that both experimental groups submitted to cold storage are clustering closely, and are well separated from the two others groups i.e control and WI groups, highlighting that the duration of the cold storage itself has moderate impact on the variability of our results and therefore on sample gene expression profiles.

To further decipher transcriptomic changes occurring during our ischemia treatments, we then focused on heatmaps-extracted differentially expressed genes between selected groups. Precisely, we found that the number of differentially expressed genes between different ischemia-treated groups and control group (Ctl) was: 29 cortex genes and 113 CMJ genes between 60 minutes of WI vs Ctl group, 1145 cortex genes and 456 CMJ genes between 60 minutes of WI followed by 6 hours of cold storage (WI + CS6h) vs Ctl group and 805 cortex genes and 485 CMJ genes between 60 minutes of WI followed by 24 hours of cold storage (WI + CS24h) vs Ctl group. Additionally, our heatmap results showed that 561 cortex genes and 462 CMJ genes were differentially expressed between WI and WI + CS6 groups, and 398 cortex genes and 446 CMJ genes were differentially expressed between WI and WI + CS24 groups (Supplementary Tables S1 and S2). The criteria for significance being |log2 fold change| ≥ 0.5 and a corrected p-value < 0.05.

### Home-made panel gene expression dynamics

We extracted from heatmaps the kinetic expression of several relevant genes significantly differentially expressed and classed in categories having an important role in IRI (Figs 2–7). Heatmaps are available on Supplementary Figures S1–S10.

**Figure 2:**
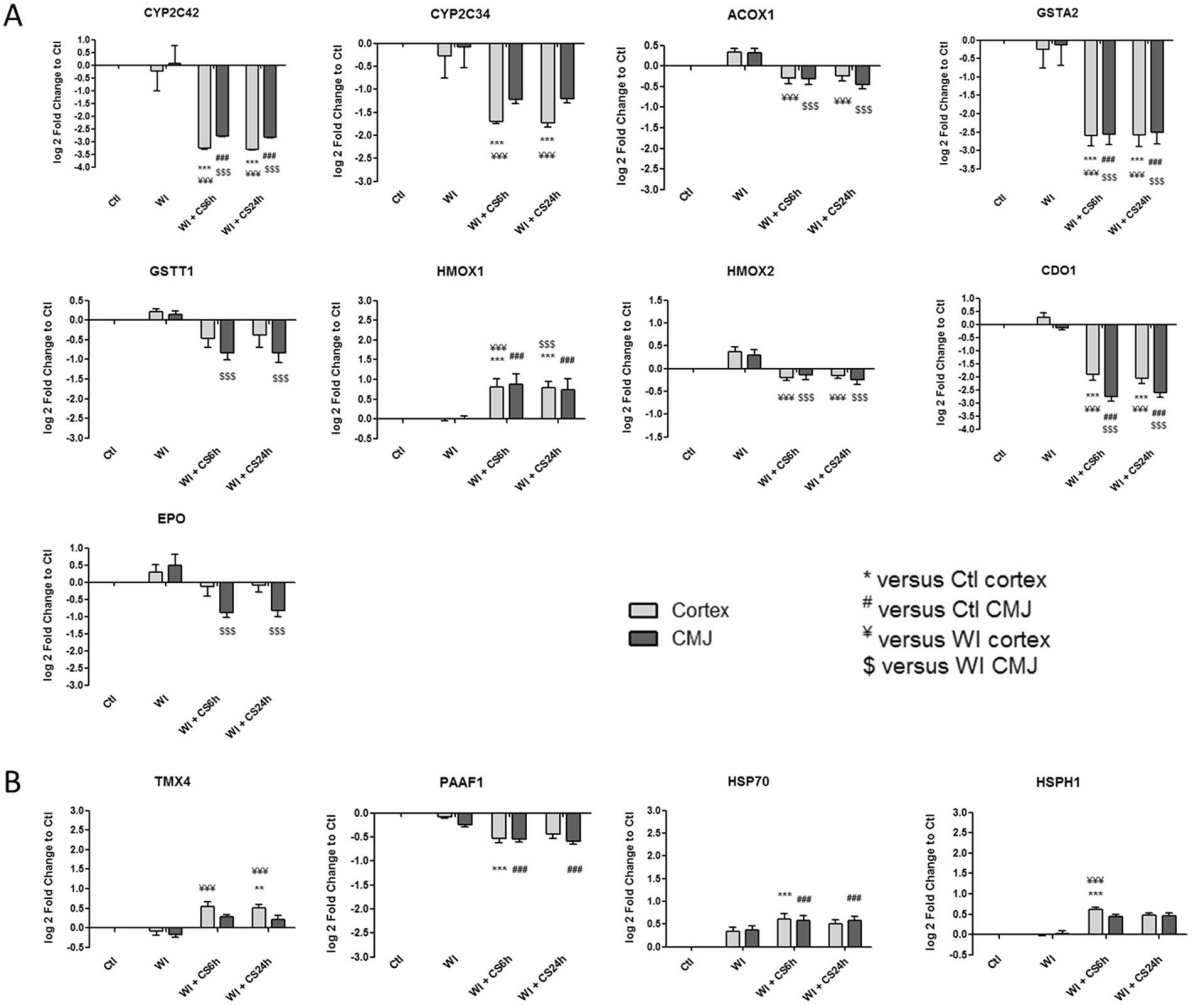
Kinetic expression of relevant genes from (**A**) “Mitochondria and redox state regulation” and (**B**) “Protein folding and proteasome” categories which are significantly differentially expressed from heatmaps. Results are expressed in mean ± SEM, **p < 0.01, ***p < 0.001 versus Ctl cortex, ^##^p < 0.01, ^###^p < 0.001 versus Ctl CMJ, ^¥¥^p < 0.01, ^¥¥¥^p < 0.001 versus WI Cortex, ^$$^p < 0.001, ^$$$^p < 0.001 versus WI CMJ.

**Figure 3:**
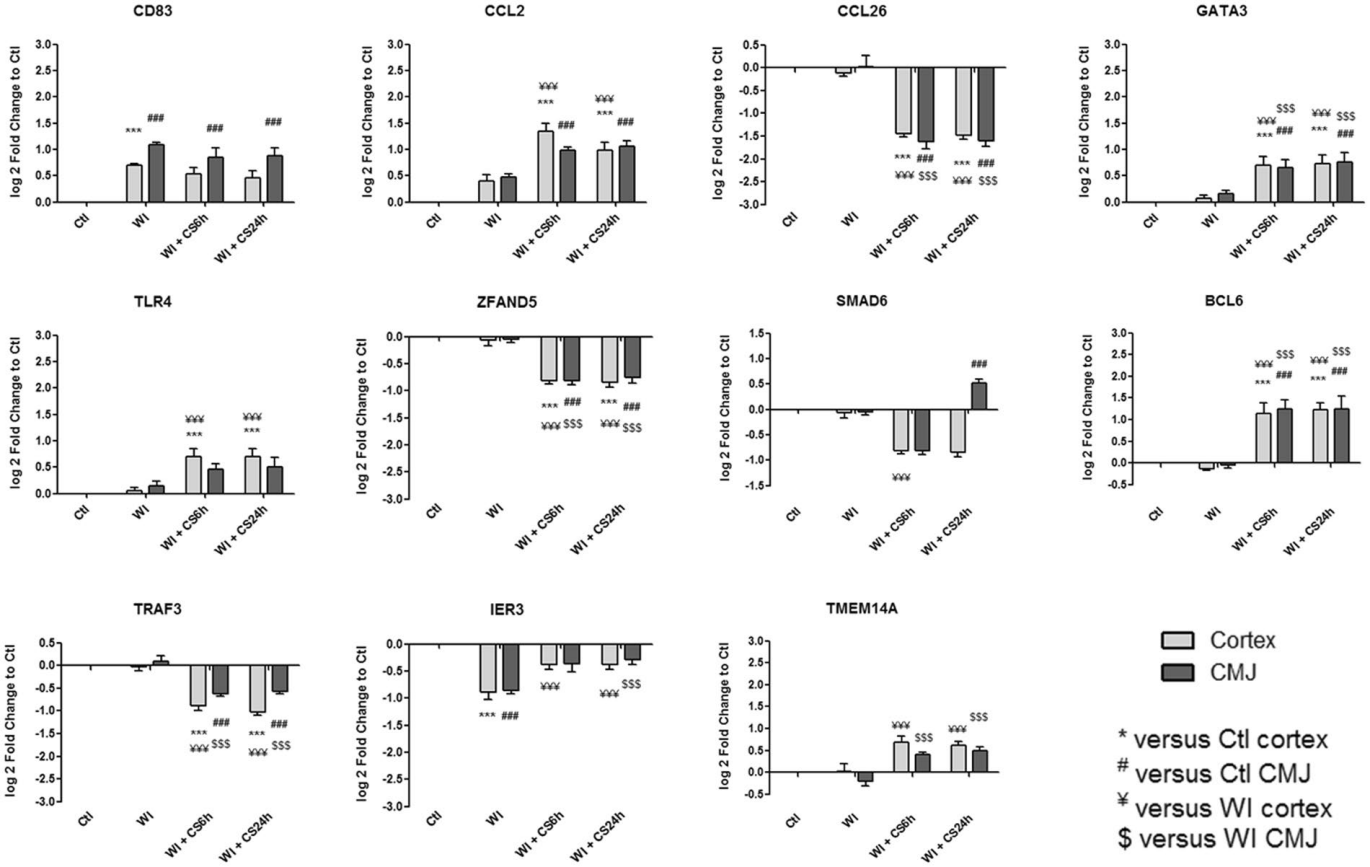
Kinetic expression of relevant genes from “Inflammation and apoptosis” category which are significantly differentially expressed from heatmaps. Results are expressed in mean ± SEM, **p < 0.01, ***p < 0.001 versus Ctl cortex, ^##^p < 0.01, ^###^p < 0.001 versus Ctl CMJ, ^¥¥^p < 0.01, ^¥¥¥^p < 0.001 versus WI Cortex, ^$$^p < 0.001, ^$$$^p < 0.001 versus WI CMJ.

**Figure 4:**
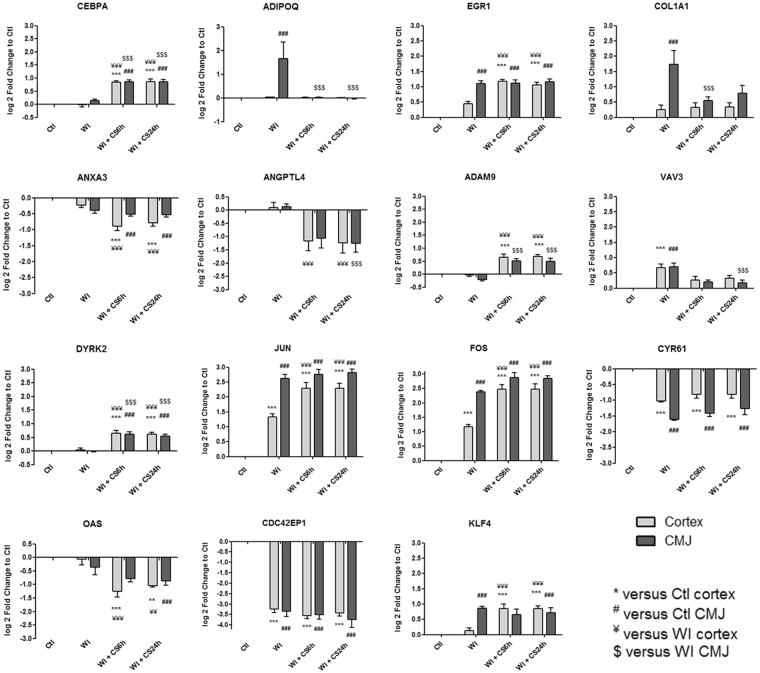
Kinetic expression of relevant genes from “Cell cycle, cellular differentiation and proliferation” category which are significantly differentially expressed from heatmaps. Results are expressed in mean ± SEM, **p < 0.01, ***p < 0.001 versus Ctl cortex, ^##^p < 0.01, ^###^p < 0.001 versus Ctl CMJ, ^¥¥^p < 0.01, ^¥¥¥^p < 0.001 versus WI Cortex, ^$$^p < 0.001, ^$$$^p < 0.001 versus WI CMJ.

**Figure 5:**
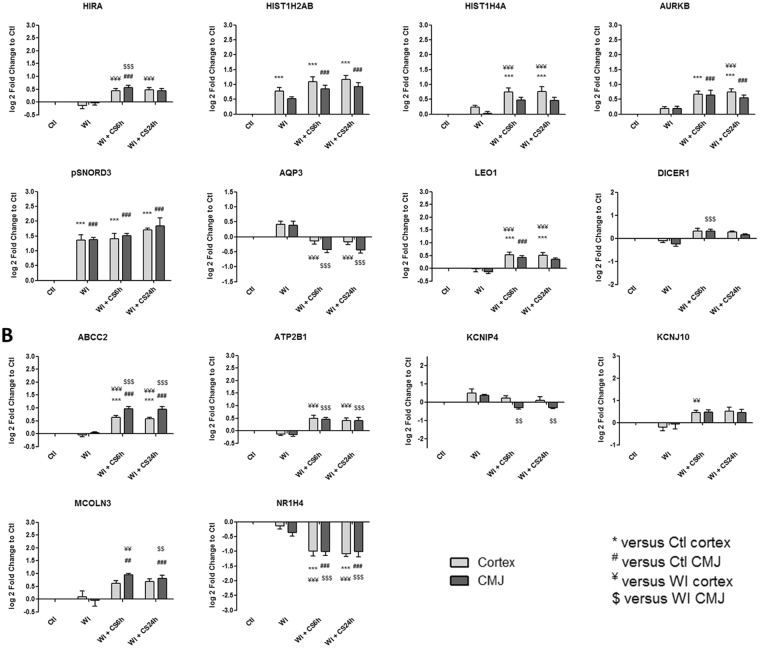
Kinetic expression of relevant genes from (**A**) “Nucleus genes and transcriptional regulation” and (**B**) “Transporters” categories which are significantly differentially expressed from heatmaps. Results are expressed in mean ± SEM, **p < 0.01, ***p < 0.001 versus Ctl cortex, ^##^p < 0.01, ^###^p < 0.001 versus Ctl CMJ, ^¥¥^p < 0.01, ^¥¥¥^p < 0.001 versus WI Cortex, ^$$^p < 0.001, ^$$$^p < 0.001 versus WI CMJ.

**Figure 6:**
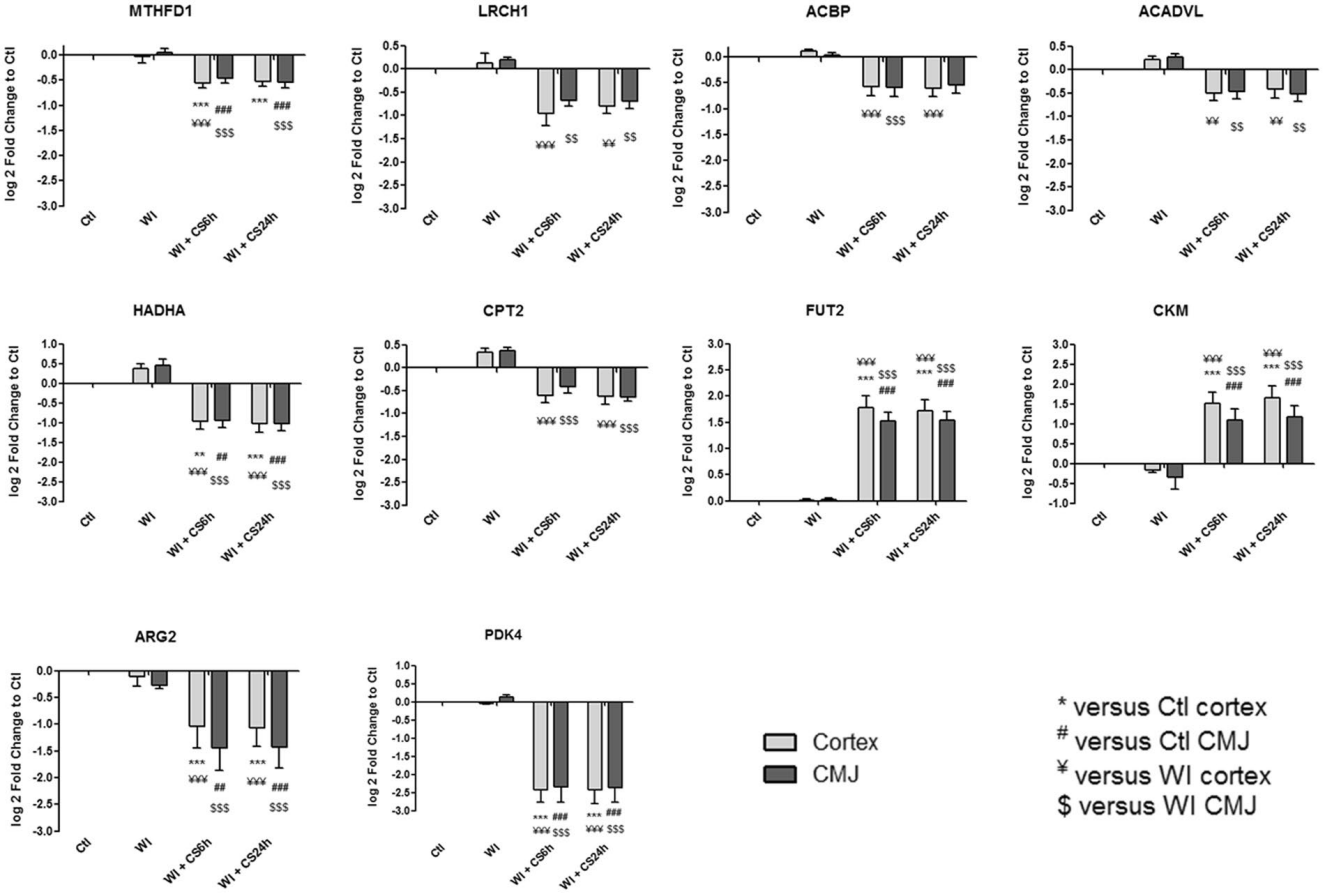
Kinetic expression of relevant genes from “Metabolism regulation” category which are significantly differentially expressed from heatmaps. Results are expressed in mean ± SEM, **p < 0.01, ***p < 0.001 versus Ctl cortex, ^##^p < 0.01, ^###^p < 0.001 versus Ctl CMJ, ^¥¥^p < 0.01, ^¥¥¥^p < 0.001 versus WI Cortex, ^$$^p < 0.001, ^$$$^p < 0.001 versus WI CMJ.

**Figure 7:**
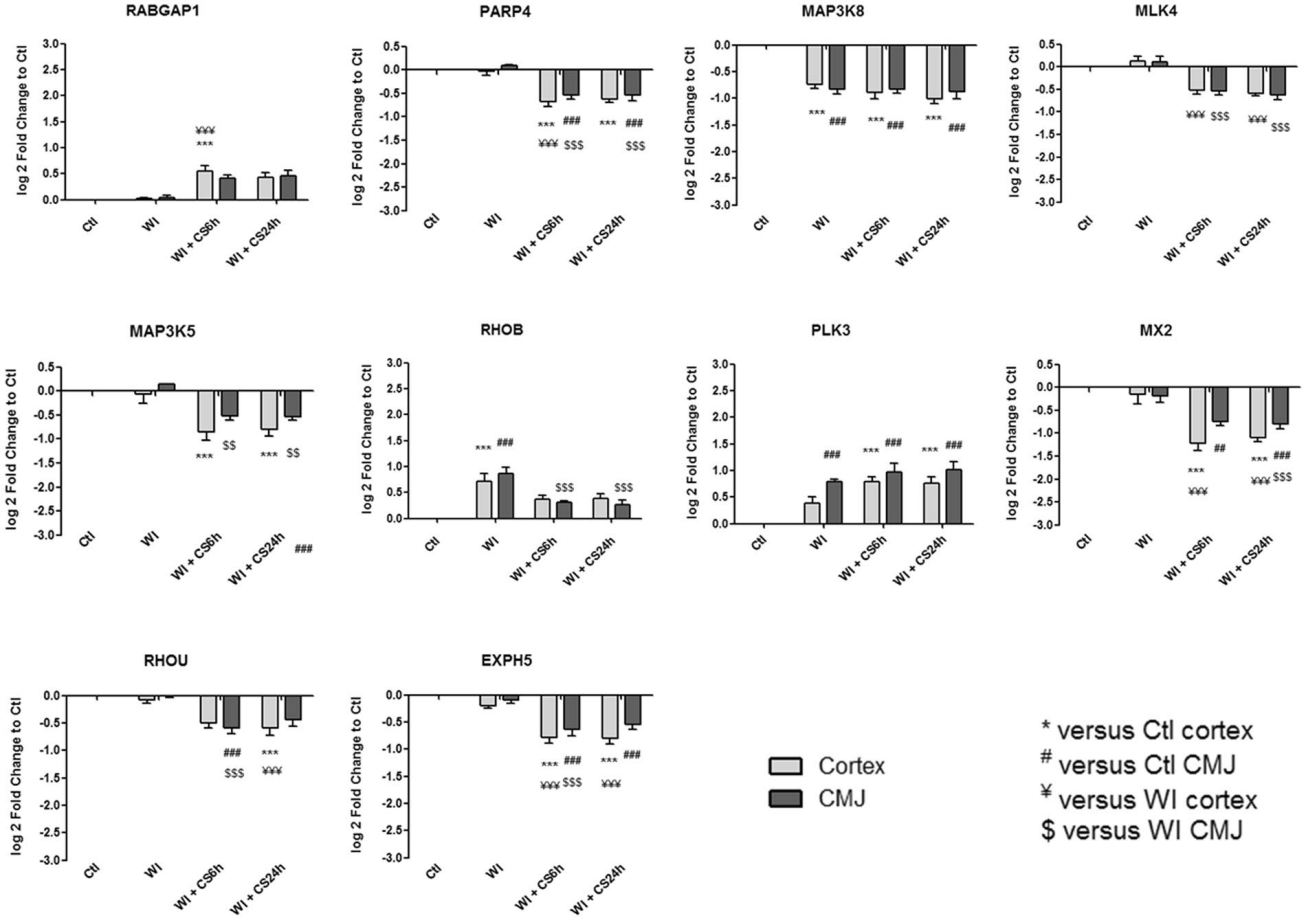
Kinetic expression of relevant genes from “MAPK and GTPase activity” category which are significantly differentially expressed from heatmaps. Results are expressed in mean ± SEM, **p < 0.01, ***p < 0.001 versus Ctl cortex, ^##^p < 0.01, ^###^p < 0.001 versus Ctl CMJ, ^¥¥^p < 0.01, ^¥¥¥^p < 0.001 versus WI Cortex, ^$$^p < 0.001, ^$$$^p < 0.001 versus WI CMJ.

### Functional enrichment analysis

The genes we identified as differentially expressed were further submitted to enrichement analysis based on Gene-Ontology Biological-Process (GO-BP) and Gene-Ontology Molecular-Function (GO-MF) categories (Gene Ontology Families). We detected up- and down-regulated categories in cortex tissues represented respectively in Supplementary Tables S3 and S5, and in CMJ represented respectively in Supplementary Tables S4 and S6 (p < 0.05). Each family contained one or more differentially expressed genes. Most significative GO-BP and GO-MF categories of genes identified by the functionnal enrichment analysis are available on Supplementary Figures S12.

### Confirmation by Real-Time Polymerase Chain Reaction

In order to confirm microarray results, we chose genes which where differentially expressed in our analysis and performed RT-qPCR to validate our results (Supplementary Table S7). We found significant correlations between Agilent heatmaps and RT-qPCR results, for both cortex (spearman r = 0.84, p < 001) and CMJ (spearman r = 0.88, p < 001) genes (Supplementary Figure S11).

## Discussion

While attempting to better understand ischemia mechanisms, we performed a high throughput transciptomic analysis of pig kidneys subjected to intense ischemia injury observed in DCD organ transplantation. Our model has significant advantage over other transcriptomic analyses of rodent kidneys submitted to warm-ischemia^4,5^, since these latters are far to be anatomically and physiologically similar to their human counterparts^3^. A clinical study of Naesens *et al*. reported transcriptomic analysis of human kidneys coming from DCD donors versus living donors at the end of preservation period, but they did not compare their data with control healthy kidneys, leading to difficulties to isolate consequences of ischemia phase itself^6^. In a similar study comparing the same type of samples, authors submitted raw data to Kyoto Encyclopedia of Genes and Genomes (KEGG) analysis and highlighted several variability factors between samples such as cause of death, donor age, warm-ischemia time, cold ischemia duration and preservation solution^7^. We investigated differential gene expression patterns in each kidney after a period of WI followed by cold storage (WI + CS) versus Control non-ischemic kidneys (Ctl) in a reproducible model. Our experimental design allowed us to avoid the high variability reported in human studies, using yet a preclinical model of kidney transplantation highly transposable to clinic^3^.

Identified gene expression profiles were submitted to functional enrichment analysis and a comprehensive bibliographic review was performed to understand the role of each marker in the biological serie of events occurring during ischemia. We analyzed the kinetics of the most differentially expressed genes and families from GO-BP and GO-MF families and we hypothesize that these genes could play a key role in IRI, and are subdivided in the following 8 categories:

1. **“Mitochondria and redox state regulation”** such as CYP2C42, CYP2C34, ACOX1, GSTA2, GSTT1, HMOX1, HMOX2, CDO1 and EPO (Fig. 2A). Mitochondria, involved in the redox state regulation, are the most important organel affected by ischemia. CYP2C42 and CYP2C34 are proteins of the Cytochromes P450 family containing heme as a cofactor. They usually are the terminal oxidase enzymes in electron transfer chains. Similarly to what we found, prolonged ischemia decrease cytochromes P450 isoforms levels in a rat kidney IRI model^8^. Acyl-CoA oxidase (ACOX) regulation may be a reflection of the loss of acyl-CoA oxidase activity^9^. Glutathione S-transferase Alpha (GSTA) and Theta (GSTT) enzymes are major actors of oxidative stress products detoxification. They are dowregulated in cold-ischemia, conforting the fact that the oxidative stress response is acting at the reperfusion time. Heme oxygenases (HMOX) are enzymes catalyzing heme degradation. HMOX1 encodes Heme oxygenase 1 (HO-1), an inducible isoform responding to hypoxia. We indeed observe HMOX1 upregulation in our study, likely trigerring heme degradation and IRI protection^10^, probably via the NRF2-AKT interactions^11^. HMOX2 encodes HO-2, a constitutive isoform that is expressed under homeostatic conditions. Cysteine dioxygenase (CDO1) is a non-heme iron enzyme catalyzing conversion of L-cysteine to cysteine sulfinic acid by dioxygen incorporation. Cysteine residues maintained and transduced redox signals in the mitochondria^12,13^. Erythropoietin (EPO), activated by HIF-1a transcription factor, is upregulated by the kidney in response to cellular hypoxia^14^. In our study, 30 min of WI seems to be too short to upregulate significantly cytoprotectives EPO and CDO1 RNAs.
2. “**Protein folding and proteasome”** such as TMX4, PAAF1, PLK3, HSP70 and HSPH1 (Fig. 2B). Like mitochondria, the endoplasmic reticulum and the proteasome are important cellular compartiments altered by ischemia. In accordance with our results, oxidative stress induces mitochondrial dysfunction and protective unfolded protein response in epithelial cells, with upregulation of thioredoxin-related transmembrane protein 4 (TMX4) mRNA^15^, as well as lower expression of catalytic and structural subunits of the proteasome, as Proteasomal ATPase-associated factor 1 (PAAF1), contributes to decreased proteolysis^16^. Polo-like kinase 3 (PLK3), herein upregulated, is involved in the GO-BP family “regulation of proteasomal ubiquitin-dependent protein catabolic” at WI + CS6h vs Ctl (Supplementary Table S3). Heat shock protein 70 kDa (Hsp70) family play a major role in cell machinery for protein folding, helpful for stress protection^17^. Hsp105 (HSPH1) interacts with Cofilin-1^18^ preventing the aggregation of denatured proteins in cells under severe stress where the ATP levels decrease markedly^19^. Similarly to other studies where ischemia activates protective mRNA transcripts for heat shock proteins in rat heart^20^, our results confort that HSP expression upregulation is protective against injury.
3. “**Inflammation and apoptosis”** such as CD83, CCL2, CCL26, GATA3, TLR4, ZFAND5, SMAD6, BCL6, TRAF3, IER3 and TMEM14A (Fig. 3). Inflammation and apoptosis are important consequences of IR with large impact on graft function and outcome. Cluster of differentiation 83 (CD83) play a significant role in antigen presentation or cellular interactions following lymphocyte activation. The chemokine (C-C motif) ligand 2 (CCL2) is also referred to monocyte chemoattractant protein-1 (MCP1). CCL2 recruits monocytes, memory T cells and dendritic cells to inflammation sites related to tissue injury^21^. CCL26 is expressed on endothelial cell surface^22^ and inhibits CCL2 mediated response^23^. Here, CCL2/CCL26 expression ratio during cold storage is in favour to CCL2. GATA-3 is a transcriptional activator which binds to the T-cell receptor genes enhancer and is required for T-helper 2 (Th2) differentiation process following immune and inflammatory responses^24^. Toll-like receptor 4 (TLR4) plays a fundamental role in damage-associated molecular patterns (DAMPs) recognition and innate immunity activation. Upregulation/release of DAMPs molecule, exacerbates renal IRI by stimulating inflammatory and immune responses through TLR4 signaling pathway^25,26^. Thus, TLR4 RNA upregulation could be associated with innate immune response stimulation. Zinc finger, AN1-type domain-5 (ZFAND5, also called ZNF216), inhibits TNF, IL-1 and TLR4-induced NF-kappa-B activation^27^. Mothers against decapentaplegic homolog-6 (SMAD6) acts as negative mediator of TGF-β and BMP antiflammatory activity, preventing NF-kappa-B activation^28^. In our study, SMAD6 expression downregulation (except in CMJ WI + CS24h) is in favour of fibrosis and inflammatory pathways. B-cell lymphoma-6 protein (BCL6) modulates STAT-dependent Interleukin 4 (IL-4) responses of B cells. During cold storage, BCL6 upregulation leads to the differentiation of naive helper T cells in Follicular Helper T cells^29^. Our data suggest that upregulation of TNF receptor-associated factor-3 (TRAF3), could represent a novel mechanism for preserving the functional integrity of the endothelial monolayer^30^. This protein is involved in the signal transduction of CD40, a TNFR family member important for the activation of the immune response. Immediate early response-3 (IER3) is member of the NUPR1/RELB/IER3 survival pathway^31^. Nevertheless, IER3, which is here down-regulated during WI, plays a complex and to some extent contradictory role in cell cycle control and apoptosis^32^. Transmembrane protein-14A (TMEM14A) inhibits apoptosis via negative regulation of the mitochondrial outer membrane permeabilization involved in apoptotic signaling pathway^33^. Herein, except CD83 and IER3, inflammation and apoptosis genes were all regulated during cold ischemia without differential expression between cortex and CMJ. These markers expression are evidences of an important immune response enhancing due to ischemia-induced stress.
4. “**Cell cycle**, **cellular differentiation and proliferation**” such as CEBPA, ADIPOQ, EGR1, COL1A1, ANXA3, ANGPTL4, ADAM9, VAV3, DYRK2, JUN, FOS, CYR61, OAS, CDC42EP1 and KLF4 (Fig. 4). Phenotypic alterations due to ischemia resulted in cellular differentiation and proliferation pathway regulation. CCAAT/enhancer-binding protein alpha (CEBPA) is a transcription factor which coordinates proliferation arrest and differentiation^34^. To modulate lipogenesis, CEBPA interacts and transcriptionally synergizes with SREBP1 (Sterol regulatory element-binding transcription factor-1) in promoter activation of specific lipogenic target genes^35^. To regulate gluconeogenesis, CEBPA seems to act as FOXO1 coactivator accessing to Adiponectin (ADIPOQ) promoter^36^. ADIPOQ regulates glucose regulation and fatty acid oxidation^37^. Early growth response protein 1 (EGR1) is a transcriptional regulator^38^ playing a role in cell differentiation, survival, proliferation and death. It regulates the transcription of numerous target genes, and thereby regulates the response to growth factors, DNA damage and ischemia^39^. Here, upregulation of EGR1 and CEBPA greatly increase plasminogen activator inhibitor-1 transcriptional response in hypoxia independently of HIF1-alpha^40^. Collagen alpha-1(I) chain (COL1A1) is a major constituent of the connective tissue, interacting with platelet-derived growth factor-B (PDGFB) and von Willebrand factor^41^, which act respectively on angiogenesis and hemostasis. Annexin A3 (ANXA3) is a inhibitor of phospholipase A2, cleaves inositol 1,2-cyclic phosphate to form inositol 1-phosphate and also possesses anti-coagulant properties. ANXA3 is expressed on healthy epithelial cells^42^ and neutrophils granules^43^. Studies show that ANXA3 enhances hypoxia-inducible factor-1 (HIF-1) transactivation activity and acts as angiogenic factor inducing VEGF production through HIF-1 pathway^44^. Angiopoietin-like-4 (ANGPTL4), involved in glucose homeostasis and lipid metabolism regulation, inhibits endothelial cells proliferation, migration, tubule formation and reduces vascular leakage^45^. Disintegrin and metalloproteinase domain-containing protein 9 (ADAM9) plays a role on angiogenesis^46^ and mediates cell-cell and cell-matrix interactions^47^. VAV3 is a guanine nucleotide exchange factor for Rho family GTPases that activate pathways leading to actin cytoskeletal rearrangements and integrin-mediated signalling. VAV3 is regulated during cell cycle^48^ and promots angiogenesis. Dual specificity tyrosine-phosphorylation-regulated kinase 2 (DYRK2) presumed to be involved in cellular growth and/or development^49^. The transcription factor c-FOS is a proto-oncogene and hence referred to be an immediate early gene expressed after stimuli^50^. It encodes a 62 kDa protein, which forms heterodimer with c-JUN, resulting in the formation of Activator Protein-1 (AP1, also called JUN)^51^ and is involved in cell proliferation, differentiation and survival associated with hypoxia and angiogenesis^52^. Alltogether, upregulation of COL1A1, ADAM9, VAV3, DYRK2, JUN and c-FOS as well as downregulation of ANGPTL4 are in favour of an angiogenic response due to ischemia.The interferon-induced 2′-5′-oligoadenylate synthetases (OAS)^53^ play a role in cellular processes such as apoptosis, cell growth, differentiation and gene regulation. Cell division control protein-42 homolog effector protein-1 (CDC42EP1), encodes CDC42 which have an essential role in organism survival, growth and development^54^. CDC42 activity in primary cells displayed a slow proliferation rate by modulating the JNK-mediated apoptotic machinery^55^. Kruppel-like factor-4 (KLF4) is involved in the regulation of proliferation, differentiation and apoptosis. KLF4 is highly expressed in non-dividing cells and its overexpression induces cell cycle arrest^56^. KLF4 mediates p53-dependent G1/S cell cycle arrest in response to DNA damage^57^, preventing entry into mitosis^58^. In our study, it was demonstrated that CDC2 (other cell division control protein) kinase measurements showed an inverse correlation between CDC2 kinase activities and KLF4 levels^58^. Most of “cell cycle, cellular differentiation and proliferation” genes, as ADIPOQ, EGR1, COL1A1, JUN, FOS and KLF4, were more regulated in CMJ than in cortex after WI. However, cold ischemia time seems to reduce this differential expression. It is interesting to note that the expression of two “immediate early” genes, EGR1 and c-FOS, have been described to be involved in response to renal ischemia after 30 min of WI^59^.
5. “**Nucleus genes and transcriptional regulation**” such as HIRA, HIST1H2AB, HIST1H4A, AURKB, pSNORD3, NR1H4, LEO1 and DICER1 (Fig. 5A). Histone cell cycle regulator (HIRA) is required for the periodic repression/regulation of histone gene transcription during cell cycle^60^. Histone H2A (HIST1H2AB), Histone H4 (HIST1H4A) and Aurora-B kinase (AURKB) are important for DNA regulation and replication. AURKB is a key regulator of mitosis^61^ interacting with histones. Small nucleolar RNA C/D box 3 cluster (pSNORD3 cluster snoRNA) is associated with RNA methylation. Nuclear Receptor Subfamily 1H4 (NR1H4) is a ligand-activated transcription factor. RNA polymerase-associated protein LEO1 (upregulated during cold-stoarge) is a component of the PAF1 complex (PAF1C) which has multiple functions during transcription and is implicated in regulation of development and maintenance of embryonic stem cell pluripotency and required for transcription of Hox and Wnt target genes^62^. Finally, Dicer-1 ribonuclease type-III (DICER1) is involved in mi-RNA production and its inhibition triggers resistance of tubular cells in a mouse model of kidney IRI^63^. This gene appears as upregulated in our model during cold storage, showing the importance of RNA interference contribution during ischemic stress.
6. **“Transporters”** such as ABCC2, ATP2B1, KCNIP4, KCNJ10, MCOLN3 and AQP3 (Fig. 5B). Organ preservation induces membrane tansporters activation to “physiologic balance” reestablishement. ATP-binding cassette (ABC) transporters, like ABCC2, utilize the energy of ATP binding and hydrolysis to transport various substrates across cellular membranes. ABCC2, also called multidrug resistance protein MRP2, acts as an ATP-dependent conjugate export pump in apical membranes of polarized cells^64^. Plasma membrane calcium-transporting ATPase-1 (ATP2B1) is a magnesium-dependent enzyme catalyzing the hydrolysis of ATP coupled with calcium transport out of the cell. Potassium voltage-gated (Kv) channel interacting protein-4 (KCNIP4) is a subunit component of native Kv4 channel complexes. ATP-sensitive inward rectifier potassium channel-10 (KCNJ10) has a greater tendency to allow potassium to flow into the cell. Regulation of these RNA during cold storage for ABBC2, ATP2B1, KCNIP4 and KCNJ10 could be due to ion balance of the preservation solution and ATP availability. Mucolipin-3 (MCOLN3) is an inwardly-rectifying cation channel^65^ which mediates Ca^2+^ release from endosomes to cytoplasm (contributing to endosomal acidification) and is involved in the regulation of membrane trafficking and fusion in the endosomal pathway^66^. Here, ATP2B1 and MCOLN3 are upregulated during cold storage due to Ca^2+^ trafficking deregulation, one major consequence of ischemia^67^. Aquaporin-3 (AQP3) provides kidney medullary collecting duct with high permeability to water, thereby enabling water toward an osmotic gradient. In our study, during WI and in response to water deprivation, AQP3 expression increases in kidney cortex and medulla^68^.
7. “**Metabolism regulation**” such as MTHFD1, LRCH1, ACBP, ACADVL, HADHA, CPT2, FUT2, CKM, ARG2 and PDK4 (Fig. 6). Ischemia alters cell metabolism, however the full range of alterations remains to be defined. Methylenetetrahydrofolate dehydrogenase-1 (MTHFD1) gene encodes the C-1-tetrahydrofolate synthase cytoplasmic protein which is involved in the pathway of tetrahydrofolate interconversion. Leucine-rich repeat and calponin homology domain-containing protein-1 (LRCH1) prevents CDC42 activation and negatively regulates CD4^+^ T-cell migration^69^. Acyl-CoA-binding domain-containing protein (ACBP) likely participates in intermembrane lipid transport from the ER to the plasma membrane, where it could maintain a membrane-associated acyl pool^70^. Acyl-CoA dehydrogenase very long chain (ACADVL) is targeted to the inner mitochondrial membrane, where it catalyzes the first step of the mitochondrial fatty acid beta-oxidation pathway^71^. Hydroxyacyl-CoA dehydrogenase trifunctional multienzyme complex subunit alpha (HADHA) catalyzes the last three steps of mitochondrial beta-oxidation of long chain fatty acids^72^. Carnitine O-palmitoyltransferase-2 (CPT2) oxidizes long-chain fatty acids in the mitochondria^73^. CPT promotes the binding of Acyl-CoA to Carnitine. ACBP, ACADVL, HADHA and CPT2 are mostly dowregulated during cold ischemia likely due to regulation of fatty-acids beta-oxydation. Fucosyltransferase-2 (FUT2) mediates glycosylation of cell surface glycoproteins and glycolipids^74^. Glycosylation, acts in rough endoplasmic reticulum, induces tissue aging but also may have protective effects^75^. Our results show that FUT2 is upregulated during cold storage-induced ischemia. Creatine kinase M-type (CKM) play a central role in energy transduction. CKM regenerates ATP from ADP, using phosphocreatine^76^. Creatine kinase is a marker of damage of CK-rich tissue such as in acute kidney injury^77^. We observed that CKM is expressed only during cold ischemia. Arginase-2 (ARG2) has multiple fonctions as it play a role in the regulation of polyamine metabolism and also in down-regulation of nitric-oxide synthesis, it is involved in the negative regulation of the survival capacity of activated CD4^+^ and CD8^+^ T cells^78^ and it inhibit endothelial autophagy independently of its enzymatic activity implicating mTORC2 signaling^79^. Blocking ARG2 expression attenuated lesions in a mice model of IRI^80^. Our results are comforting these observations and the dowregulation of ARG2 seems to be protective. Pyruvate dehydrogenase lipoamide kinase isozyme-4 (PDK4) is located in the mitochondria matrix and inhibits the pyruvate dehydrogenase complex by phosphorylating one of its subunits, reducing the conversion of pyruvate, which is produced from the oxydation of glucose and amino acids to acetyl-CoA and contributing to glucose metabolism regulation. PDK4 helps to decrease metabolism and conserve glucose by limiting its conversion to acetyl-CoA, which enters the citric acid cycle and is converted to ATP^81^.
8. “**MAPK and GTPase activity”** such as RABGAP1, PARP4, MAP3K8, MLK4, MAP3K5, RHOB, PLK3, MX2, RHOU, JUN and EXPH5 (Fig. 7). Ischemia activates intracellular enzymes as G Proteins and MAPK to regulate several pathways. CDC42 effector protein 1 (CDC42EP1) is a member of the Rho GTPase family regulating multiple cellular activities, including organization of the actin cytoskeleton^82^. Rab GTPase-activating protein-1 (RABGAP1), upregulated herein during cold storage, acts as a GTPase-activating protein in the RAB6A-mediated pathway involved in the mitotic metaphase-anaphase transition^83^. Poly [ADP-ribose] polymerase-4 (PARP4) is able to catalyze a poly(ADP-ribosyl)ation reaction. PARP4 interacts with Major vault protein, which interacts with the inactive PERK (protein involved in endoplasmic reticulum stress). Mitogen-activated protein kinase-kinase-kinase-8 (MAP3K8, also called TPL-2) can activate both the ERK1/2 and p38 MAP kinases^84^. MAP3K8 regulates renal cell apoptosis in ischemia/reperfusion injury^85^. Herein, MAP3K8 is dowregulated from WI to end of cold storage. Mitogen-activated protein kinase kinase kinase 21 (MAP3K21 also called MLK4), herein downregulated during cold storage, is a negative regulator of TLR4 signaling^86^. This is in favour of the TLR4 sigaling activation, one of the major pathway involved during IRI^87^. Thus, activation of MLK4 could be an interesting target for new therapy. Mitogen-activated protein kinase kinase kinase 5 (MAP3K5), also called apoptosis signal-regulating kinase-1 (ASK1), is pivotal component in cell apoptosis and can be activated by a variety of death stimuli including TNF-alpha and oxidative stress^88^. Herein, the downregulation of MAP3K5 during cold ischemia is protective. Ras homolog gene family member-B (RHOB), upregulated during WI, enhances cytokine-induced transcription of inductible nitric-oxide synthase-2 (iNOS)^89^ inducing a oxidative environment. PLK3, upregulated from WI to cold storage end, is involved in cell cycle regulation, response to stress, Golgi disassembly and DNA damage response^90^. PLK3 is rapidly activated upon stress stimulation, such as ROS, hyperosmotic stress and hypoxia. PLK3 is important for the downregulation of apoptosis and regulation of microtubule dynamics and centrosomal function^91^. Interferon-induced GTP-binding protein MX2, also dowregulated during cold storage, regulates nucleocytoplasmic transport and cell-cycle progression^92^. Rho-related GTP-binding protein RHOU is encoded by a non-canonical Wnt induced gene^93^. RHOU/Wrch delineates with RhoV/Chp a Rho subclass related to RAC and CDC42, which emerged in early multicellular organisms during evolution^94^. Similarly to CDC42, RHOU and Exophilin-5 (EXPH5) are also dowregulated during cold storage. EXPH5 may act as Rab effector protein and play a role in intracellular vesicle trafficking. Reduced expression of this gene results in keratin filament defects, in association with collagen structure.

**Figure 8:**
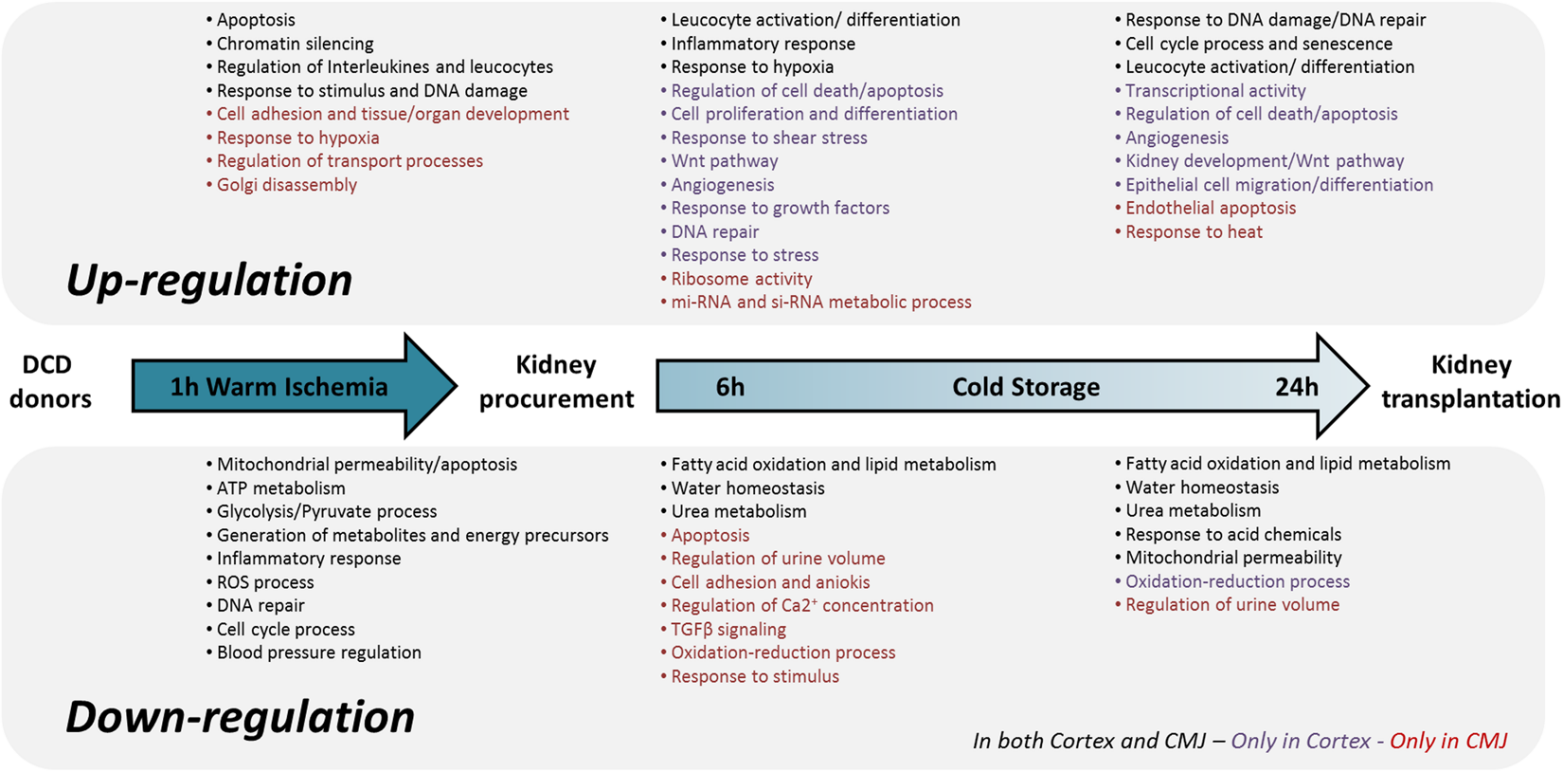
Proposed summary of relevant cortex and CMJ up and down regulated gene categories during ischemic temporal sequence mimicking DCD donor kidney.

We summarized the kinetic of altered pathways during the experiment timecourse in Fig. 8, taking into account the Gene-Ontology Analysis and our data interpretation in the context of renal ischemia. Several gene families are up- or down-regulated similarly in both cortex and CMJ, but we noted some differences of expression among renal regions at specific timepoints, as reported in Fig. 8.

Alltogether, our study highlight critical genetic alterations induced by ischemia at the cellular/tissular levels, dissecting ischemia mechanism and kinetics using an experimental model extremely close to human conditions. Several of these pathways have “opposite” roles (e.g.: survival/development versus apoptosis/death) which may be linked to the high stress level withstood by the kidneys in our experiments resulting to highly complex responses at the cellular level aimed at counterbalancing stress-induced lesions. Beyond improving our understanding of IRI, our study point out several dysregulated genes which could be used as biomarkers of ischemia injury, allowing a thinner evaluation of kidney quality, one of the major challenges in renal transplantation. In order to validate their quality as biomarkers, further studies are required to evaluate the protein expression level in kidneys subjected to different levels of ischemic injury and correlate these data to kidney transplantation outcome. In conclusion, our data strengthen the fact that ischemia is a key step during the transplantation process with important transcriptional modifications inducing a full reprogramming of the transcriptome of major pathways such those related to oxidative stress responses, cell reprogramming, cell-cycle, inflammation and cell metabolism. These pathways provide interesting research prospects for the development of strategies which could be used during kidney conservation, aimed at improving whole transplantation outcome.

## Methods

### Animal experimentation

The animal experimental protocol was approved by French Government and institutional Committee on the Ethics of Animal Experiments of the Poitou-Charentes (France) (comity number C2EA-84, protocol number: CE2012-4). Experimentations were performed in accordance with EU Directive 2010/63/EU at the IBiSA MOPICT platform, INRA Magneraud, France. Full methods for animal experimentation are provided in Suplementary Material and Methods.

### Microarrays slides

Porcine Gene Expression Microarray G2519F (Agilent) contains 60-mer oligonucleotide probes to 43,803 porcine probes for the pig *Sus Scrofa*. The data discussed in this article have been deposited in NCBIs Gene Expression Omnibus Database (GEO), accessible through accession number GSE109719^95^.

### RNA Isolation

RNA was extracted using a commercial kit including a DNAse step to remove genomic DNA (Qiagen RNeasy plus mini). The RNA yield and integrity were controlled using a Nanodrop ND-1000 and a Bioanalyzer 2100 Expert from Agilent, then labelled with the Low Input Quick Amp Labeling kit, designed to reliably amplify and label target RNA for the robust generation of complementary RNA.

### Hybridizations

Hybridization was performed following the One-Color Microarray-Based Gene Expression Analysis Protocol. Assembled slide chamber was placed in rotisserie in a hybridization oven. Hybridization took place during 17 hours at 65 °C and at 10 rpm.

### Slide scanning and image analysis/treatment

The slides were scanned on a Tecan MS200 scanner and analyzed by Feature Extraction V.11.5.1.1. Pre-analysis data quality assessment was performed by visual inspection of individual false color hybridization images and standard diagnostic plots of probe level intensity distributions using Bioconductor (http://www.bioconductor.org/) and R software (version 2.15.2). All data were analyzed using the Bioconductor software project and the statistical language R. After transformed log2 data, the data were normalized by condition using quantile method^96^ with limma package^97^ and finally summarized. Significant genes were identified using the limma package. A False Discovery Rate corrected p-value < 0.05 and a log2 fold change >0.5 were used as significance criterion.

### Heatmap

The heatmap was generated with R software using Euclidean distance and Ward linkage from the list of differentially expressed genes between Ischemia and Control.

### Functional enrichment analysis

The detection of significantly overrepresented Gene Ontology categories was performed using the GOStats package in Bioconductor^98^. The list of differentially expressed genes was divided in two parts: up-regulated and down-regulated genes. For each sublist, we performed a hypergeometrical statistical test; corresponding to the ratio of differentially expressed genes found in the category over the total number of genes in the category compared to the ratio of the total number of differentially expressed genes over total number of genes on the chip.

### Real-Time Quantitative-PCR confirmation

Full methods for Real-Time Quantitative-PCR are provided in Supplementary Material and Methods.

## Electronic supplementary material

Supplementary information
