# Supplementary material for: Dynamic transcriptomic analysis of Ischemic Injury in a Porcine Pre-Clinical Model mimicking Donors Deceased after Circulatory Death
Source: Sci Rep. 2018 Apr 13;8:5986. doi: 10.1038/s41598-018-24282-6 (PMC5899088; doi:10.1038/s41598-018-24282-6)
Supplement: Supplementary file 1 — Supplementary information [file 41598_2018_24282_MOESM1_ESM.pdf]

Dynamic transcriptomic analysis of Ischemic Injury in a Porcine Pre-Clinical Model mimicking Donors Deceased after Circulatory Death

Sebastien Giraud [1,2,3,†], Clara Steichen [1,2,†], Geraldine Allain [1,2,4], Pierre Couturier [1,3,5], Delphine Labourdette [6], Sophie Lamarre [6], Virginie Ameteau [1,2], Solenne Tillet [1,2], Patrick Hannaert [1], Raphael Thuillier [1,2,3], Thierry Hauet [1,2,3,5,7] *

Department and institution

[1] Inserm U1082 IRTOMIT, Poitiers, F-86000, France.

[2] Université de Poitiers, Faculté de Médecine et de Pharmacie, Poitiers, F-86000, France.

[3] CHU Poitiers, Service de Biochimie, Poitiers, F-86000, France.

[4] CHU Poitiers, Service de chirurgie cardio-thoracique, Poitiers, 86000, France.

[5] MOPICT, IBiSA plateforme 'Experimental Surgery and Transplantation', INRA, GENESI, Domaine du Magneraud, Surgères, F-17700, France.

[6] LISBP, Université de Toulouse, CNRS, INRA, INSA, Toulouse, F- 31077, France

[7] FHU SUPORT 'SUrvival oPtimization in ORgan Transplantation', Poitiers, F-86000, France.

† S Giraud and C Steichen have contributed equally to this work

* Corresponding author

Thierry Hauet (MD, PhD), Email: thierry.hauet@gmail.com

Supplementary Material and Methods

Animal experimentation:

The animal experimental protocol was approved by French Government and institutional Committee on the Ethics of Animal Experiments of the Poitou-Charentes (France) (comity number C2EA-84, protocol number: CE2012-4). Experimentations were performed in accordance with EU Directive 2010/63/EU at the IBiSA MOPICT platform, INRA Magneraud, France. We used 3 months-old Large White pigs weighting 40±4 kg. Animal anesthesia was performed with a Hunter mask with a 50/50 mixture of nitrogen protoxyde and oxygen associated with 8% sevoflurane. Animals were scoped and perfused all along the procedure. Before endotracheal tube placement, anesthesia was depth with an injection of propofol 2.5 mg/kg, midazolam 0.5 mg/kg, fentanyl 10 µg/kg and rocuronium bromure 1 mg/kg. Ventilation was monitored with a tidal volume of 10 ml/kg, a respiratory rate set to maintain an End-tidal CO_2 expired between 35 and 45mmHg. Curarization of the animal was performed by reinjections of rocuronium bromure adapted to the behaviour of each animal. Intraoperative analgesia was maintained by continuous infusion of fentanyl 10 µg/kg/h. Anesthesia was maintained by isoflurane 2.5%. Hydro-sodic losses were compensated. Midline abdominal incision was performed to reach the right kidney under anesthesia. The right renal vascular pedicle and the ureter were atraumatically dissected, and the renal pedicle was clamping. Immediately after renal pedicle clamping, the right kidney was removed, maintained clamped for 60 min at 37°C (warm ischemia), rapidly collected and flushing with cold (4°C) University of Wisconsin preservation solution (UW) and stored at 4°C for 6h or 24h in static condition. At the end of each period (cf. experimental design), samples of cortex (C) and corticomedullary junction (CMJ) tissues from kidneys were immediately frozen in liquid nitrogen (N=5). These samples were compared to samples from control non-treated kidneys freshly removed from

independent normal brothers pigs and immediately frozen in liquid nitrogen (control group) (N=5). All kidneys were obtained from 3 month-old independent brother pigs belonging to the same breed submitted to the same anaesthetic protocol and the same surgical procedure.

Experimental design:

Control non-treated kidneys (N=5), cortex tissue (Ctl-C) and corticomedullary junction tissue (Ctl-CMJ).

Kidneys submitted to 60 min of warm ischemia (WI) (N=5), cortex tissue (WI-C) and corticomedullary junction tissue (WI-CMJ).

Kidneys submitted to 60 min of WI + 6h cold storage (N=5), cortex tissue (WI+CS6h-C) and corticomedullary junction tissue (WI+CS6h-CMJ).

Kidneys submitted to 60 min of WI + 24h cold storage (N=5), cortex tissue (WI+CS24h-C) and corticomedullary junction tissue (WI+CS24h-CMJ).

To determine the number of animal required in each group, we performed a covariance analysis based on previous data collected from our swine model in the laboratory. This number of animal per group was set to 5 (n=5); based on a known and ascertain difference (0.55 fold change) and standard error (0.30) on the principal criteria (RNA expression at the end of preservation period), considering an alpha risk of 5%, a power of 80%, on a bilateral hypothesis. This choice is in agreement with the legislation on laboratory animal use and on the need to reduce animal number in experimentation. Furthermore, our swine model is highly reproducible due to the same breed of the animals. All experiments were performed in identical conditions and all samples RNA extraction were performed at the same time with the same material and methods until processing the microarray. Finally, in addition of being often accepted for microarray studies, an n=5 replicate, in our conditions, did not show any

distribution difference and very few variabilities between the samples highlighting the high reproducibility of our microarray study.

Real-Time Quantitative-PCR

Porcine DNA primers were designed using OligoPerfect™ (Invitrogen), QuantPrim (Universität Potsdam, Max-Planck-Gesellschaft) and OligoAnalyser (Integrated DNA Technologies, Inc). Sequences are detailed in Supplementary Table S8. Total RNA were extracted using a commercial kit including a DNAse step to remove genomic DNA (Rneasy plus mini, Qiagen, France). First-strand reverse transcription was performed using a commercial kit (Applied-Biosystems, Life Technologies, Saint-Aubin, France). RT q-PCR was performed on a RotorGene-Q (Qiagen, France). mRNA expression levels in the samples relative to expression in normal kidney were determined with the Pfaffl method (expressed as Relative Fold Change). β-actin, ribosomal protein large P0 (RPLPO), succinate dehydrogenase complex, subunit A (SDHA) and cyclophilin A 62 (CYA62) were chosen as house-keeping genes using the GeNorm algorythm. For RT-qPCR analysis, results are expressed as mean ± SEM (Standard Error of the Mean). For statistical analysis among groups, we used NCSS software (NCSS LLC, USA) to perform Kruskal Wallis + Dunns post test. Statistical significance was accepted for $p<0.05$.

Supplementary information (Figures and tables)

Figures S1 to S10: Heatmaps, using R-software, based on the differentially expressed genes in cortex (C) tissues: WI-C versus Ctl-C (Figure S1), WI+CS6h-C versus Ctl-C (Figure S2), WI+CS24h-C versus Ctl-C (Figure S3), WI+CS6h-C versus WI-C (Figure S4), WI+CS24h-C versus WI-C (Figure S5), and in corticomedullary junction tissues (J): WI-J versus Ctl-J (Figure S6), WI+CS6h-J versus Ctl-J (Figure S7), WI+CS24h-J versus Ctl-J (Figure S8), WI+CS6h-J versus WI-J (Figure S9), WI+CS24h-J versus WI-J (Figure S10). Samples are in columns and expression level for each gene is in rows. Red: over expressed in Ischemia compared to Control. Black: neither over expressed nor under expressed in Ischemia compared to Control. Green: under expressed in Ischemia compared to Control. The samples and genes have been ordered using the *Ward* agglomeration method and a Euclidean *distance* metric.

WI-C versus Ctl-C (Figure S1)

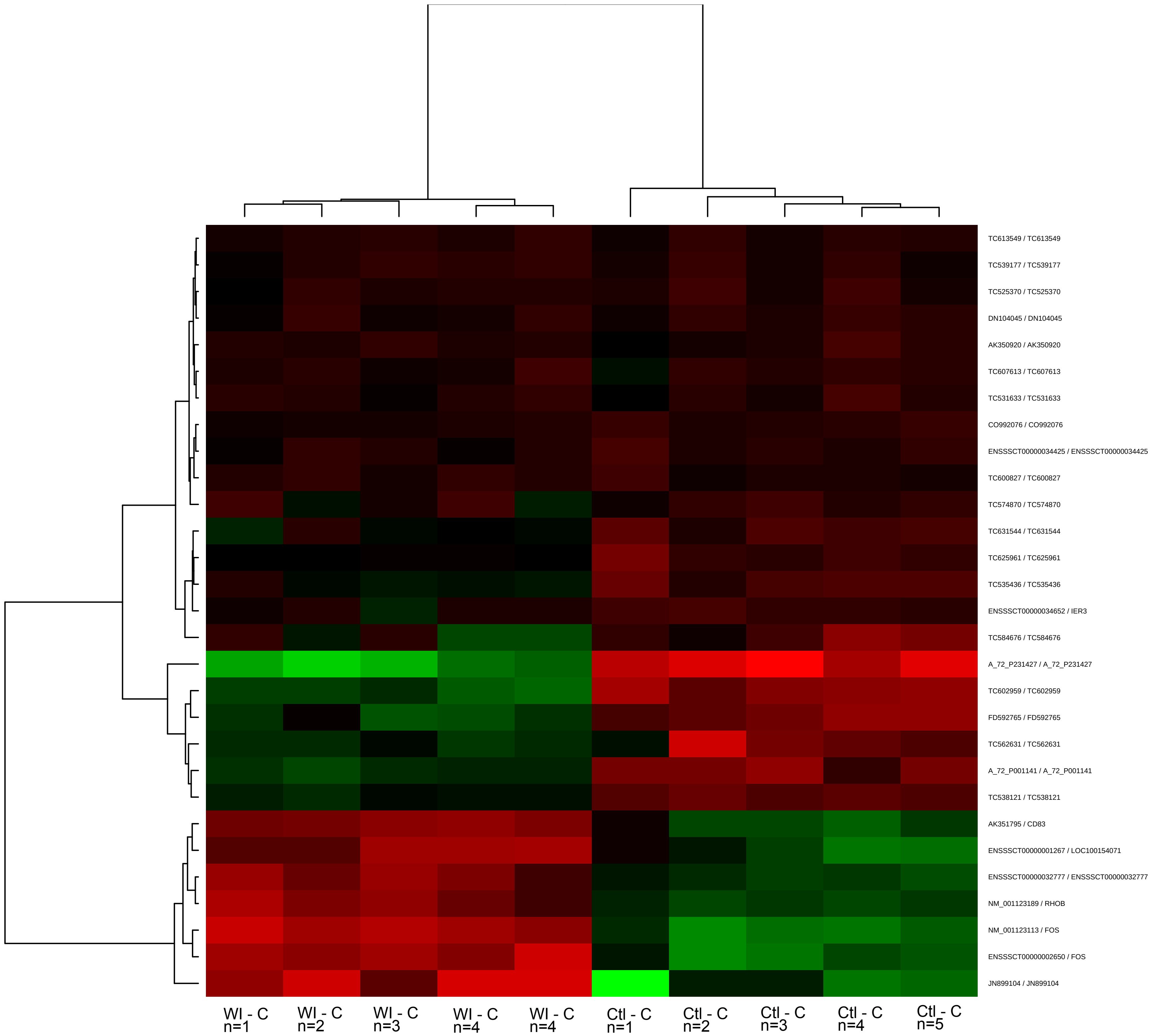
TC613549 / TC613549
TC539177 / TC539177
TC525370 / TC525370
DN104045 / DN104045
AK350920 / AK350920
TC607613 / TC607613
TC531633 / TC531633
CO992076 / CO992076
ENSSSCT00000034425 / ENSSSCT00000034425
TC600827 / TC600827
TC574870 / TC574870
TC631544 / TC631544
TC625961 / TC625961
TC535436 / TC535436
ENSSSCT00000034652 / IER3
TC584676 / TC584676
A_72_P231427 / A_72_P231427
TC602959 / TC602959
FD592765 / FD592765
TC562631 / TC562631
A_72_P001141 / A_72_P001141
TC538121 / TC538121
AK351795 / CD83
ENSSSCT00000001267 / LOC100154071
ENSSSCT00000032777 / ENSSSCT00000032777
NM_001123189 / RHOB
NM_001123113 / FOS
ENSSSCT00000002650 / FOS
JN899104 / JN899104
WI - C n=1
WI - C n=2
WI - C n=3
WI - C n=4
WI - C n=4
Ctl - C n=1
Ctl - C n=2
Ctl - C n=3
Ctl - C n=4
Ctl - C n=5

WI+CS6h-C versus Ctl-C (Figure S2)

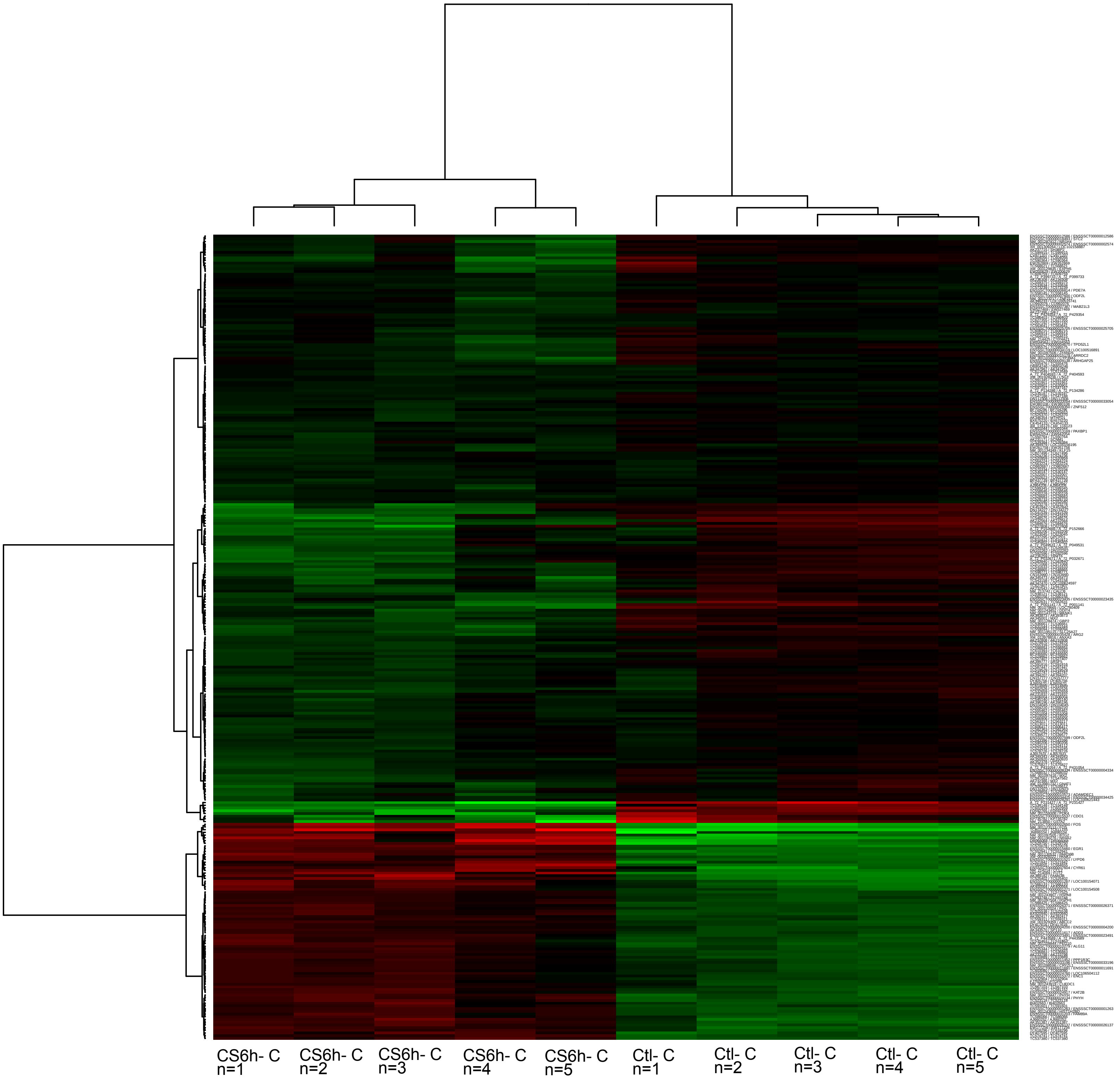
CS6h- C n=1
CS6h- C n=2
CS6h- C n=3
CS6h- C n=4
CS6h- C n=5
Ctl- C n=1
Ctl- C n=2
Ctl- C n=3
Ctl- C n=4
Ctl- C n=5

WI+CS24h-C versus Ctl-C (Figure S3)

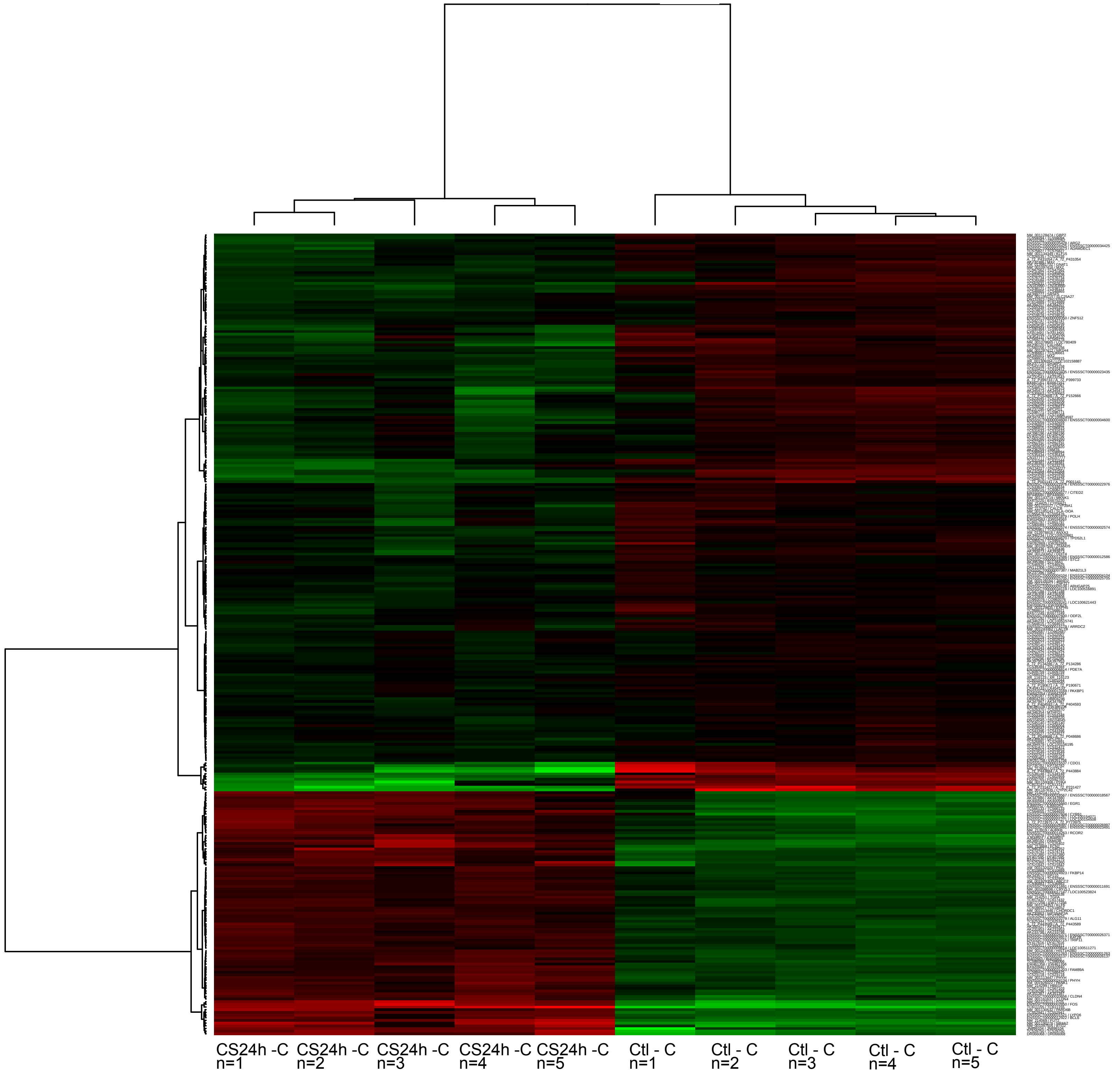
CS24h -C n=1
CS24h -C n=2
CS24h -C n=3
CS24h -C n=4
CS24h -C n=5
Ctl - C n=1
Ctl - C n=2
Ctl - C n=3
Ctl - C n=4
Ctl - C n=5

WI+CS6h-C versus WI-C (Figure S4)

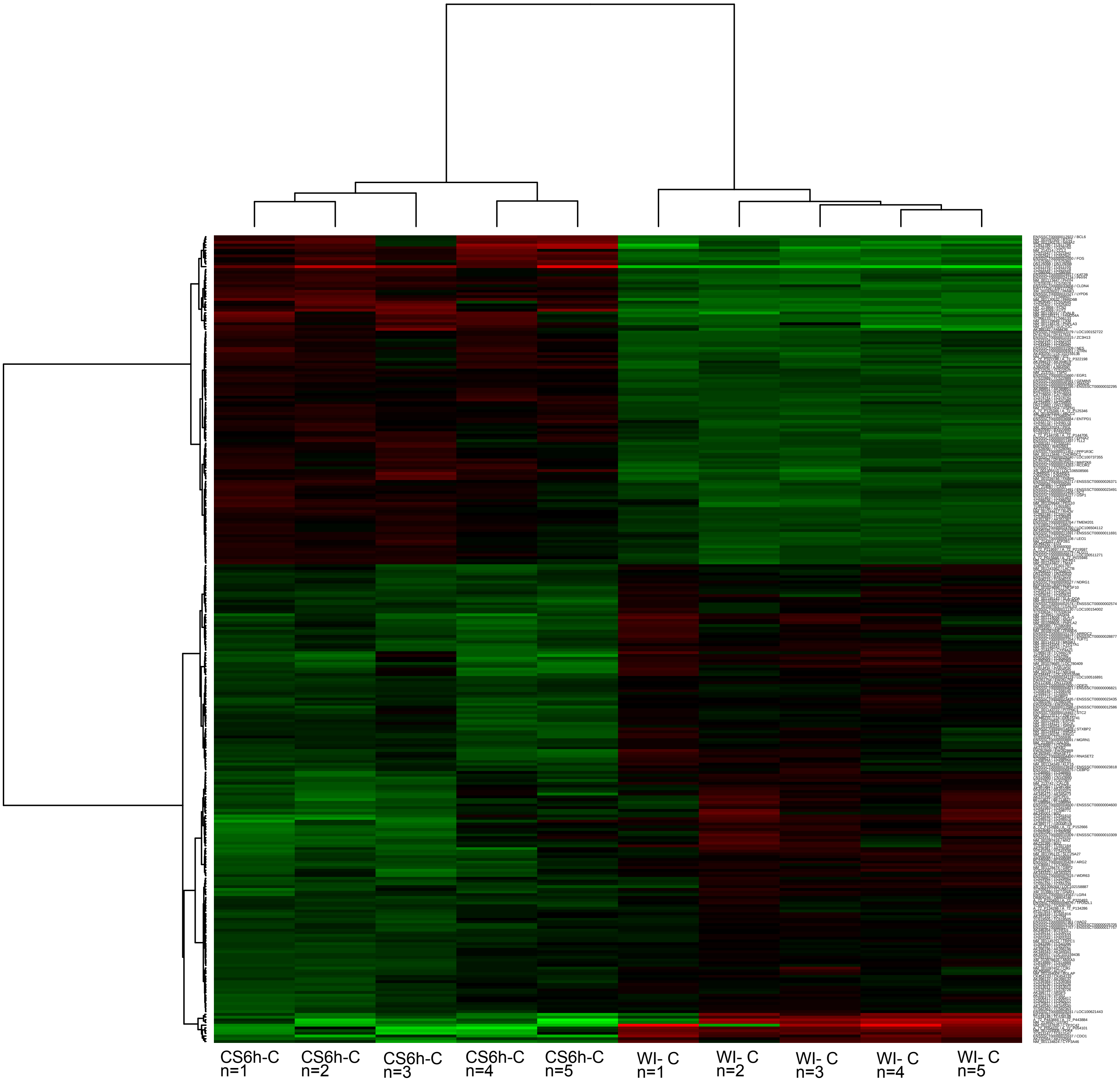
CS6h-C n=1
CS6h-C n=2
CS6h-C n=3
CS6h-C n=4
CS6h-C n=5
WI- C n=1
WI- C n=2
WI- C n=3
WI- C n=4
WI- C n=5

WI+CS24h-C versus WI-C (Figure S5)

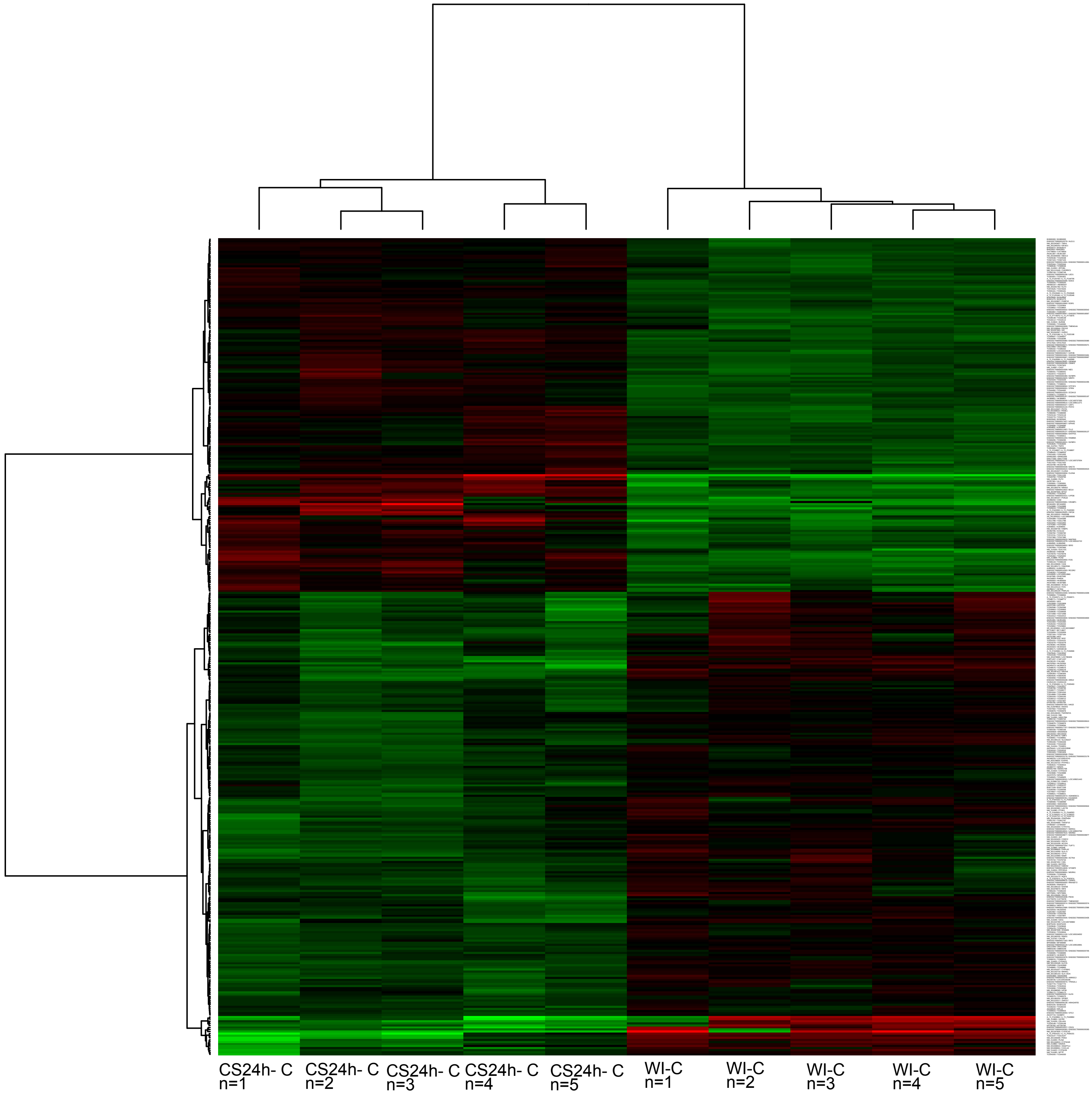

CS24h- C n=1
CS24h- C n=2
CS24h- C n=3
CS24h- C n=4
CS24h- C n=5
WI-C n=1
WI-C n=2
WI-C n=3
WI-C n=4
WI-C n=5

WI-J versus Ctl-J (Figure S6)

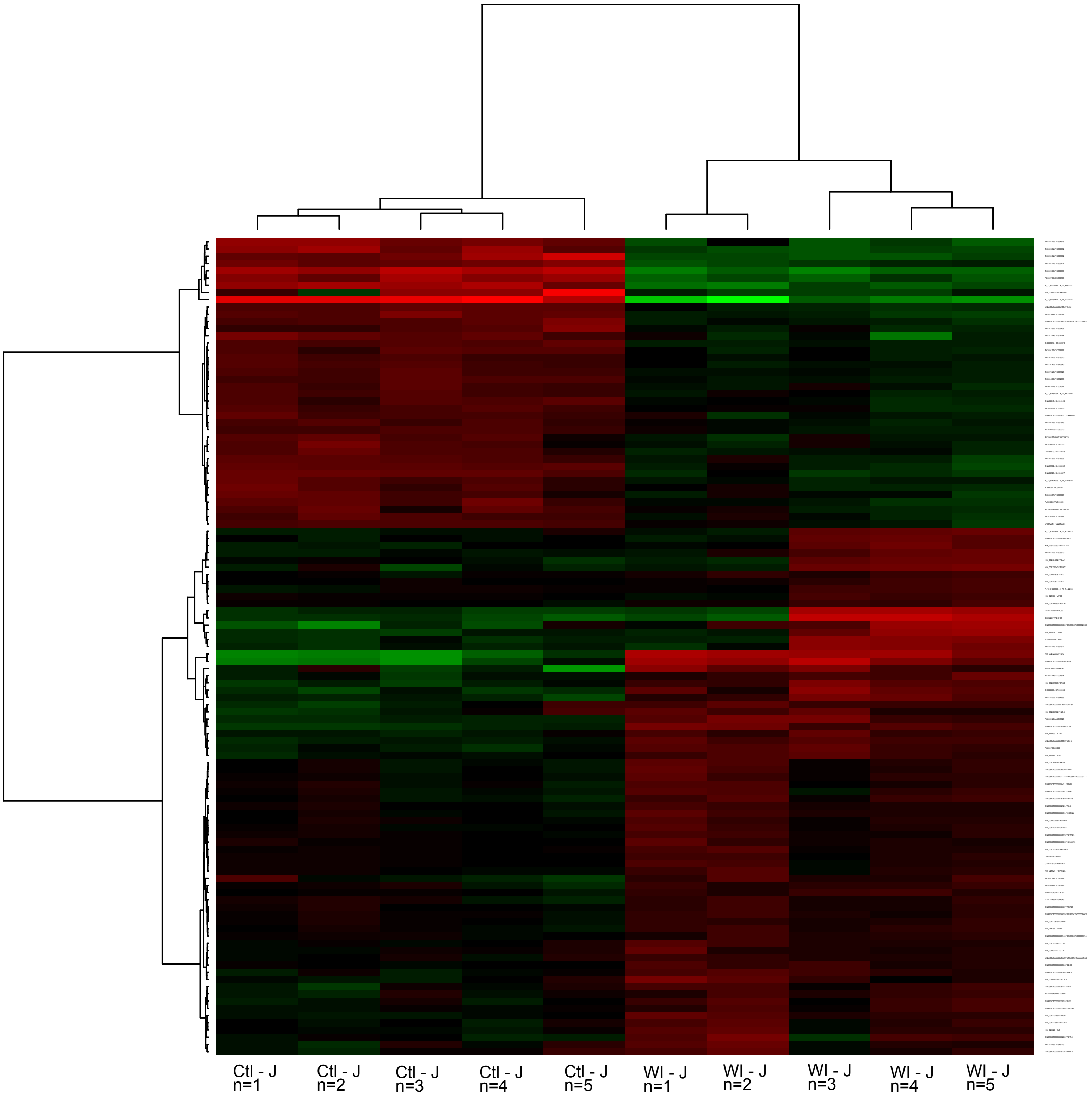
Ctl - J n=1
Ctl - J n=2
Ctl - J n=3
Ctl - J n=4
Ctl - J n=5
WI - J n=1
WI - J n=2
WI - J n=3
WI - J n=4
WI - J n=5

WI+CS6h-J versus Ctl-J (Figure S7)

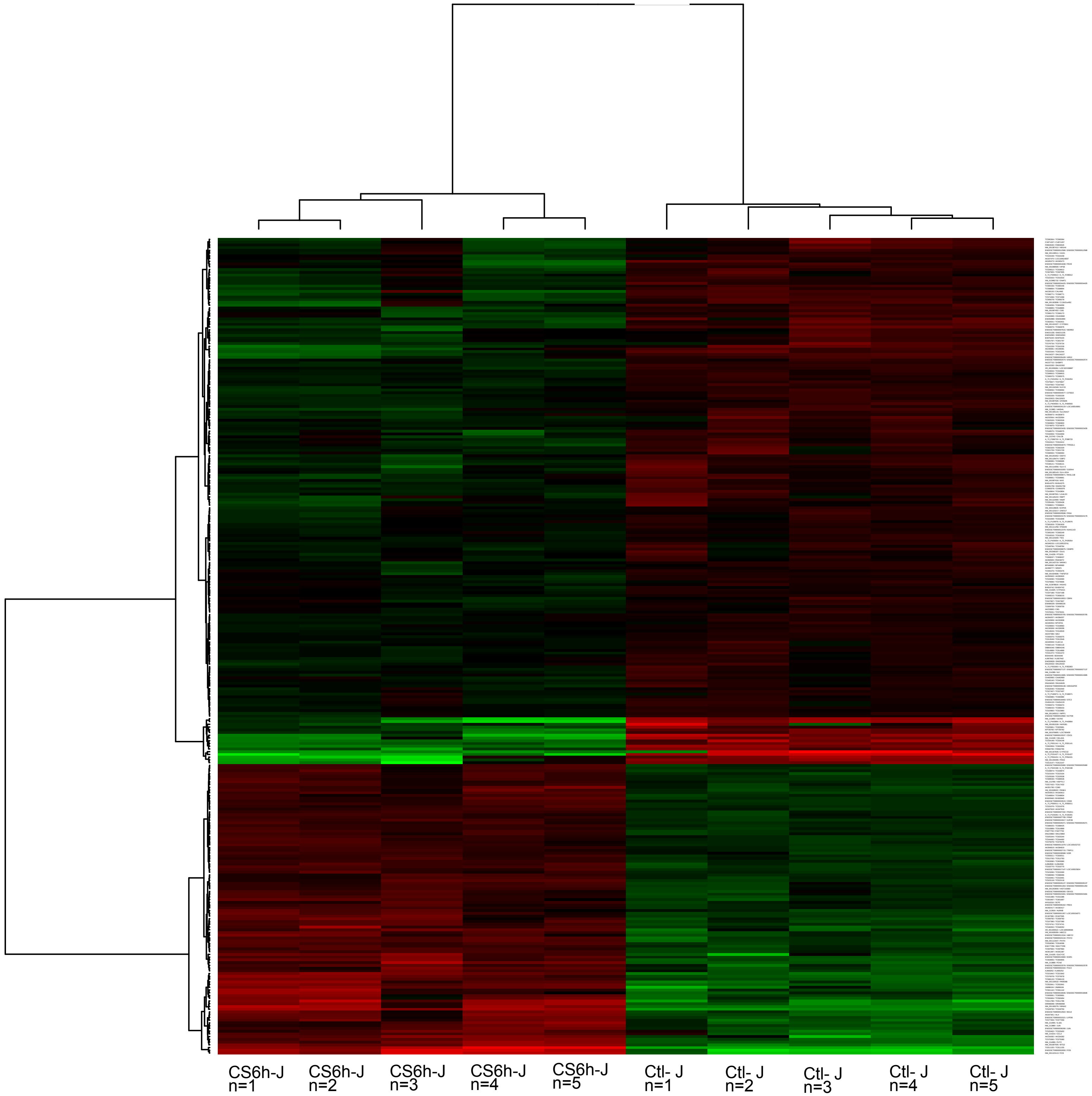
CS6h-J n=1
CS6h-J n=2
CS6h-J n=3
CS6h-J n=4
CS6h-J n=5
Ctl- J n=1
Ctl- J n=2
Ctl- J n=3
Ctl- J n=4
Ctl- J n=5

WI+CS24h-J versus Ctl-J (Figure S8)

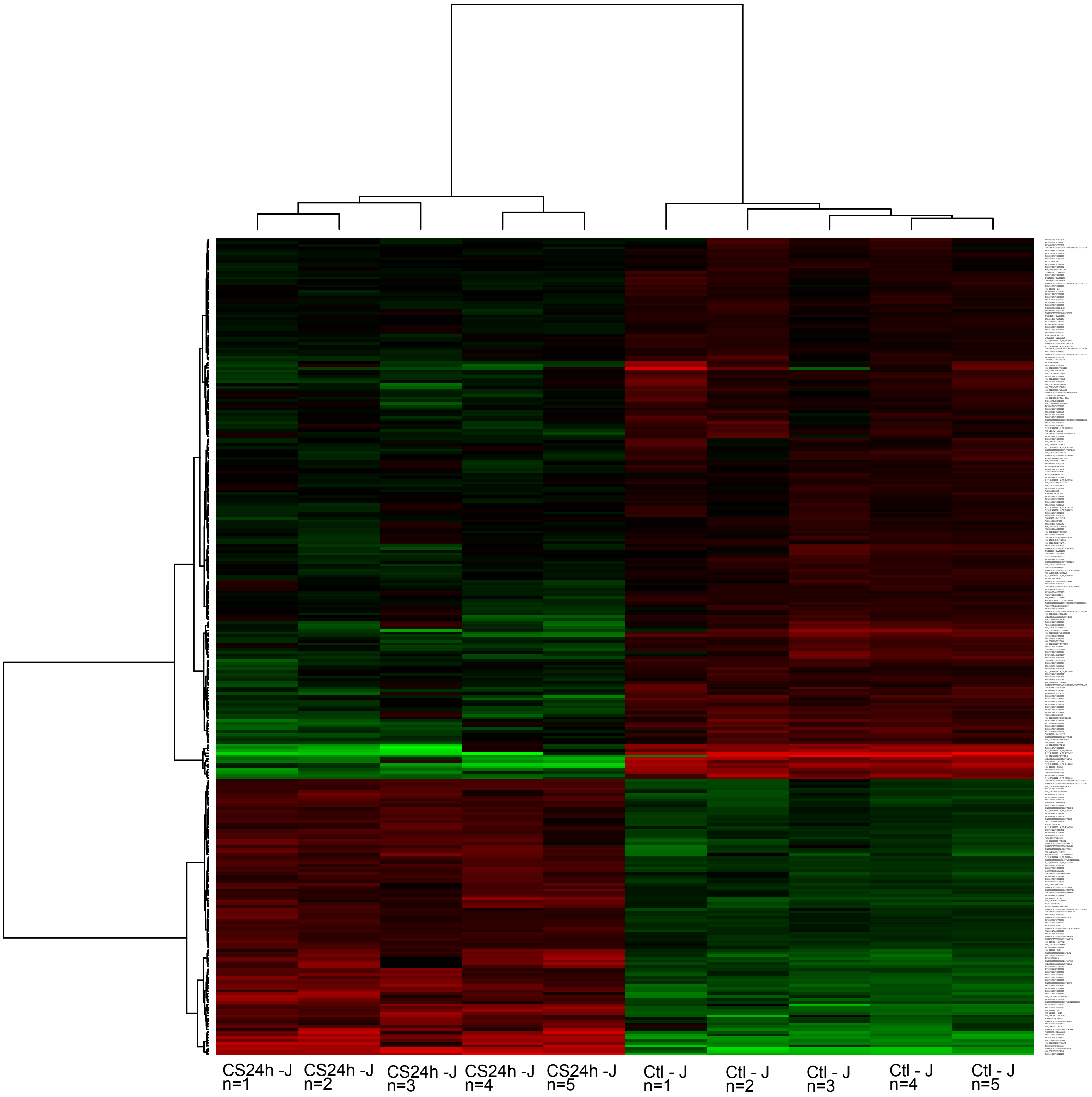
CS24h -J n=1
CS24h -J n=2
CS24h -J n=3
CS24h -J n=4
CS24h -J n=5
Ctl - J n=1
Ctl - J n=2
Ctl - J n=3
Ctl - J n=4
Ctl - J n=5

WI+CS6h-J versus WI-J (Figure S9)

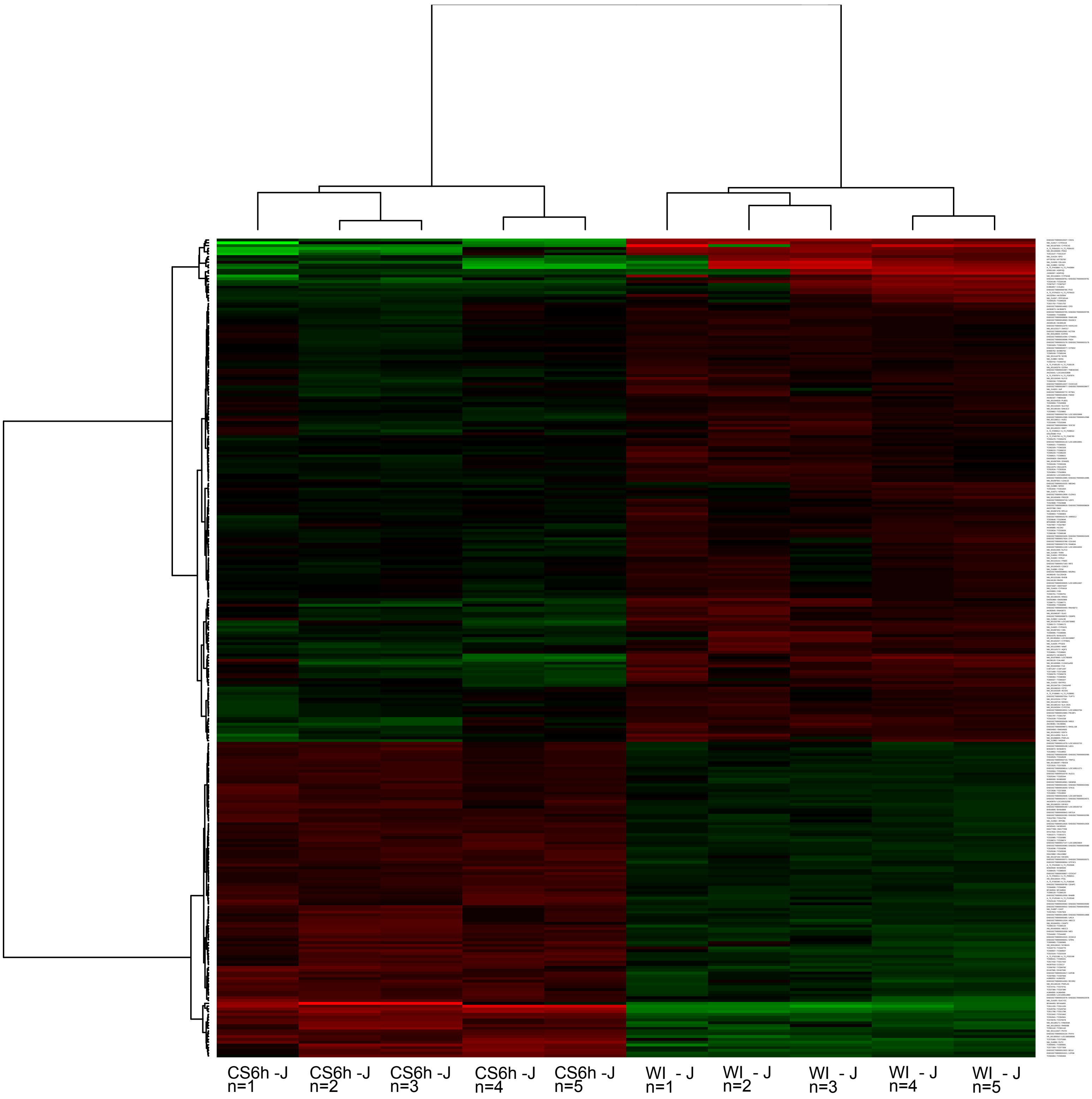
CS6h -J n=1
CS6h -J n=2
CS6h -J n=3
CS6h -J n=4
CS6h -J n=5
WI - J n=1
WI - J n=2
WI - J n=3
WI - J n=4
WI - J n=5

WI+CS24h-J versus WI-J (Figure S10)

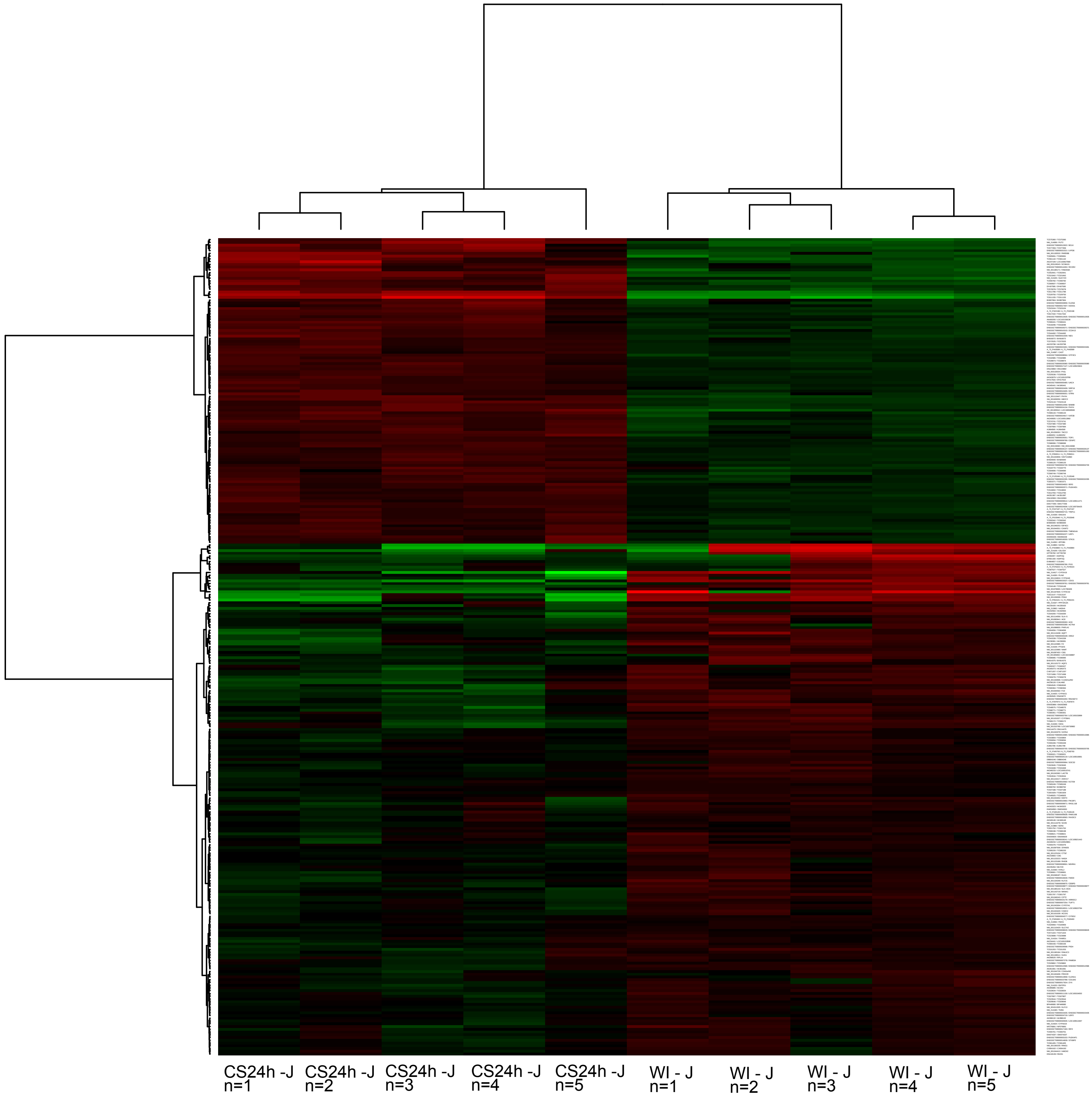

CS24h -J n=1
CS24h -J n=2
CS24h -J n=3
CS24h -J n=4
CS24h -J n=5
WI - J n=1
WI - J n=2
WI - J n=3
WI - J n=4
WI - J n=5

Figure S11: Gene expression correlation between Agilent (from heatmaps) and RT-qPCR results for (A) cortex tissues genes and (B) CMJ tissues genes. Statistics were performed with Spearman r (line) and 95% confidence interval (doted lines) (GraphPad software).

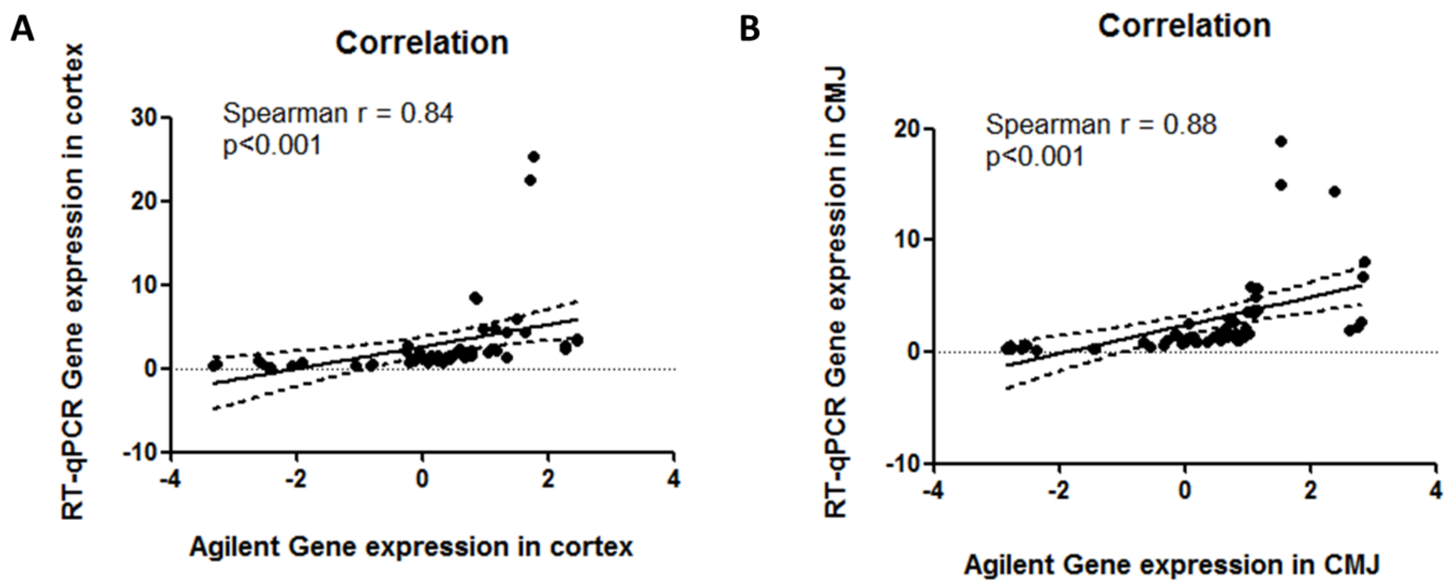

Figure S12: Principal (most significative) GO-BP and GO-MF categories of genes identified by the functionnal enrichment analysis (related to the Supplementary Table S3 to S6).

A: WI-C versus Ctl-C

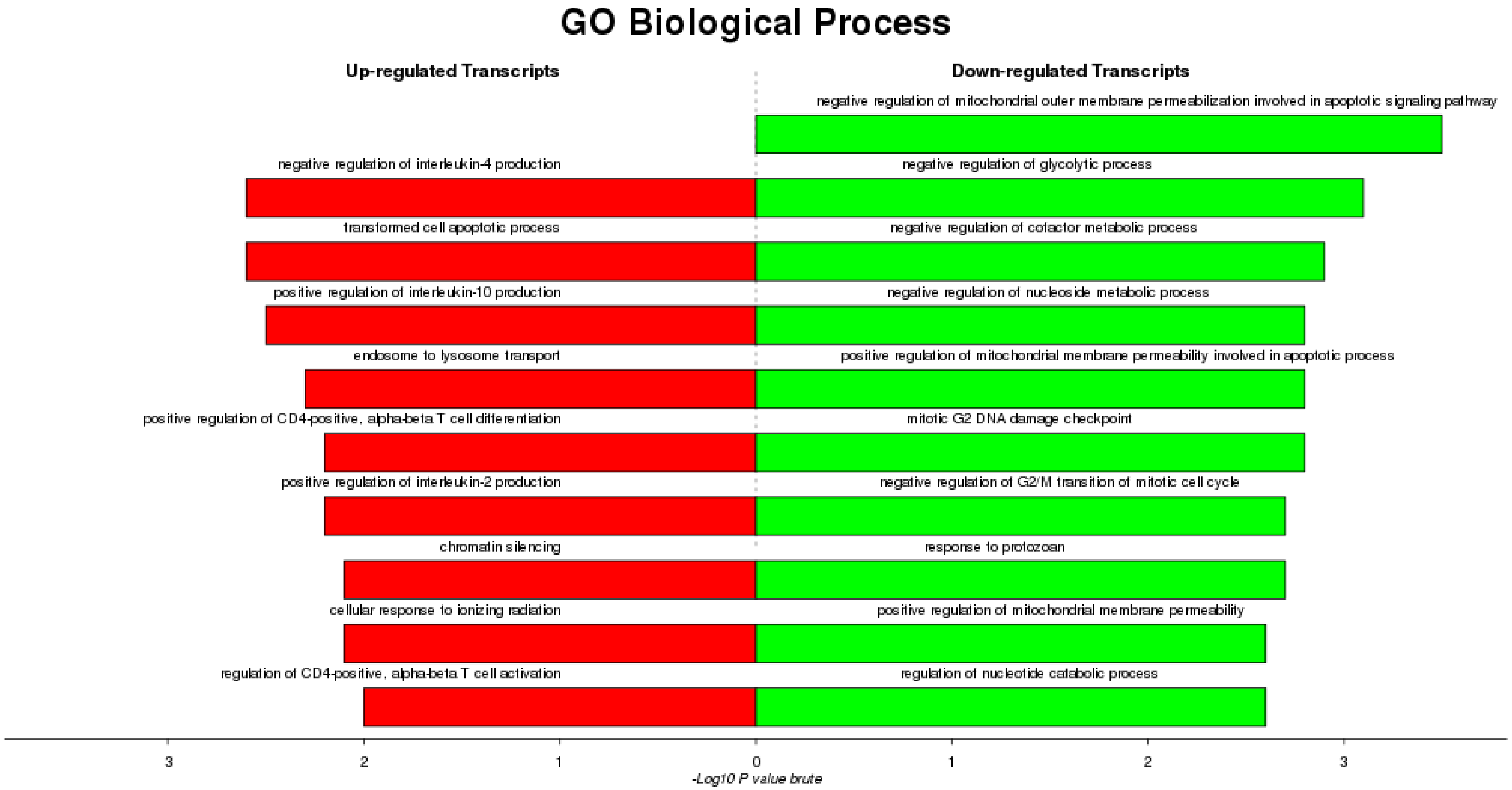

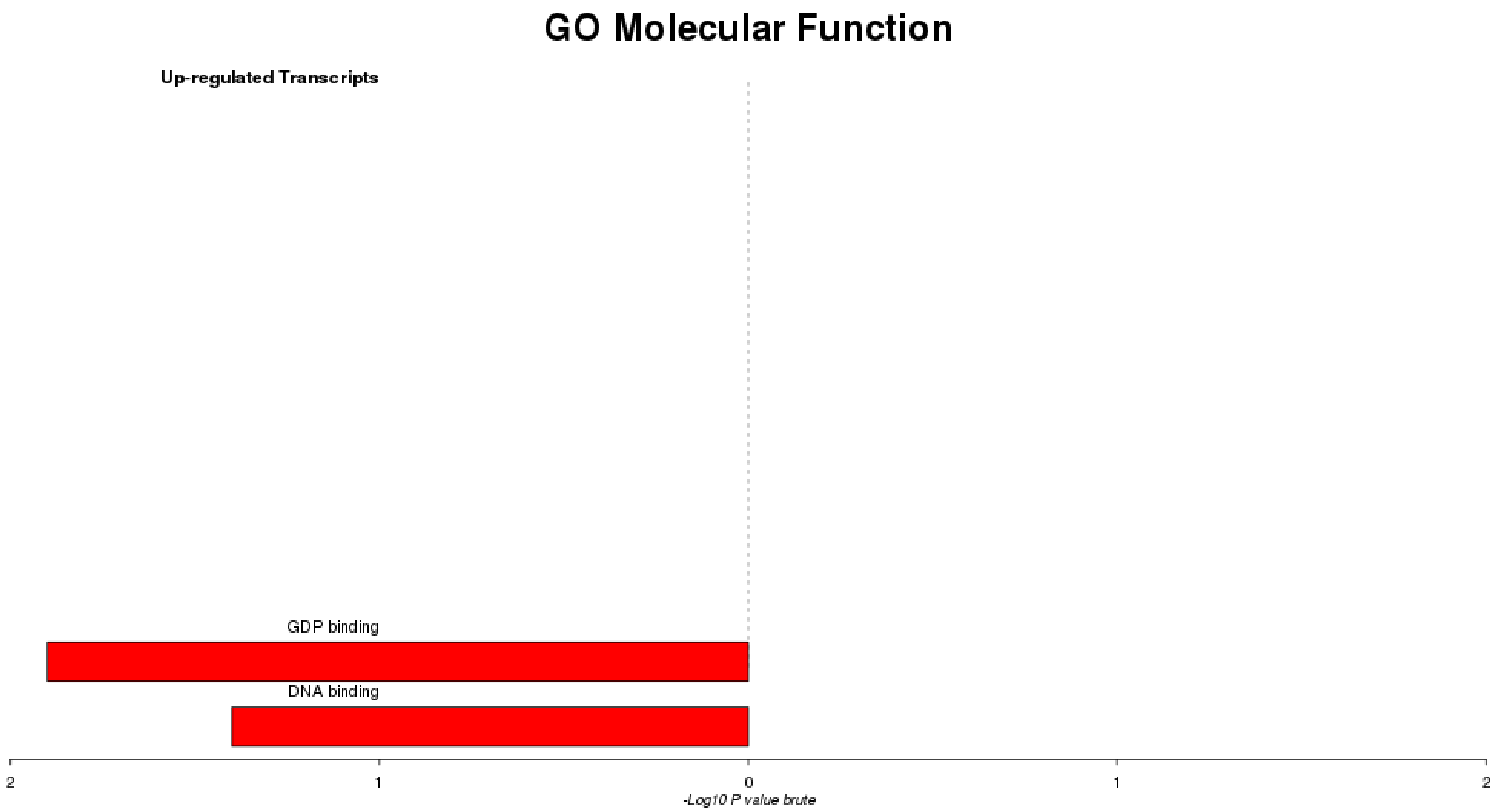
GO Molecular Function
Up-regulated Transcripts
GDP binding
DNA binding
2
1
0
1
2
-Log10 P value brute

B: WI+CS6h-C versus Ctl-C

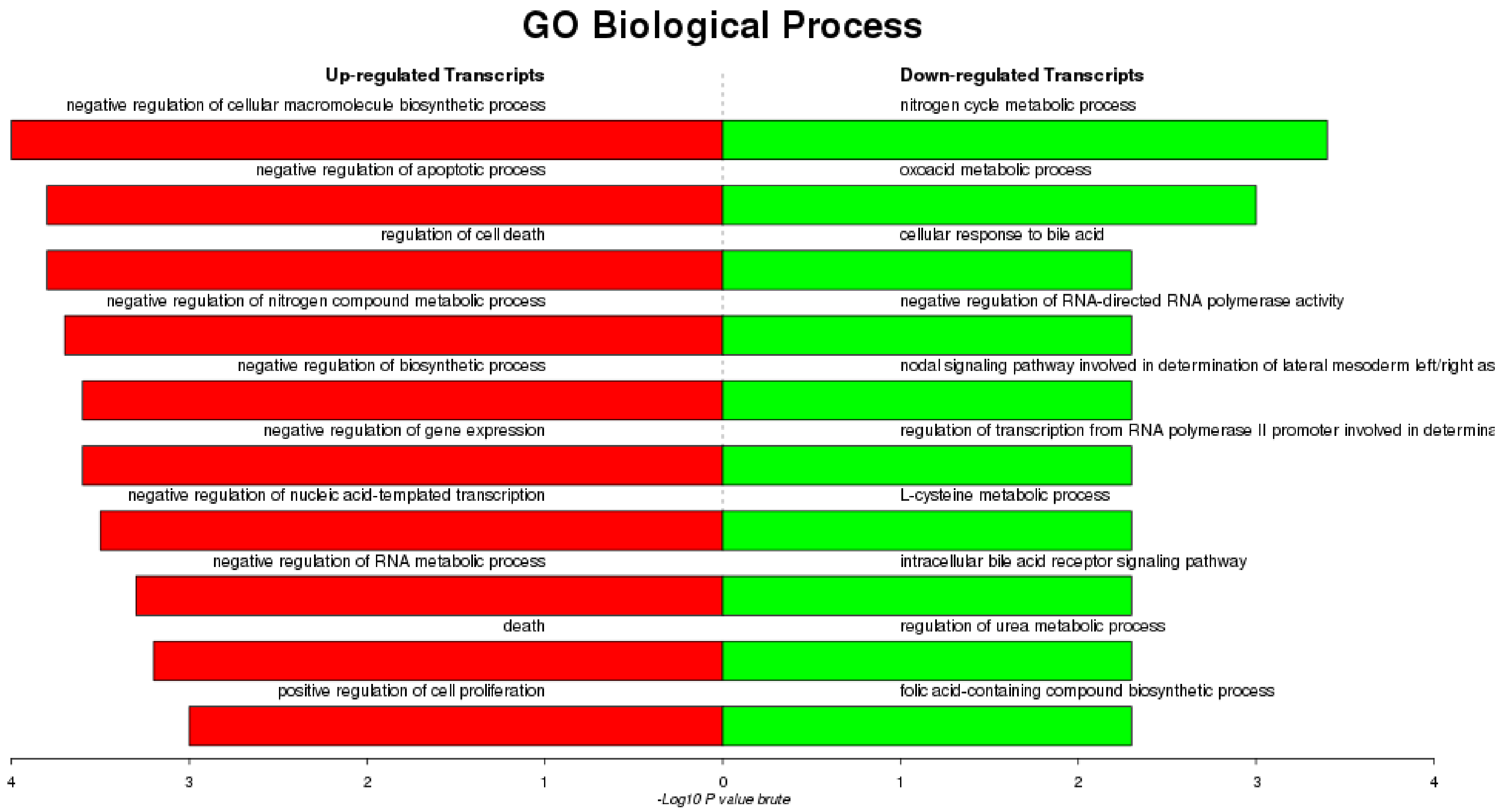

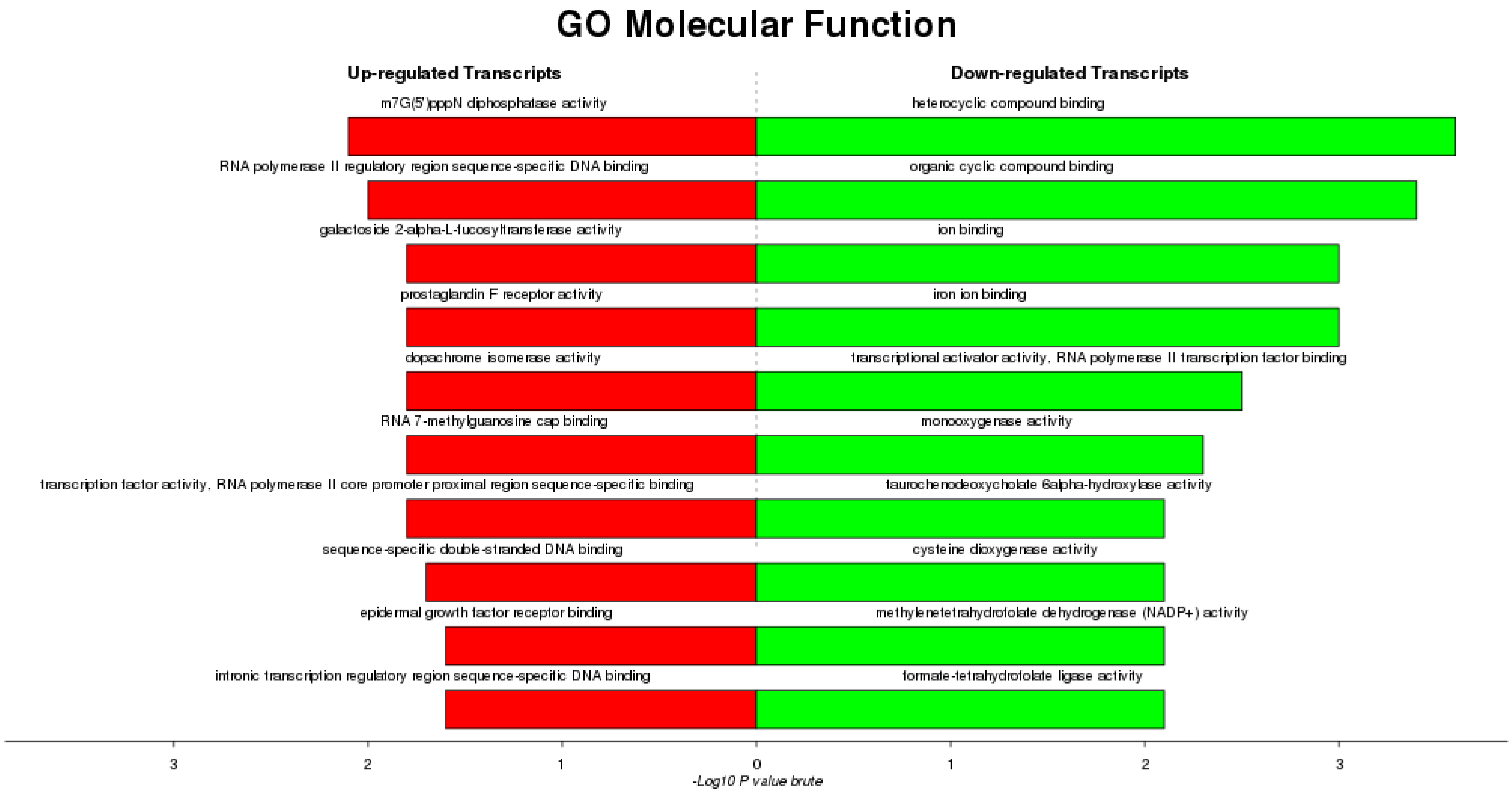
GO Molecular Function
Up-regulated Transcripts
Down-regulated Transcripts
m7G(5')pppN diphosphatase activity
RNA polymerase II regulatory region sequence-specific DNA binding
galactoside 2-alpha-L-fucosyltransferase activity
prostaglandin F receptor activity
dopachrome isomerase activity
RNA 7-methylguanosine cap binding
transcription factor activity, RNA polymerase II core promoter proximal region sequence-specific binding
sequence-specific double-stranded DNA binding
epidermal growth factor receptor binding
intronic transcription regulatory region sequence-specific DNA binding
heterocyclic compound binding
organic cyclic compound binding
ion binding
iron ion binding
transcriptional activator activity, RNA polymerase II transcription factor binding
monooxygenase activity
taurochenodeoxycholate 6alpha-hydroxylase activity
cysteine dioxygenase activity
methylenetetrahydrofolate dehydrogenase (NADP+) activity
formate-tetrahydrofolate ligase activity
3
2
1
0
1
2
3
-Log10 P value brute

C: WI+CS24h-C versus Ctl-C

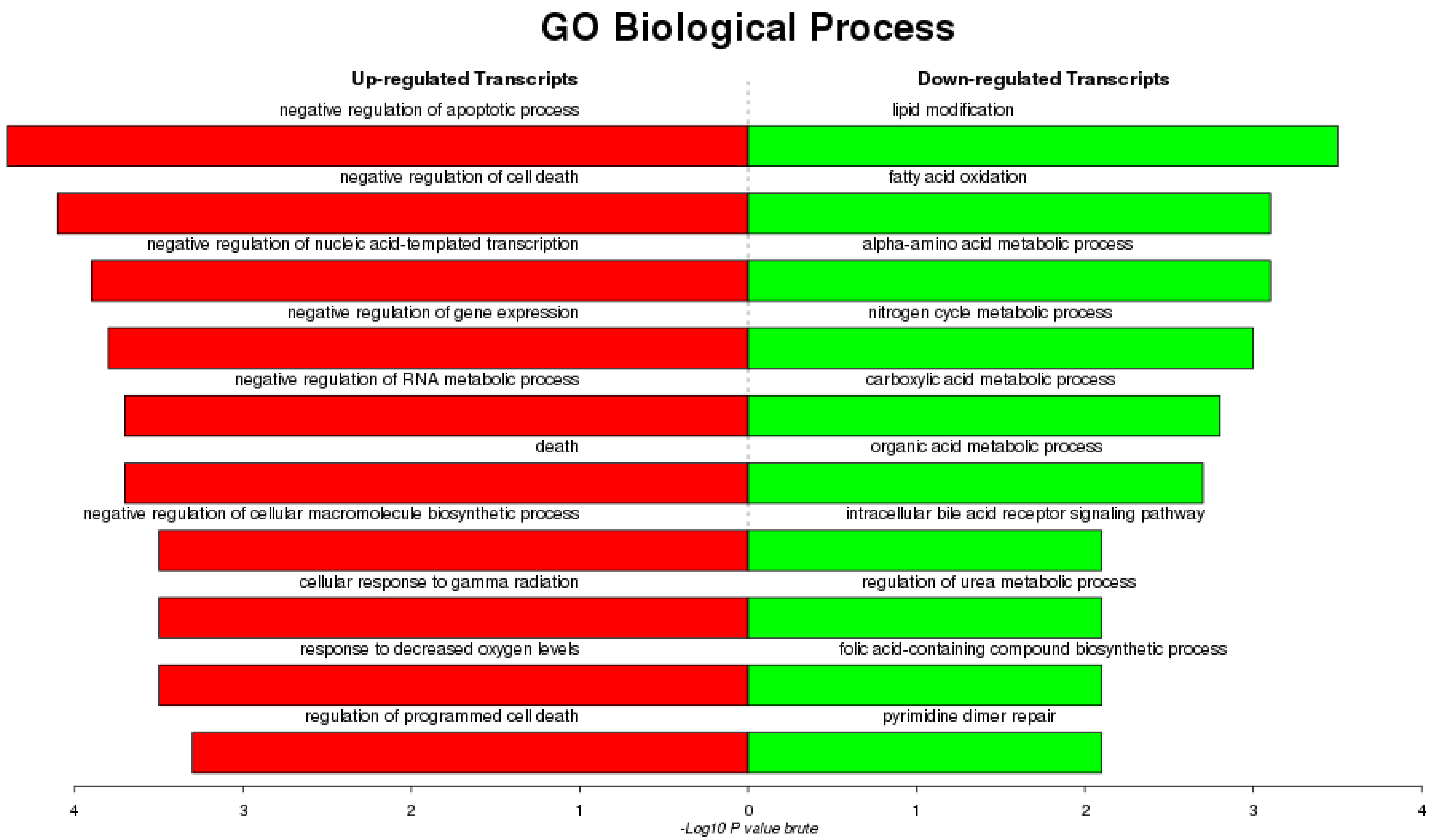

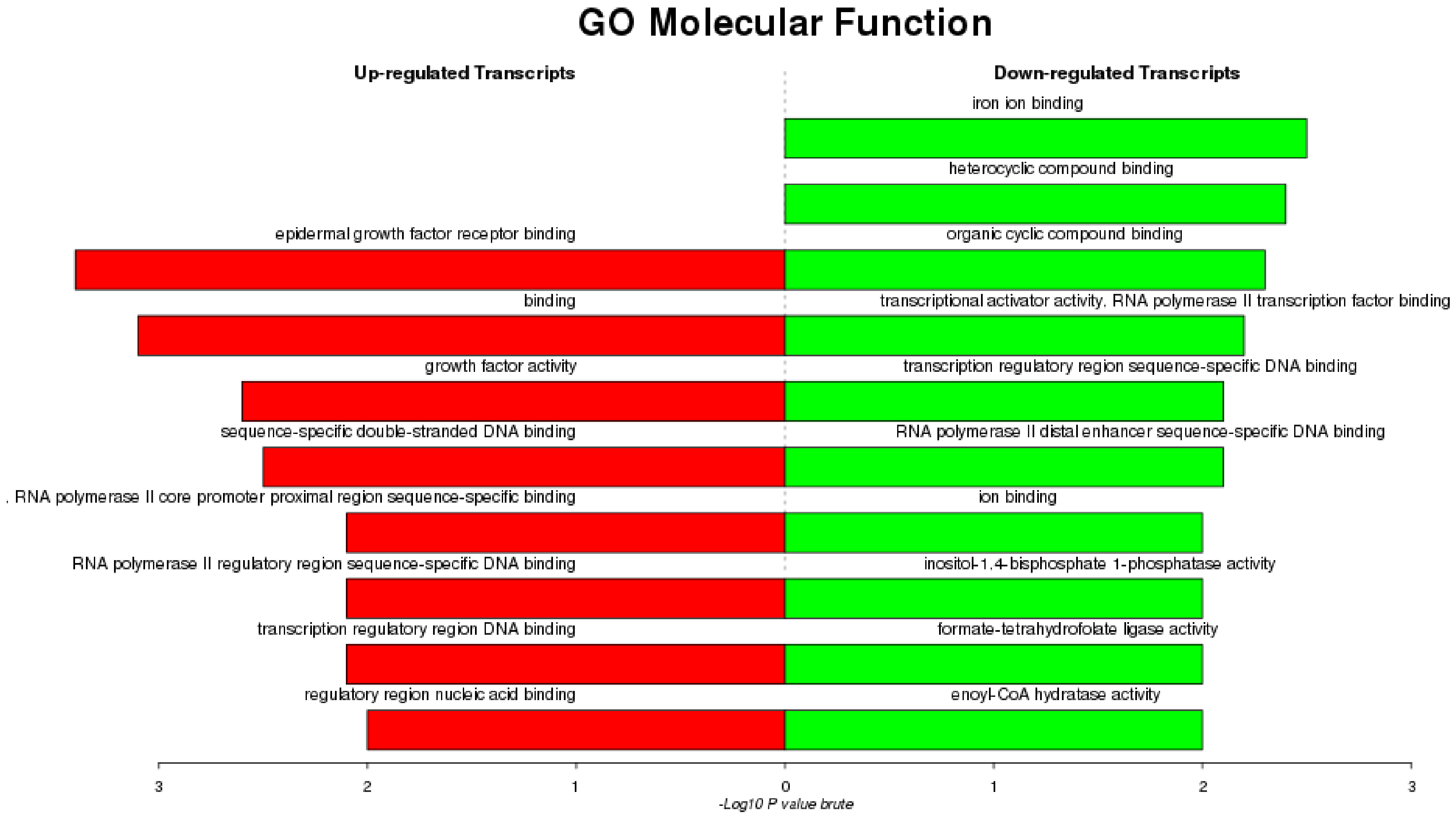
GO Molecular Function
Up-regulated Transcripts
Down-regulated Transcripts
iron ion binding
heterocyclic compound binding
epidermal growth factor receptor binding
organic cyclic compound binding
binding
transcriptional activator activity, RNA polymerase II transcription factor binding
growth factor activity
transcription regulatory region sequence-specific DNA binding
sequence-specific double-stranded DNA binding
RNA polymerase II distal enhancer sequence-specific DNA binding
. RNA polymerase II core promoter proximal region sequence-specific binding
ion binding
RNA polymerase II regulatory region sequence-specific DNA binding
inositol-1,4-bisphosphate 1-phosphatase activity
transcription regulatory region DNA binding
formate-tetrahydrofolate ligase activity
regulatory region nucleic acid binding
enoyl-CoA hydratase activity
3
2
1
0
1
2
3
-Log10 P value brute

D: WI+CS6h-C versus WI-C

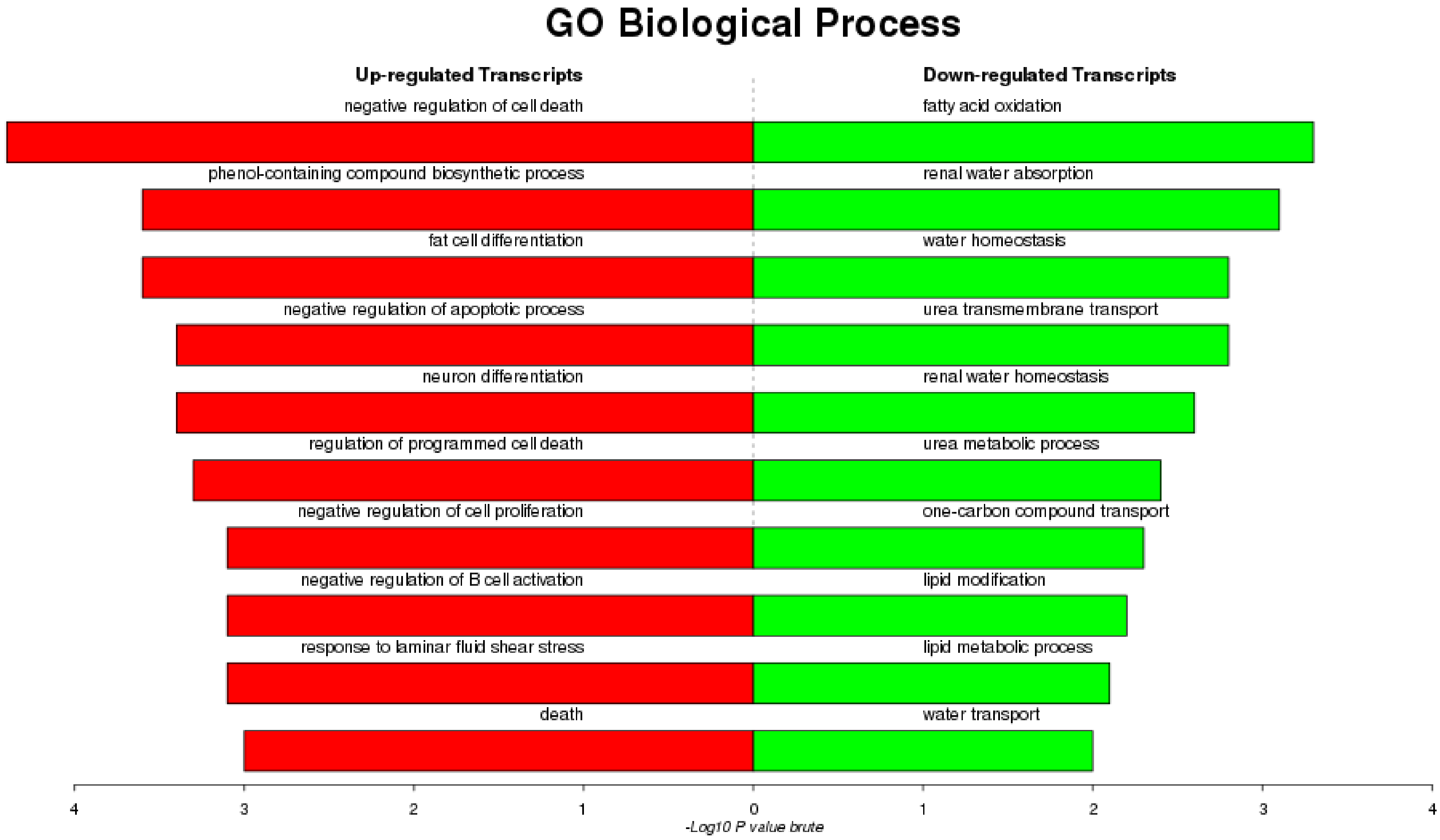

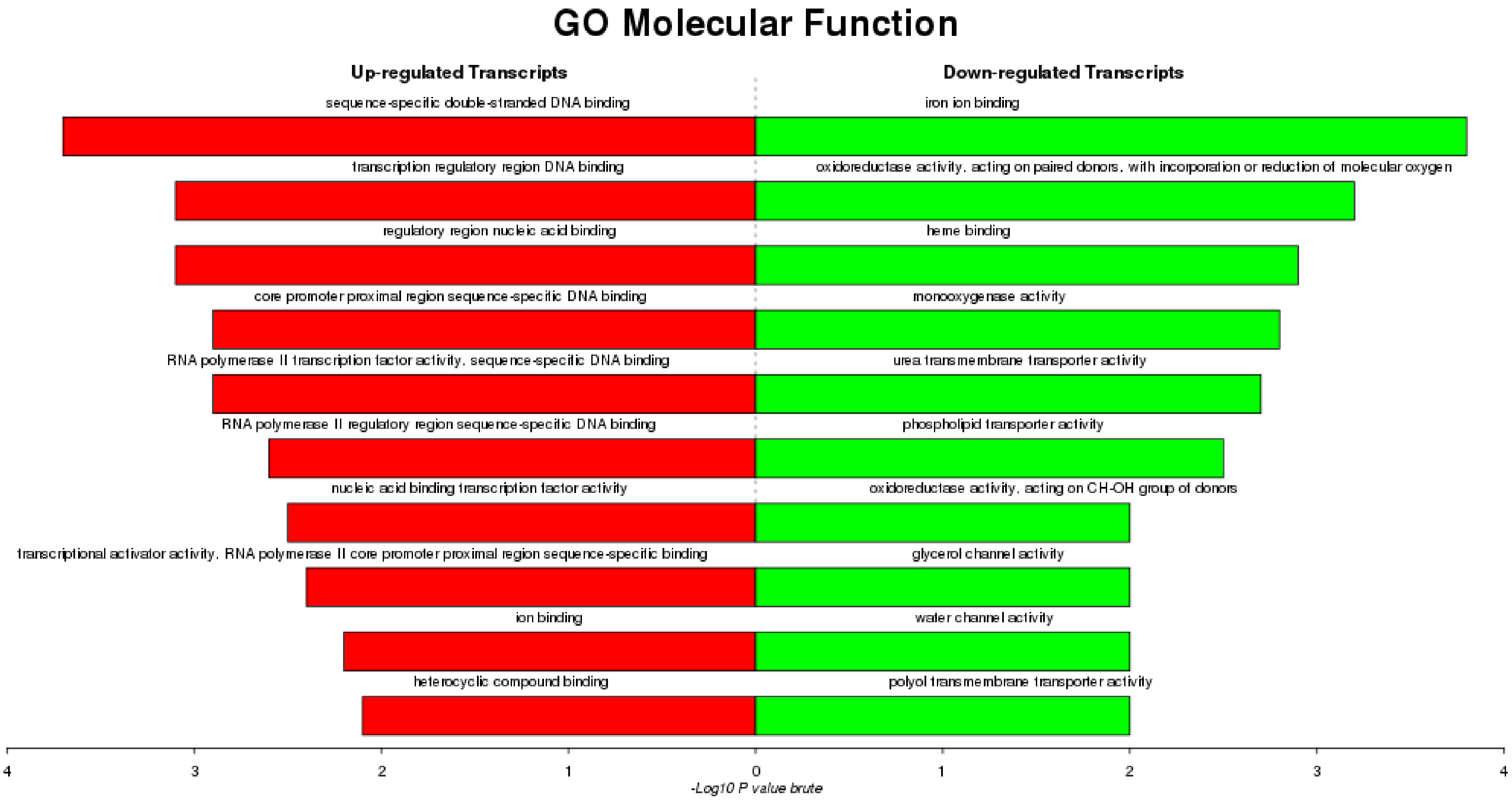
GO Molecular Function
Up-regulated Transcripts
Down-regulated Transcripts
sequence-specific double-stranded DNA binding
iron ion binding
transcription regulatory region DNA binding
oxidoreductase activity, acting on paired donors, with incorporation or reduction of molecular oxygen
regulatory region nucleic acid binding
heme binding
core promoter proximal region sequence-specific DNA binding
monooxygenase activity
RNA polymerase II transcription factor activity, sequence-specific DNA binding
urea transmembrane transporter activity
RNA polymerase II regulatory region sequence-specific DNA binding
phospholipid transporter activity
nucleic acid binding transcription factor activity
oxidoreductase activity, acting on CH-OH group of donors
transcriptional activator activity, RNA polymerase II core promoter proximal region sequence-specific binding
glycerol channel activity
ion binding
water channel activity
heterocyclic compound binding
polyol transmembrane transporter activity
4
3
2
1
0
1
2
3
4
-Log10 P value brute

E: WI+CS24h-C versus WI-C

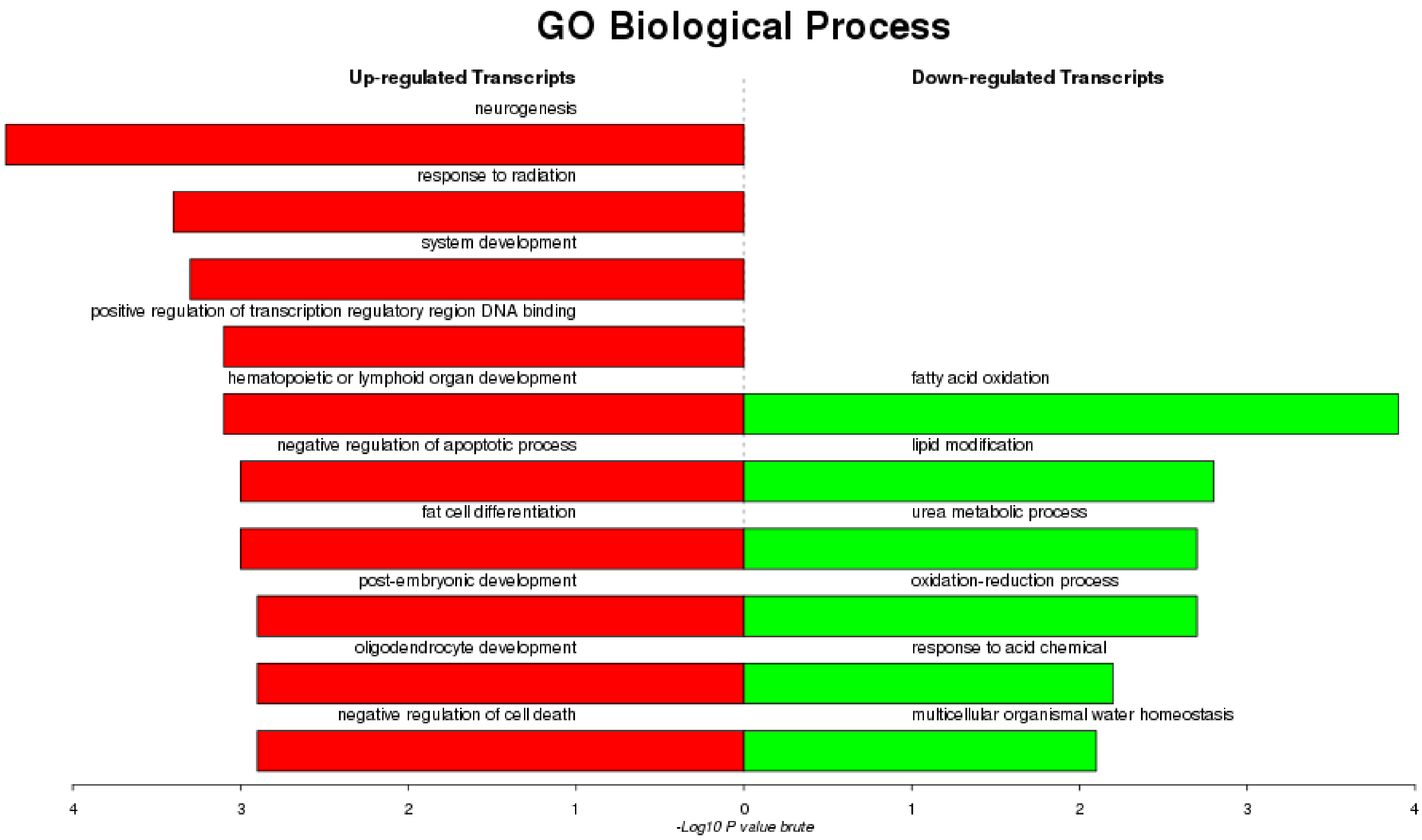

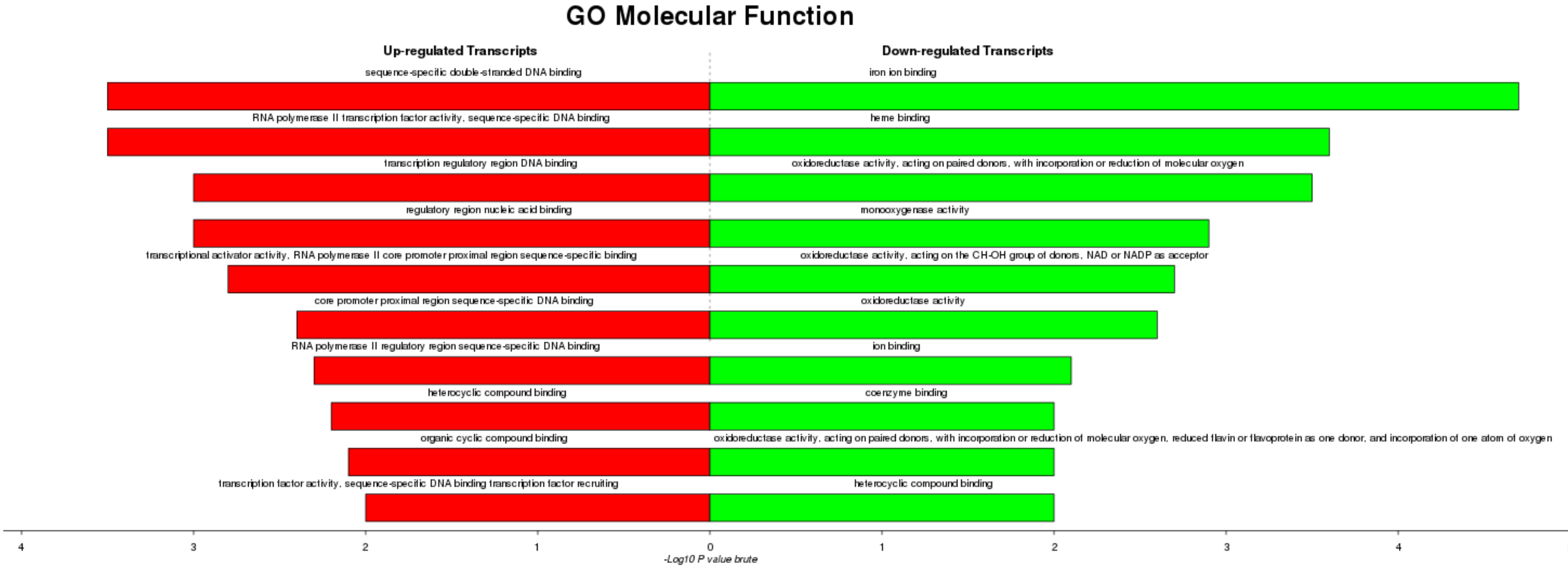
GO Molecular Function
Up-regulated Transcripts
Down-regulated Transcripts
sequence-specific double-stranded DNA binding
iron ion binding
RNA polymerase II transcription factor activity, sequence-specific DNA binding
heme binding
transcription regulatory region DNA binding
oxidoreductase activity, acting on paired donors, with incorporation or reduction of molecular oxygen
regulatory region nucleic acid binding
monooxygenase activity
transcriptional activator activity, RNA polymerase II core promoter proximal region sequence-specific binding
oxidoreductase activity, acting on the CH-OH group of donors, NAD or NADP as acceptor
core promoter proximal region sequence-specific DNA binding
oxidoreductase activity
RNA polymerase II regulatory region sequence-specific DNA binding
ion binding
heterocyclic compound binding
coenzyme binding
organic cyclic compound binding
oxidoreductase activity, acting on paired donors, with incorporation or reduction of molecular oxygen, reduced flavin or flavoprotein as one donor, and incorporation of one atom of oxygen
transcription factor activity, sequence-specific DNA binding transcription factor recruiting
heterocyclic compound binding
4
3
2
1
0
1
2
3
4
-Log10 P value brute

F: WI-CMJ versus Ctl-CMJ

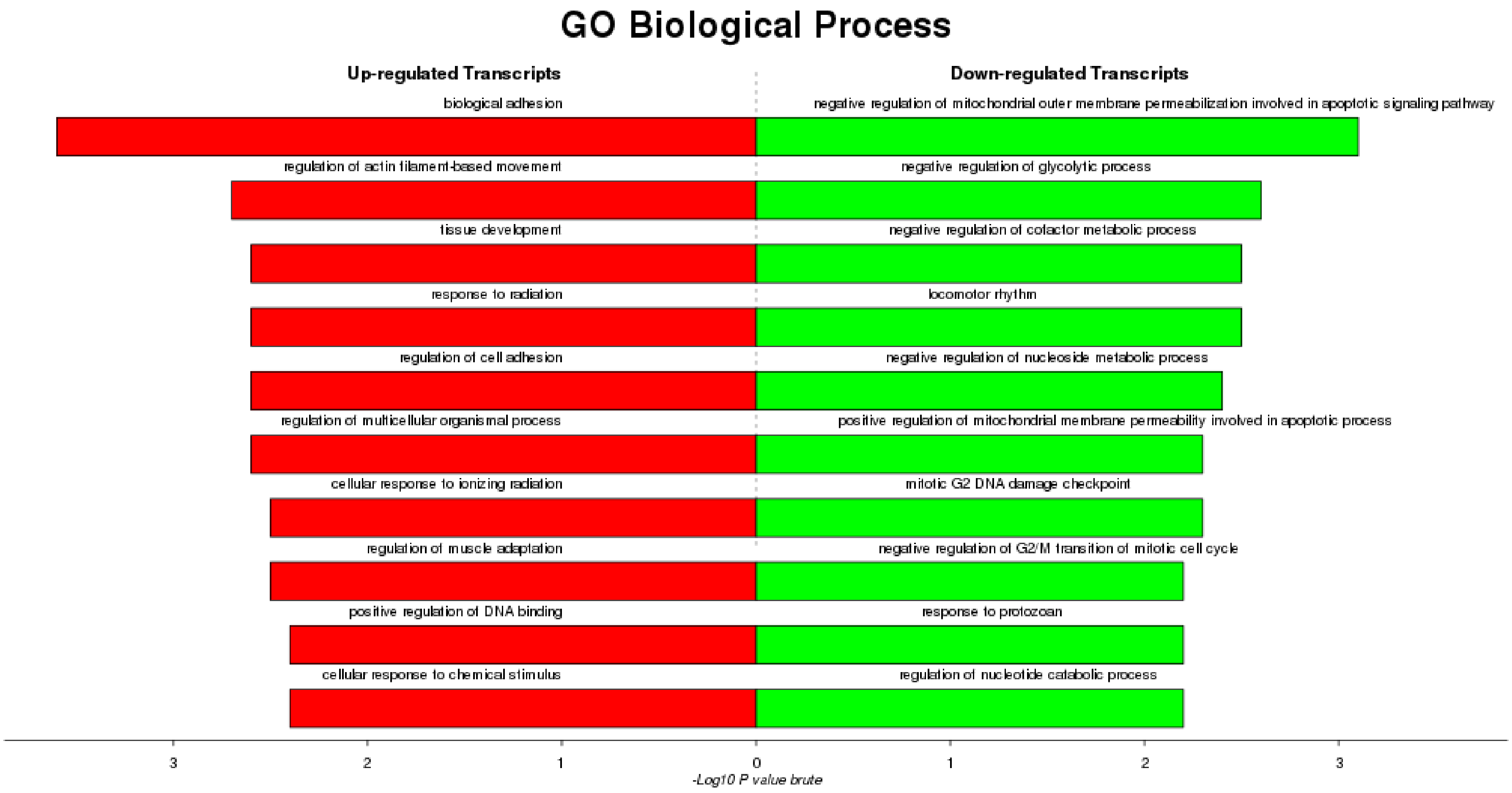

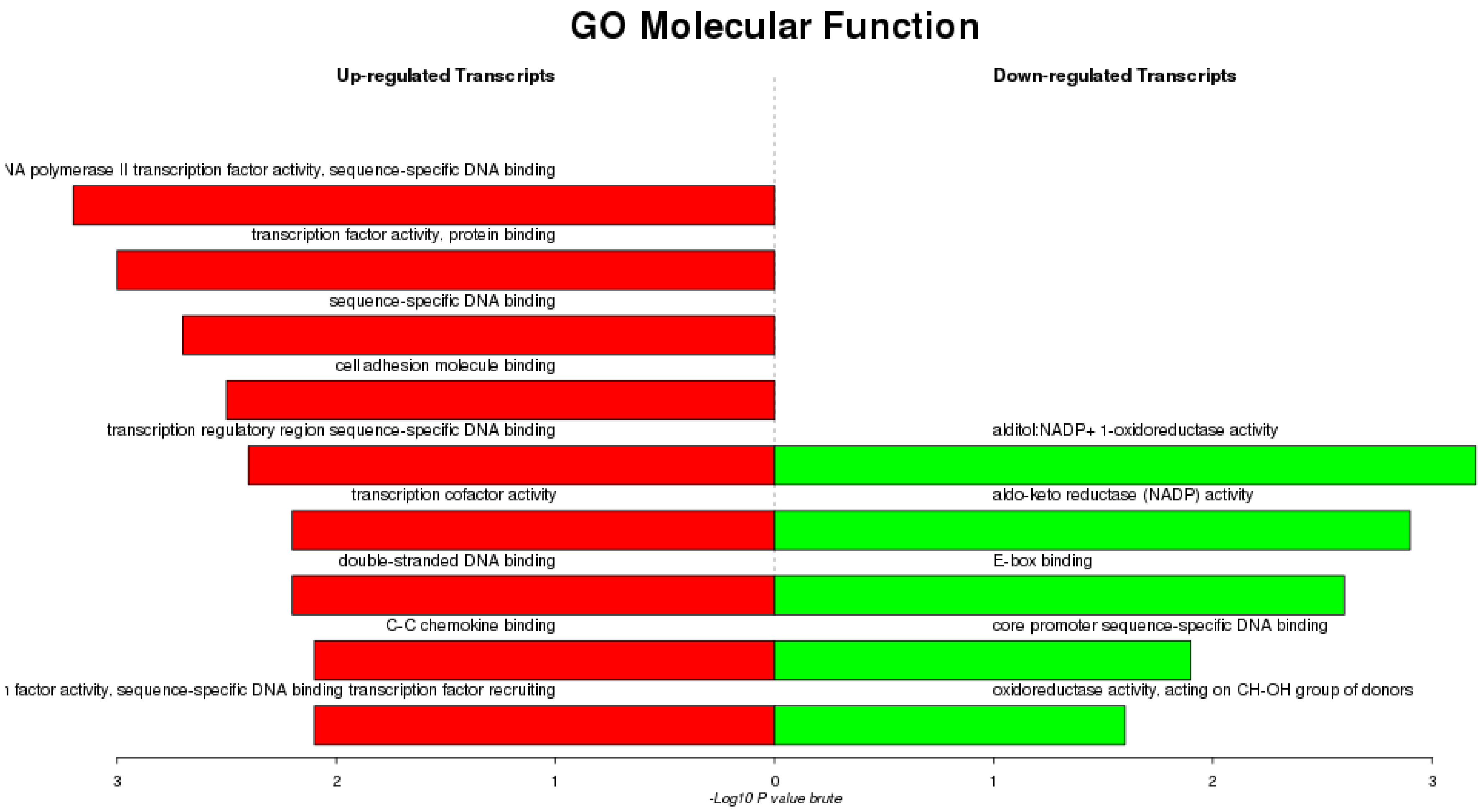
GO Molecular Function
Up-regulated Transcripts
Down-regulated Transcripts
NA polymerase II transcription factor activity, sequence-specific DNA binding
transcription factor activity, protein binding
sequence-specific DNA binding
cell adhesion molecule binding
transcription regulatory region sequence-specific DNA binding
transcription cofactor activity
double-stranded DNA binding
C-C chemokine binding
factor activity, sequence-specific DNA binding transcription factor recruiting
alditol:NADP+ 1-oxidoreductase activity
aldo-keto reductase (NADP) activity
E-box binding
core promoter sequence-specific DNA binding
oxidoreductase activity, acting on CH-OH group of donors
3
2
1
0
1
2
3
-Log10 P value brute

G: WI+CS6h-CMJ versus Ctl-CMJ

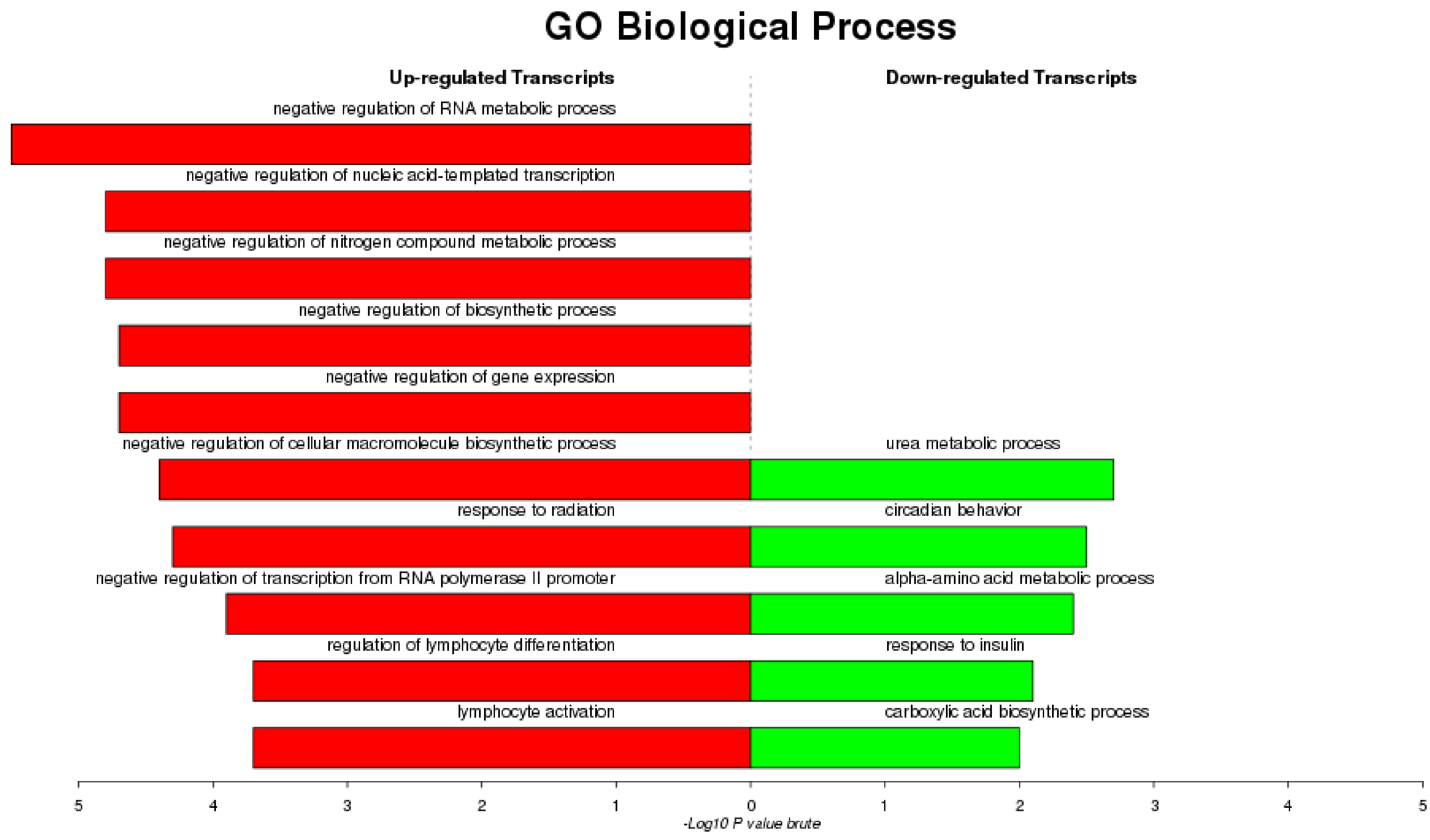

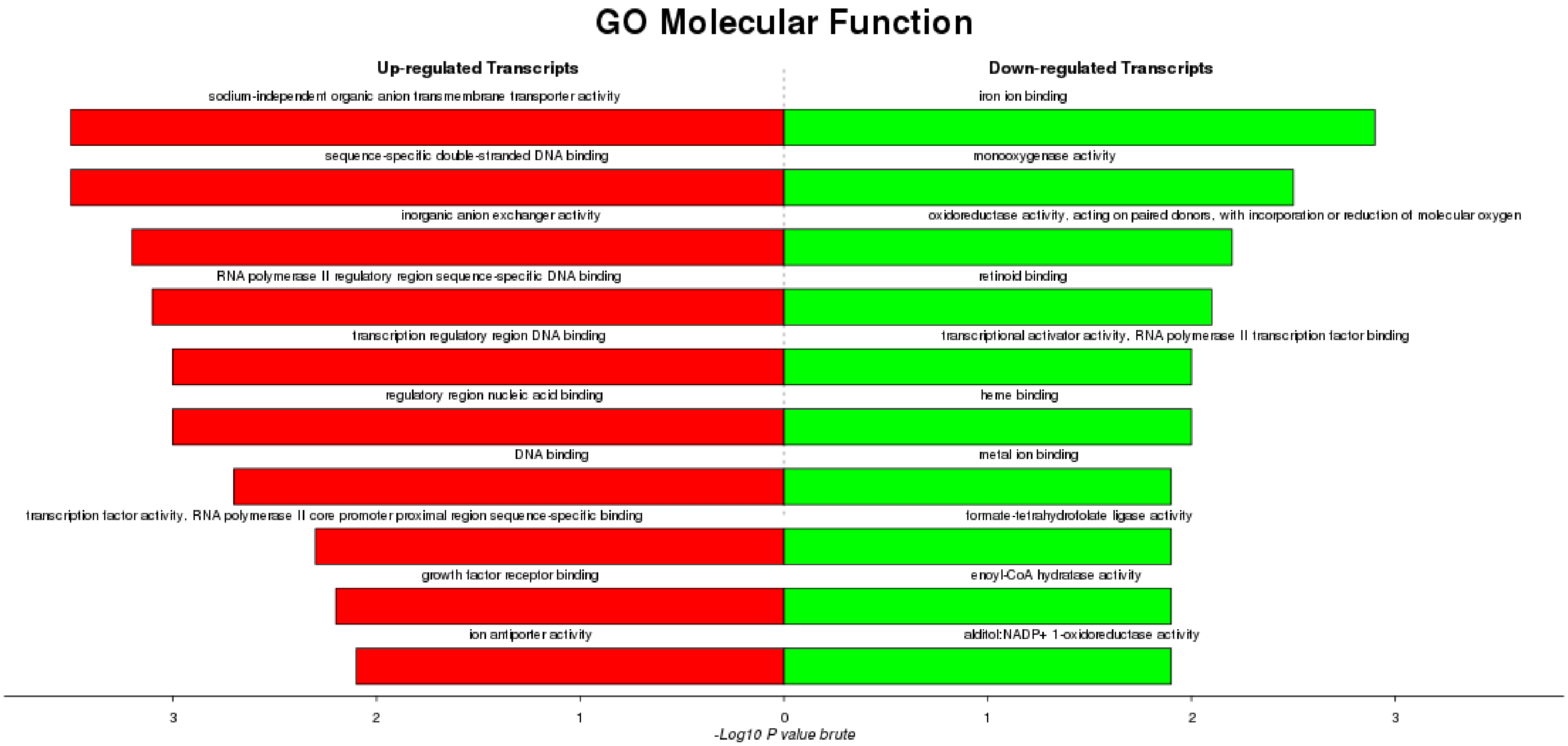
GO Molecular Function
Up-regulated Transcripts
Down-regulated Transcripts
sodium-independent organic anion transmembrane transporter activity
iron ion binding
sequence-specific double-stranded DNA binding
monooxygenase activity
inorganic anion exchanger activity
oxidoreductase activity, acting on paired donors, with incorporation or reduction of molecular oxygen
RNA polymerase II regulatory region sequence-specific DNA binding
retinoid binding
transcription regulatory region DNA binding
transcriptional activator activity, RNA polymerase II transcription factor binding
regulatory region nucleic acid binding
heme binding
DNA binding
metal ion binding
transcription factor activity, RNA polymerase II core promoter proximal region sequence-specific binding
formate-tetrahydrofolate ligase activity
growth factor receptor binding
enoyl-CoA hydratase activity
ion antiporter activity
alditol:NADP+ 1-oxidoreductase activity
3
2
1
0
1
2
3
-Log10 P value brute

H: WI+CS24h-CMJ versus Ctl-CMJ

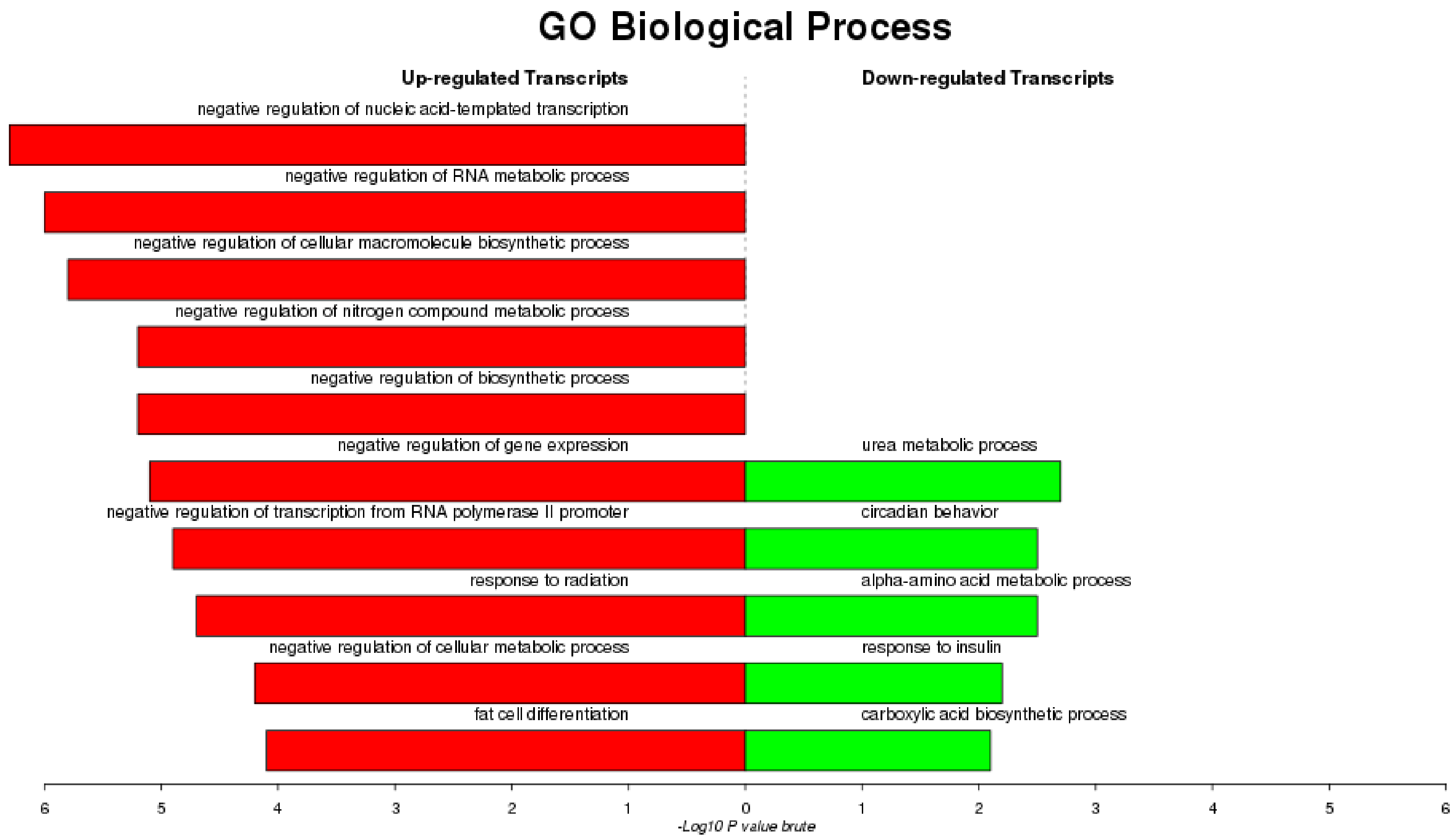

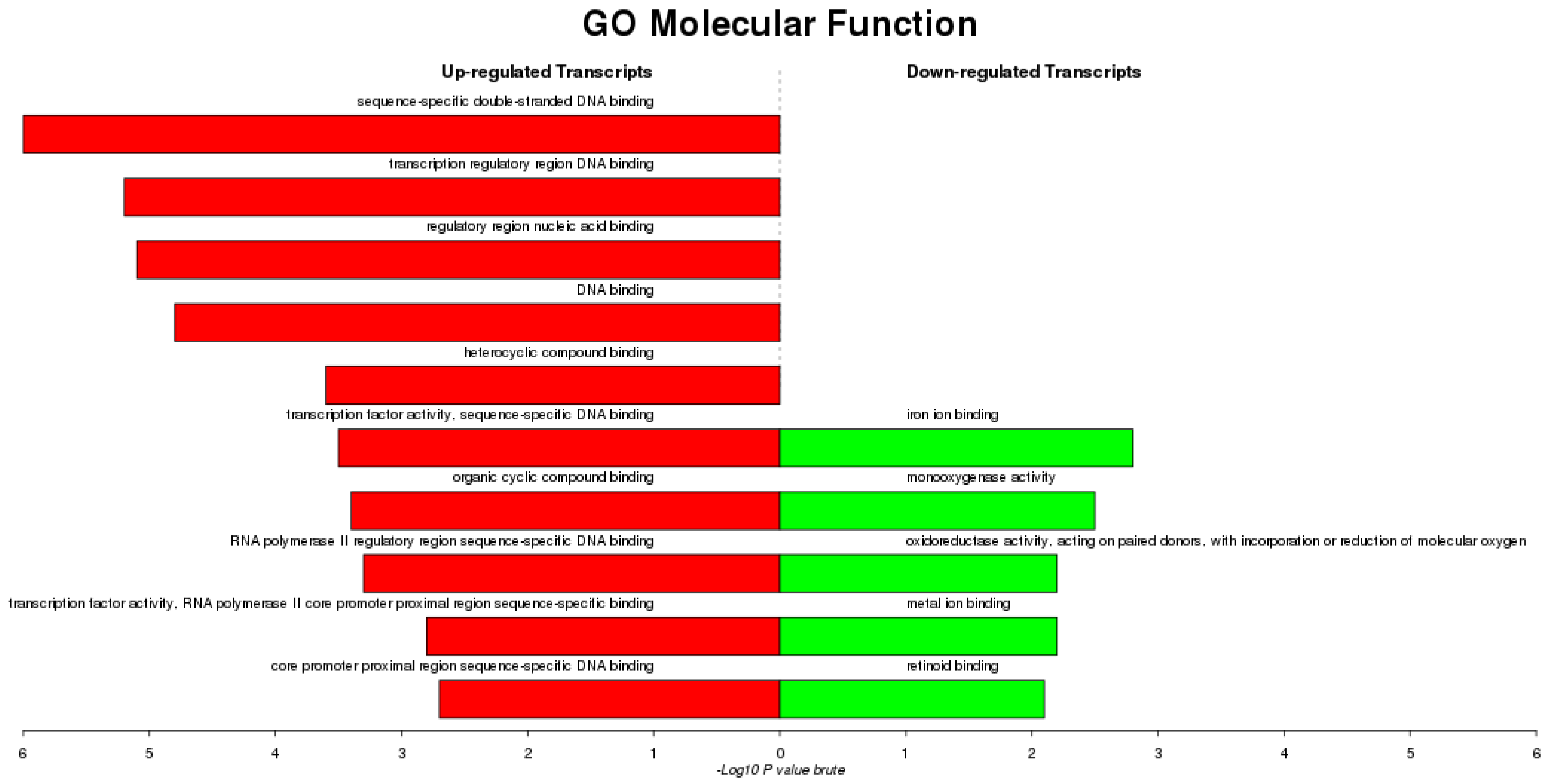
GO Molecular Function
Up-regulated Transcripts
Down-regulated Transcripts
sequence-specific double-stranded DNA binding
transcription regulatory region DNA binding
regulatory region nucleic acid binding
DNA binding
heterocyclic compound binding
transcription factor activity, sequence-specific DNA binding
iron ion binding
organic cyclic compound binding
monooxygenase activity
RNA polymerase II regulatory region sequence-specific DNA binding
oxidoreductase activity, acting on paired donors, with incorporation or reduction of molecular oxygen
transcription factor activity, RNA polymerase II core promoter proximal region sequence-specific binding
metal ion binding
core promoter proximal region sequence-specific DNA binding
retinoid binding
6 5 4 3 2 1 0 1 2 3 4 5 6
-Log10 P value brute

I: WI+CS6h-CMJ versus WI-CMJ

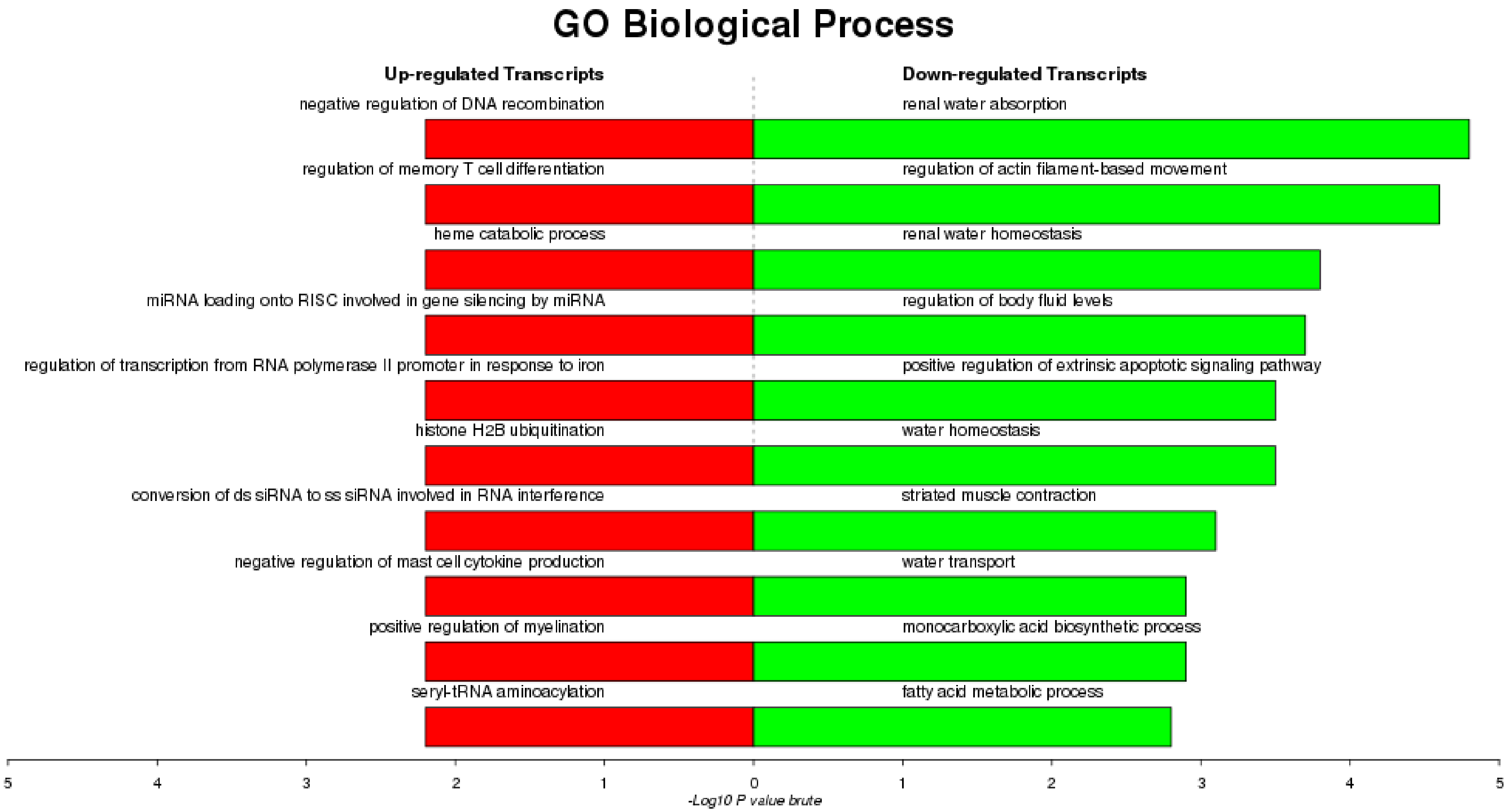

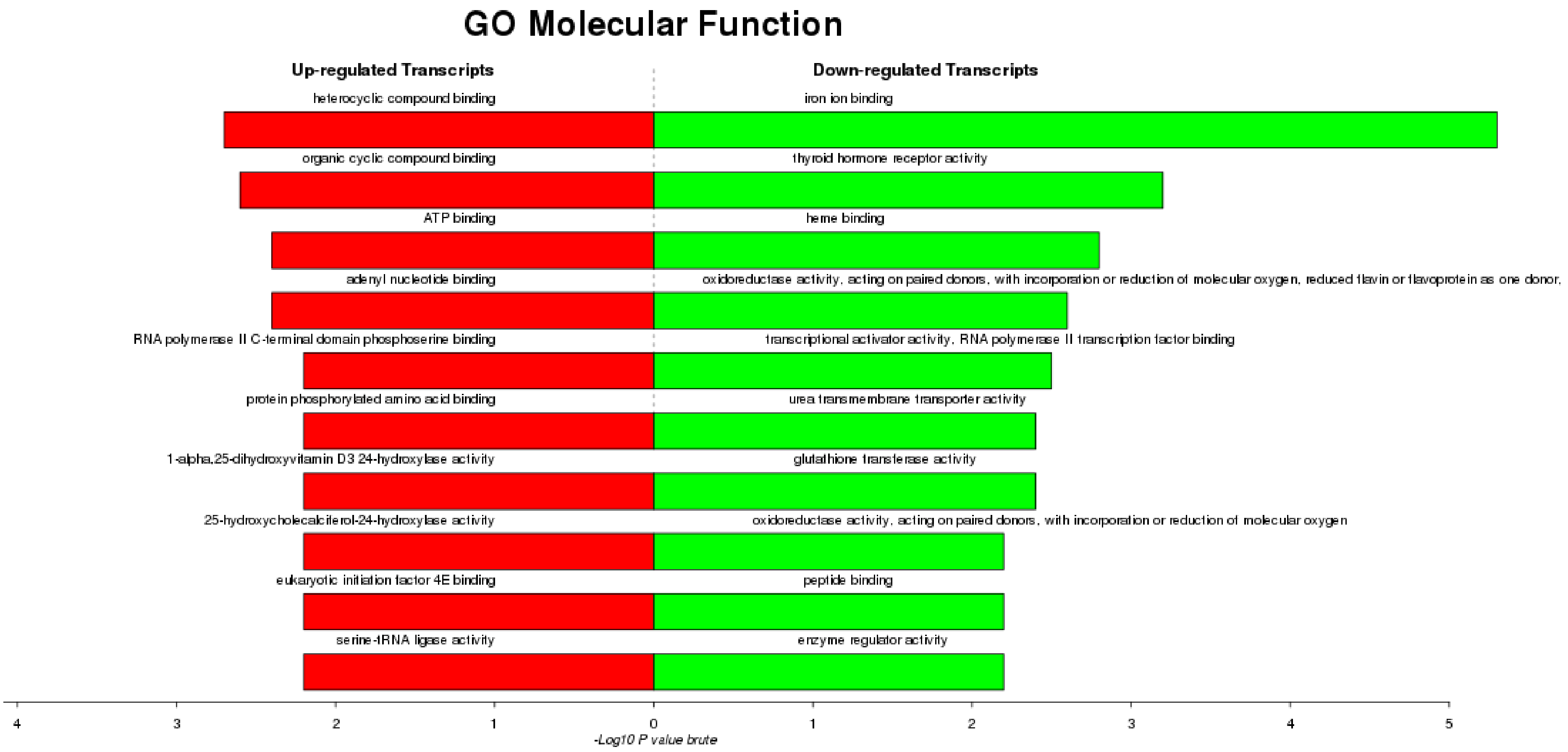
GO Molecular Function
Up-regulated Transcripts
Down-regulated Transcripts
heterocyclic compound binding
iron ion binding
organic cyclic compound binding
thyroid hormone receptor activity
ATP binding
heme binding
adenyl nucleotide binding
oxidoreductase activity, acting on paired donors, with incorporation or reduction of molecular oxygen, reduced flavin or flavoprotein as one donor,
RNA polymerase II C-terminal domain phosphoserine binding
transcriptional activator activity, RNA polymerase II transcription factor binding
protein phosphorylated amino acid binding
urea transmembrane transporter activity
1-alpha,25-dihydroxyvitamin D3 24-hydroxylase activity
glutathione transferase activity
25-hydroxycholecalciferol-24-hydroxylase activity
oxidoreductase activity, acting on paired donors, with incorporation or reduction of molecular oxygen
eukaryotic initiation factor 4E binding
peptide binding
serine-tRNA ligase activity
enzyme regulator activity
4
3
2
1
0
1
2
3
4
5
-Log10 P value brute

J: WI+CS24h-CMJ versus WI-CMJ

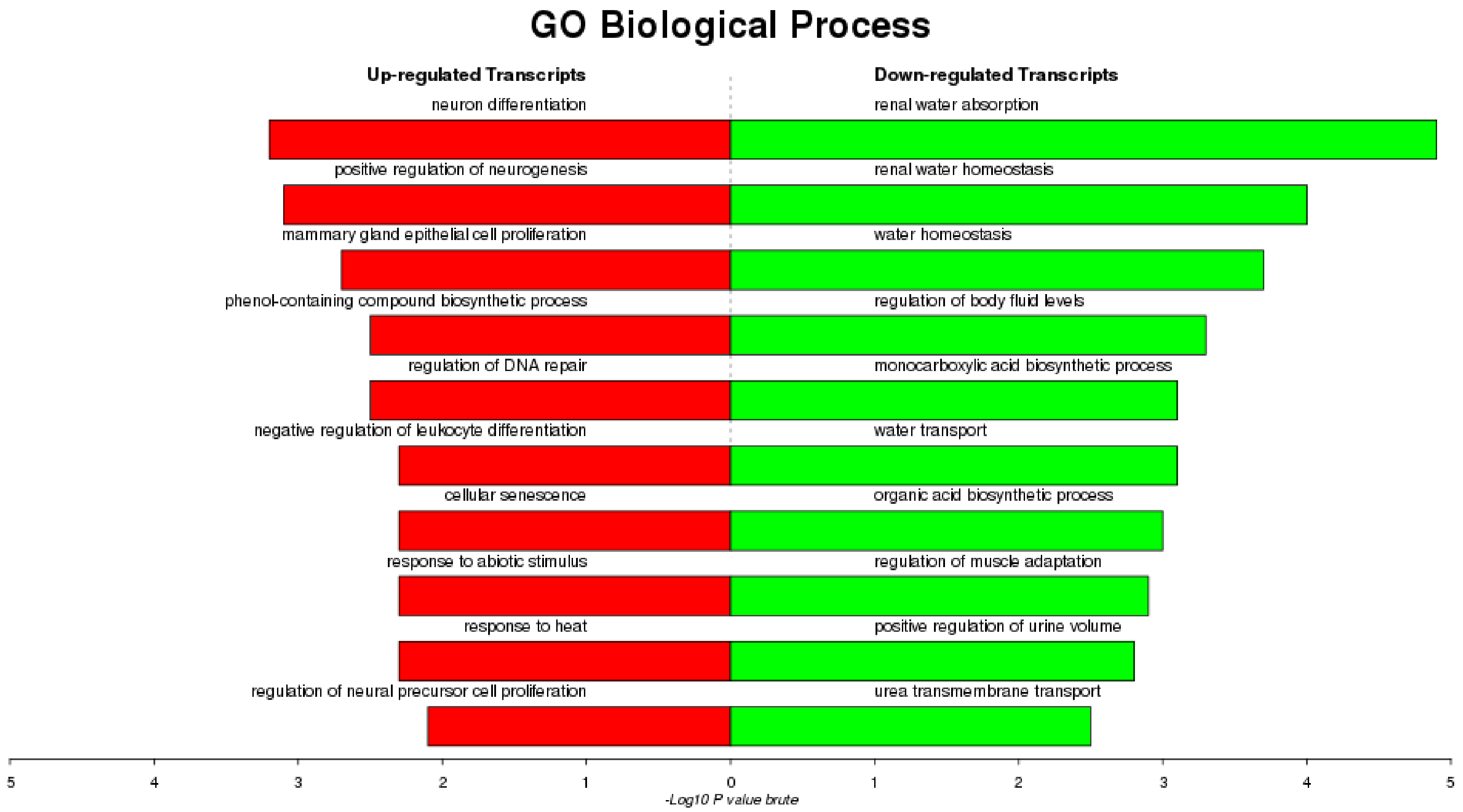

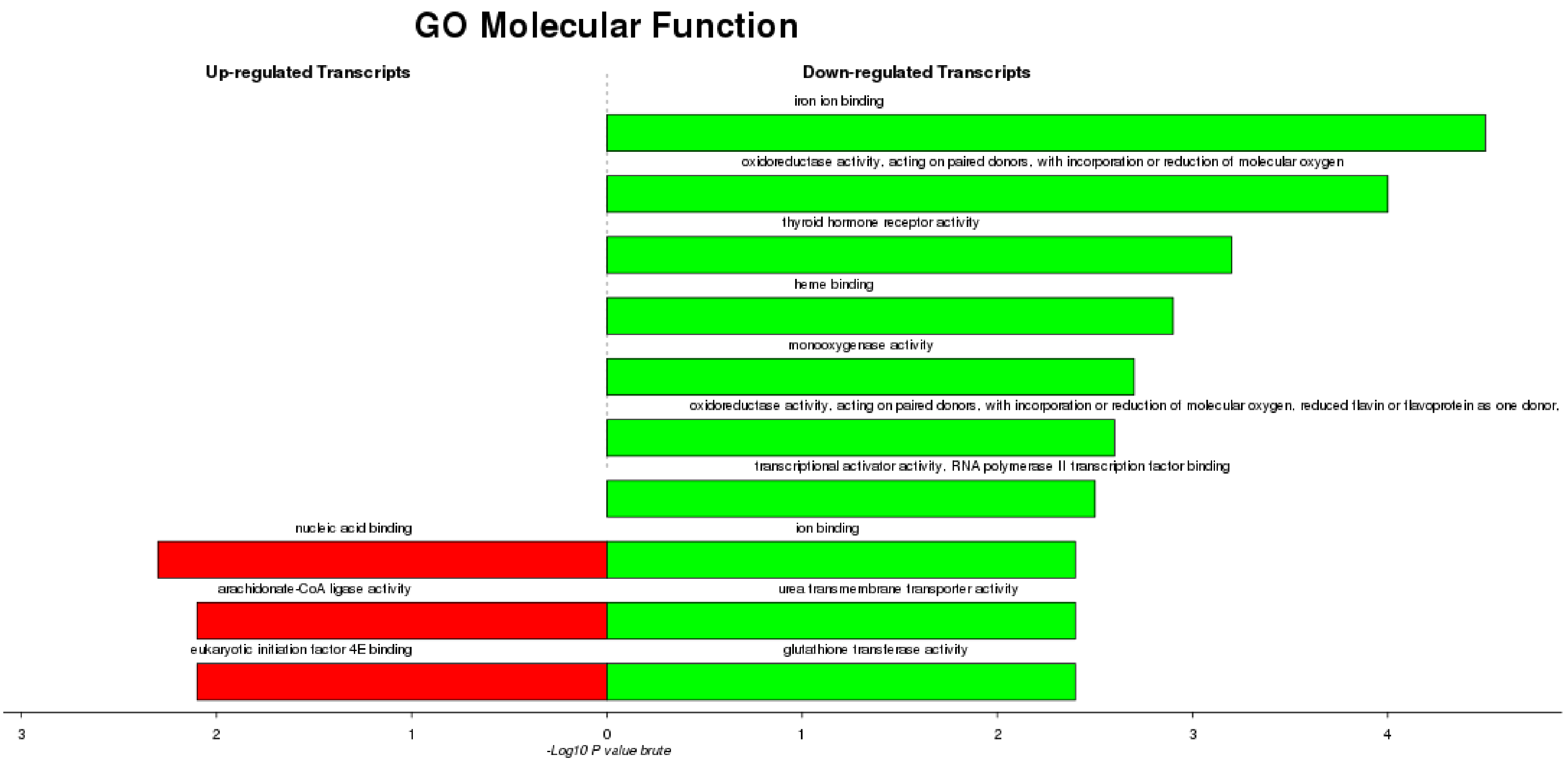
GO Molecular Function
Up-regulated Transcripts
Down-regulated Transcripts
iron ion binding
oxidoreductase activity, acting on paired donors, with incorporation or reduction of molecular oxygen
thyroid hormone receptor activity
heme binding
monooxygenase activity
oxidoreductase activity, acting on paired donors, with incorporation or reduction of molecular oxygen, reduced flavin or flavoprotein as one donor,
transcriptional activator activity, RNA polymerase II transcription factor binding
nucleic acid binding
ion binding
arachidonate-CoA ligase activity
urea transmembrane transporter activity
eukaryotic initiation factor 4E binding
glutathione transferase activity
3
2
1
0
1
2
3
4
-Log10 P value brute

Table S1: List of differentially expressed genes in cortex (C) tissues extracted from heatmaps.

The table provided represents the down-regulated and up-regulated differentially expressed genes in cortex compared conditions, with a corrected p value < 5% and a log2 fold change > 0.5. The annotation was obtained from Feature Extraction software.

Systematic Name	Gene Name or predictive Gene Name	Log 2 Fold change	p value (correction BH)
**WI-C versus Ctl-C** (Down-regulated genes)			
A_72_P231427	Cell Division Control Protein 42 Effector Protein 1 (CDC42EP1)	-3.232	1.02E-08
TC602959	-	-2.054	7.94E-11
FD592765	-	-1.759	2.74E-03
A_72_P001141	-	-1.716	7.94E-11
TC562631	-	-1.567	9.57E-08
TC538121	-	-1.367	6.75E-10
TC584676	-	-1.210	1.77E-03
TC535436	-	-1.142	2.00E-04
TC631544	-	-1.066	2.37E-03
TC625961	-	-1.021	7.00E-04
ENSSSCT00000034652	Immediate Early Response 3 (IER3)	-0.896	2.30E-04
ENSSSCT00000034425	Mitogen-Activated Protein Kinase Kinase Kinase 8 (MAP3K8)	-0.737	4.54E-03
TC574870	-	-0.732	9.60E-03
CO992076	Endometrium Gilt D6 Of Estrous Cycle	-0.724	8.28E-09
TC525370	-	-0.681	3.70E-04
DN104045	-	-0.668	1.51E-03
TC607613	-	-0.603	4.95E-02
TC531633	-	-0.598	2.96E-02
TC613549	-	-0.591	5.32E-06
AK350920	-	-0.578	3.24E-02
TC539177	-	-0.571	1.30E-03
TC600827	-	-0.522	1.40E-04
**WI-C versus Ctl-C** (Up- regulated genes)			
JN899104	Small Nucleolar RNA, C/D Box 3 Cluster (pSNORD3)	1.366	7.94E-03
NM_001123113	Fos Proto-Oncogene, AP-1 Transcription Factor Subunit	1.335	1.58E-03
ENSSSCT00000002650	Proto-Oncogene c-Fos (FOS)	1.166	7.68E-03
ENSSSCT00000001267	Histone H2A Type 1-Like (HIST1H2AB)	0.779	3.68E-02
NM_001123189	RAS Homolog Family Member B (RHOB)	0.721	1.78E-03
AK351795	CD83	0.707	4.95E-02

ENSSSCT00000032777	Guanine Nucleotide Exchange Factor 3 (VAV3)	0.669	1.15E-02
**WI+CS6h-C versus Ctl-C** (Down-regulated genes)			
A_72_P231427	Cell Division Control Protein 42 Effector Protein 1 (CDC42EP1)	-3.566	7.15E-10
NM_001167835	Cytochrome P450 2C42 (CYP2C42)	-3.264	6.10E-04
A_72_P443884	-	-2.839	1.03E-03
TC602959	-	-2.671	3.46E-14
FD592765	-	-2.612	4.81E-07
NM_213850	Glutathione S-Transferase Alpha 2 (GSTA2)	-2.590	3.90E-04
TC534148	-	-2.419	2.39E-08
NM_001159306	Pyruvate Dehydrogenase Kinase Isozyme 4, Mitochondrial (PDK4)	-2.419	5.53E-05
A_72_P054101	-	-2.344	2.08E-03
TC613147	-	-2.300	7.30E-04
TC548575	-	-1.936	6.68E-07
ENSSSCT00000015537	Cysteine Dioxygenase Type 1 (CDO1)	-1.910	1.37E-06
KP735782	Isolate S1-V1 Glutathione S-Transferase A2 (GSTA2)	-1.889	5.60E-04
TC541539	-	-1.780	1.89E-06
TC584676	-	-1.768	3.32E-07
AK232564	-	-1.756	5.09E-06
TC622808	-	-1.722	4.80E-04
A_72_P001141	-	-1.701	3.75E-11
TC623278	Sex comb on midleg-like 1 (SCML1)	-1.648	2.80E-04
TC541610	-	-1.559	5.71E-05
DN134227	-	-1.542	1.85E-05
TC631544	-	-1.469	1.39E-06
NM_001078665	Chemokine Ligand 26-Like (CCL26) (MIP-4a, Eotaxin-3)	-1.449	1.23E-06
CN163990	-	-1.444	3.19E-07
TC566965	-	-1.434	9.20E-04
TC592596	-	-1.426	2.47E-05
CK452842	-	-1.411	4.00E-04
AK345473	-	-1.401	7.38E-05
TC562631	-	-1.387	3.63E-07
TC598771	-	-1.350	3.69E-06
TC528367	-	-1.321	2.88E-03
TC548865	-	-1.307	7.48E-08
TC538121	-	-1.279	1.39E-09
A_72_P032671	-	-1.275	5.90E-04
AK236120	CALHM2	-1.275	1.12E-03
TC530663	-	-1.270	6.90E-04
TC593206	-	-1.266	1.10E-04
XM_003130522	HHIP-Like Protein 2-Like	-1.266	7.80E-04
ENSSSCT00000035428	Arginase 2 (ARG2)	-1.262	2.42E-06
TC623045	-	-1.261	1.71E-05
TC557164	2'-5' Oligoadenylate Synthetase 2 (OAS)	-1.259	1.42E-03
AK237295	GPCPD1	-1.257	1.10E-04

TC590865	-	-1.256	1.88E-03
DN102262	-	-1.255	8.70E-06
TC590364	-	-1.254	2.31E-05
A_72_P152666	-	-1.250	2.00E-04
TC537819	-	-1.231	1.83E-03
TC531633	-	-1.229	3.22E-08
TC583840	-	-1.219	4.10E-04
AJ944339	-	-1.218	1.61E-03
TC571068	-	-1.215	9.83E-07
AK232386	MX2	-1.214	7.03E-05
TC559094	-	-1.208	5.61E-07
AK345001	MX2	-1.200	5.70E-04
TC526612	-	-1.199	1.40E-04
TC597211	-	-1.191	1.80E-03
TC604056	-	-1.189	3.90E-04
AK236725	OVRM10220H04	-1.180	1.55E-03
TC542297	-	-1.172	2.67E-03
NM_001195115	SLC25A27	-1.170	5.09E-06
NM_001128474	Guanylate binding protein 2, interferon-inducible (GBP2)	-1.165	2.86E-07
TC536661	-	-1.158	1.08E-06
TC547562	Zinc finger protein 473 (ZNF473)	-1.140	5.94E-06
XM_013981732	GNAT1	-1.136	1.31E-06
AK236259	TRMT6	-1.130	1.10E-04
TC548235	-	-1.124	2.62E-03
BF713827	-	-1.112	1.32E-03
TC610430	-	-1.106	9.70E-04
TC576734	Inter-Alpha-Trypsin Inhibitor Heavy Chain H1 Precursor	-1.098	7.70E-04
TC561223	-	-1.089	7.90E-04
NM_001243452	DNA-damage-inducible transcript 4 (DDIT4)	-1.088	5.57E-05
NM_001097416	MX2	-1.087	1.20E-04
TC526535	-	-1.086	1.40E-04
TC545802	-	-1.081	1.10E-04
FD604545	-	-1.081	7.40E-04
NM_001129953	DMP1	-1.073	7.90E-04
TC532659	-	-1.072	5.60E-04
TC602146	-	-1.071	6.90E-04
AK232543	-	-1.051	1.50E-04
AK400481	EIF4A2	-1.049	2.28E-03
EW262869	-	-1.046	5.02E-05
TC610412	-	-1.046	1.50E-04
TC569766	-	-1.045	2.15E-03
TC569278	-	-1.028	1.07E-03
TC604030	-	-1.027	1.41E-03
TC560156	-	-1.024	3.57E-08
TC543158	Histamine N-Methyltransferase	-1.019	4.35E-05

TC525588	-	-1.018	6.40E-04
TC575827	-	-1.014	8.96E-06
TC532441	-	-1.011	1.08E-03
TC528319	-	-1.011	3.01E-03
CV871207	-	-1.006	1.60E-04
TC550796	-	-1.006	1.11E-03
AK350920	-	-1.005	9.85E-07
ENSSSCT00000002106	MAN2C1	-1.003	1.25E-03
A_72_P049531	-	-1.000	4.90E-04
TC598894	-	-0.990	5.60E-04
NM_001287412	Nuclear Receptor Subfamily 1H4 (NR1H4)	-0.988	4.10E-04
AK349880	-	-0.985	8.10E-04
NM_213742	Calcitonin-related polypeptide beta (CALCB)	-0.983	3.63E-05
AK350303	SPL010030C03	-0.979	2.68E-03
TC602526	-	-0.977	4.88E-05
AK393873	-	-0.970	1.90E-04
TC566289	-	-0.966	1.05E-03
TC555339	-	-0.961	1.89E-05
AK237715	SH3BP2	-0.960	4.33E-13
BP446680	-	-0.960	3.19E-07
TC600327	-	-0.960	1.51E-03
TC597094	-	-0.958	2.08E-03
A_72_P431054	-	-0.956	2.05E-06
TC537402	-	-0.951	9.20E-04
TC574870	-	-0.950	1.91E-05
ENSSSCT00000010573	ADAM-like, decysin 1 (ADAMDEC1)	-0.944	2.60E-04
AK399777	SRSF5	-0.943	1.64E-06
TC627807	LOC106504276	-0.936	1.20E-04
CN157777	-	-0.932	3.10E-04
ENSSSCT00000007615	WDR63	-0.929	2.09E-03
AJ955670	-	-0.928	6.00E-04
TC609498	-	-0.926	1.07E-03
EW034563	-	-0.913	2.80E-04
AK347470	LOC100624597	-0.912	1.80E-04
TC529862	-	-0.910	2.18E-05
TC527153	-	-0.907	8.60E-04
TC610393	-	-0.905	2.90E-04
TC529859	-	-0.904	2.60E-04
DN132923	-	-0.903	3.00E-04
CK454137	-	-0.903	2.24E-03
AK352378	VPS51	-0.896	2.60E-04
ENSSSCT00000034425	Mitogen-Activated Protein Kinase Kinase Kinase 8 (Map3k8)	-0.893	2.35E-05
AK343323	-	-0.892	1.71E-03
TC614889	-	-0.891	4.88E-05
XM_013978816	Annexin A3 (ANXA3)	-0.890	6.85E-06

CJ030181	-	-0.890	1.29E-03
NM_001134349	Kruppel-like factor 15 (KLF15)	-0.889	5.53E-06
NM_001101027	CYP39A1	-0.888	5.94E-06
AK346234	LOC100520861	-0.886	6.50E-04
ENSSSCT00000023435	TNF Receptor Associated Factor 3 (TRAF3)	-0.885	2.33E-05
CF177312	-	-0.885	2.37E-03
XR_001309264	LOC102158887	-0.874	2.60E-04
TC538132	-	-0.874	6.10E-04
TC588483	-	-0.873	1.59E-03
NM_001143719	MAP kinase interacting serine/threonine kinase 1 (MKNK1)	-0.870	1.80E-05
ENSSSCT00000002574	Serine/Arginine-Rich Splicing Factor	-0.869	1.49E-07
TC529458	-	-0.865	1.59E-03
TC527407	-	-0.859	3.70E-05
TC621729	-	-0.859	1.01E-03
TC535434	-	-0.857	1.12E-03
TC568621	-	-0.856	4.10E-04
ENSSSCT00000004600	Mitogen-Activated Protein Kinase Kinase Kinase 5 (MEKK5)(MAP3K5)	-0.854	9.60E-04
TC521859	-	-0.850	1.11E-03
A_72_P214667	-	-0.849	2.98E-03
TC601797	Mitochondrial Carnitine O-Palmitoyltransferase 2.	-0.847	8.70E-04
AK351081	-	-0.845	8.90E-04
CN157151	-	-0.844	1.00E-03
TC580089	-	-0.842	1.25E-03
TC527857	-	-0.839	1.27E-03
ENSSSCT00000004670	Tumor protein D52-like 1 (TPD52L1)	-0.835	5.56E-05
TC587447	-	-0.834	5.30E-04
TC548784	-	-0.829	1.20E-03
TC591816	-	-0.826	9.91E-05
TC621173	-	-0.826	2.37E-03
TC557067	-	-0.821	1.22E-03
AK232808	-	-0.820	9.27E-06
TC625961	-	-0.820	9.10E-04
NM_001113439	SFRS18	-0.820	1.57E-03
DY405756	-	-0.819	2.60E-04
TC528988	-	-0.817	2.33E-03
NM_001097506	AN1-Type Zinc Finger Protein 5 (ZFAND5)	-0.813	4.41E-06
ENSSSCT00000015179	Arrestin domain containing 2 (ARRDC2)	-0.813	2.50E-04
AK231832	-	-0.812	3.80E-04
DY437600	-	-0.812	1.79E-03
TC586815	-	-0.807	1.64E-03
EW200629	-	-0.806	1.32E-05
TC606417	SJCHGC06720	-0.803	2.00E-04
TC526891	-	-0.803	1.07E-03
TC568032	-	-0.801	1.20E-04
AK394979	LOC100156195	-0.799	1.60E-04

ENSSSCT00000004334	Muty DNA Glycosylase	-0.796	4.10E-04
AW416888	-	-0.796	1.02E-03
TC531472	-	-0.794	7.80E-04
TC519529	-	-0.792	8.46E-06
ENSSSCT00000034119	LOC100516891	-0.791	3.26E-08
EW261758	-	-0.791	3.57E-08
ENSSSCT00000019569	Period circadian clock 1 (PER1)	-0.790	2.39E-03
AK396196	-	-0.788	1.52E-05
TC555462	-	-0.787	4.10E-07
TC523245	-	-0.785	3.00E-04
NM_214425	CYP4A21	-0.784	9.27E-06
TC535581	-	-0.781	1.71E-03
XM_003129835	Exophilin 5 (EXPH5)	-0.779	2.33E-05
TC567905	-	-0.777	2.94E-03
TC542747	-	-0.775	5.64E-05
BX675220	-	-0.771	1.81E-05
AK349343	-	-0.770	1.10E-04
AK394207	LVRM10091F11	-0.768	5.34E-05
AJ964208	-	-0.765	4.20E-04
TC546244	-	-0.760	1.35E-03
TC543254	-	-0.758	8.50E-04
TC605363	-	-0.757	2.24E-03
TC613011	-	-0.756	1.70E-04
ENSSSCT00000028241	SMAD specific E3 ubiquitin protein ligase 1	-0.756	3.70E-04
TC599345	-	-0.753	3.80E-04
TC536098	-	-0.749	1.37E-03
TC533014	-	-0.749	1.71E-03
TC599915	-	-0.748	3.86E-06
TC551004	-	-0.748	5.90E-04
BX677249	-	-0.747	1.08E-03
TC538766	-	-0.747	1.09E-03
TC585103	MGLL protein	-0.747	2.62E-03
TC578726	-	-0.746	1.80E-04
AK346823	MLN010061B03	-0.745	3.02E-03
DN104045	-	-0.741	2.45E-05
TC625653	-	-0.740	2.18E-05
AK395554	-	-0.735	1.18E-03
TC545140	-	-0.734	8.28E-05
ENSSSCT00000009350	ZNF512	-0.733	2.20E-04
TC606004	-	-0.733	2.70E-04
TC559220	-	-0.732	1.83E-05
TC564362	-	-0.731	6.40E-04
AJ657631	-	-0.730	3.30E-04
TC566906	-	-0.727	5.10E-04
TC595575	-	-0.725	6.41E-07

TC527868	-	-0.725	1.27E-03
TC526256	-	-0.719	6.10E-04
A_72_P214557	-	-0.719	2.62E-03
TC563260	-	-0.714	7.30E-04
TC535761	-	-0.708	3.09E-03
TC543396	-	-0.706	1.10E-04
BX914448	BX914448	-0.705	2.23E-03
TC535677	-	-0.695	1.81E-03
TC563217	-	-0.694	2.30E-04
TC524112	-	-0.693	2.90E-04
EW642054	-	-0.691	1.89E-05
A_72_P435344	-	-0.690	2.74E-03
TC539577	-	-0.689	2.60E-04
TC582041	-	-0.689	1.39E-03
AK236406	LSM6	-0.689	2.23E-03
TC559573	-	-0.687	1.10E-03
ENSSSCT00000017757	Ubiquitin specific peptidase 40	-0.687	1.24E-03
AK345540	-	-0.677	9.00E-04
TC601044	-	-0.676	2.14E-05
AK346354	C-1-tetrahydrofolate synthase, cytoplasmic (MTHFD1)	-0.675	2.33E-05
AK351744	THY010072F12	-0.675	1.25E-03
NM_001123217	ZNF217	-0.674	1.51E-06
ENSSSCT00000025705	poly(ADP-ribose) polymerase family member 4 (PARP4)	-0.674	7.25E-05
EV981363	-	-0.674	1.71E-03
ENSSSCT00000012586	Succinate-CoA ligase. GDP-forming. beta subunit	-0.669	3.10E-04
TC532469	-	-0.668	1.34E-03
AK351999	THY010123D06	-0.664	2.71E-03
ENSSSCT00000006821	Tocopherol (alpha) transfer protein	-0.663	9.10E-04
TC547188	-	-0.658	7.48E-08
TC627042	-	-0.658	3.80E-04
ENSSSCT00000007361	hydroxyacid oxidase 2 (HAO2)	-0.658	1.45E-03
TC582363	-	-0.656	3.10E-04
A_72_P404593	-	-0.655	8.60E-05
TC568444	-	-0.651	1.36E-03
CK454133	-	-0.650	3.50E-04
TC613549	-	-0.647	1.73E-07
A_72_P399733	-	-0.645	2.70E-04
CO992076	Endometrium gilt D6 of estrous cycle	-0.643	4.28E-08
ENSSSCT00000007599	ODF2L	-0.643	4.40E-04
TC608215	-	-0.642	1.40E-04
TC555476	-	-0.642	4.50E-04
NM_001185143	SLA-DOA	-0.642	9.20E-04
TC619505	-	-0.641	5.60E-04
AK397101	DCTN4	-0.641	1.14E-03
TC529140	-	-0.639	2.08E-03

DN112306	-	-0.638	5.81E-06
TC550764	-	-0.638	1.70E-04
ENSSSCT00000009138	Rho GTPase activating protein 25 (ARHGAP25)	-0.637	9.27E-06
EW380108	-	-0.637	1.03E-05
TC535187	-	-0.636	6.63E-05
GT640554	-	-0.636	2.28E-03
TC525370	-	-0.635	8.85E-05
TC620229	-	-0.632	1.89E-05
TC627496	-	-0.630	1.70E-04
AK348827	PBL010036G07	-0.630	9.40E-04
TC528683	-	-0.624	1.17E-05
BP437739	BP437739	-0.623	2.20E-04
TC609759	-	-0.618	5.59E-07
TC585025	-	-0.617	1.08E-03
ENSSSCT00000023742	-	-0.617	2.36E-03
ENSSSCT00000013169	PAX3 and PAX7 binding protein 1 (PAXBP1)	-0.615	2.63E-05
ENSSSCT00000007600	outer dense fiber of sperm tails 2-like (ODF2L)	-0.613	1.02E-07
BF704296	-	-0.612	1.08E-05
A_72_P134286	-	-0.609	5.89E-05
XR_116123	LOC100521813	-0.609	6.63E-05
AK230830	GPCPD1	-0.604	6.30E-04
TC564615	-	-0.603	6.00E-04
TC590206	-	-0.602	5.60E-04
ENSSSCT00000018493	STC2	-0.601	3.80E-04
TC586934	-	-0.599	2.49E-03
TC552040	-	-0.596	1.20E-04
TC524236	-	-0.595	1.20E-04
TC522007	-	-0.594	5.37E-05
AK236308	-	-0.593	9.87E-07
TC563641	-	-0.592	4.79E-05
AK231171	SCNM1	-0.589	4.79E-05
CO992687	Endometrium gilt D6 of estrous cycle	-0.585	4.35E-05
TC533634	-	-0.585	8.85E-05
TC554060	-	-0.582	1.97E-03
TC591669	-	-0.581	2.56E-03
TC570334	-	-0.580	1.24E-05
ENSSSCT00000014563	LGR4	-0.580	1.71E-03
TC602623	-	-0.575	5.10E-04
TC539384	-	-0.572	1.50E-04
TC621175	-	-0.572	1.48E-03
TC623527	-	-0.571	6.09E-06
TC576808	-	-0.568	3.02E-03
AK347987	-	-0.566	1.79E-05
ENSSSCT00000033054	LENG8-002	-0.562	1.24E-05
TC525615	-	-0.562	1.91E-03

TC564224	-	-0.561	4.10E-04
ENSSSCT00000029598	phosphatidylinositol glycan anchor biosynthesis, class H (PIGH)	-0.560	2.51E-03
TC612329	-	-0.558	2.88E-03
ENSSSCT00000014644	Zinc finger. BED-type containing 5	-0.556	1.11E-03
TC530147	-	-0.555	2.30E-04
TC560803	-	-0.554	2.75E-03
ENSSSCT00000005463	alpha- and gamma-adaptin binding protein (AAGAB)	-0.551	1.46E-03
DB804246	-	-0.550	3.20E-04
AK231705	DHX57	-0.548	2.09E-03
TC522516	-	-0.547	1.71E-03
ENSSSCT00000006814	phosphodiesterase 7A (PDE7A)	-0.543	1.02E-07
TC591345	-	-0.542	6.33E-05
TC593193	-	-0.542	9.80E-04
TC564838	-	-0.542	2.15E-03
AK346233	LOC100515741	-0.541	3.20E-04
TC527051	-	-0.539	2.32E-03
TC573239	-	-0.538	6.09E-06
TC563224	-	-0.538	2.00E-04
DT328281	-	-0.537	2.00E-03
TC559375	-	-0.533	3.20E-07
TC558646	-	-0.533	1.50E-04
ENSSSCT00000007387	MAB21L3	-0.530	3.27E-05
TC627355	-	-0.530	4.40E-04
TC546925	-	-0.530	2.24E-03
TC520959	-	-0.525	3.90E-04
XM_003128194	JARID2	-0.525	1.91E-03
CK466533	-	-0.525	2.83E-03
A_72_P429354	proteasomal ATPase associated factor 1 (PAAF1)	-0.524	2.00E-04
TC576666	MGC179351	-0.524	2.03E-03
TC547167	-	-0.523	1.52E-05
AK237396	SIK2	-0.523	5.10E-05
AK346547	MLN010009C11	-0.520	2.36E-03
TC557192	-	-0.518	2.60E-04
TC522670	-	-0.518	1.11E-03
TC568446	-	-0.518	1.36E-03
AK400880	CLTA	-0.517	1.56E-03
TC620901	-	-0.516	3.80E-04
TC592650	-	-0.516	1.16E-03
ENSSSCT00000012536	Calcium channel. voltage-dependent (CACNA1D)	-0.515	1.43E-03
BQ599706	-	-0.515	3.01E-03
TC528733	-	-0.511	3.40E-04
AK398132	-	-0.511	1.16E-03
ENSSSCT00000006875	CCAAT/enhancer binding protein (C/EBP), delta (CEBPD)	-0.510	2.65E-03
ENSSSCT00000004577	CITED2	-0.509	1.25E-03
TC558145	-	-0.508	4.50E-04

XM_001928236	Large subunit GTPase 1 homolog (LSG1)	-0.507	1.89E-05
TC531951	-	-0.506	1.49E-06
EW527469	-	-0.505	1.29E-05
TC586403	-	-0.504	1.79E-05
AK390557	LOC102159436	-0.504	1.82E-03
TC566914	-	-0.503	5.40E-04
TC564134	-	-0.502	2.11E-03
BX677284	BX677284	-0.501	1.24E-03
**WI+CS6h-C versus Ctl-C** (Up-regulated genes)			
TC611155	-	3.121	7.31E-12
ENSSSCT00000002650	Proto-oncogene c-Fos (FOS)	2.477	1.39E-09
NM_001123113	Fos proto-oncogene, AP-1 transcription factor subunit	2.283	1.58E-08
NM_001190276	nuclear receptor subfamily 4, group A, member 2 (NR4A2)	2.241	2.44E-06
TC529750	-	1.878	8.16E-10
NM_214069	Fucosyltransferase 2 (FUT2)	1.777	7.48E-08
DR066068	-	1.695	5.70E-04
NM_001097505	BTG2	1.635	2.33E-05
AK234282	-	1.611	9.50E-04
AK389182	FAM43B	1.471	5.70E-04
JN899104	small nucleolar RNA, C/D box 3 cluster (pSNORD3)	1.408	3.80E-04
NM_001130532	par-6 family cell polarity regulator beta (PARD6B)	1.403	1.20E-04
ENSSSCT00000007604	Cysteine-rich angiogenic inducer 61 (CYR61)	1.367	3.20E-04
NM_214214	C-C motif chemokine 2 (CCL2)	1.339	2.63E-05
TC525402	Pleckstrin homology-like domain family A member 2	1.335	2.30E-04
TC570078	-	1.282	1.04E-07
TC552941	-	1.266	3.46E-14
NM_213868	Ficolin (collagen/fibrinogen domain containing lectin) 2 (FCN2)	1.253	1.25E-03
TC564655	-	1.236	2.80E-04
TC566133	-	1.229	8.46E-06
ENSSSCT00000015660	Early growth response protein 1 (EGR1)	1.174	1.62E-08
ENSSSCT00000010370	DIS3 homolog, exosome endoribonuclease and 3'-5' exoribonuclease	1.164	2.10E-03
ENSSSCT00000031521	LY6/PLAUR domain containing 6 (LYPD6)	1.150	7.53E-05
ENSSSCT00000012922	B-cell lymphoma 6 protein (BCL6)	1.145	1.47E-03
NM_001185171	FAM204A	1.098	2.66E-03
ENSSSCT00000001267	histone H2A type 1-like (HIST1H2AB)	1.086	4.38E-05
TC575360	-	1.079	1.41E-03
TC521842	-	1.075	5.68E-05
AJ660252	-	1.055	1.34E-05
NM_001113447	phytanoyl-CoA 2-hydroxylase (PHYH)	1.002	1.43E-08
AK351387	-	0.994	1.71E-09
AJ648501	-	0.979	1.46E-03
XM_001926022	pantothenate kinase 1 (PANK1)	0.968	3.70E-04
ENSSSCT00000033656	CLDN4	0.935	6.10E-04
TC523118	-	0.933	4.06E-06

ENSSSCT00000024134	phytanoyl-CoA 2-hydroxylase (PHYH)	0.919	3.20E-06
ENSSSCT00000001271	LOC100154508	0.910	4.10E-04
TC548138	-	0.907	9.40E-04
AK400564		0.897	5.30E-04
NM_001039746	fatty acid binding protein 5 (FABP5)	0.867	1.80E-03
NM_001161637	Claudin 4 (CLDN4)	0.856	1.18E-03
NM_001109944	PEG10	0.844	1.10E-04
TC618296	CCAAT/enhancer binding protein (C/EBP) (CEBPA)	0.839	1.86E-06
TC569507	-	0.834	1.03E-03
DY407595	-	0.827	2.10E-04
ENSSSCT00000018567	Cytoplasmic FMR1 interacting protein 2	0.818	2.88E-03
ENSSSCT00000026137	histone cluster 1, H2bo	0.811	2.90E-06
TC588710	-	0.809	6.70E-04
EW177256	-	0.804	9.23E-07
TC537380	-	0.801	2.67E-06
TC601667	-	0.795	1.39E-03
ENSSSCT00000004344	Serine/threonine-protein kinase (PLK3)	0.791	1.23E-03
TC581152	-	0.771	5.70E-04
ENSSSCT00000023491	Histone H4	0.768	4.35E-05
AK347880	-	0.752	1.88E-03
ENSSSCT00000026997	Histone H4 (HIST1H4A)	0.750	6.90E-04
ENSSSCT00000031509	Nestin (NES)	0.749	2.69E-03
ENSSSCT00000001263	Histone cluster 1, H2bh	0.744	3.22E-08
TC586066	-	0.736	1.34E-05
TC567103	-	0.735	8.29E-05
TC533770	ENSP00000381892	0.720	1.34E-03
NM_001243656	HIST1H2BD	0.719	1.83E-07
ENSSSCT00000024917	K(lysine) acetyltransferase 2B (KAT2B)	0.716	2.00E-04
BX920940	-	0.716	2.60E-04
NM_214299	Heparin-binding EGF-like growth factor (HBEGF)	0.712	1.44E-03
NM_001037965	Inhibitor of DNA binding 2 (ID2)	0.709	1.76E-03
TC574741	-	0.698	1.51E-06
AK400200	LOC102159136	0.693	2.25E-03
ENSSSCT00000011511	Ubiquitin domain containing 1 (UBTD1)	0.673	1.97E-03
ENSSSCT00000011534	ATP binding cassette subfamily C member 2 (ABCC2)	0.672	2.65E-03
ENSSSCT00000031203	FAM89A	0.671	2.50E-04
TC536883	-	0.666	2.04E-08
NM_213919	Aurora kinase B (AURKB)	0.666	2.33E-03
TC589536	-	0.658	1.41E-03
TC610889	-	0.657	1.70E-05
TC531463	-	0.657	5.60E-04
TC525538	dual specificity tyrosine phosphorylation regulated kinase 2 (DYRK2)	0.656	8.32E-06
ENSSSCT00000026371	ADAM metallopeptidase domain 9 (ADAM9)	0.654	1.70E-04
BX921275	BX921275	0.650	2.96E-03
ENSSSCT00000016238	Integrator complex subunit 4	0.646	1.89E-03

BI402663	-	0.645	5.70E-04
AK233798	-	0.643	4.08E-06
A_72_P443589	-	0.640	2.10E-04
A_72_P322198	-	0.637	2.00E-03
XM_001929359	ATP binding cassette subfamily C member 2 (ABCC2)	0.632	1.40E-04
EW461356	-	0.624	2.90E-03
AK230863	NIPSNAP3A	0.608	1.74E-03
NM_001097504	Heat shock protein family H (Hsp110) member 1 (HSPH1)	0.606	2.59E-06
ENSSSCT00000005603	DHRS7	0.604	2.82E-03
NM_213766	Heat shock protein 70 (HSP70)	0.603	2.08E-03
ENSSSCT00000011497	TLL2	0.600	3.02E-03
TC595337	-	0.599	2.06E-03
ENSSSCT00000010279	Alpha-1,2-mannosyltransferase (ALG11)	0.595	8.46E-06
DY417616	-	0.591	2.46E-05
TC625344	-	0.591	2.50E-04
NM_213790	decapping enzyme, scavenger (DCPS)	0.580	3.08E-03
XM_003132024	N-acetylglucosaminyl-phosphatidylinositol de-N-acetylase-like (PIGL)	0.579	5.80E-04
ENSSSCT00000011691	SEC23 interacting protein	0.578	8.69E-05
AK345185	LOC100156940	0.576	1.07E-03
ENSSSCT00000011452	PPP1R3C	0.569	6.10E-04
AK343570	SP110	0.568	2.51E-05
TC572525	-	0.565	4.11E-06
DN122400	-	0.565	1.81E-03
TC588425	-	0.562	3.85E-05
NM_001113446	CHORDC1	0.553	7.30E-04
ENSSSCT00000010344	Kelch repeat and BTB domain containing	0.552	1.22E-03
AK392417	-	0.548	1.20E-04
ENSSSCT00000024760	RAB GTPASE Activating 1 (RABGAP1)	0.543	5.00E-04
AK394292	EI24	0.535	1.42E-03
NM_001098596	Crystallin, zeta (quinone reductase)-like 1 (CRYZL1)	0.534	2.00E-04
AK395310	DNAJB1	0.534	2.01E-03
TC559311	-	0.531	2.30E-04
NM_001243919	CUEDC1	0.531	3.20E-04
ENSSSCT00000010405	dopachrome tautomerase (DCT)	0.531	2.67E-03
FJ750950	Prostaglandin F receptor (PTGFR)	0.530	4.70E-04
CN156022	-	0.528	1.96E-03
NM_001243907	Heat shock 70kDa protein 8 (HSPA8)	0.524	1.39E-06
TC603080	-	0.521	3.20E-04
TC562874	-	0.520	2.28E-03
TC593748	-	0.515	1.57E-05
ENSSSCT00000015372	ENC1	0.511	6.00E-04
ENSSSCT00000027156	CDR2L	0.511	2.94E-03
TC532904	-	0.509	3.60E-04
ENSSSCT00000013027	Uroplakin 1B (UPK1B)	0.509	2.71E-03
ENSSSCT00000033196	Tumor necrosis factor-alpha-converting enzyme (TACE)	0.508	3.00E-04

TC591601	Pleckstrin homology domain-containing A 2-like	0.508	5.70E-04
ENSSSCT00000011617	Adducin 3 (ADD3)	0.507	5.89E-06
ENSSSCT00000030581	CRYZL1	0.505	8.90E-04
ENSSSCT00000004200	wntless WNT ligand secretion mediator	0.504	1.80E-04
AK392230	DBX2	0.500	1.48E-03
**WI+CS24h-C versus Ctl-C** (Down-regulated genes)			
A_72_P231427	Cell division control protein 42 (CDC42) effector protein 1 (CDC42EP1)	-3.435	1.00E-04
NM_001167835	Cytochrome P450 2C42 (CYP2C42)	-3.316	6.46E-04
NM_213850	Glutathione S-transferase alpha 2 (GSTA2)	-2.575	5.10E-04
NM_001159306	Pyruvate dehydrogenase kinase isozyme 4, mitochondrial (PDK4)	-2.431	6.06E-05
TC534148	-	-2.396	3.28E-08
FD592765	-	-2.371	2.92E-06
TC602959	-	-2.361	8.89E-13
NM_214109	Chymotrypsin-like elastase family, member 2A (CELA2A)	-2.224	2.55E-03
TC613147	-	-2.150	1.97E-03
ENSSSCT00000015537	Cysteine Dioxygenase Type 1 (CDO1)	-2.066	3.95E-07
KP735782	isolate S1-V1 glutathione S-transferase A2 (GSTA2)	-2.003	3.36E-04
TC541539	-	-1.725	3.71E-06
ENSSSCT00000026393	Cytochrome P450 2C34 (CYP2C34)	-1.724	8.60E-03
TC584676	-	-1.702	7.52E-07
TC548575	-	-1.670	8.56E-06
AK232564	-	-1.657	1.43E-05
TC622808	-	-1.560	1.84E-03
NM_001078665	Chemokine ligand 26-like (CCL26) (MIP-4a, eotaxin-3)	-1.483	9.22E-07
TC631544	-	-1.466	1.65E-06
TC623278	Sex comb on midleg-like 1 (SCML1)	-1.434	1.76E-03
DN134227	-	-1.402	8.85E-05
TC590364	-	-1.365	6.85E-06
AK345473	-	-1.359	1.35E-04
TC562631	-	-1.352	7.11E-07
TC538121	-	-1.349	4.13E-10
CN163990	-	-1.340	1.40E-06
AK236120	CALHM2	-1.330	9.29E-04
TC530663	-	-1.327	5.08E-04
TC566965	-	-1.296	3.48E-03
AK238381	-	-1.282	1.58E-03
TC598771	-	-1.277	9.74E-06
TC592596	-	-1.226	2.64E-04
CK452842	-	-1.202	3.10E-03
TC604056	-	-1.198	4.47E-04
TC569278	-	-1.193	2.29E-04
TC548865	-	-1.189	5.61E-07
ENSSSCT00000035428	Arginase 2 (ARG2)	-1.185	7.28E-06
TC590865	-	-1.179	4.72E-03

FD604545	-	-1.175	3.38E-04
AK237295	GPCPD1	-1.170	3.13E-04
TC571068	-	-1.154	2.56E-06
TC559094	-	-1.145	1.65E-06
AK345001	MX2	-1.135	1.33E-03
TC547062	-	-1.133	4.52E-03
TC536661	-	-1.130	1.82E-06
CV871207	-	-1.127	3.61E-05
TC541610	-	-1.123	3.73E-03
NM_001128474	Guanylate binding protein 2, interferon-inducible (GBP2)	-1.117	7.11E-07
TC542297	-	-1.116	5.76E-03
XM_013981732	GNAT1	-1.104	2.36E-06
AK232386	MX2	-1.099	3.15E-04
NM_001287412	Nuclear receptor subfamily 1H4 (NR1H4)	-1.086	1.55E-04
TC526612	-	-1.080	6.09E-04
TC597211	-	-1.071	6.67E-03
NM_001243452	DNA-damage-inducible transcript 4 (DDIT4)	-1.069	8.38E-05
TC576734	Inter-alpha-trypsin inhibitor heavy chain H1 precursor	-1.057	1.50E-03
TC593206	-	-1.047	1.31E-03
TC623045	-	-1.045	2.84E-04
TC584417	-	-1.040	5.31E-03
TC557164	2'-5' oligoadenylate synthetase 2 (OAS)	-1.038	1.12E-02
BF713827	-	-1.038	3.59E-03
TC547562	Zinc finger protein 473 (ZNF473)	-1.036	3.14E-05
NM_213962	Hydroxyacyl-CoA dehydrogenase trifunctional multienzyme complex subunit alpha (HADHA)	-1.033	3.64E-03
ENSSSCT00000023435	TNF receptor associated factor 3 (TRAF3)	-1.031	2.37E-06
DN102262	-	-1.029	1.95E-04
EW262869	-	-1.028	7.59E-05
TC543158	Histamine N-methyltransferase	-1.027	4.47E-05
ENSSSCT00000034425	Mitogen-Activated Protein Kinase Kinase Kinase 8 (MAP3K8)	-1.015	3.63E-06
TC532659	-	-1.015	1.31E-03
ENSSSCT00000010573	ADAM-like, decysin 1 (ADAMDEC1)	-1.011	1.29E-04
TC549936	-	-0.997	1.03E-02
TC531633	-	-0.997	1.70E-06
ENSSSCT00000001838	Peroxisomal biogenesis factor 6 (PEX6)	-0.995	4.50E-03
TC610412	-	-0.995	3.38E-04
TC560156	-	-0.995	7.75E-08
TC610430	-	-0.994	3.90E-03
TC583840	-	-0.986	5.17E-03
AK346015	-	-0.979	1.14E-02
AK232543	-	-0.969	4.92E-04
TC525588	-	-0.968	1.44E-03
NM_001097416	MX2	-0.967	6.46E-04
AK236259	TRMT6	-0.966	9.52E-04

AK237715	SH3BP2	-0.964	5.80E-13
TC580089	-	-0.963	3.28E-04
AK393873	-	-0.958	2.58E-04
NM_001315766	Carboxylesterase	-0.953	1.04E-02
CK454137	-	-0.952	1.77E-03
TC602526	-	-0.941	1.00E-04
TC594147	-	-0.939	4.80E-03
NM_001195115	Solute carrier family 25, member 27 (SLC25A27)	-0.938	1.69E-04
TC528319	-	-0.937	7.86E-03
TC574870	-	-0.931	3.23E-05
TC602146	-	-0.931	4.03E-03
TC601797	Carnitine O-palmitoyltransferase 2, mitochondrial precursor	-0.928	3.67E-04
CN157777	-	-0.926	4.09E-04
TC545802	-	-0.924	9.47E-04
AK349941	SKNB10098B09	-0.913	3.14E-03
TC599915	-	-0.910	1.42E-07
XR_001309264	LOC102158887	-0.910	1.86E-04
TC576690	-	-0.909	4.64E-03
NM_001101027	CYP39A1	-0.909	4.64E-06
AK350303	SPL010030C03	-0.897	7.82E-03
AK399777	SRSF5	-0.891	5.10E-06
TC598894	-	-0.890	2.33E-03
TC526535	-	-0.889	1.83E-03
TC575827	-	-0.888	7.91E-05
TC522339	-	-0.886	7.00E-03
ENSSSCT00000004670	Tumor protein D52-like 1 (TPD52L1)	-0.885	2.74E-05
NM_001134349	Kruppel-like factor 15 (KLF15)	-0.872	7.84E-06
AK350920	-	-0.870	1.15E-05
TC557067	-	-0.870	8.40E-04
TC609498	-	-0.870	2.73E-03
BP446680	-	-0.866	2.22E-06
TC608733	-	-0.865	9.95E-03
TC627807	LOC106504276	-0.865	4.09E-04
AK347470	LOC100624597	-0.861	4.42E-04
AK346234	LOC100520861	-0.861	1.17E-03
AK343323	-	-0.853	3.64E-03
EW034563	-	-0.852	8.16E-04
EW200629	-	-0.851	6.22E-06
TC600327	-	-0.850	6.57E-03
NM_001097506	AN1-type zinc finger protein 5 (ZFAND5)	-0.848	2.47E-06
TC535434	-	-0.847	1.67E-03
TC555339	-	-0.846	1.53E-04
TC604030	-	-0.846	1.13E-02
TC535436	-	-0.845	1.17E-03
TC566289	-	-0.840	5.59E-03

ENSSSCT00000000716	alpha-2-macroglobulin	-0.839	1.15E-02
TC568621	-	-0.838	6.58E-04
ENSSSCT00000007615	WDR63	-0.834	7.56E-03
TC529862	-	-0.827	1.08E-04
TC550796	-	-0.827	9.32E-03
TC526891	-	-0.825	1.04E-03
DN132923	-	-0.825	1.10E-03
NM_001143719	MAP kinase interacting serine/threonine kinase 1 (MKNK1)	-0.823	4.78E-05
AJ955670	-	-0.823	2.92E-03
ENSSSCT00000015179	Arrestin domain containing 2 (ARRDC2)	-0.821	2.64E-04
NM_214425	CYP4A21	-0.817	5.44E-06
TC625961	-	-0.817	1.22E-03
TC598274	-	-0.814	7.62E-03
TC586815	-	-0.810	2.12E-03
AK349880	-	-0.808	7.37E-03
CJ030181	-	-0.807	4.62E-03
AK233531	LVRM10168F04	-0.807	8.68E-03
ENSSSCT00000002574	Serine/arginine-rich splicing factor 5	-0.807	7.11E-07
TC528988	-	-0.803	3.84E-03
ENSSSCT00000004600	Mitogen-activated protein kinase kinase kinase 5 (MEKK5)(MAP3K5)	-0.803	2.42E-03
XM_003129835	Exophilin 5 (EXPH5)	-0.801	1.84E-05
TC537402	-	-0.801	6.55E-03
NM_213742	Calcitonin-related polypeptide beta (CALCB)	-0.800	6.93E-04
DN104045	-	-0.794	9.47E-06
TC588483	-	-0.789	5.75E-03
TC527153	-	-0.788	4.78E-03
BX677249	-	-0.781	8.48E-04
XM_013978816	Annexin A3 (ANXA3)	-0.780	6.37E-05
BX667161	-	-0.779	1.50E-03
AK394207	LVRM10091F11	-0.778	5.17E-05
TC521859	-	-0.775	3.89E-03
TC548784	-	-0.772	3.31E-03
TC621729	-	-0.767	4.26E-03
TC540384	-	-0.767	1.00E-02
NM_001098590	HPS6	-0.766	6.45E-03
TC595575	-	-0.765	2.91E-07
CV873561	-	-0.762	5.97E-03
DY405756	-	-0.759	8.40E-04
TC542747	-	-0.753	1.03E-04
TC614889	-	-0.752	5.56E-04
DV904193	F10R porcine skeletal muscle	-0.749	7.49E-03
AK352378	VPS51	-0.748	2.57E-03
TC529859	-	-0.746	2.93E-03
ENSSSCT00000005804	Transient receptor potential cation channel M-6 (TRPM6)	-0.746	4.50E-03
TC531472	-	-0.744	2.04E-03

TC550764	-	-0.742	2.23E-05
TC610393	-	-0.737	3.59E-03
AK394979	LOC100156195	-0.737	5.35E-04
XM_003128430	TNFRSF21	-0.735	7.13E-03
TC567905	-	-0.732	6.77E-03
TC599345	-	-0.731	6.89E-04
TC538132	-	-0.726	5.52E-03
TC519529	-	-0.725	3.89E-05
ENSSSCT00000034119	LOC100516891	-0.724	2.13E-07
AK351081	-	-0.722	5.71E-03
CN157151	-	-0.718	6.49E-03
EW261758	-	-0.717	2.91E-07
TC587447	-	-0.716	3.57E-03
TC527857	-	-0.714	7.72E-03
TC523245	-	-0.711	1.20E-03
ENSSSCT00000022976	Rho guanine nucleotide exchange factor (GEF) 3	-0.706	1.69E-03
AK396196	-	-0.705	9.27E-05
TC559220	-	-0.703	3.90E-05
ENSSSCT00000028241	SMAD specific E3 ubiquitin protein ligase 1	-0.701	1.15E-03
BX675220	-	-0.700	8.85E-05
NM_001243362	Lactamase, beta (LACTB)	-0.698	1.69E-03
NM_001123217	ZNF217	-0.698	9.70E-07
BF704296	-	-0.694	1.65E-06
TC585103	MGLL protein	-0.693	7.00E-03
TC563260	-	-0.693	1.29E-03
TC526256	-	-0.691	1.23E-03
DY437600	-	-0.689	1.07E-02
TC625653	-	-0.685	8.23E-05
ENSSSCT00000012586	Succinate-CoA ligase, GDP-forming, beta subunit	-0.685	2.81E-04
TC594876	-	-0.684	7.58E-03
TC605363	-	-0.681	7.85E-03
TC545140	-	-0.680	2.69E-04
TC555462	-	-0.677	6.20E-06
NM_001024696	TNFSF10	-0.677	7.46E-03
TC568032	-	-0.675	1.18E-03
TC533014	-	-0.669	6.68E-03
CO992076	Endometrium gilt D6 of estrous cycle pd6end	-0.669	2.52E-08
ENSSSCT00000009138	Rho GTPase activating protein 25 (ARHGAP25)	-0.667	5.17E-06
TC527407	-	-0.667	1.21E-03
AK232808	LVRM10030B02	-0.666	2.37E-04
TC551004	-	-0.666	2.73E-03
TC547188	-	-0.663	7.75E-08
AK349343	-	-0.659	9.29E-04
AK230830	GPCPD1	-0.658	2.77E-04
TC538766	-	-0.658	5.22E-03

TC543254	-	-0.657	4.82E-03
TC535677	-	-0.655	4.46E-03
TC546244	-	-0.655	7.45E-03
DN120432	-	-0.655	5.22E-03
TC608215	-	-0.654	1.32E-04
TC529140	-	-0.654	2.23E-03
AJ963247	-	-0.653	1.02E-02
NM_001185143	SLA-DOA	-0.652	9.95E-04
TC555476	-	-0.652	4.63E-04
EV986999	-	-0.650	9.64E-03
ENSSSCT00000009350	ZNF512	-0.644	1.24E-03
DB804246	-	-0.643	4.77E-05
TC559573	-	-0.641	3.00E-03
TC591816	-	-0.641	2.52E-03
AK231832	-	-0.636	6.48E-03
TC606004	-	-0.636	1.77E-03
TC613549	-	-0.634	2.91E-07
AK236308	-	-0.633	3.54E-07
TC564362	-	-0.633	3.90E-03
CK454133	-	-0.631	6.41E-04
TC539577	-	-0.627	1.01E-03
ENSSSCT00000025705	Poly(ADP-ribose) polymerase family member 4 (PARP4)	-0.624	2.46E-04
NM_213851	Phosphoinositide-3-kinase, regulatory subunit 5 (PIK3R5)	-0.623	6.16E-03
ENSSSCT00000017757	Ubiquitin specific peptidase 40	-0.623	4.46E-03
TC592842	-	-0.622	6.89E-03
TC542180	-	-0.621	1.06E-02
TC532469	-	-0.620	3.90E-03
AK234441	LOC100153598	-0.619	7.73E-03
NM_001143722	Phosphatidylinositol transfer protein, cytoplasmic 1 (PITPNC1)	-0.614	3.35E-03
ENSSSCT00000007361	Hydroxyacid oxidase 2 (HAO2)	-0.614	3.97E-03
AJ657631	-	-0.613	2.98E-03
TC530781	-	-0.613	6.48E-03
AK351999	THY010123D06	-0.612	7.52E-03
TC533634	-	-0.609	5.84E-05
ENSSSCT00000018493	STC2	-0.606	4.23E-04
TC528683	-	-0.603	2.28E-05
AK391669	CITED2	-0.603	4.57E-03
DN112306	-	-0.602	1.63E-05
TC627042	-	-0.600	1.37E-03
AK237396	SIK2	-0.599	6.87E-06
TC568444	-	-0.598	4.34E-03
AK347987	-	-0.598	7.84E-06
TC606417	SJCHGC06720 protein	-0.597	6.16E-03
ENSSSCT00000001143	HIVEP1	-0.594	4.58E-03
TC548599	-	-0.593	9.38E-03

AK236406	LSM6	-0.593	1.15E-02
AK351744	THY010072F12	-0.591	6.09E-03
TC578726	-	-0.591	3.24E-03
ENSSSCT00000023179	RAS homolog family member U (RHOU)	-0.585	4.79E-03
TC564615	-	-0.585	1.09E-03
ENSSSCT00000011130	Mitogen-activated protein kinase kinase kinase 21 (MLK4)	-0.584	3.64E-03
GT640554	-	-0.584	6.81E-03
TC618316	-	-0.584	3.10E-03
TC535187	-	-0.583	2.58E-04
AK346354	C-1-tetrahydrofolate synthase, cytoplasmic (MTHFD1)	-0.582	2.37E-04
TC606037	-	-0.580	4.52E-03
TC524112	-	-0.578	2.93E-03
ENSSSCT00000029598	phosphatidylinositol glycan anchor biosynthesis, class H (PIGH)	-0.578	2.53E-03
AK345509	CLEC1A	-0.578	8.96E-03
TC543396	-	-0.577	1.58E-03
AK398979	MLN010035G02	-0.572	6.29E-03
TC601044	-	-0.572	2.77E-04
NM_001245012	Inositol polyphosphate-1-phosphatase (INPP1)	-0.571	3.25E-03
TC607613	-	-0.570	7.08E-03
TC552040	-	-0.566	2.69E-04
TC591669	-	-0.564	4.68E-03
AK346233	LOC100515741	-0.562	2.42E-04
ENSSSCT00000004577	CITED2	-0.562	5.07E-04
TC620371	-	-0.562	1.09E-02
AK236978	SLA2	-0.560	1.14E-02
EW642054	-	-0.559	4.38E-04
TC546925	-	-0.558	1.78E-03
TC613011	-	-0.558	5.93E-03
TC586934	-	-0.556	6.70E-03
TC525370	-	-0.556	6.06E-04
ENSSSCT00000001879	Polymerase (POLH)	-0.556	1.23E-03
TC566906	-	-0.555	9.46E-03
ENSSSCT00000013169	PAX3 and PAX7 binding protein 1 (PAXBP1)	-0.555	1.53E-04
TC554042	-	-0.554	9.52E-04
ENSSSCT00000006821	Tocopherol (alpha) transfer protein	-0.554	7.00E-03
XM_003128651	Lysine-specific demethylase NO66-like	-0.553	9.56E-03
ENSSSCT00000023742	-	-0.551	8.75E-03
EW380108	-	-0.548	1.17E-04
TC560803	-	-0.545	4.50E-03
AK395386	SLC38A1	-0.544	6.69E-05
TC582363	-	-0.544	3.25E-03
TC532987	-	-0.544	8.25E-03
BX924155	-	-0.543	6.14E-03
TC609759	-	-0.543	5.80E-06
TC573441	-	-0.541	1.00E-02

TC585025	-	-0.540	5.40E-03
ENSSSCT00000007599	ODF2L	-0.539	3.93E-03
TC623527	-	-0.538	1.84E-05
XM_003128194	JARID2	-0.538	1.99E-03
TC562329	-	-0.537	5.00E-03
CO992687	Endometrium gilt D6 of estrous cycle pd6end	-0.537	1.82E-04
XR_116123	LOC100521813	-0.536	4.38E-04
TC529517	-	-0.536	5.40E-03
ENSSSCT00000004104	Cdk5 and Abl enzyme substrate 1	-0.534	3.37E-05
TC602623	-	-0.533	1.50E-03
TC620229	-	-0.532	2.68E-04
ENSSSCT00000005463	alpha- and gamma-adaptin binding protein (AAGAB)	-0.530	3.00E-03
ENSSSCT00000007600	outer dense fiber of sperm tails 2-like (ODF2L)	-0.527	1.82E-06
AK397953	TCH010039C03	-0.523	2.69E-04
ENSSSCT00000028294	LOC100737496	-0.523	4.28E-03
TC576666	-	-0.522	2.96E-03
TC539212	-	-0.519	5.50E-04
TC576808	-	-0.519	9.00E-03
TC554060	-	-0.519	7.64E-03
AK344560	DCI010075D05	-0.517	6.68E-03
AK392845	RNASET2	-0.516	7.58E-03
BP437739	LNG010075E10 5'	-0.513	2.57E-03
TC557192	-	-0.512	3.52E-04
TC522007	-	-0.512	4.69E-04
ENSSSCT00000007387	MAB21L3	-0.512	6.70E-05
AK348827	PBL010036G07	-0.505	1.00E-02
TC539384	-	-0.504	9.08E-04
ENSSSCT00000012536	Calcium channel, voltage-dependent, L type (CACNA1D)	-0.504	2.46E-03
ENSSSCT00000006814	phosphodiesterase 7A (PDE7A)	-0.502	5.61E-07
TC553348	-	-0.502	2.98E-04
ENSSSCT00000006875	CCAAT/enhancer binding protein (C/EBP), delta (CEBPD)	-0.501	4.34E-03
**WI+CS24h-C versus Ctl-C** (Up-regulated genes)			
TC611155	-	3.100	8.85E-12
ENSSSCT00000002650	Proto-oncogene c-Fos (FOS)	2.473	1.28E-09
NM_001123113	Fos proto-oncogene, AP-1 transcription factor subunit	2.283	1.58E-08
NM_001190276	nuclear receptor subfamily 4, group A, member 2 (NR4A2)	2.268	2.36E-06
TC529750	-	1.953	3.22E-10
NM_214069	Fucosyltransferase 2 (FUT2)	1.725	1.56E-07
DR066068	-	1.719	5.98E-04
JN899104	small nucleolar RNA, C/D box 3 cluster (pSNORD3)	1.709	3.23E-05
AK398294	Creatine kinase, M-type (CKM)	1.648	7.41E-03
NM_001097505	BTG2	1.575	4.99E-05
AK234282	-	1.508	2.53E-03
ENSSSCT00000025681	CRABP1	1.485	4.60E-03

TC611786	-	1.423	2.73E-03
AK389182	FAM43B	1.403	1.23E-03
NM_001130532	Par-6 family cell polarity regulator beta (PARD6B)	1.395	1.55E-04
TC525402	Pleckstrin homology-like domain family A member 2	1.350	2.42E-04
TC595819	-	1.314	7.96E-03
NM_001129949	CKM	1.297	3.10E-03
NM_213868	Ficolin (collagen/fibrinogen domain containing lectin) 2 (FCN2)	1.290	1.19E-03
TC570078	-	1.284	1.30E-07
TC552941	-	1.264	7.24E-14
ENSSSCT00000007604	Cysteine-rich angiogenic inducer 61 (CYR61)	1.237	1.29E-03
ENSSSCT00000012922	B-cell lymphoma 6 protein (BCL6)	1.218	1.01E-03
TC564655	-	1.207	4.63E-04
ENSSSCT00000031521	LY6/PLAUR domain containing 6 (LYPD6)	1.205	4.47E-05
TC521842	-	1.201	1.15E-05
NM_214105	Guanylate cyclase 2C (heat stable enterotoxin receptor) (GUCY2C)	1.178	6.24E-03
TC566133	-	1.177	1.92E-05
TC575360	-	1.165	8.18E-04
ENSSSCT00000001267	Histone H2A type 1-like (HIST1H2AB)	1.158	1.93E-05
AJ660252	-	1.074	1.12E-05
ENSSSCT00000015660	Early growth response protein 1 (EGR1)	1.064	1.27E-07
ENSSSCT00000033656	CLDN4	1.052	1.79E-04
AK351387	-	1.045	5.44E-10
NM_001185171	FAM204A	1.033	6.39E-03
ENSSSCT00000010370	DIS3 homolog, exosome endoribonuclease and 3'-5' exoribonuclease	1.017	9.62E-03
AK400564	-	1.013	1.35E-04
TC546352	-	1.006	1.59E-03
ENSSSCT00000018567	Cytoplasmic FMR1 interacting protein 2	0.990	4.69E-04
NM_214214	C-C motif chemokine 2 (CCL2)	0.978	1.93E-03
AK347361	HLX	0.977	5.83E-03
ENSSSCT00000001271	histone H2A type 1-F-like	0.977	2.10E-04
AJ648501	-	0.962	2.37E-03
NM_214055	IL1B1	0.954	3.86E-03
NM_001113447	phytanoyl-CoA 2-hydroxylase (PHYH)	0.939	5.47E-08
NM_001024695	CCL28	0.902	5.42E-03
XM_001926022	pantothenate kinase 1 (PANK1)	0.900	1.10E-03
ENSSSCT00000026137	Histone cluster 1, H2bo	0.889	7.11E-07
ENSSSCT00000036290	Transcription factor AP-1 (JUN)	0.888	5.75E-03
NM_001161637	Claudin 4 (CLDN4)	0.886	1.06E-03
ENSSSCT00000023491	Histone H4	0.870	6.87E-06
TC618296	CCAAT/enhancer binding protein (C/EBP) (CEBPA)	0.858	1.57E-06
NM_001031782	Krueppel-like factor 4 (KLF4)	0.849	7.41E-03
ENSSSCT00000024134	phytanoyl-CoA 2-hydroxylase (PHYH)	0.829	1.93E-05
TC523118	-	0.816	3.88E-05
NM_001160084	Claudin 19 (CLDN19)	0.815	1.11E-02
EW461356	-	0.813	1.86E-04

ENSSSCT00000016389	CRYAB	0.804	8.75E-03
AK234663	RAB3A	0.802	6.10E-03
TC574741	-	0.801	1.54E-07
AK347880	-	0.801	1.27E-03
ENSSSCT00000001263	Histone cluster 1, H2bh	0.796	8.76E-09
ENSSSCT00000014263	REST corepressor 2 (RCOR2)	0.791	2.09E-03
TC548138	-	0.790	5.01E-03
NM_001039746	fatty acid binding protein 5 (FABP5)	0.783	6.48E-03
TC566441	Tripartite motif containing 4 (TRIM4)	0.771	1.14E-02
TC586066	-	0.764	7.84E-06
ENSSSCT00000026997	Histone H4 (HIST1H4A)	0.763	7.24E-04
NM_001243656	HIST1H2BD	0.762	7.74E-08
TC537380	-	0.758	7.18E-06
ENSSSCT00000004344	Serine/threonine-protein kinase (PLK3)	0.757	2.56E-03
TC567103	-	0.756	6.40E-05
NM_213919	Aurora kinase B (AURKB)	0.746	9.60E-04
BX921275	-	0.734	1.12E-03
ENSSSCT00000024917	K(lysine) acetyltransferase 2B (KAT2B)	0.728	1.86E-04
EW177256	-	0.720	6.26E-06
TC601667	-	0.720	5.04E-03
DY407595	-	0.719	1.38E-03
NM_214299	Heparin-binding EGF-like growth factor (HBEGF)	0.703	2.20E-03
TC518112	Toll-like receptor 4 (TLR4)	0.703	1.01E-02
ENSSSCT00000031203	FAM89A	0.692	2.04E-04
ENSSSCT00000026371	ADAM metallopeptidase domain 9 (ADAM9)	0.684	1.08E-04
TC581152	-	0.674	3.20E-03
BX920940	-	0.669	7.18E-04
ENSSSCT00000031509	Nestin (NES)	0.669	9.88E-03
TC588710	-	0.652	7.52E-03
AK400200	LOC102159136	0.650	5.66E-03
XM_003126745	Lin-7 homolog A (LIN7A)	0.648	1.06E-02
NM_001037965	Inhibitor of DNA binding 2 (ID2)	0.644	6.05E-03
TC533770	ENSP00000381892	0.643	5.52E-03
ENSSSCT00000016238	Integrator complex subunit 4	0.636	3.00E-03
ENSSSCT00000011511	Ubiquitin domain containing 1 (UBTD1)	0.628	5.29E-03
TC617432	-	0.623	1.76E-03
AK230863	NIPSNAP3A	0.623	1.78E-03
AK343570	SP110	0.621	7.28E-06
AK233798	-	0.619	7.84E-06
NM_214030	Gamma-glutamyltransferase 1 (GGT1)	0.618	2.94E-03
NM_001128467	Aquaporin 6 (AQP6)	0.616	6.06E-03
ENSSSCT00000003857	EPH receptor A2 (EPHA2)	0.614	9.09E-03
TC525538	Dual specificity tyrosine phosphorylation regulated kinase 2 (DYRK2)	0.614	2.74E-05
AK231953	LNG010057E03	0.605	7.74E-08
BI402663	-	0.605	1.48E-03

AK392417	-	0.602	3.37E-05
TC625344	-	0.601	2.42E-04
TC610889	-	0.600	7.58E-05
NM_001244520	TFAP2B	0.592	4.32E-03
AJ964590	-	0.582	7.30E-03
TC597684	-	0.581	1.10E-02
TC572525	-	0.580	3.04E-06
TC588425	-	0.580	2.95E-05
TC559311	-	0.578	8.51E-05
ENSSSCT00000030542	Ubiquitin carboxyl terminal hydrolase 10	0.574	7.65E-03
TC621005	-	0.574	7.52E-03
XM_001929359	ATP binding cassette subfamily C member 2 (ABCC2)	0.571	6.07E-04
NM_214251	Transforming growth factor, alpha (TGFA)	0.567	2.46E-04
NM_214390	IL15	0.556	5.21E-03
TC536883	-	0.554	7.11E-07
DY417616	-	0.554	8.00E-05
TC532904	-	0.553	1.53E-04
ENSSSCT00000024923	FKBP14	0.549	5.95E-05
NM_001128492	Cellular retinoic acid binding protein 1 (CRABP1)	0.545	9.44E-03
ENSSSCT00000011691	SEC23 interacting protein	0.545	2.34E-04
ENSSSCT00000011497	TLL2	0.542	9.88E-03
TC534949	-	0.541	8.65E-03
DN122400	-	0.539	3.96E-03
ENSSSCT00000010279	Alpha-1,2-mannosyltransferase (ALG11)	0.538	4.77E-05
AK345185	LOC100156940	0.534	3.10E-03
ENSSSCT00000011578	Actin-related protein 1 homolog A (ACTR1A)	0.533	6.99E-03
NM_001113446	CHORDC1	0.532	1.42E-03
NM_001134353	KLF6	0.529	3.56E-04
ENSSSCT00000010315	ZC3H13	0.529	7.96E-03
ENSSSCT00000017147	Myomesin (M-protein) 2, 165kDa	0.521	2.88E-04
XM_003132024	N-acetylglucosaminyl-phosphatidylinositol de-N-acetylase-like (PIGL)	0.521	2.42E-03
ENSSSCT00000031454	PTG010033E08	0.520	1.12E-02
NM_001243407	Thioredoxin-related transmembrane protein 4 (TMX4)	0.517	7.41E-03
NM_001098596	Crystallin, zeta (quinone reductase)-like 1 (CRYZL1)	0.517	3.65E-04
NM_214067	Calpastatin (CAST)	0.516	1.14E-02
TC518652	-	0.512	2.34E-04
ENSSSCT00000031951	Histone cluster 2, H2bf	0.511	2.59E-03
ENSSSCT00000005108	LEO1	0.509	6.53E-03
ENSSSCT00000009614	Replication factor c subunit 1 activator 1	0.507	8.48E-04
ENSSSCT00000002715	Thyroid hormone receptor interactor 11 (TRIP11)	0.505	5.77E-05
ENSSSCT00000010344	Kelch repeat and BTB domain containing	0.502	4.32E-03
**WI+CS6h-C versus WI-C** (Down-regulated genes)			
NM_001167835	Cytochrome P450 2C42 (CYP2C42)	-3.025	2.57E-03
A_72_P443884	-	-2.587	4.48E-03

A_72_P054101	-	-2.552	1.61E-03
NM_001159306	Pyruvate dehydrogenase kinase isozyme 4, mitochondrial (PDK4)	-2.369	1.43E-04
NM_213850	Glutathione S-transferase alpha 2 (GSTA2)	-2.338	2.22E-03
ENSSSCT00000034581	Hemoglobin, theta 1	-2.229	3.69E-02
TC613147	-	-2.190	2.24E-03
ENSSSCT00000015537	Cysteine Dioxygenase Type 1 (CDO1)	-2.166	3.50E-07
TC534148	-	-1.893	5.68E-06
NM_214109	Chymotrypsin-like elastase family, member 2A (CELA2A)	-1.788	2.05E-02
KP735782	Isolate S1-V1 glutathione S-transferase A2 (GSTA2)	-1.784	1.86E-03
NM_001134824	CYP3A46	-1.673	5.06E-03
TC548575	-	-1.627	2.75E-05
NM_214200	Perilipin 2 (PLIN2)	-1.551	2.16E-02
AK232564	-	-1.497	1.16E-04
ENSSSCT00000026393	Cytochrome P450 2C34 (CYP2C34)	-1.423	3.95E-02
NM_001008691	Chemokine (C-X-C motif) ligand 10 (CXCL10)	-1.349	1.55E-02
NM_213962	Hydroxyacyl-CoA dehydrogenase trifunctional multienzyme complex subunit alpha (HADHA)	-1.336	2.69E-04
NM_001078665	Chemokine ligand 26-like (CCL26) (MIP-4a, eotaxin-3)	-1.333	1.19E-05
TC623278	Sex comb on midleg-like 1 (SCML1)	-1.284	6.81E-03
NM_214422	CYP3A39	-1.283	3.11E-02
ENSSSCT00000004065	Facilitator superfamily domain containing 2A (MFSD2A)	-1.281	3.09E-02
TC622808	-	-1.277	1.42E-02
NM_001038644	Angiopoietin like 4 (ANGPTL4)	-1.258	4.37E-02
TC541610	-	-1.255	1.61E-03
TC598771	-	-1.248	2.86E-05
AK345473	-	-1.192	1.07E-03
TC557164	2'-5' oligoadenylate synthetase 2 (OAS)	-1.186	4.48E-03
AK236120	CALHM2	-1.168	4.64E-03
TC525379	Intrinsic factor cobalamin receptor Vitamin B12	-1.147	2.54E-02
TC623045	-	-1.144	1.39E-04
AK238381	-	-1.139	6.68E-03
TC604056	-	-1.132	1.30E-03
NM_001114056	MHC class I antigen 5 (SLA-5)	-1.124	1.51E-03
TC569278	-	-1.103	8.87E-04
TC592596	-	-1.099	1.49E-03
NM_214185	Microsomal triglyceride transfer protein (MTTP)	-1.098	2.81E-02
TC544200	-	-1.098	3.48E-02
NM_001161753	Phosphoenolpyruvate carboxykinase 2 (mitochondrial) (PCK2)	-1.094	4.77E-02
TC541539	-	-1.082	3.14E-03
ENSSSCT00000010309	Leucine Rich Repeats And Calponin Homology Domain Containing 1 (LRCH1)	-1.079	3.28E-03
TC590364	-	-1.073	3.84E-04
AK232386	MX2	-1.063	7.67E-04
NM_001195115	SLC25A27	-1.061	4.94E-05
NM_001128474	guanylate binding protein 2, interferon-inducible (GBP2)	-1.060	4.13E-06

CK452842	-	-1.053	1.23E-02
A_72_P152666	-	-1.048	2.62E-03
ENSSSCT00000035428	Arginase 2 (ARG2)	-1.048	8.93E-05
TC610430	-	-1.045	3.11E-03
TC537819	-	-1.043	1.35E-02
NM_001113438	AQP7	-1.038	1.94E-02
A_72_P032671	-	-1.037	7.90E-03
EW262869	-	-1.035	1.15E-04
BF713827	-	-1.030	4.83E-03
CN163990	-	-1.026	1.49E-04
CV871207	-	-1.020	2.36E-04
AK237295	GPCPD1	-1.011	2.47E-03
TC566965	-	-1.005	3.12E-02
NM_214425	CYP4A21	-1.002	3.50E-07
NM_001122990	NNAT	-1.000	2.86E-04
TC536661	-	-0.998	2.75E-05
AK345001	MX2	-0.984	7.22E-03
TC528367	-	-0.981	4.35E-02
TC571068	-	-0.977	6.89E-05
FD604545	-	-0.971	4.01E-03
NM_001315766	LOC100736962	-0.963	1.18E-02
EW034563	-	-0.961	2.62E-04
NM_001044599	GADD45A	-0.959	2.75E-02
NM_001128469	ISG15	-0.959	3.20E-02
NM_001044592	F10	-0.959	1.33E-02
TC627807	LOC106504276	-0.956	1.75E-04
AK389171	GRAMD1B	-0.951	6.79E-03
NM_001246243	carnitine palmitoyltransferase 2 (CPT2)	-0.951	2.22E-04
CN153597	-	-0.942	4.58E-02
AK230683	OAS1	-0.939	4.90E-02
NM_214246	carboxylesterase 1 (CES1)	-0.936	2.20E-02
NM_001101027	CYP39A1	-0.934	6.28E-06
TC530663	-	-0.933	1.94E-02
TC610412	-	-0.929	1.17E-03
NM_001098605	PNPLA2	-0.929	3.67E-03
NM_001143719	MAP kinase interacting serine/threonine kinase 1 (MKNK1)	-0.927	1.46E-05
AK236725	OVRM10220H04	-0.925	2.15E-02
TC524151	-	-0.921	7.39E-03
NM_001097416	MX2	-0.914	1.73E-03
TC555339	-	-0.912	8.78E-05
TC548235	-	-0.910	2.46E-02
ENSSSCT00000001838	Peroxisomal biogenesis factor 6 (PEX6)	-0.909	1.19E-02
TC543985	-	-0.908	3.96E-02
AK239975	-	-0.908	1.36E-02
TC548865	-	-0.907	7.92E-05

TC598894	-	-0.895	2.97E-03
AK392845	RNASET2	-0.894	1.51E-05
NM_001134349	Kruppel-like factor 15 (KLF15)	-0.884	1.35E-05
TC549936	-	-0.883	2.96E-02
NM_001243304	CYP27A1	-0.879	4.22E-04
TC541448	-	-0.878	3.73E-02
ENSSSCT00000007615	WDR63	-0.868	6.94E-03
TC526612	-	-0.866	7.84E-03
XM_003130522	HHIP-like protein 2-like	-0.861	3.39E-02
AK343323	-	-0.851	4.69E-03
ENSSSCT00000023435	TNF receptor associated factor 3 (TRAF3)	-0.851	8.78E-05
TC597211	-	-0.851	4.11E-02
NM_001185143	SLA-DOA	-0.849	4.94E-05
NM_001044573	Angiogenin, ribonuclease, RNase A family, 5 (ANG)	-0.849	3.09E-02
NM_001287412	Nuclear receptor subfamily 1H4 (NR1H4)	-0.846	3.86E-03
AK343540	-	-0.845	1.82E-02
A_72_P344163	IQ motif containing F3 (IQCF3)	-0.844	1.63E-02
NM_001083941	ACE	-0.843	4.42E-02
TC576734	Inter-alpha-trypsin inhibitor heavy chain H1 precursor	-0.843	1.50E-02
TC566172	-	-0.839	9.95E-03
TC601797	Mitochondrial Carnitine O-palmitoyltransferase 2	-0.836	1.76E-03
TC528319	-	-0.835	2.38E-02
EV943900	-	-0.835	1.43E-02
AK347600	SF1	-0.833	3.71E-02
TC568621	-	-0.832	1.06E-03
BP446680	-	-0.827	1.04E-05
TC532659	-	-0.827	1.18E-02
ENSSSCT00000015179	Arrestin domain containing 2 (ARRDC2)	-0.827	3.66E-04
AK344079	-	-0.824	2.23E-02
NM_001163696	C13H21orf62	-0.824	2.84E-02
NM_213742	Calcitonin-related polypeptide beta (CALCB)	-0.822	7.67E-04
TC623525	-	-0.821	1.51E-02
ENSSSCT00000003289	Actinin, alpha 4 (ACTN4)	-0.820	1.51E-02
AK349880	-	-0.818	8.26E-03
TC559094	-	-0.813	4.66E-04
TC583840	-	-0.797	3.11E-02
AK351081	-	-0.797	2.96E-03
TC560156	-	-0.796	9.09E-06
AK239735	LOC100155195	-0.791	3.16E-02
TC553534	-	-0.790	1.52E-03
ENSSSCT00000004450	RNASET2	-0.787	4.51E-04
XM_013981732	GNAT1	-0.787	5.85E-04
TC547562	Zinc finger protein 473 (ZNF473)	-0.782	1.77E-03
A_72_P705022	-	-0.780	4.96E-02
ENSSSCT00000004600	Mitogen-activated protein kinase kinase kinase 5 (MEKK5)(MAP3K5)	-0.780	4.21E-03

ENSSSCT00000002106	MAN2C1	-0.780	1.92E-02
TC560294	HC12887	-0.779	3.89E-02
NM_001097452	C8G	-0.778	1.62E-03
AK236066	OVRM10149G11	-0.778	1.31E-02
TC600327	-	-0.766	1.83E-02
TC529862	-	-0.764	4.71E-04
NM_001024696	TNFSF10	-0.762	3.35E-03
AK232543	-	-0.762	7.90E-03
TC532382	-	-0.754	3.34E-02
TC545802	-	-0.750	9.44E-03
TC593206	-	-0.749	2.95E-02
NM_001097506	AN1-type zinc finger protein 5 (ZFAND5)	-0.746	3.70E-05
AK236259	TRMT6	-0.742	1.46E-02
TC548175	-	-0.738	9.79E-03
TC580089	-	-0.738	7.59E-03
ENSSSCT00000012586	Succinate-CoA ligase, GDP-forming, beta subunit	-0.734	1.80E-04
ENSSSCT00000034119	Sequence similarity 122C	-0.734	3.50E-07
ENSSSCT00000028877	Aminoacylproline aminopeptidase prolidase	-0.733	1.37E-03
TC546244	-	-0.732	3.54E-03
ENSSSCT00000028241	SMAD specific E3 ubiquitin protein ligase 1	-0.729	1.07E-03
ENSSSCT00000004670	Tumor protein D52-like 1 (TPD52L1)	-0.727	6.41E-04
NM_001129953	DMP1	-0.727	3.50E-02
NP276891	major histocompatibility complex class I antigen	-0.725	1.13E-02
TC580257	-	-0.722	2.45E-02
ENSSSCT00000019531	Acyl-CoA dehydrogenase very long chain (ACADVL)	-0.721	1.12E-02
TC602146	-	-0.715	3.57E-02
TC561223	-	-0.713	4.30E-02
TC527407	-	-0.711	8.68E-04
TC532441	-	-0.709	3.41E-02
ENSSSCT00000007254	tuftelin 1 (TUFT1)	-0.705	2.96E-03
XR_001309264	LOC102158887	-0.705	4.55E-03
CK454137	-	-0.699	2.93E-02
NM_001078670	Interferon regulatory factor 9 (IRF9)	-0.695	2.43E-02
ENSSSCT00000014563	LGR4	-0.695	4.14E-04
TC572510	-	-0.693	3.17E-02
EW200629	-	-0.690	2.33E-04
DN120432	-	-0.690	4.12E-03
NM_214119	DBI	-0.689	1.76E-02
AK393873	-	-0.689	1.08E-02
TC576690	-	-0.689	4.28E-02
AK352378	VPS51	-0.687	7.33E-03
DN102262	-	-0.685	1.67E-02
ENSSSCT00000006527	NDRG1	-0.684	2.41E-03
TC575827	-	-0.682	2.94E-03

CF365587	-	-0.678	2.87E-02
ENSSSCT00000006875	CCAAT/enhancer binding protein (C/EBP), delta (CEBPD)	-0.678	1.96E-04
TC605742	Acyl-CoA-binding protein (ACBP)	-0.676	2.18E-02
TC567770		-0.675	3.49E-02
DN112306	-	-0.672	6.28E-06
TC535434	-	-0.670	1.72E-02
A_72_P531712	Acetyl-CoA acyltransferase 2 (ACAA2)	-0.668	2.90E-02
NM_214385	prostaglandin reductase 1 (PTGR1)	-0.668	9.72E-03
TC541583	-	-0.667	4.86E-03
TC550796	-	-0.666	4.82E-02
XM_013978816	Annexin A3 (ANXA3)	-0.666	7.96E-04
NM_001190235	RING1	-0.666	4.51E-05
ENSSSCT00000027714	TP53INP1	-0.665	4.01E-02
TC529043	-	-0.663	2.76E-02
CN157151	-	-0.662	1.53E-02
TC596995	-	-0.661	1.29E-02
ENSSSCT00000002574	Serine/arginine-rich splicing factor 5	-0.661	3.33E-05
A_72_P049531	-	-0.661	3.16E-02
TC614889	-	-0.659	3.35E-03
ENSSSCT00000005761	KIAA0020	-0.658	2.15E-02
TC609498	-	-0.657	3.14E-02
TC533377	-	-0.657	2.27E-02
ENSSSCT00000036302	GADD45G	-0.655	2.97E-02
CJ030181	-	-0.655	2.86E-02
A_72_P320493	-	-0.653	6.28E-06
TC591816	-	-0.652	2.89E-03
ENSSSCT00000017757	Ubiquitin specific peptidase 40	-0.652	3.70E-03
ENSSSCT00000011130	Mitogen-activated protein kinase kinase kinase 21 (MLK4)	-0.651	1.61E-03
TC527153	-	-0.649	2.65E-02
TC588483	-	-0.646	3.11E-02
NM_001243452	DNA-damage-inducible transcript 4 (DDIT4)	-0.646	2.18E-02
TC533634	-	-0.644	4.51E-05
AK237715	SH3BP2	-0.643	2.72E-08
TC595220	-	-0.642	3.41E-03
NM_001246264	ANKS1A	-0.642	1.81E-02
AK234441	LOC100153598	-0.642	7.33E-03
TC529458	-	-0.641	3.10E-02
TC563217	-	-0.639	1.12E-03
TC538766	-	-0.638	8.36E-03
TC555476	-	-0.637	9.08E-04
NM_214155	CD247	-0.636	2.59E-02
ENSSSCT00000025705	Poly(ADP-ribose) polymerase family member 4 (PARP4)	-0.636	2.89E-04
AK345540	-	-0.633	3.19E-03
AK395885	ACOX2	-0.633	1.19E-03
TC531633	-	-0.632	1.72E-03

TC536098	-	-0.630	1.15E-02
A_72_P297874	Dysbindin domain containing 1 (DBNDD1)	-0.630	1.64E-02
A_72_P214667	-	-0.629	4.47E-02
ENSSSCT00000014828	STXBP2	-0.629	4.32E-03
DV904193	F10R porcine skeletal muscle	-0.627	3.29E-02
TC526535	-	-0.626	3.84E-02
NM_214289	UCP2	-0.626	2.70E-02
TC584301	-	-0.626	4.69E-02
NM_001101028	Acyl-CoA Oxidase 1 (ACOX1)	-0.624	8.98E-03
TC550878	-	-0.624	2.18E-02
TC608215	-	-0.622	3.84E-04
NM_001243362	Lactamase, beta (LACTB)	-0.622	6.91E-03
DB804246	-	-0.620	1.32E-04
ENSSSCT00000007361	hydroxyacid oxidase 2 (HAO2)	-0.620	4.58E-03
NM_001143722	Phosphatidylinositol transfer protein, cytoplasmic 1 (PITPNC1)	-0.620	4.01E-03
TC602959	-	-0.617	1.44E-02
TC613011	-	-0.616	3.00E-03
TC533014	-	-0.611	1.69E-02
TC606417	SJCHGC06720 protein	-0.610	6.53E-03
TC601044	-	-0.607	1.97E-04
TC621173	-	-0.605	4.21E-02
TC594876	-	-0.603	2.46E-02
TC585103	MGLL protein	-0.601	2.54E-02
NM_001243420	cold shock domain containing C2, RNA binding (CSDC2)	-0.600	1.18E-02
XM_003128430	TNFRSF21	-0.600	3.70E-02
AK349506	-	-0.599	4.58E-02
AK346234	LOC100520861	-0.598	3.25E-02
BX675220	-	-0.597	1.07E-03
NM_001031796	OAS2	-0.596	3.17E-02
BX677249	-	-0.594	1.45E-02
A_72_P226162	-	-0.593	3.48E-02
TC612901	-	-0.593	3.92E-02
AK346354	C-1-tetrahydrofolate synthase, cytoplasmic (MTHFD1)	-0.591	2.88E-04
DY405756	-	-0.589	1.23E-02
ENSSSCT00000015281	GUK1	-0.588	2.91E-02
TC586594	-	-0.588	2.72E-02
TC555936	-	-0.587	7.39E-03
NM_213966	Hydroxyacyl-CoA dehydrogenase (HADHB)	-0.585	1.67E-02
TC578726	-	-0.585	4.50E-03
TC628187	-	-0.583	4.10E-02
NM_214306	hydroxysteroid (17-beta) dehydrogenase 4 (HSD17B4)	-0.583	3.95E-02
TC539577	-	-0.583	2.99E-03
ENSSSCT00000031997	TMEM150C	-0.581	1.82E-02
ENSSSCT00000023818	SGK2, serine/threonine kinase 2	-0.581	5.91E-04
TC548784	-	-0.580	3.63E-02

TC629646	-	-0.580	1.51E-02
AK397101	DCTN4	-0.580	5.34E-03
EW261758	-	-0.580	2.12E-05
ENSSSCT00000006821	Tocopherol (alpha) transfer protein	-0.579	6.10E-03
XM_003129835	Exophilin 5 (EXPH5)	-0.578	2.01E-03
NM_214323	Junction plakoglobin (JUP)	-0.577	2.75E-02
NM_001164004	Bone gamma-carboxyglutamate (gla) protein (BGLAP)	-0.576	2.23E-03
TC543804	-	-0.575	8.77E-03
ENSSSCT00000019569	Period circadian clock 1 (PER1)	-0.573	4.48E-02
TC582363	-	-0.572	2.63E-03
ENSSSCT00000017183	interferon regulatory factor 2 (IRF2)	-0.571	1.17E-02
BX914375	-	-0.570	2.42E-02
TC526256	-	-0.567	1.06E-02
TC564362	-	-0.566	1.25E-02
NM_001244412	Heme oxygenase 2 (HMOX2)	-0.565	4.45E-03
TC529859	-	-0.564	3.23E-02
NM_001097501	LGALS3	-0.564	7.55E-03
AK396814	WDFY3	-0.564	9.67E-03
NM_001123217	ZNF217	-0.563	5.89E-05
TC539384	-	-0.562	3.39E-04
NM_001184895	PMM1	-0.562	4.01E-02
TC593193	-	-0.561	1.21E-03
AK347470	LOC100624597	-0.558	3.06E-02
TC564615	-	-0.557	2.57E-03
AK399777	SRSF5	-0.556	3.79E-03
NM_001110172	Aquaporine 3 (AQP3)	-0.556	4.17E-02
BX924155	-	-0.556	6.42E-03
AK349343	-	-0.554	6.96E-03
ENSSSCT00000024590	Histone deacetylase 10 hd10	-0.552	2.82E-02
ENSSSCT00000035772	IFITM1	-0.552	3.36E-02
TC543396	-	-0.552	3.38E-03
TC543158	Histamine N-methyltransferase	-0.550	3.50E-02
AK231832	-	-0.550	2.43E-02
TC623527	-	-0.550	2.59E-05
ENSSSCT00000010983	PIK3IP1	-0.547	3.97E-02
AK396196	-	-0.543	3.11E-03
TC610393	-	-0.543	4.31E-02
TC527232	-	-0.543	1.25E-02
ENSSSCT00000004334	mutY DNA glycosylase	-0.543	2.43E-02
TC601929	-	-0.541	9.24E-03
TC554397	-	-0.538	4.35E-02
TC619505	-	-0.536	5.92E-03
EV981363	-	-0.535	2.09E-02
DN132923	-	-0.534	4.78E-02
ENSSSCT00000002527	MTHFD1	-0.533	3.20E-02

TC526891	-	-0.532	4.71E-02
TC524236	-	-0.530	8.87E-04
CK454133	-	-0.529	5.44E-03
TC543254	-	-0.529	3.07E-02
TC569075	-	-0.528	4.69E-02
NM_001190254	SAM pointed domain containing ets transcription factor (SPDEF)	-0.527	2.69E-04
A_72_P134286	-	-0.526	7.67E-04
TC566906	-	-0.526	1.76E-02
TC584539	-	-0.525	4.31E-02
TC539212	-	-0.524	7.67E-04
TC527868	-	-0.523	3.15E-02
TC558145	-	-0.520	6.27E-04
AK398132	-	-0.520	1.72E-03
A_72_P103106	-	-0.519	2.12E-02
ENSSSCT00000008691	MGRN1	-0.519	2.89E-03
ENSSSCT00000007600	outer dense fiber of sperm tails 2-like (ODF2L)	-0.518	5.68E-06
NM_001145751	transient receptor potential cation channel, subfamily C, member 1 (TRPC1)	-0.517	5.48E-03
NM_213955	galactosamine (N-acetyl)-6-sulfate sulfatase (GALNS)	-0.516	6.14E-07
ENSSSCT00000018493	STC2	-0.515	3.54E-03
ENSSSCT00000029598	phosphatidylinositol glycan anchor biosynthesis, class H (PIGH)	-0.515	9.21E-03
NM_001144122	sarcoglycan, alpha (SGCA)	-0.515	5.52E-03
A_72_P244002	-	-0.510	4.77E-02
AK346233	LOC100515741	-0.510	1.21E-03
AK237079	NFKB2	-0.509	2.81E-03
NM_214154	TXNRD1	-0.508	1.53E-02
TC551004	-	-0.508	2.98E-02
TC592234	-	-0.506	2.75E-02
TC606004	-	-0.506	1.69E-02
AK390557	LOC102159436	-0.506	3.20E-03
TC609759	-	-0.505	3.33E-05
AK348520	RPL14	-0.504	4.35E-02
NM_213851	Phosphoinositide-3-kinase, regulatory subunit 5 (PIK3R5)	-0.504	3.51E-02
TC523688	Protein CutA homolog	-0.503	1.25E-04
TC559375	-	-0.503	2.56E-06
AY517853	WNK1	-0.502	7.40E-03
**WI+CS6h-C versus WI-C** (Up-regulated genes)			
TC611155	-	3.223	3.14E-16
BF444493	-	2.001	1.39E-03
TC529750	-	1.994	2.98E-14
NM_214069	Fucosyltransferase 2 (FUT2)	1.771	5.44E-11
AK398294	Creatine kinase, M-type (CKM)	1.681	1.28E-04
NM_001130532	Par-6 family cell polarity regulator beta (PARD6B)	1.587	7.04E-08
NM_001190276	nuclear receptor subfamily 4, group A, member 2 (NR4A2)	1.504	8.78E-06
TC611786	-	1.460	2.65E-05

ENSSSCT00000019353	PIPOX	1.416	2.82E-04
NM_001190157	PVALB	1.341	8.09E-05
TC552941	-	1.335	3.75E-19
DR066068	-	1.327	1.95E-04
NM_001097505	BTG2	1.316	4.73E-06
ENSSSCT00000002650	Proto-oncogene c-Fos (FOS)	1.311	7.52E-07
NM_001185171	FAM204A	1.304	4.40E-06
NM_213868	Ficolin (collagen/fibrinogen domain containing lectin) 2 (FCN2)	1.295	1.18E-05
ENSSSCT00000012922	B-cell lymphoma 6 protein (BCL6)	1.288	4.01E-06
TC543988	-	1.271	1.50E-04
AK389182	FAM43B	1.226	8.98E-05
NM_001129949	CKM	1.214	1.09E-04
NM_214105	Guanylate cyclase 2C (heat stable enterotoxin receptor) (GUCY2C)	1.191	1.08E-04
TC525402	Pleckstrin homology-like domain family A member 2	1.185	1.26E-05
TC570078	-	1.177	6.15E-10
TC521842	-	1.172	5.14E-08
TC595819	-	1.165	7.14E-04
A_72_P443393	-	1.162	1.58E-03
TC566133	-	1.140	1.06E-07
AK234282	-	1.136	1.03E-03
ENSSSCT00000031521	LY6/PLAUR domain containing 6 (LYPD6)	1.097	9.49E-07
DN126098	-	1.073	6.92E-05
TC553645	-	1.068	8.71E-05
TC569507	-	1.017	5.24E-07
TC605691	V-set domain containing T cell activation inhibitor 1 (VTCN1)	1.002	4.62E-04
TC575360	-	0.997	7.15E-05
AJ660252	-	0.987	1.61E-07
TC523118	-	0.977	3.18E-09
XR_001305510	LOC106508566	0.960	1.03E-04
ENSSSCT00000031509	Nestin (NES)	0.953	1.39E-06
ENSSSCT00000024134	phytanoyl-CoA 2-hydroxylase (PHYH)	0.952	3.01E-09
TC617990	LOC106507828	0.950	1.23E-03
NM_001123113	Fos proto-oncogene, AP-1 transcription factor subunit	0.948	1.45E-04
NM_214214	C-C motif chemokine 2 (CCL2)	0.946	3.92E-05
AK347361	HLX	0.931	2.02E-04
NM_001039746	fatty acid binding protein 5 (FABP5)	0.926	1.26E-05
NM_001113447	phytanoyl-CoA 2-hydroxylase (PHYH)	0.925	3.38E-11
AK398217	SFXN4	0.925	5.70E-04
XM_001926022	pantothenate kinase 1 (PANK1)	0.906	1.03E-05
AJ648501	-	0.889	9.43E-05
NM_001146126	Patatin-like phospholipase domain containing 3 (PNPLA3)	0.886	1.95E-05
ENSSSCT00000010370	DIS3 homolog, exosome endoribonuclease and 3'-5' exoribonuclease	0.867	1.31E-03
NM_001109944	PEG10	0.866	4.13E-07
TC618296	CCAAT/enhancer binding protein (C/EBP) (CEBPA)	0.862	1.74E-09

ENSSSCT00000014263	REST corepressor 2 (RCOR2)	0.833	1.25E-05
DY407595	-	0.833	1.44E-06
ENSSSCT00000033656	CLDN4	0.817	5.14E-05
NM_001004027	Heme oxygenase 1 (HMOX1)	0.814	1.53E-03
TC556782	-	0.813	3.48E-04
ENSSSCT00000030572	TUBB6	0.804	6.22E-04
EW177256	-	0.803	1.22E-09
TC566441	Tripartite motif containing 4 (TRIM4)	0.800	1.95E-04
TC537380	-	0.792	6.61E-09
ENSSSCT00000024917	K(lysine) acetyltransferase 2B (KAT2B)	0.788	2.46E-07
TC586066	-	0.783	1.13E-08
ENSSSCT00000009301	striatin, calmodulin binding protein (STRN)	0.770	2.88E-05
NM_001038694	Acyl-CoA synthetase long-chain family member 4 (ACSL4)	0.769	9.83E-04
TC548138	-	0.764	1.39E-04
AK400200	LOC102159136	0.763	1.12E-05
NM_213829	AWN	0.760	2.11E-03
A_72_P322198	Zinc finger and BTB domain containing 44 (ZBTB44)	0.755	2.78E-06
TC533770	ENSP00000381892	0.755	1.06E-05
TC601667	-	0.737	6.59E-05
A_72_P218807	GABRB2	0.736	1.36E-04
ENSSSCT00000003857	EPH receptor A2 (EPHA2)	0.723	2.53E-05
ENSSSCT00000035633	MAP2K6	0.723	5.34E-05
ENSSSCT00000015660	Early growth response protein 1 (EGR1)	0.722	3.35E-07
TC540065	Serine/threonine-protein kinase Kist	0.722	1.51E-04
NM_001031782	Krueppel-like factor 4 (KLF4)	0.718	1.04E-03
TC573830	-	0.710	3.17E-04
DN129482	-	0.702	1.77E-04
AJ964590	-	0.698	1.30E-05
A_72_P773975	-	0.696	2.06E-03
ENSSSCT00000026371	ADAM metallopeptidase domain 9 (ADAM9)	0.695	3.88E-07
TC555671	-	0.686	1.22E-04
XM_001929359	ATP binding cassette subfamily C member 2 (ABCC2)	0.678	2.45E-07
TC531463	-	0.673	4.05E-06
CA778604	-	0.671	2.97E-07
NM_001037965	Inhibitor of DNA binding 2 (ID2)	0.668	7.23E-05
TC589536	-	0.665	2.02E-05
TC607789	-	0.664	1.10E-03
A_72_P125346	SAP30-like (SAP30L)	0.657	3.07E-06
ENSSSCT00000002909	Transmembrane protein 14A (TMEM14A)	0.654	2.13E-04
ENSSSCT00000018567	Cytoplasmic FMR1 interacting protein 2	0.654	8.91E-04
TC600965	-	0.651	2.14E-04
DY417616	-	0.651	1.40E-08
XM_003124543	Amiloride-sensitive sodium channel subunit gamma-like (SCNN1G)	0.650	1.06E-03
AK240605	LOC100512960	0.649	2.52E-04
EW461055	-	0.648	1.74E-04

ENSSSCT00000011452	PPP1R3C	0.647	7.91E-07
TC588425	-	0.644	1.08E-08
TC607541	Alpha-aminoadipic semialdehyde synthase (LKR/SDH)	0.642	1.53E-03
TC574741	-	0.640	1.67E-08
TC518112	Toll-like receptor 4 (TLR4)	0.639	7.33E-04
TC573765	-	0.638	6.44E-04
ENSSSCT00000000280	IGFBP6	0.636	1.50E-03
AF245504	CYP24A1	0.634	9.33E-04
NM_001109947	ANGPTL1	0.632	1.67E-04
ENSSSCT00000017407	HOXD1	0.631	4.88E-04
NM_214352	ATPase. Ca++ transporting (ATP2B1)	0.630	1.22E-05
A_72_P144706	-	0.630	2.29E-05
NM_001044567	Trans-acting T-cell-specific transcription factor GATA3	0.629	1.93E-03
NM_214067	CAST	0.628	2.29E-05
BX920940	-	0.627	1.74E-05
TC523104	-	0.625	6.08E-05
TC588710	-	0.624	2.79E-04
TC525538	Dual specificity tyrosine phosphorylation regulated kinase 2 (DYRK2)	0.622	6.51E-08
NM_001243807	FKBP10	0.622	1.39E-04
ENSSSCT00000009614	Replication factor c subunit 1 activator 1	0.622	2.18E-07
NM_001243407	Thioredoxin-related transmembrane protein 4 (TMX4)	0.622	1.28E-05
ENSSSCT00000010315	ZC3H13	0.620	2.15E-05
NM_214286	lipoprotein lipase (LPL)	0.619	1.72E-03
A_72_P015946	DGCR8	0.618	4.33E-11
AK234663	RAB3A	0.613	2.17E-03
NM_001244417	RHOV	0.613	1.39E-05
NM_213753	TSPO	0.608	4.09E-05
NM_001097504	Heat shock protein family H (Hsp110) member 1 (HSPH1)	0.607	4.93E-09
NM_001161637	Claudin 4 (CLDN4)	0.605	1.24E-03
NM_001246253	EIF4G1	0.604	3.59E-05
TC536883	-	0.604	9.28E-11
TC528090	-	0.603	2.60E-06
EW350228	-	0.600	6.35E-04
ENSSSCT00000002071	MESP2	0.600	1.84E-03
A_72_P347573	-	0.600	5.37E-04
NM_001244700	ID1	0.599	2.08E-03
TC557923	-	0.598	7.02E-04
ENSSSCT00000010279	Alpha-1,2-mannosyltransferase (ALG11)	0.594	2.48E-08
ENSSSCT00000012056	BAMBI	0.587	4.98E-04
ENSSSCT00000011497	TLL2	0.586	9.24E-05
ENSSSCT00000030542	Ubiquitin carboxyl terminal hydrolase 10	0.585	1.38E-04
A_72_P321533	-	0.584	8.69E-04
TC569589	-	0.584	4.56E-05
TC590226	-	0.580	2.90E-04
ENSSSCT00000036684	ENTPD1	0.579	4.13E-05

ENSSSCT00000011079	Histone cell cycle regulator (HIRA)	0.573	2.78E-05
NM_001315728	OGN	0.572	2.22E-04
TC555161	-	0.570	7.77E-06
ENSSSCT00000011691	SEC23 interacting protein	0.570	6.26E-07
ENSSSCT00000005197	Microtubule associated 1A MAP 1A	0.568	1.56E-04
TC621005	-	0.567	1.92E-04
ENSSSCT00000023491	Histone H4	0.567	3.21E-05
BX921275	-	0.564	3.81E-04
ENSSSCT00000025040	NAGS	0.562	1.65E-03
ENSSSCT00000018581	GEMIN5	0.560	5.40E-05
AK389851	-	0.558	5.38E-06
TC625344	-	0.558	5.23E-06
TC581152	-	0.557	4.98E-04
ENSSSCT00000032295	GSK3B interacting protein	0.556	1.08E-05
ENSSSCT00000004439	DACT2	0.555	1.03E-03
ENSSSCT00000010405	dopachrome tautomerase (DCT)	0.554	3.21E-05
CF361654	-	0.553	7.25E-04
TC593748	-	0.549	1.34E-08
ENSSSCT00000009295	CCAAT/enhancer binding protein (C/EBP), zeta (CEBPZ)	0.548	1.61E-03
XM_003132024	N-acetylglucosaminyl-phosphatidylinositol de-N-acetylase-like (PIGL)	0.544	1.72E-05
ENSSSCT00000014676	Midnolin	0.544	1.70E-04
BX675553	-	0.544	1.49E-05
ENSSSCT00000005108	LEO1	0.543	5.58E-05
AW360167	-	0.543	1.58E-03
TC544492	-	0.543	7.88E-05
AK394292	EI24	0.541	2.03E-05
TC520488	-	0.541	1.74E-03
AK394819	-	0.541	5.61E-05
TC550626	-	0.541	4.24E-04
BI402663	-	0.540	8.35E-05
AJ954855	-	0.539	1.79E-04
A_72_P219597	-	0.537	1.58E-06
TC531386	-	0.536	4.49E-04
DN123882	-	0.534	1.24E-05
ENSSSCT00000019021	IGFBP4	0.533	1.22E-03
TC522889	-	0.530	2.14E-06
NM_001244501	Glutamate dehydrogenase 1 (GLUD1)	0.528	2.97E-04
TC584892	-	0.528	2.52E-04
AK351387	-	0.527	9.24E-07
AK233798	-	0.525	5.24E-07
TC597684	-	0.524	8.70E-04
NM_001113446	CHORDC1	0.524	2.06E-05
TC556284	-	0.523	5.06E-04
TC572525	-	0.523	4.65E-08
TC594690	-	0.522	3.83E-04

AK234955	-	0.522	8.15E-06
ENSSSCT00000004227	USP1	0.521	1.16E-05
ENSSSCT00000024760	RAB GTPASE Activating 1 (RABGAP1)	0.520	1.05E-05
TC566120	-	0.519	1.68E-04
ENSSSCT00000009568	Prominin 1 (PROM1)	0.518	2.04E-04
TC518652	-	0.517	1.18E-06
TC553228	-	0.516	3.26E-04
TC591601	Pleckstrin homology domain-containing family A2-like	0.515	4.98E-06
ENSSSCT00000029240	KAT8 regulatory NSL complex subunit 1-like	0.515	1.41E-05
ENSSSCT00000034652	Immediate Early Response 3 (IER3)	0.514	4.29E-04
ENSSSCT00000026997	Histone H4 (HIST1H4A)	0.514	1.04E-03
AK345185	LOC100156940	0.513	7.74E-05
ENSSSCT00000005460	Mothers against decapentaplegic homolog 6 (SMAD6)	0.511	1.24E-07
TC617432	-	0.510	2.94E-04
ENSSSCT00000003764	TMEM201	0.510	6.41E-07
TC595432	-	0.508	1.25E-04
ENSSSCT00000031203	FAM89A	0.507	1.29E-04
BX669300	-	0.505	5.65E-06
TC579787	Calmodulin	0.505	1.71E-03
TC608758	-	0.504	1.07E-03
BX675986	-	0.501	3.65E-04
**WI+CS24h-C versus WI-C** (Down-regulated genes)			
NM_001167835	Cytochrome P450 2C42 (CYP2C42)	-3.077	2.63E-03
A_72_P443884	-	-2.591	5.83E-03
A_72_P054101	-	-2.533	2.20E-03
NM_001159306	Pyruvate dehydrogenase kinase isozyme 4, mitochondrial (PDK4)	-2.380	1.59E-04
NM_213850	Glutathione S-transferase alpha 2 (GSTA2)	-2.324	2.97E-03
ENSSSCT00000015537	Cysteine Dioxygenase Type 1 (CDO1)	-2.323	1.10E-07
TC613147	-	-2.041	5.78E-03
NM_214109	Chymotrypsin-like elastase family, member 2A (CELA2A)	-1.987	1.20E-02
KP735782	Isolate S1-V1 glutathione S-transferase A2 (GSTA2)	-1.898	1.25E-03
TC534148	-	-1.869	9.17E-06
NM_001134824	Cytochrome P450 3A46 (CYP3A46)	-1.709	5.59E-03
NM_214200	Perilipin 2 (PLIN2)	-1.583	2.52E-02
ENSSSCT00000026393	Cytochrome P450 2C34 (CYP2C34)	-1.446	4.89E-02
AK232564	-	-1.397	3.45E-04
NM_213962	Hydroxyacyl-CoA dehydrogenase trifunctional multienzyme complex subunit alpha (HADHA)	-1.397	1.82E-04
NM_001008691	Chemokine (C-X-C motif) ligand 10 (CXCL10)	-1.372	1.81E-02
NM_001078665	Chemokine ligand 26-like (CCL26) (MIP-4a, eotaxin-3)	-1.367	9.37E-06
TC548575	-	-1.362	3.72E-04
NM_001038644	Angiopoietin like 4 (ANGPTL4)	-1.342	4.00E-02
NM_214422	CYP3A39	-1.281	4.27E-02
TC569278	-	-1.268	1.82E-04
AK238381	-	-1.266	3.14E-03

AK236120	CALHM2	-1.223	3.96E-03
TC590364	-	-1.185	1.24E-04
TC598771	-	-1.175	8.10E-05
TC544200	-	-1.162	3.33E-02
AK345473	-	-1.150	2.01E-03
CV871207	-	-1.142	6.46E-05
TC604056	-	-1.141	1.49E-03
NM_214185	Microsomal triglyceride transfer protein (MTTP)	-1.128	3.17E-02
TC622808	-	-1.114	4.84E-02
NM_001114056	MHC class I antigen 5 (SLA-5)	-1.098	2.38E-03
TC623278	Sex comb on midleg-like 1 (SCML1)	-1.070	3.57E-02
FD604545	-	-1.065	2.03E-03
NM_214425	CYP4A21	-1.036	2.22E-07
TC541539	-	-1.027	6.66E-03
EW262869	-	-1.017	1.65E-04
NM_001122990	NNAT	-1.016	2.95E-04
NM_001128474	Guanylate binding protein 2, interferon-inducible (GBP2)	-1.012	9.37E-06
NM_001098605	PNPLA2	-1.009	2.05E-03
ENSSSCT00000023435	TNF receptor associated factor 3 (TRAF3)	-0.997	1.02E-05
TC530663	-	-0.990	1.64E-02
NM_214246	carboxylesterase 1 (CES1)	-0.982	2.06E-02
NM_001315766	carboxylesterase	-0.972	1.46E-02
ENSSSCT00000035428	Arginase 2 (ARG2)	-0.971	3.03E-04
TC536661	-	-0.970	4.76E-05
NM_001246243	Carnitine palmitoyltransferase 2 (CPT2)	-0.967	2.15E-04
TC557164	2'-5' oligoadenylate synthetase 2 (OAS)	-0.966	3.11E-02
NM_001044599	GADD45A	-0.965	3.54E-02
BF713827	-	-0.956	1.22E-02
NM_001128469	ISG15	-0.956	4.44E-02
NM_001101027	Cytochrome P450, family 39A1 (CYP39A1)	-0.955	6.50E-06
AK232386	MX2	-0.948	3.17E-03
TC549936	-	-0.945	2.54E-02
NM_001287412	Nuclear receptor subfamily 1H4 (NR1H4)	-0.945	1.61E-03
TC610430	-	-0.932	1.13E-02
TC623045	-	-0.928	2.20E-03
AK237295	GPCPD1	-0.924	7.55E-03
ENSSSCT00000010309	Leucine Rich Repeats And Calponin Homology Domain Containing 1 (LRCH1)	-0.923	1.63E-02
CN163990	-	-0.922	7.22E-04
AK345001	MX2	-0.918	1.65E-02
TC601797	Mitochondrial Carnitine O-palmitoyltransferase 2	-0.916	7.97E-04
TC571068	-	-0.916	1.83E-04
ENSSSCT00000001838	Peroxisomal biogenesis factor 6 (PEX6)	-0.913	1.53E-02
EW034563	-	-0.900	7.55E-04
TC592596	-	-0.899	1.24E-02

TC627807	LOC106504276	-0.885	5.54E-04
NM_001143719	MAP kinase interacting serine/threonine kinase 1 (MKNK1)	-0.881	3.68E-05
A_72_P152666	GPT2 (glutamic--pyruvic transaminase 2)	-0.880	1.57E-02
TC610412	-	-0.879	2.60E-03
A_72_P344163	-	-0.871	1.68E-02
NM_001134349	Kruppel-like factor 15 (KLF15)	-0.867	2.26E-05
AK392845	RNASET2	-0.863	3.07E-05
NM_001185143	SLA-DOA	-0.859	4.87E-05
TC580089	-	-0.858	2.31E-03
A_72_P032671	CLEC2D (C type lectin domain family 2)	-0.856	4.32E-02
A_72_P705022	Acetyl-CoA acyltransferase 2 (ACAA2)	-0.853	3.96E-02
ENSSSCT00000015179	Arrestin domain containing 2 (ARRDC2)	-0.835	3.93E-04
NM_001195115	SLC25A27	-0.829	1.46E-03
AK239735	LOC100155195	-0.824	3.33E-02
ENSSSCT00000028877	Aminoacylproline aminopeptidase prolidase	-0.818	4.33E-04
TC568621	-	-0.814	1.70E-03
AK343323	-	-0.812	9.55E-03
TC576734	Inter-alpha-trypsin inhibitor heavy chain H1 precursor	-0.801	2.92E-02
TC555339	-	-0.796	6.04E-04
TC598894	-	-0.795	1.12E-02
NM_001097416	MX2	-0.794	9.00E-03
TC548865	-	-0.790	5.66E-04
AK389171	GRAMD1B	-0.789	3.67E-02
ENSSSCT00000004450	RNASET2	-0.783	5.67E-04
TC566172	-	-0.782	2.29E-02
NM_001097506	AN1-type zinc finger protein 5 (ZFAND5)	-0.781	2.26E-05
ENSSSCT00000003289	Actinin, alpha 4 (ACTN4)	-0.779	2.96E-02
ENSSSCT00000004670	Tumor protein D52-like 1 (TPD52L1)	-0.778	3.34E-04
ENSSSCT00000007615	WDR63	-0.774	2.29E-02
TC532659	-	-0.769	2.73E-02
TC560156	-	-0.766	1.88E-05
NM_001243304	Cytochrome P450, family 27 A1 (CYP27A1)	-0.765	2.60E-03
TC524151	-	-0.764	3.98E-02
CA779378	-	-0.764	3.95E-02
NM_001024696	TNFSF10	-0.757	4.54E-03
XM_013981732	GNAT1	-0.755	1.21E-03
TC595220	-	-0.754	7.55E-04
TC559094	-	-0.750	1.49E-03
ENSSSCT00000012586	Succinate-CoA ligase, GDP-forming, beta subunit	-0.750	1.60E-04
CK454137	-	-0.748	2.53E-02
TC526612	-	-0.746	3.27E-02
XR_001309264	LOC102158887	-0.741	3.68E-03
NP276891	major histocompatibility complex class I antigen	-0.741	1.25E-02
A_72_P297874	Dysbindin domain containing 1 (DBNDD1)	-0.738	5.86E-03
TC553534	-	-0.736	4.01E-03

EW200629	-	-0.735	1.22E-04
BP446680	-	-0.733	6.91E-05
ENSSSCT00000004600	Mitogen-activated protein kinase kinase kinase 5 (MEKK5)(MAP3K5)	-0.729	1.00E-02
NM_001243362	Lactamase, beta (LACTB)	-0.724	2.03E-03
ENSSSCT00000000924	Thymine DNA glycosylase	-0.715	4.29E-02
DB804246	-	-0.712	2.26E-05
NM_001078670	Interferon regulatory factor 9 (IRF9)	-0.712	2.74E-02
TC605742	Acyl-CoA-binding protein (ACBP)	-0.709	2.06E-02
ENSSSCT00000011130	Mitogen-activated protein kinase kinase kinase 21 (MLK4)	-0.709	7.65E-04
ENSSSCT00000006527	NDRG1	-0.703	2.25E-03
DN120432	-	-0.699	4.64E-03
NM_214385	prostaglandin reductase 1 (PTGR1)	-0.694	9.48E-03
NM_214119	diazepam binding inhibitor (GABA receptor modulator, acyl-CoA binding protein) (DBI)	-0.688	2.42E-02
TC529862	-	-0.681	2.20E-03
NM_001190235	RING1	-0.680	3.70E-05
AK232543	-	-0.679	2.62E-02
TC547562	Zinc finger protein 473 (ZNF473)	-0.678	9.31E-03
AK393873	-	-0.677	1.62E-02
ENSSSCT00000007254	tuftelin 1 (TUFT1)	-0.676	5.73E-03
ENSSSCT00000028241	SMAD specific E3 ubiquitin protein ligase 1	-0.674	3.07E-03
AK351081	-	-0.674	1.64E-02
TC567770		-0.672	4.87E-02
ENSSSCT00000006875	CCAAT/enhancer binding protein (C/EBP), delta (CEBPD)	-0.669	2.87E-04
TC533634	-	-0.668	3.07E-05
TC596995	-	-0.667	1.60E-02
ENSSSCT00000034119	LOC100516891	-0.667	2.56E-06
AK234441	LOC100153598	-0.663	7.30E-03
TC535434	-	-0.660	2.59E-02
ENSSSCT00000005761	KIAA0020	-0.657	2.90E-02
NM_001143722	Phosphatidylinositol transfer protein, cytoplasmic 1 (PITPNC1)	-0.654	3.00E-03
NM_214306	hydroxysteroid (17-beta) dehydrogenase 4 (HSD17B4)	-0.654	2.62E-02
CF365587	-	-0.651	4.97E-02
AK237715	SH3BP2	-0.647	1.87E-08
TC555476	-	-0.646	9.38E-04
NM_001098590	HPS6	-0.646	3.78E-02
XM_003128430	TNFRSF21	-0.643	3.32E-02
A_72_P531712	-	-0.642	5.00E-02
ENSSSCT00000019531	Acyl-CoA dehydrogenase very long chain (ACADVL)	-0.641	3.53E-02
NM_001243420	cold shock domain containing C2, RNA binding (CSDC2)	-0.640	9.44E-03
NM_213742	Calcitonin-related polypeptide beta (CALCB)	-0.639	1.17E-02
DN112306	-	-0.636	1.65E-05
TC608215	-	-0.634	3.74E-04
BX677249	-	-0.629	1.24E-02
NM_001243452	DNA-damage-inducible transcript 4 (DDIT4)	-0.628	3.54E-02

TC546244	-	-0.627	1.75E-02
TC594876	-	-0.621	2.72E-02
ENSSSCT00000029337	KLF9	-0.615	3.54E-02
NM_214323	Junction plakoglobin (JUP)	-0.605	2.72E-02
ENSSSCT00000022976	Guanine nucleotide exchange factor (GEF) 3	-0.602	1.29E-02
XM_003129835	Exophilin 5 (EXPH5)	-0.601	1.74E-03
ENSSSCT00000002574	Serine/arginine-rich splicing factor 5	-0.599	1.59E-04
ENSSSCT00000010573	ADAM-like, decysin 1 (ADAMDEC1)	-0.595	4.12E-02
NM_214154	TXNRD1	-0.593	5.59E-03
TC555936	-	-0.593	9.07E-03
TC601929	-	-0.590	5.74E-03
ENSSSCT00000017757	Ubiquitin specific peptidase 40	-0.588	1.21E-02
NM_001123217	ZNF217	-0.587	3.68E-05
TC629646	-	-0.587	1.82E-02
ENSSSCT00000025705	Poly(ADP-ribose) polymerase family member 4 (PARP4)	-0.586	1.01E-03
NM_001101028	Acyl-CoA Oxidase 1 (ACOX1)	-0.585	1.98E-02
NM_001110172	Aquaporine 3 (AQP3)	-0.580	4.41E-02
TC541583	-	-0.579	2.10E-02
TC550878	-	-0.577	4.80E-02
ENSSSCT00000007361	hydroxyacid oxidase 2 (HAO2)	-0.576	1.17E-02
TC557067	-	-0.575	4.99E-02
TC618316	-	-0.568	6.86E-03
NM_213966	Hydroxyacyl-CoA dehydrogenase (HADHB)	-0.563	2.96E-02
NM_214153	ENTPD1	-0.562	1.08E-02
ENSSSCT00000026913	Zinc finger cchc domain containing 4	-0.561	2.69E-02
TC539212	PITPNC1	-0.561	3.85E-04
TC543158	Histamine N-methyltransferase	-0.558	4.37E-02
ENSSSCT00000014828	STXBP2	-0.558	1.56E-02
BX924155	-	-0.557	8.26E-03
TC575827	-	-0.557	2.14E-02
XM_013978816	Annexin A3 (ANXA3)	-0.555	6.66E-03
NM_001097452	Complement component 8, gamma polypeptide (C8G)	-0.551	3.95E-02
TC523688	Protein CutA homolog	-0.549	4.24E-05
TC538766	-	-0.548	3.53E-02
TC564615	-	-0.539	4.52E-03
TC526256	-	-0.538	2.10E-02
NM_001199132	DHX58	-0.534	3.13E-02
AK396814	WDFY3	-0.533	2.01E-02
ENSSSCT00000029598	phosphatidylinositol glycan anchor biosynthesis, class H (PIGH)	-0.532	9.35E-03
ENSSSCT00000031997	TMEM150C	-0.532	4.43E-02
AK346233	LOC100515741	-0.531	9.11E-04
TC599915	-	-0.530	1.22E-03
NM_001190254	SAM pointed domain containing ets transcription factor (SPDEF)	-0.530	3.08E-04
ENSSSCT00000017183	interferon regulatory factor 2 (IRF2)	-0.530	2.74E-02
DY405756	-	-0.528	3.60E-02

A_72_P320493	-	-0.527	1.62E-04
BX675220	-	-0.526	5.05E-03
NM_001244412	Heme oxygenase 2 (HMOX2)	-0.524	1.13E-02
AK348520	RPL14	-0.522	4.79E-02
TC539577	-	-0.521	1.06E-02
ENSSSCT00000018493	STC2	-0.521	4.12E-03
A_72_P444563	Prostaglandin reductase 1 (PTGR1)	-0.520	1.11E-02
TC614889	-	-0.520	3.09E-02
NM_214024	PPP2R1A	-0.519	1.82E-02
TC527407	-	-0.519	2.10E-02
TC623527	-	-0.516	7.30E-05
ENSSSCT00000009138	Rho GTPase activating protein 25 (ARHGAP25)	-0.515	4.39E-04
ENSSSCT00000023179	RAS homolog family member U (RHOU)	-0.512	2.38E-02
NM_214424	CYP4A24	-0.511	2.29E-05
CK454133	-	-0.509	1.00E-02
EW261758	-	-0.506	1.65E-04
ENSSSCT00000008691	MGRN1	-0.506	4.61E-03
TC593193	-	-0.506	4.47E-03
TC533090	-	-0.505	3.35E-02
TC595575	-	-0.504	4.03E-04
AK399777	SRSF5	-0.504	1.20E-02
TC601044	-	-0.503	2.32E-03
AK237079	NFKB2	-0.501	4.11E-03
TC546925	-	-0.500	9.00E-03
**WI+CS24h-C versus WI-C** (Up-regulated genes)			
TC611155	-	3.203	6.81E-12
BF444493	-	2.186	2.86E-02
TC529750	-	2.069	1.25E-10
AK398294	Creatine kinase, M-type (CKM)	1.814	5.37E-03
NM_214069	Fucosyltransferase 2 (FUT2)	1.719	5.12E-07
NM_001130532	Par-6 family cell polarity regulator beta (PARD6B)	1.579	5.27E-05
NM_001190276	nuclear receptor subfamily 4, group A, member 2 (NR4A2)	1.531	1.36E-03
TC611786	-	1.524	2.25E-03
ENSSSCT00000019353	PIPOX	1.441	1.55E-02
ENSSSCT00000025681	CRABP1	1.413	1.17E-02
ENSSSCT00000012922	B-cell lymphoma 6 protein (BCL6)	1.362	4.43E-04
DR066068	-	1.351	1.20E-02
TC552941	-	1.333	1.41E-14
NM_213868	Ficolin (collagen/fibrinogen domain containing lectin) 2 (FCN2)	1.332	1.49E-03
ENSSSCT00000002650	Proto-oncogene c-Fos (FOS)	1.307	2.89E-04
NM_001190157	PVALB	1.306	9.78E-03
TC521842	-	1.299	9.37E-06
NM_001129949	CKM	1.271	6.35E-03
NM_001097505	BTG2	1.256	1.81E-03

NM_001185171	FAM204A	1.238	1.83E-03
TC525402	Pleckstrin homology-like domain family A member 2	1.199	1.88E-03
A_72_P443393	-	1.178	4.97E-02
TC570078	-	1.178	1.80E-06
TC543988	-	1.175	2.15E-02
TC595819	-	1.164	3.33E-02
AK389182	FAM43B	1.158	1.34E-02
NM_214105	Guanylate cyclase 2C (heat stable enterotoxin receptor) (GUCY2C)	1.152	1.24E-02
ENSSSCT00000031521	LY6/PLAUR domain containing 6 (LYPD6)	1.152	1.64E-04
TC566133	-	1.088	1.22E-04
TC575360	-	1.082	3.16E-03
TC605691	V-set domain containing T cell activation inhibitor 1 (VTCN1)	1.047	1.75E-02
AJ660252	-	1.006	6.91E-05
AK398217	SFXN4	0.977	1.86E-02
XR_001305510	LOC106508566	0.957	9.48E-03
NM_001123113	Fos proto-oncogene, AP-1 transcription factor subunit	0.948	1.15E-02
ENSSSCT00000014263	REST corepressor 2 (RCOR2)	0.939	4.94E-04
AK347361	HLX	0.938	1.34E-02
ENSSSCT00000033656	CLDN4	0.934	1.44E-03
NM_001146126	Patatin-like phospholipase domain containing 3 (PNPLA3)	0.929	1.75E-03
TC556782	-	0.913	8.03E-03
TC544389	-	0.908	4.60E-02
TC553645	-	0.887	3.44E-02
TC618296	CCAAT/enhancer binding protein (C/EBP) (CEBPA)	0.881	2.56E-06
ENSSSCT00000031509	Nestin (NES)	0.873	1.29E-03
AJ648501	-	0.872	1.00E-02
NM_001113447	phytanoyl-CoA 2-hydroxylase (PHYH)	0.863	7.52E-07
ENSSSCT00000024134	phytanoyl-CoA 2-hydroxylase (PHYH)	0.862	2.30E-05
TC523118	-	0.859	3.70E-05
NM_001039746	fatty acid binding protein 5 (FABP5)	0.841	5.72E-03
XM_001926022	pantothenate kinase 1 (PANK1)	0.838	4.12E-03
ENSSSCT00000018567	cytoplasmic FMR1 interacting protein 2	0.826	6.08E-03
TC586066	-	0.812	9.34E-06
NM_001038694	Acyl-CoA synthetase long-chain family member 4 (ACSL4)	0.810	2.87E-02
ENSSSCT00000024917	K(lysine) acetyltransferase 2B (KAT2B)	0.800	9.71E-05
TC566441	Tripartite motif containing 4 (TRIM4)	0.789	1.55E-02
TC569507	-	0.787	4.27E-03
A_72_P773975	-	0.782	3.12E-02
AK240605	LOC100512960	0.770	3.59E-03
TC537380	-	0.750	2.26E-05
TC574741	-	0.743	1.80E-06
TC546352	-	0.739	3.74E-02
ENSSSCT00000009301	striatin, calmodulin binding protein (STRN)	0.738	5.78E-03
ENSSSCT00000004523	Mitochondrial MTHFD1L	0.728	4.41E-02

ENSSSCT00000026371	ADAM metallopeptidase domain 9 (ADAM9)	0.725	9.35E-05
DY407595	-	0.725	2.25E-03
AK400200	LOC102159136	0.720	3.54E-03
EW177256	-	0.719	1.63E-05
NM_001031782	Krueppel-like factor 4 (KLF4)	0.712	4.41E-02
AK392785	KCNJ10	0.711	4.90E-02
A_72_P015946	-	0.702	1.42E-08
ENSSSCT00000000280	IGFBP6	0.701	2.85E-02
TC523104	-	0.698	2.10E-03
A_72_P322198	ZBTB44	0.696	1.86E-03
ENSSSCT00000003857	EPH receptor A2 (EPHA2)	0.694	5.25E-03
ENSSSCT00000017407	HOXD1	0.684	1.40E-02
AJ964590	-	0.679	2.91E-03
TC533770	ENSP00000381892	0.678	5.73E-03
TC569295	-	0.674	4.97E-02
AK234663	RAB3A	0.672	3.82E-02
ENSSSCT00000023491	Histone H4	0.669	6.35E-04
A_72_P218807	GABRB2	0.663	2.49E-02
TC601667	-	0.662	1.65E-02
ENSSSCT00000035633	MAP2K6	0.662	1.26E-02
TC588425	-	0.661	9.37E-06
NM_001044567	Trans-acting T-cell-specific transcription factor GATA3	0.659	4.67E-02
TC518112	Toll-like receptor 4 (TLR4)	0.649	3.01E-02
TC557923	-	0.648	1.79E-02
BX921275	-	0.648	7.06E-03
TC548138	-	0.647	3.84E-02
TC544492	-	0.644	1.34E-03
TC600965	-	0.643	1.61E-02
NM_214067	CAST	0.641	2.54E-03
ENSSSCT00000010315	ZC3H13	0.641	2.20E-03
TC555671	-	0.639	1.79E-02
NM_001161637	Claudin 4 (CLDN4)	0.636	3.42E-02
ENSSSCT00000009614	Replication factor c subunit 1 activator 1	0.634	8.86E-05
TC597684	-	0.624	1.00E-02
TC617432	-	0.620	3.20E-03
XM_001929359	ATP binding cassette subfamily C member 2 (ABCC2)	0.617	4.10E-04
DY417616	-	0.613	3.68E-05
NM_001246253	EIF4G1	0.613	3.80E-03
ENSSSCT00000015660	Early growth response protein 1 (EGR1)	0.612	1.29E-03
ENSSSCT00000018581	GEMIN5	0.609	2.51E-03
ENSSSCT00000015920	NRIP3	0.607	1.69E-02
ENSSSCT00000019021	IGFBP4	0.606	1.88E-02
ENSSSCT00000011079	Histone cell cycle regulator (HIRA)	0.603	2.20E-03
NM_001037965	Inhibitor of DNA binding 2 (ID2)	0.603	1.68E-02
EW461055	-	0.603	2.29E-02

NM_001243407	Thioredoxin-related transmembrane protein 4 (TMX4)	0.601	3.02E-03
ENSSSCT00000030542	Ubiquitin carboxyl terminal hydrolase 10	0.599	9.13E-03
ENSSSCT00000029240	KAT8 regulatory NSL complex subunit 1-like	0.594	4.03E-04
TC540065	Serine/threonine-protein kinase Kist	0.593	4.82E-02
AK389851	-	0.590	5.69E-04
AK400564	-	0.588	4.67E-02
ENSSSCT00000002909	Transmembrane protein 14A (TMEM14A)	0.585	3.41E-02
TC622072	-	0.582	4.19E-02
BX920940	-	0.580	5.82E-03
TC525538	Dual specificity tyrosine phosphorylation regulated kinase 2 (DYRK2)	0.580	1.22E-04
ENSSSCT00000009295	CCAAT/enhancer binding protein (C/EBP), zeta (CEBPZ)	0.578	3.96E-02
AK351387	-	0.578	9.24E-05
AK347880	-	0.568	4.09E-02
TC625344	ZNF212	0.567	9.11E-04
ENSSSCT00000004439	DACT2	0.566	3.67E-02
AJ954855	-	0.564	9.13E-03
TC555161	-	0.563	1.75E-03
TC621005	-	0.561	1.52E-02
NM_213753	TSPO	0.560	1.01E-02
NM_213919	Aurora kinase B (AURKB)	0.558	2.37E-02
NM_001109944	PEG10	0.558	1.83E-02
NM_001243807	FKBP10	0.558	2.66E-02
A_72_P443589	Histone H4	0.557	2.44E-03
ENSSSCT00000005197	Microtubule associated 1A MAP 1A	0.555	1.44E-02
AW360167	-	0.554	4.84E-02
ENSSSCT00000032295	GSK3B interacting protein	0.542	2.45E-03
A_72_P144706	-	0.541	1.29E-02
TC518652	-	0.538	2.22E-04
CA778604	-	0.538	2.20E-03
TC572525	-	0.538	2.30E-05
ENSSSCT00000011691	SEC23 interacting protein	0.536	4.96E-04
ENSSSCT00000010279	Alpha-1,2-mannosyltransferase (ALG11)	0.536	9.89E-05
ENSSSCT00000008564	general transcription factor IIIC (GTF3C1)	0.533	8.07E-04
ENSSSCT00000005108	LEO1	0.533	7.25E-03
NM_214352	ATPase. Ca++ transporting (ATP2B1)	0.533	1.03E-02
ENSSSCT00000036684	ENTPD1	0.532	1.03E-02
ENSSSCT00000004227	USP1	0.532	1.59E-03
TC586031	LOC522928 protein	0.532	2.70E-02
ENSSSCT00000031203	FAM89A	0.528	7.51E-03
ENSSSCT00000011497	TLL2	0.528	1.95E-02
ENSSSCT00000026997	Histone H4 (HIST1H4A)	0.526	3.57E-02
TC589536	-	0.526	2.32E-02
TC595432	-	0.524	8.03E-03
DN123882	-	0.524	2.52E-03
ENSSSCT00000025080	E3 Ubiquitin ligase dtx2	0.524	4.19E-02

ENSSSCT00000034652	Immediate Early Response 3 (IER3)	0.524	2.04E-02
A_72_P125346	SAP30-like	0.522	8.69E-03
BX669300	-	0.521	8.14E-04
TC567103	-	0.514	1.00E-02
ENSSSCT00000026137	Histone cluster 1, H2bo	0.512	3.48E-03
ENSSSCT00000024456	DOK4	0.512	4.77E-02
TC590749	-	0.510	9.82E-03
ENSSSCT00000030173	Cordon-bleu protein-like 1	0.509	2.53E-02
TC559320	-	0.509	1.52E-02
TC565811	-	0.508	2.91E-02
TC591601	Pleckstrin homology domain-containing family A2-like	0.506	1.36E-03
TC532904	-	0.506	8.68E-04
BX916072	-	0.505	4.06E-03
BX916806	-	0.504	3.63E-05
TC594690	-	0.503	2.88E-02
NM_001113446	CHORDC1	0.503	4.48E-03
AK233798	-	0.501	3.81E-04
BI402663	-	0.501	1.50E-02

Table S2: List of differentially expressed genes in corticomedullary junction (CMJ) tissues extracted from heatmaps.

The table provided represents the down-regulated and up-regulated differentially expressed genes in compared CMJ conditions, with a corrected p value < 5% and a log2 fold change > 0.5. The annotation was obtained from Feature Extraction software.

**Systematic Name**	**Gene Name or predictive (by blast notably) Gene Name**	**Log 2 Fold change**	**p value (correction BH)**
**WI-CMJ versus Ctl-CMJ** (Down-regulated genes)			
A_72_P231427	Cell division control protein 42 (CDC42) effector protein 1 (CDC42EP1)	-3,342	4,25E-09
TC602959	-	-2,290	3,28E-12
A_72_P001141	-	-1,991	1,83E-12
FD592765	-	-1,729	2,42E-03
TC625961	-	-1,611	4,13E-08
TC562631	-	-1,561	7,37E-08
TC584676	-	-1,481	4,29E-05
TC538121	-	-1,225	5,06E-09
NM_001001539	AKR1B1	-1,082	2,90E-02
TC631544	-	-1,037	2,36E-03
DN134227	-	-1,023	2,00E-02
TC521714	-	-0,957	3,03E-02
DN102262	-	-0,930	4,88E-03
ENSSSCT00000034652	Immediate Early Response 3 (IER3)	-0,858	3,03E-04
ENSSSCT00000034425	Mitogen-Activated Protein Kinase Kinase Kinase 8 (MAP3K8)	-0,831	5,92E-04
AJ961685	-	-0,809	5,25E-06
TC526535	-	-0,752	3,45E-02
CO992076	Endometrium gilt D6 of estrous cycle	-0,737	4,25E-09
TC535436	-	-0,685	3,43E-02
DN132923	-	-0,676	3,15E-02
TC539177	-	-0,659	7,27E-05
A_72_P404593	TXNIP (Thioredoxin-interacting protein)	-0,651	7,55E-04
AJ959301	-	-0,637	3,45E-02
TC531633	-	-0,634	7,79E-03
TC575827	-	-0,630	2,27E-02
AK394979	LOC100156195	-0,626	1,87E-02
EW642054	-	-0,622	7,55E-04

TC563627	-	-0,615	2,12E-02
TC607613	-	-0,612	1,96E-02
DN104045	-	-0,611	3,33E-03
TC553365	LOC100625598	-0,607	8,18E-03
AK398427	LOC100739725	-0,600	1,87E-02
ENSSSCT00000028177	CFAP126	-0,589	3,23E-02
TC576066	-	-0,586	4,56E-02
TC613549	-	-0,585	4,04E-06
A_72_P431054	Mannan-binding lectin serine peptidase 2 (MASP2)	-0,560	1,87E-02
TC601571	-	-0,547	4,63E-02
TC582618	-	-0,545	1,76E-07
TC525370	-	-0,535	6,55E-03
AK350920	-	-0,532	2,78E-02
**WI-CMJ versus Ctl-CMJ** (Up- regulated genes)			
NM_001123113	Fos proto-oncogene, AP-1 transcription factor subunit	2,621	1,55E-09
ENSSSCT00000002650	Proto-oncogene c-Fos (FOS)	2,375	5,06E-09
ENSSSCT00000019139	Collagen type I (COL1A1)	1,734	3,82E-03
JX092267	Adiponectin, C1Q And Collagen Domain Containing (ADIPOQ)	1,650	1,30E-02
EF601160	Adiponectin, C1Q And Collagen Domain Containing (ADIPOQ)	1,441	1,14E-02
ENSSSCT00000036290	Transcription factor AP-1 (JUN)	1,429	7,56E-05
DR066068	-	1,423	2,27E-02
JN899104	Small nucleolar RNA, C/D box 3 cluster (pSNORD3)	1,378	4,46E-03
NM_213878	CNN1	1,349	3,07E-02
NM_001097505	BTG2	1,298	4,70E-03
TC564655	-	1,297	1,30E-03
AK343913	AMP010080F05 macrophage	1,288	4,74E-05
EV864657	COL8A1	1,233	8,24E-03
TC587527		1,173	2,07E-02
ENSSSCT00000015660	Early growth response protein 1 (EGR1)	1,109	8,21E-08
AK351795	CD83	1,088	9,24E-05
AK391674	-	1,061	7,53E-03
NM_001130243	troponin C type 1 (TNNC1)	1,058	3,07E-02
ENSSSCT00000007604	Cysteine-rich angiogenic inducer 61 (CYR61)	0,973	4,38E-02
TC595529	MGC148805 protein	0,972	2,88E-02
NM_214055	IL1B1	0,944	2,12E-02
NM_213880	Transcription factor AP-1 (JUN)	0,936	2,88E-02
XM_003130083	ADAMTS8	0,924	1,26E-02
ENSSSCT00000003289	Actinin, alpha 4 (ACTN4)	0,892	2,88E-02
A_72_P376423	-	0,885	3,62E-02
NM_001123189	RAS homolog family member B (RHOB)	0,868	5,74E-05
NM_001031782	Krueppel-like factor 4 (KLF4)	0,866	2,88E-02
NM_001164652	Aggrecan (ACAN)	0,846	3,07E-02
ENSSSCT00000017924	ZYX	0,831	1,24E-02
ENSSSCT00000025250	HSPB6	0,812	3,07E-02

ENSSSCT00000035115	Biglycan (BGN)	0,796	8,16E-03
ENSSSCT00000004344	Serine/threonine-protein kinase (PLK3)	0,795	9,91E-03
ENSSSCT00000006766	Peptidase inhibitor 15 (PI15)	0,794	3,99E-02
TC585714	Bladder cancer-associated protein	0,775	2,90E-02
NM_001160426	ARF3	0,766	1,87E-02
NM_001009579	CCL3L1	0,758	2,90E-02
ENSSSCT00000023788	COL6A2	0,737	1,65E-03
NM_214323	Junction plakoglobin (JUP)	0,734	1,87E-02
NM_001122984	MFGE8	0,733	1,87E-02
ENSSSCT00000036039	PDK2	0,715	2,12E-02
AK240364	LOC733585	0,709	3,03E-02
ENSSSCT00000032777	Guanine nucleotide exchange factor 3 (VAV3)	0,693	4,55E-03
ENSSSCT00000015281	GUK1	0,689	3,07E-02
ENSSSCT00000033515	CD83	0,685	1,20E-03
NM_001001535	DES	0,682	4,91E-02
TC626843	-	0,664	4,38E-02
ENSSSCT00000008691	MGRN1	0,653	8,81E-04
NM_001244095	ACKR1	0,648	2,52E-02
NM_214190	THRA	0,634	2,12E-02
A_72_P442293	-	0,630	2,27E-02
NP276701	-	0,630	1,60E-02
ENSSSCT00000018236	AEBP1	0,614	4,19E-02
TC546273	-	0,611	4,91E-02
NM_001123104	Cathepsin Z (CTSZ)	0,611	3,03E-02
NM_001243527	PI16	0,602	2,28E-02
ENSSSCT00000006411	EDF1	0,594	4,13E-02
NM_001243420	cold shock domain containing C2, RNA binding (CSDC2)	0,588	4,08E-02
NM_214024	PPP2R1A	0,580	2,21E-02
ENSSSCT00000011578	Actin-related protein 1 homolog A (ACTR1A)	0,558	2,27E-02
ENSSSCT00000002721	RIN3	0,550	4,18E-02
NM_001123165	PPP1R10	0,548	1,26E-02
DN118139	RHOG	0,543	2,88E-02
BX914343	-	0,537	2,52E-02
NM_001033008	AGPAT1	0,533	1,37E-02
ENSSSCT00000035130	Transforming growth factor. beta receptor II	0,530	1,14E-02
ENSSSCT00000026670	TAX1	0,525	3,20E-02
NM_001037721	CTSD	0,524	1,88E-02
NM_001173519	ORAI1	0,516	3,60E-02
CX064162	-	0,516	3,35E-02
ENSSSCT00000035744	Forkhead box O1	0,513	3,29E-02
ENSSSCT00000018167	PRR15	0,507	8,24E-03
NM_213986	MYOC	0,503	3,28E-02
ENSSSCT00000010906	KIAA1671	0,501	3,16E-02
**WI+CS6h-CMJ versus Ctl-CMJ** (Down-regulated genes)			

A_72_P231427	Cell division control protein 42 effector protein 1 (CDC42EP1)	-3,516	6,33E-10
A_72_P443884	-	-2,791	2,23E-03
NM_001167835	Cytochrome P450 2C42 (CYP2C42)	-2,772	6,39E-03
ENSSSCT00000015537	Cysteine Dioxygenase Type 1 (CDO1)	-2,747	7,12E-10
NM_213850	Glutathione S-transferase alpha 2 (GSTA2)	-2,559	7,67E-04
TC602959	-	-2,403	5,54E-13
NM_001159306	Pyruvate dehydrogenase kinase isozyme 4, mitochondrial (PDK4)	-2,356	1,30E-04
A_72_P054101	-	-2,334	4,22E-03
TC534148	-	-2,262	8,16E-08
TC613147	-	-2,251	1,69E-03
NM_214109	Chymotrypsin-like elastase family, member 2A (CELA2A)	-2,251	3,22E-03
A_72_P001141	-	-1,998	5,54E-13
FD592765	-	-1,916	1,19E-04
KP735782	Isolate S1-V1 glutathione S-transferase A2 (GSTA2)	-1,767	2,13E-03
NM_001078665	Chemokine ligand 26-like (CCL26) (MIP-4a, eotaxin-3)	-1,615	1,67E-07
TC584676	-	-1,508	6,93E-06
NM_001134824	CYP3A46	-1,470	1,75E-02
ENSSSCT00000002555	Arginase 2 (ARG2)	-1,443	3,84E-02
TC625961	-	-1,420	2,77E-07
TC541539	-	-1,408	1,24E-04
TC631544	-	-1,364	6,50E-06
NM_001001539	AKR1B1	-1,359	8,77E-04
DN134227	-	-1,316	3,07E-04
TC562631	-	-1,255	2,96E-06
AK238136	C4BPA	-1,254	1,85E-02
TC598771	-	-1,249	1,81E-05
ENSSSCT00000004065	Facilitator superfamily domain containing 2A (MFSD2A)	-1,244	4,71E-02
AK238381	-	-1,185	5,15E-03
AK236120	CALHM2	-1,184	4,55E-03
AK345473	-	-1,173	1,23E-03
TC538121	-	-1,145	1,57E-08
EW262869	-	-1,139	1,97E-05
NM_001128469	ISG15	-1,133	1,13E-02
TC590364	-	-1,129	1,81E-04
TC548575	-	-1,111	3,22E-03
TC569278	-	-1,109	7,67E-04
TC544200	-	-1,099	4,42E-02
CV871207	-	-1,097	8,02E-05
ENSSSCT00000035428	Arginase 2 (ARG2)	-1,091	3,78E-05
NM_001101027	Cytochrome P450. family 39A1 (CYP39A1)	-1,079	2,01E-07
NM_001163696	C13H21orf62 (chromosome 13 open reading frame)	-1,069	3,95E-03
TC571068	-	-1,067	1,13E-05
TC623278	Sex comb on midleg-like 1 (SCML1)	-1,036	4,11E-02

TC604056	-	-1,023	3,85E-03
TC576734	Inter-alpha-trypsin inhibitor heavy chain H1 precursor	-1,020	3,18E-03
TC548865	-	-1,016	8,86E-06
NM_001287412	Nuclear receptor subfamily 1H4 (NR1H4)	-1,009	5,41E-04
AK232564	-	-1,004	1,02E-02
TC596995	-	-0,996	1,67E-04
ENSSSCT00000001838	Peroxisomal biogenesis factor 6 (PEX6)	-0,992	6,57E-03
TC560156	-	-0,987	7,78E-08
TC566172	-	-0,973	2,80E-03
NM_001114056	MHC class I antigen 5 (SLA-5)	-0,972	6,78E-03
ENSSSCT00000004670	Tumor protein D52-like 1 (TPD52L1)	-0,971	7,13E-06
CN163990	-	-0,951	3,68E-04
NM_213962	Hydroxyacyl-CoA dehydrogenase trifunctional multienzyme complex subunit alpha (HADHA)	-0,949	1,10E-02
NM_001122990	NNAT	-0,946	5,29E-04
FD604545	-	-0,944	5,76E-03
AK345001	MX2	-0,939	1,21E-02
TC568621	-	-0,933	2,36E-04
DN102262	-	-0,929	9,91E-04
EW034563	-	-0,925	4,04E-04
TC526612	-	-0,924	4,89E-03
NM_001044573	Angiogenin, ribonuclease, RNase A family, 5 (ANG)	-0,922	2,17E-02
TC531633	LOC107521672	-0,917	8,14E-06
TC559094	-	-0,913	8,81E-05
NM_001243452	DNA-damage-inducible transcript 4 (DDIT4)	-0,910	9,80E-04
A_72_P273929	Zinc finger protein 12	-0,904	4,02E-02
NM_001097501	LGALS3	-0,897	2,51E-05
NM_001098590	HPS6	-0,897	1,83E-03
ENSSSCT00000024997	S100A2 (S100 calcium binding a2)	-0,890	1,46E-02
NM_001128474	Guanylate binding protein 2, interferon-inducible (GBP2)	-0,884	4,22E-05
TC536661	-	-0,883	1,24E-04
EW261758	-	-0,880	3,64E-09
TC543804	-	-0,873	7,64E-05
TC598894	-	-0,872	4,09E-03
ENSSSCT00000007615	WDR63	-0,870	7,72E-03
NM_001097452	C8G	-0,860	4,74E-04
NM_001044557	S100A6	-0,858	4,27E-02
TC532659	-	-0,855	1,04E-02
XR_001309264	LOC102158887	-0,847	6,39E-04
A_72_P404593	TXNIP (Thioredoxin-interacting protein)	-0,846	2,12E-06
ENSSSCT00000009671	RASL11B	-0,831	3,73E-03
ENSSSCT00000004577	CITED2	-0,830	1,83E-06
NM_001315766	Carboxylesterase	-0,827	4,32E-02
ENSSSCT00000034425	Mitogen-Activated Protein Kinase Kinase Kinase 8 (MAP3K8)	-0,824	1,30E-04
ENSSSCT00000033355	S100A4	-0,823	6,77E-03

TC532382	-	-0,822	2,36E-02
NM_001097506	AN1-type zinc finger protein 5 (ZFAND5)	-0,818	5,56E-06
NM_001195115	SLC25A27	-0,817	1,32E-03
TC547562	Zinc finger protein 473 (ZNF473)	-0,816	1,11E-03
ENSSSCT00000034119	Family with sequence similarity 122C	-0,815	1,71E-08
TC601797	Mitochondrial Carnitine O-palmitoyltransferase 2	-0,814	2,42E-03
BX675220	-	-0,814	1,08E-05
XM_013981732	GNAT1	-0,811	3,67E-04
ENSSSCT00000028953	Zinc finger protein 114	-0,809	2,96E-02
TC610412	-	-0,805	5,40E-03
NM_001097416	MX2	-0,805	6,73E-03
TC621729	-	-0,803	3,79E-03
TC535436	-	-0,798	3,12E-03
ENSSSCT00000002574	Serine/arginine-rich splicing factor 5	-0,779	1,54E-06
CO992076	Endometrium gilt D6 of estrous cycle	-0,777	6,33E-10
A_72_P399733	-	-0,772	3,40E-05
TC567905	-	-0,769	6,31E-03
TC562329	-	-0,764	1,07E-04
NM_001134349	Kruppel-like factor 15 (KLF15)	-0,763	9,47E-05
TC555339	-	-0,763	7,67E-04
AK346233	LOC100515741	-0,760	2,96E-06
AK399810	S100A4	-0,757	1,81E-02
AK237715	SH3BP2	-0,757	1,65E-10
AK232386	MX2	-0,752	2,19E-02
ENSSSCT00000012586	Succinate-CoA ligase. GDP-forming. beta subunit	-0,751	1,13E-04
NM_001185143	SLA-DOA	-0,750	2,36E-04
TC592596	-	-0,750	4,32E-02
TC595575	-	-0,749	4,03E-07
AK393873	-	-0,745	6,31E-03
TC575827	-	-0,742	1,15E-03
NM_001145222	RBP7	-0,741	4,27E-03
TC548784	-	-0,738	7,27E-03
CA779378	-	-0,733	4,75E-02
NM_213742	Calcitonin-related polypeptide beta (CALCB)	-0,732	2,71E-03
TC576690	-	-0,730	3,81E-02
TC533634	-	-0,722	4,68E-06
TC566289	-	-0,718	2,88E-02
TC543158	Histamine N-methyltransferase	-0,715	5,60E-03
NM_213787	SERPINA3-2	-0,715	2,76E-02
BX914375	-	-0,714	4,28E-03
TC602146	-	-0,707	4,91E-02
EW221235	-	-0,701	1,74E-03
TC599915	-	-0,697	1,71E-05
ENSSSCT00000006875	CCAAT/enhancer binding protein (C/EBP), delta (CEBPD)	-0,695	1,19E-04
ENSSSCT00000027137	Neuromedin-U-like	-0,688	9,46E-08

NM_001024696	TNFSF10	-0,688	9,68E-03
TC560803	-	-0,687	4,57E-04
A_72_P431054	MASP2	-0,687	4,75E-04
ENSSSCT00000009138	Rho GTPase activating protein 25 (ARHGAP25)	-0,685	4,07E-06
DN132923	-	-0,684	1,03E-02
TC574870	-	-0,684	2,51E-03
AK236259	TRMT6	-0,683	3,24E-02
ENSSSCT00000010653	carbonyl reductase 4 (CBR4)	-0,680	3,64E-03
AK347470	LOC100624597	-0,678	8,44E-03
NM_001245012	Inositol polyphosphate-1-phosphatase (INPP1)	-0,674	7,03E-04
CK454137	-	-0,672	4,74E-02
NM_001143719	MAP kinase interacting serine/threonine kinase 1 (MKNK1)	-0,669	1,08E-03
A_72_P206912	-	-0,667	8,66E-03
TC627807	LOC106504276	-0,664	9,90E-03
TC529859	-	-0,663	1,19E-02
EW200629	-	-0,661	3,78E-04
TC582968	-	-0,655	1,94E-02
TC590063	-	-0,649	7,38E-03
NM_001123217	ZNF217	-0,648	4,10E-06
TC531848	-	-0,647	1,35E-03
TC531472	LOC108300678	-0,647	1,10E-02
TC602526	-	-0,643	1,09E-02
XM_003129835	Exophilin 5 (EXPH5)	-0,641	5,85E-04
TC623045	-	-0,638	4,55E-02
TC587447	-	-0,638	1,38E-02
AK399777	SRSF5	-0,637	8,96E-04
TC533090	-	-0,635	4,35E-03
DN104045	-	-0,634	3,80E-04
ENSSSCT00000029598	phosphatidylinositol glycan anchor biosynthesis, class H (PIGH)	-0,634	1,28E-03
DB804246	-	-0,633	8,51E-05
TC527857	-	-0,633	2,98E-02
BP446680	-	-0,632	3,76E-04
DN120432	-	-0,631	1,02E-02
ENSSSCT00000009246	PRKCE	-0,630	1,29E-02
TC614889	-	-0,630	5,60E-03
TC575521	-	-0,627	1,14E-02
NM_001136511	HUS1	-0,624	4,96E-03
TC532111	-	-0,624	1,12E-02
A_72_P353363	-	-0,624	7,73E-07
ENSSSCT00000023435	TNF receptor associated factor 3 (TRAF3)	-0,624	3,64E-03
ENSSSCT00000031997	TMEM150C	-0,622	1,27E-02
CK462660	-	-0,622	7,86E-03
AK346234	LOC100520861	-0,620	3,25E-02
NM_001111258	IFNGR2	-0,616	1,10E-04
AK230805	ZSCAN21	-0,616	1,29E-02

AK392845	RNASET2	-0,615	1,94E-03
A_72_P297874	Dysbindin domain containing 1 (DBNDD1)	-0,614	2,45E-02
TC580089	-	-0,613	3,71E-02
NM_001123200	TES	-0,611	1,91E-03
ENSSSCT00000003166	G patch domain containing 1	-0,608	2,80E-02
NM_214391	FCGR3B	-0,607	4,77E-02
NP276891	Major histocompatibility complex class I antigen	-0,606	4,86E-02
NM_214228	prostaglandin D2 synthase (PTGDS)	-0,606	1,11E-02
TC545140	-	-0,602	1,69E-03
AK351081	-	-0,602	3,45E-02
ENSSSCT00000010582	KCTD9	-0,600	7,13E-06
TC571472	-	-0,596	4,16E-02
TC585103	MGLL protein	-0,596	3,31E-02
AK345509	CLEC1A	-0,595	1,02E-02
ENSSSCT00000023179	RAS homolog family member U (RHOU)	-0,595	5,86E-03
ENSSSCT00000018493	STC2	-0,593	7,58E-04
TC584539	-	-0,593	2,47E-02
TC606037	-	-0,584	6,04E-03
TC527407	-	-0,582	7,19E-03
AK350920	-	-0,581	3,64E-03
AW416039	-	-0,581	4,50E-02
ENSSSCT00000013085	Fibronectin type III domain containing 1 precursor	-0,579	1,67E-04
NM_001243362	Lactamase, beta (LACTB)	-0,579	1,40E-02
EW030444	-	-0,578	1,12E-02
AJ957942	-	-0,570	6,33E-03
NM_213851	Phosphoinositide-3-kinase, regulatory subunit 5 (PIK3R5)	-0,570	1,87E-02
TC519529	-	-0,570	1,37E-03
TC532501	-	-0,569	1,38E-02
AK352378	VPS51	-0,568	3,71E-02
AK236308	-	-0,566	2,96E-06
ENSSSCT00000015179	Arrestin domain containing 2 (ARRDC2)	-0,565	1,85E-02
NM_001190184	DnaJ (Hsp40) homolog, subfamily C, member 3 (DNAJC3)	-0,564	1,26E-02
A_72_P129976	-	-0,562	7,84E-03
TC538766	-	-0,561	2,76E-02
TC528961	MGC159919 protein	-0,561	3,90E-02
TC559375	-	-0,561	1,22E-07
TC528683	-	-0,560	9,95E-05
TC581829	-	-0,560	2,12E-06
TC538670	-	-0,559	3,06E-02
CK454133	-	-0,558	3,64E-03
TC555476	-	-0,555	4,11E-03
ENSSSCT00000010983	PIK3IP1	-0,553	4,76E-02
AK234441	LOC100153598	-0,553	2,81E-02
TC592842	-	-0,553	2,61E-02
TC546244	-	-0,552	4,05E-02

NM_214399	IL6	-0,551	5,60E-06
NM_001243898	SMU1	-0,550	3,65E-02
AK391669	CITED2	-0,550	1,42E-02
BI344446	-	-0,549	1,48E-03
NM_001243304	Cytochrome P450. family 27 A 1 (CYP27A1)	-0,548	3,90E-02
NM_001048187	DLK1	-0,547	2,79E-03
TC608215	-	-0,546	1,74E-03
ENSSSCT00000012379	KIAA1143	-0,546	3,53E-05
DN125588	PLN	-0,544	3,44E-02
AK345540	-	-0,544	1,35E-02
A_72_P429354	Proteasomal ATPase associated factor 1 (PAAF1)	-0,544	1,97E-04
BX667161	-	-0,543	4,72E-02
ENSSSCT00000007254	tuftelin 1 (TUFT1)	-0,541	3,12E-02
TC563627	-	-0,541	1,83E-02
NM_214425	CYP4A21	-0,540	2,36E-03
AK394979	LOC100156195	-0,540	1,76E-02
AK233048	LVRM10074A01	-0,539	1,81E-02
TC613549	-	-0,538	5,92E-06
TC565249	-	-0,538	4,31E-04
ENSSSCT00000025705	Poly(ADP-ribose) polymerase family member 4 (PARP4)	-0,537	2,15E-03
ENSSSCT00000011130	Mitogen-activated protein kinase kinase kinase 21 (MLK4)	-0,536	1,11E-02
ENSSSCT00000006821	Tocopherol (alpha) transfer protein	-0,536	1,31E-02
A_72_P118726	-	-0,535	1,22E-02
TC576582	-	-0,535	2,63E-02
ENSSSCT00000000994	SOCS2	-0,534	3,06E-02
TC618316	-	-0,534	1,02E-02
TC576666	MGC179351 protein	-0,533	3,35E-03
AK232808	LVRM10030B02	-0,530	4,73E-03
ENSSSCT00000009350	ZNF512	-0,527	1,26E-02
TC609759	-	-0,523	1,22E-05
EW486229	-	-0,522	4,75E-04
AK237396	SIK2	-0,522	8,81E-05
AK346354	C-1-tetrahydrofolate synthase, cytoplasmic (MTHFD1)	-0,522	1,28E-03
A_72_P190671	CCDC93 (coiled-coil domain containing 93)	-0,518	1,76E-03
TC522883	-	-0,518	2,35E-03
AK233683	C8G	-0,517	6,21E-04
BX677249	-	-0,517	4,71E-02
TC588408	-	-0,516	1,29E-02
TC576331	-	-0,516	3,35E-04
TC582116	-	-0,516	1,11E-02
XM_013978816	Annexin A3 (ANXA3)	-0,515	1,09E-02
TC556474	-	-0,514	4,00E-03
BX924742	-	-0,513	1,69E-03
A_72_P103106	-	-0,513	2,80E-02
AK394207	LVRM10091F11	-0,512	1,02E-02

TC589233	-	-0,511	4,06E-06
TC552040	-	-0,510	1,38E-03
NM_001244363	IFIT1	-0,510	4,77E-02
TC539892	LOC506521 protein	-0,507	4,41E-02
TC547188	-	-0,503	1,13E-05
TC583980	-	-0,502	1,93E-04
**WI+CS6h-CMJ versus Ctl-CMJ** (Up-regulated genes)			
ENSSSCT00000002650	Proto-oncogene c-Fos (FOS)	2,873	3,86E-11
TC611155	-	2,854	6,41E-11
NM_001123113	Fos proto-oncogene, AP-1 transcription factor subunit	2,750	1,65E-10
TC529750	-	2,373	1,76E-12
BF444493	-	2,272	1,92E-02
TC611786	-	2,043	3,33E-05
NM_001190276	nuclear receptor subfamily 4, group A, member 2 (NR4A2)	1,965	3,14E-05
NM_001097505	BTG2	1,777	8,86E-06
DR066068	-	1,769	5,66E-04
TC521842	-	1,577	8,93E-08
NM_214069	Fucosyltransferase 2 (FUT2)	1,523	1,83E-06
JN899104	Small nucleolar RNA, C/D box 3 cluster (pSNORD3)	1,511	2,65E-04
AK234282	-	1,503	3,67E-03
TC552941	-	1,456	8,87E-16
NM_001130532	Par-6 family cell polarity regulator beta (PARD6B)	1,404	1,93E-04
TC575360	-	1,399	1,02E-04
TC570078	-	1,380	2,47E-08
NM_213868	Ficolin (collagen/fibrinogen domain containing lectin) 2 (FCN2)	1,375	7,67E-04
TC557333	-	1,345	3,00E-02
ENSSSCT00000019353	PIPOX	1,284	3,31E-02
AK347361	HLX	1,265	4,74E-04
NM_214105	Guanylate cyclase 2C (heat stable enterotoxin receptor) (GUCY2C)	1,260	4,74E-03
TC564655	-	1,245	4,34E-04
ENSSSCT00000012922	B-cell lymphoma 6 protein (BCL6)	1,244	1,08E-03
TC554916	-	1,226	4,59E-02
TC566133	-	1,211	1,45E-05
TC561142	-	1,187	6,77E-03
TC577359	-	1,181	3,86E-03
TC593464	-	1,178	6,81E-03
TC605691	V-set domain containing T cell activation inhibitor 1 (VTCN1)	1,146	7,28E-03
ENSSSCT00000015660	Early growth response protein 1 (EGR1)	1,124	3,42E-08
AK389182	FAM43B	1,115	1,59E-02
TC543988	-	1,111	3,00E-02
ENSSSCT00000031521	LY6/PLAUR domain containing 6 (LYPD6)	1,105	2,14E-04
CN161830	-	1,099	3,31E-02
TC595819	-	1,095	4,56E-02
NM_214277	ANPEP	1,095	1,15E-02

NM_214055	IL1B1	1,085	1,35E-03
ENSSSCT00000036290	Transcription factor AP-1 (JUN)	1,084	9,87E-04
XR_001305510	LOC106508566	1,081	2,36E-03
AK395517	-	1,064	3,25E-02
NM_001113447	phytanoyl-CoA 2-hydroxylase (PHYH)	1,036	5,02E-09
TC546352	-	1,022	1,87E-03
TC525402	Pleckstrin homology-like domain family A member 2	1,017	8,14E-03
TC556782	-	0,998	2,74E-03
TC568589	-	0,997	4,77E-02
NM_214214	C-C motif chemokine 2 (CCL2)	0,990	2,40E-03
ENSSSCT00000024134	phytanoyl-CoA 2-hydroxylase (PHYH)	0,982	1,52E-06
ENSSSCT00000004344	Serine/threonine-protein kinase (PLK3)	0,975	1,67E-04
ENSSSCT00000010370	DIS3 homolog, exosome endoribonuclease and 3'-5' exoribonuclease	0,965	2,16E-02
XM_001929359	ATP binding cassette subfamily C member 2 (ABCC2)	0,957	1,67E-07
ENSSSCT00000018946	Vacuolar proton translocating ATPASE A1	0,957	9,33E-03
NM_213880	Transcription factor AP-1 (JUN)	0,948	8,32E-03
AK237533	SPL010057H01	0,947	1,90E-02
ENSSSCT00000022578	Mucolipin 3 (MCOLN3)	0,939	9,72E-03
AJ660252	-	0,894	2,51E-04
NM_001004027	Heme oxygenase 1 (HMOX1)	0,885	2,92E-02
TC618296	CCAAT/enhancer binding protein (C/EBP) (CEBPA)	0,865	1,58E-06
NM_001001861	CXCL2	0,864	2,63E-02
DY407595	-	0,861	2,08E-04
NM_001044600	RGS2	0,858	1,48E-02
TC546915	-	0,853	2,46E-02
ENSSSCT00000027822	HSPA12A	0,853	4,59E-02
ENSSSCT00000001267	Histone H2A type 1-like (HIST1H2AB)	0,852	1,72E-03
AK351795	CD83	0,850	7,76E-04
ENSSSCT00000028312	SLC7A8	0,849	2,37E-02
ENSSSCT00000011534	ATP binding cassette subfamily C member 2 (ABCC2)	0,845	3,66E-04
TC574741	-	0,839	5,33E-08
A_72_P139871	GPD1L (glycerol-3-phosphate dehydrogenase 1-like)	0,834	3,26E-02
AK351387	-	0,832	7,78E-08
TC537380	-	0,830	2,10E-06
A_72_P773975	-	0,803	2,37E-02
NM_214455	SLC22A8	0,798	1,16E-02
TC533770	ENSP00000381892	0,797	7,58E-04
BI339629	-	0,791	3,67E-02
TC525987	-	0,783	2,47E-02
TC561001	-	0,777	4,74E-02
XM_001926022	pantothenate kinase 1 (PANK1)	0,773	7,60E-03
EW177256	-	0,762	2,96E-06
TC523118	-	0,756	1,67E-04
AJ964590	-	0,755	6,65E-04
AK343913	AMP010080F05 macrophage	0,750	1,10E-02

TC597684	-	0,747	1,35E-03
ENSSSCT00000023162	SUSD2	0,742	4,82E-02
ENSSSCT00000011662	HSPA12A	0,741	3,14E-02
TC588713	-	0,739	3,52E-02
AF245504	CYP24A1	0,736	1,12E-02
TC575922	-	0,727	4,32E-02
TC548138	-	0,722	1,54E-02
TC574304	-	0,717	4,90E-02
BX920940	-	0,717	4,24E-04
TC531386	-	0,712	1,48E-03
TC575673	-	0,698	3,52E-02
AK392332	-	0,689	3,02E-02
TC601667	-	0,685	1,10E-02
NM_001244160	DHRS7	0,684	2,11E-02
ENSSSCT00000002071	MESP2	0,674	2,63E-02
FS677750	-	0,670	2,96E-06
ENSSSCT00000018037	striatin interacting protein 2 (STRIP2)	0,664	4,80E-02
XM_003126745	Lin-7 homolog A (LIN7A)	0,662	1,28E-02
A_72_P322198	ZBTB44 (tzinc finger and BTB domain containing 44)	0,659	2,71E-03
XM_003124543	amiloride-sensitive sodium channel subunit gamma-like (SCNN1G)	0,656	3,69E-02
NM_001044567	Trans-acting T-cell-specific transcription factor GATA3	0,655	4,53E-02
ENSSSCT00000031509	Nestin (NES)	0,642	2,02E-02
ENSSSCT00000012056	BAMBI	0,640	1,15E-02
NM_213919	Aurora kinase B (AURKB)	0,639	6,77E-03
NM_001146126	Patatin-like phospholipase domain containing 3 (PNPLA3)	0,636	4,11E-02
ENSSSCT00000001271	Histone H2A type 1-F-like	0,632	2,58E-02
ENSSSCT00000014263	REST corepressor 2 (RCOR2)	0,631	2,31E-02
TC605264	-	0,629	4,96E-02
ENSSSCT00000026137	Histone cluster 1. H2bo	0,627	2,44E-04
A_72_P050011	-	0,625	4,21E-08
ENSSSCT00000017147	Myomesin (M-protein) 2	0,621	3,14E-05
ENSSSCT00000025080	E3 Ubiquitin ligase DTX2	0,621	1,07E-02
TC539874	-	0,619	2,09E-03
ENSSSCT00000024917	K(lysine) acetyltransferase 2B (KAT2B)	0,619	1,89E-03
NM_001243656	HIST1H2BD	0,613	4,27E-06
NM_001001261	SLC22A6	0,610	3,39E-02
TC523104	-	0,610	6,78E-03
TC525538	Dual specificity tyrosine phosphorylation regulated kinase 2 (DYRK2)	0,610	3,87E-05
TC599654	-	0,609	3,64E-02
NM_001161637	Claudin 4 (CLDN4)	0,605	4,39E-02
ENSSSCT00000007332	PDZK1	0,604	3,19E-05
ENSSSCT00000025040	NAGS	0,602	3,39E-02
TC564542	-	0,594	2,95E-02
TC589536	-	0,592	7,75E-03

ENSSSCT00000001263	Histone cluster 1. H2bh	0,590	2,96E-06
NM_001243629	cathepsin A (CTSA)	0,587	1,59E-02
NM_001037965	Inhibitor of DNA binding 2 (ID2)	0,584	1,95E-02
ENSSSCT00000006300	DDX31	0,581	7,79E-03
NM_213766	Heat shock protein 70 (HSP70)	0,578	6,21E-03
ENSSSCT00000033515	CD83	0,577	1,89E-03
TC603080	-	0,577	1,35E-04
TC548344	-	0,576	1,44E-02
TC581152	-	0,571	1,95E-02
TC548604	-	0,564	1,40E-03
TC533491	-	0,563	9,79E-03
NM_214299	Heparin-binding EGF-like growth factor (HBEGF)	0,561	2,42E-02
NM_001243410	LACTB2	0,560	3,31E-02
TC625344	-	0,560	7,95E-04
ENSSSCT00000011511	Ubiquitin domain containing 1 (UBTD1)	0,560	1,98E-02
TC559311	-	0,558	1,98E-04
AK394819	-	0,557	3,74E-03
ENSSSCT00000011079	Histone cell cycle regulator (HIRA)	0,557	4,09E-03
TC531076	-	0,556	1,69E-03
ENSSSCT00000023491	Histone H4	0,556	4,09E-03
TC523260	-	0,556	9,79E-03
AK348278	LOC100628052	0,554	2,47E-02
NM_001244501	Glutamate dehydrogenase 1 (GLUD1)	0,552	1,13E-02
BX921275	-	0,552	2,37E-02
TC556284	-	0,551	1,56E-02
ENSSSCT00000036588	KDR	0,551	9,87E-03
ENSSSCT00000006162	PBX3	0,547	4,04E-04
ENSSSCT00000018200	STK31	0,547	1,91E-02
TC560692	-	0,546	3,31E-02
TC617432	-	0,546	9,33E-03
DN123882	-	0,543	1,35E-03
EW461055	-	0,540	4,50E-02
TC588425	-	0,534	1,31E-04
A_72_P222142	FARP1	0,531	1,75E-02
TC612783	-	0,531	1,10E-02
TC610889	-	0,530	5,41E-04
NM_213853	FOLR2	0,529	2,15E-02
TC544492	-	0,529	8,26E-03
ENSSSCT00000011497	TLL2	0,528	1,77E-02
TC575078	-	0,524	7,58E-03
AY610218	SCP2	0,523	9,82E-03
A_72_P104281	INTS7 (Integrator complex subunit 7)	0,523	1,70E-03
ENSSSCT00000007708	PRNP	0,521	2,72E-04
ENSSSCT00000030542	Ubiquitin carboxyl terminal hydrolase 10	0,520	2,53E-02
AK390282	LOC100737343	0,519	2,77E-02

AK347919	OVRM10018E07	0,518	4,65E-03
ENSSSCT00000018941	ATP6V0A1	0,516	4,57E-02
TC586066	-	0,509	2,95E-03
NM_001243807	FKBP10	0,509	4,50E-02
TC534949	-	0,509	2,09E-02
ENSSSCT00000002715	TRIP11	0,506	7,95E-05
ENSSSCT00000026371	ADAM metallopeptidase domain 9 (ADAM9)	0,505	5,68E-03
AJ956332	-	0,504	4,80E-02
AK392417	-	0,502	5,73E-04
**WI+CS24h-CMJ versus Ctl-CMJ** (Down-regulated genes)			
A_72_P231427	Cell division control protein 42 effector protein 1 (CDC42EP1)	-3,738	3,20E-14
A_72_P443884	-	-2,837	1,45E-05
NM_001167835	Cytochrome P450 2C42 (CYP2C42)	-2,837	5,66E-05
ENSSSCT00000015537	Cysteine Dioxygenase Type 1 (CDO1)	-2,607	9,01E-13
TC613147	-	-2,556	1,59E-06
NM_213850	glutathione S-transferase alpha 2 (GSTA2)	-2,559	6,03E-06
A_72_P054101	-	-2,419	2,62E-05
NM_001159306	Pyruvate dehydrogenase kinase isozyme 4, mitochondrial (PDK4)	-2,362	4,21E-07
TC602959	-	-2,273	2,29E-16
TC534148	-	-2,214	9,25E-11
NM_214109	Chymotrypsin-like elastase family, member 2A (CELA2A)	-2,115	6,99E-05
FD592765	-	-2,025	1,32E-07
A_72_P001141	-	-1,987	5,46E-17
KP735782	Isolate S1-V1 glutathione S-transferase A2 (GSTA2)	-1,684	3,49E-05
NM_001134824	CYP3A46	-1,679	6,25E-05
NM_001078665	Chemokine ligand 26-like (CCL26) (MIP-4a, eotaxin-3)	-1,602	1,63E-10
ENSSSCT00000002555	Arginase 2 (ARG2)	-1,437	1,06E-03
TC541539	-	-1,427	3,21E-07
TC584676	-	-1,414	4,72E-08
ENSSSCT00000004065	Facilitator superfamily domain containing 2A (MFSD2A)	-1,396	4,24E-04
TC548575	-	-1,378	9,28E-07
TC562631	-	-1,337	8,85E-10
TC631544	-	-1,317	2,35E-08
ENSSSCT00000007319	TXNIP	-1,287	1,01E-03
TC625961	-	-1,267	3,37E-09
NM_001001539	AKR1B1	-1,263	1,69E-05
DN134227	-	-1,214	5,00E-06
TC598771	-	-1,181	1,25E-07
TC596995	-	-1,172	2,47E-08
AK238381	-	-1,166	7,02E-05
TC590364	-	-1,165	3,68E-07

AK238136	C4BPA	-1,158	8,24E-04
TC544200	-	-1,146	8,35E-04
EW262869	-	-1,141	4,25E-08
AK345001	MX2	-1,140	1,39E-05
CN163990	-	-1,130	6,52E-08
AK236120	CALHM2	-1,127	9,41E-05
TC569278	-	-1,119	3,99E-06
AK232564	-	-1,114	3,80E-05
AK345473	-	-1,093	2,43E-05
TC623278	Sex comb on midleg-like 1 (SCML1)	-1,090	6,71E-04
ENSSSCT00000035428	Arginase 2 (ARG2)	-1,088	1,00E-07
FD604545	-	-1,087	8,52E-06
TC566965	-	-1,082	4,91E-04
DN102262	-	-1,070	5,60E-07
TC566172	-	-1,062	6,55E-06
CV871207	-	-1,056	4,48E-07
TC548865	-	-1,056	7,35E-09
TC571068	-	-1,054	2,89E-08
NM_213962	Hydroxyacyl-CoA dehydrogenase trifunctional multienzyme complex subunit alpha (HADHA)	-1,020	6,72E-05
NM_001287412	Nuclear receptor subfamily 1H4 (NR1H4)	-1,016	2,61E-06
NM_001101027	CYP39A1	-1,014	7,32E-10
TC538121	-	-1,010	1,57E-10
TC531633	LOC107521672	-1,008	1,86E-09
AK350112	ISG15	-1,006	1,66E-03
TC604056	-	-0,987	6,30E-05
TC532659	-	-0,984	2,32E-05
TC576734	Inter-alpha-trypsin inhibitor heavy chain H1 precursor	-0,982	4,95E-05
NM_001163696	C13H21orf62	-0,980	1,26E-04
NM_001097452	C8G	-0,979	2,35E-07
NM_001044573	Angiogenin, ribonuclease, RNase A family, 5 (ANG)	-0,978	2,22E-04
TC541448	-	-0,978	4,77E-04
TC559094	-	-0,974	7,23E-08
NM_001243452	DNA-damage-inducible transcript 4 (DDIT4)	-0,970	2,16E-06
ENSSSCT00000007615	WDR63	-0,960	2,60E-05
ENSSSCT00000001838	Peroxisomal biogenesis factor 6 (PEX6)	-0,954	1,31E-04
A_72_P273929	MATP44 (Interferon induced 44 associated)	-0,949	6,59E-04
NM_001114056	MHC class I antigen 5 (SLA-5)	-0,941	1,28E-04
NM_001122990	NNAT	-0,933	3,53E-06
TC601797	Mitochondrial Carnitine O-palmitoyltransferase 2	-0,930	2,52E-06
TC541610	-	-0,924	6,18E-04
NM_214246	carboxylesterase 1 (CES1)	-0,906	8,37E-04
NM_001195115	Solute carrier family 25. member 27 (SLC25A27)	-0,905	1,72E-06
TC560156	-	-0,903	4,29E-10
NM_213742	Calcitonin-related polypeptide beta (CALCB)	-0,892	9,82E-07

NM_001315766	LOC100736962	-0,890	5,72E-04
ENSSSCT00000004670	Tumor protein D52-like 1 (TPD52L1)	-0,885	8,64E-08
NM_001044592	Coagulation factor X (F10)	-0,881	7,11E-04
TC536661	-	-0,877	4,71E-07
TC557164	2'-5' oligoadenylate synthetase 2 (OAS)	-0,877	1,70E-03
TC568621	-	-0,872	3,02E-06
ENSSSCT00000034425	Mitogen-Activated Protein Kinase Kinase Kinase 8 (MAP3K8)	-0,870	1,57E-07
A_72_P032671	CLEC2D	-0,854	1,13E-03
EW034563	-	-0,852	7,40E-06
TC530663	-	-0,843	1,40E-03
NM_001128474	Guanylate binding protein 2, interferon-inducible (GBP2)	-0,840	2,91E-07
TC547562	Zinc finger protein 473 (ZNF473)	-0,836	4,93E-06
TC575827	-	-0,836	1,06E-06
TC592596	-	-0,826	4,45E-04
ENSSSCT00000034119	Family with sequence similarity 122C	-0,823	5,82E-12
A_72_P404593	TXNIP (Thioredoxin-interacting protein)	-0,821	4,87E-09
NM_001098590	Hermansky-Pudlak syndrome 6 (HPS6)	-0,820	5,27E-05
TC623045	-	-0,817	8,02E-05
ENSSSCT00000028953	Zinc finger protein 114	-0,803	7,38E-04
TC555339	-	-0,802	2,01E-06
TC598894	-	-0,802	1,26E-04
AK232386	MX2	-0,800	2,16E-04
EW261758	-	-0,800	1,48E-11
ENSSSCT00000002574	Serine/arginine-rich splicing factor 5	-0,798	8,64E-10
TC526612	-	-0,798	3,37E-04
A_72_P399733	-	-0,797	4,30E-08
XM_013981732	GNAT1	-0,795	2,30E-06
XR_001309264	LOC102158887	-0,795	1,00E-05
NM_001097416	MX2	-0,794	9,92E-05
AK343323	-	-0,791	1,53E-04
AK346233	LOC100515741	-0,787	1,68E-09
ENSSSCT00000004577	CITED2	-0,781	7,62E-09
TC548784	-	-0,774	4,84E-05
TC528319	-	-0,774	1,43E-03
TC532382	-	-0,773	9,27E-04
TC621729	-	-0,772	6,42E-05
TC587447	-	-0,767	2,08E-05
BX914375	-	-0,766	1,64E-05
NM_001185143	SLA-DOA	-0,764	6,76E-07
NM_001097501	LGALS3	-0,762	1,25E-06
NM_001097506	AN1-type zinc finger protein 5 (ZFAND5)	-0,758	4,31E-08
TC576690	-	-0,756	7,03E-04
TC610412	-	-0,753	1,42E-04
NM_001243362	Lactamase, beta (LACTB)	-0,747	7,22E-06
TC602146	-	-0,745	8,96E-04

NM_001134349	Kruppel-like factor 15 (KLF15)	-0,745	4,44E-07
CA779378	-	-0,739	1,32E-03
TC566289	-	-0,739	4,84E-04
TC627807	LOC106504276	-0,738	3,51E-05
TC533634	-	-0,736	4,74E-09
CF365587	-	-0,729	4,27E-04
A_72_P049531	GRB14 (growth factor receptor bound protein 14)	-0,729	3,87E-04
CK454137	-	-0,729	6,24E-04
BX675220	-	-0,725	2,13E-07
NM_001024696	TNFSF10	-0,725	6,90E-05
TC600327	-	-0,721	8,78E-04
TC543158	Histamine N-methyltransferase	-0,719	5,90E-05
AK237715	SH3BP2	-0,716	1,68E-13
TC525588	-	-0,716	7,47E-04
ENSSSCT00000006875	CCAAT/enhancer binding protein (C/EBP), delta (CEBPD)	-0,715	2,26E-07
AK399777	SRSF5	-0,713	8,04E-07
NM_001143719	MAP kinase interacting serine/threonine kinase 1 (MKNK1)	-0,712	2,54E-06
NM_214425	CYP4A21	-0,712	1,83E-07
AK232543	-	-0,707	3,17E-04
TC595575	-	-0,706	1,44E-09
DN132923	-	-0,705	1,04E-04
ENSSSCT00000000924	Thymine DNA glycosylase	-0,704	1,27E-03
ENSSSCT00000033355	S100A4	-0,703	5,48E-04
TC567905	-	-0,700	2,42E-04
AK347470	LOC100624597	-0,700	7,52E-05
TC535436	-	-0,698	1,67E-04
A_72_P431054	Mannan-binding lectin serine peptidase 2 (MASP2)	-0,696	1,94E-06
BP446680	-	-0,693	3,28E-07
ENSSSCT00000029598	phosphatidylinositol glycan anchor biosynthesis, class H (PIGH)	-0,692	2,08E-06
ENSSSCT00000015179	Arrestin domain containing 2 (ARRDC2)	-0,690	2,66E-05
ENSSSCT00000012586	Succinate-CoA ligase. GDP-forming. beta subunit	-0,690	1,64E-06
ENSSSCT00000010653	carbonyl reductase 4 (CBR4)	-0,689	2,95E-05
TC574870	-	-0,689	1,93E-05
TC602526	-	-0,688	6,84E-05
TC543804	-	-0,682	1,42E-05
CO992076	Endometrium gilt D6 of estrous cycle	-0,682	5,50E-12
ENSSSCT00000009671	RASL11B	-0,682	4,41E-04
CV873561	-	-0,681	4,44E-04
ENSSSCT00000009138	Rho GTPase activating protein 25 (ARHGAP25)	-0,675	7,75E-09
ENSSSCT00000027137	Neuromedin-U-like	-0,675	1,08E-10
TC529859	-	-0,675	1,55E-04
AK346234	LOC100520861	-0,673	3,29E-04
AK236259	TRMT6	-0,672	9,41E-04
AK399810	S100A4	-0,672	1,20E-03
A_72_P297874	Dysbindin domain containing 1 (DBNDD1)	-0,670	1,88E-04

TC562329	-	-0,667	3,60E-06
CK462660	-	-0,659	4,79E-05
AK393873	-	-0,658	3,47E-04
TC621173	-	-0,657	7,66E-04
TC547188	-	-0,657	5,03E-11
TC614889	-	-0,653	3,89E-05
DB804246	-	-0,653	1,33E-07
NM_214228	prostaglandin D2 synthase (PTGDS)	-0,652	6,74E-05
TC550878	-	-0,651	3,68E-04
DN120432	-	-0,651	1,02E-04
AK350920	-	-0,649	6,71E-06
TC527407	-	-0,648	2,00E-05
NM_001123217	ZNF217	-0,643	6,88E-09
NM_001245012	Inositol polyphosphate-1-phosphatase (INPP1)	-0,642	8,72E-06
EW221235	-	-0,641	4,97E-05
NM_001145222	RBP7	-0,639	2,91E-04
NM_001246243	Carnitine palmitoyltransferase 2 (CPT2)	-0,637	2,79E-04
TC532111	-	-0,636	1,37E-04
AK352378	VPS51	-0,632	3,07E-04
DN104045	-	-0,630	1,99E-06
ENSSSCT00000011130	Mitogen-activated protein kinase kinase kinase 21 (MLK4)	-0,630	1,96E-05
NM_001190184	DnaJ (Hsp40) homolog, subfamily C, member 3 (DNAJC3)	-0,630	4,93E-05
TC555476	-	-0,628	6,61E-06
ENSSSCT00000000715	Ovostatin precursor	-0,626	1,56E-03
AK351081	-	-0,624	5,95E-04
TC531848	-	-0,624	1,64E-05
TC531203	-	-0,623	6,57E-04
NP276891	Major histocompatibility complex class I antigen	-0,623	1,13E-03
NM_001123200	TES	-0,622	1,16E-05
BX667161	-	-0,618	3,63E-04
AK392845	RNASET2	-0,616	1,53E-05
ENSSSCT00000017757	Ubiquitin specific peptidase 40	-0,609	9,96E-05
TC580089	-	-0,606	1,08E-03
AK233683	C8G	-0,603	2,28E-07
A_72_P103106	-	-0,603	9,52E-05
ENSSSCT00000013085	ABI family member 3 binding protein	-0,593	3,83E-07
NM_214385	prostaglandin reductase 1 (PTGR1)	-0,591	7,00E-04
TC519529	-	-0,591	5,44E-06
AW416039	-	-0,591	1,10E-03
AJ959301	-	-0,590	5,09E-04
TC606417	SJCHGC06720 protein	-0,589	1,45E-04
EW642054	-	-0,588	1,41E-06
AJ961685	-	-0,587	1,31E-06
A_72_P429354	Proteasomal ATPase associated factor 1 (PAAF1)	-0,586	1,82E-07

CN157777	-	-0,586	1,22E-03
A_72_P129976	-	-0,585	6,01E-05
ENSSSCT00000003166	G patch domain containing 1	-0,583	9,83E-04
TC576666	MGC179351 protein	-0,582	8,42E-06
TC557192	-	-0,582	2,53E-07
TC606037	-	-0,581	7,54E-05
TC527857	-	-0,581	1,61E-03
AK394979	LOC100156195	-0,577	1,48E-04
TC613549	-	-0,577	2,04E-09
TC582618	-	-0,576	1,44E-11
AK391669	CITED2	-0,576	1,39E-04
ENSSSCT00000023435	TNF receptor associated factor 3 (TRAF3)	-0,576	1,03E-04
NM_001136511	HUS1	-0,576	1,54E-04
TC582968	-	-0,573	1,49E-03
AK347987	-	-0,573	4,60E-08
AK234441	LOC100153598	-0,573	4,33E-04
TC538766	-	-0,573	4,96E-04
TC536098	-	-0,572	6,63E-04
TC575521	-	-0,570	5,38E-04
TC590063	-	-0,568	4,56E-04
XM_003128651	Lysine-specific demethylase NO66-like	-0,568	1,68E-04
NM_001243304	CYP27A1	-0,568	7,27E-04
TC599915	-	-0,568	1,76E-06
EW200629	-	-0,566	2,08E-05
ENSSSCT00000005804	Transient receptor potential cation channel M-6 (TRPM6)	-0,566	1,68E-03
TC618316	-	-0,565	7,12E-05
TC578726	-	-0,565	8,83E-05
TC584539	-	-0,565	8,84E-04
AK230805	ZSCAN21	-0,564	5,79E-04
A_72_P018866	-	-0,563	1,06E-05
TC564615	-	-0,563	1,98E-05
TC533014	-	-0,562	9,84E-04
TC542747	-	-0,562	5,28E-05
AK396196	-	-0,560	1,98E-05
TC621175	-	-0,558	3,65E-05
TC559375	-	-0,557	1,09E-10
A_72_P345793	-	-0,557	8,03E-07
TC545140	-	-0,557	4,02E-05
TC585103	MGLL protein	-0,556	1,62E-03
XM_001926065	LIMK2	-0,555	2,59E-05
CN162068	-	-0,554	1,01E-03
AK346354	C-1-tetrahydrofolate synthase, cytoplasmic (MTHFD1)	-0,553	3,45E-06
XM_003129835	Exophilin 5 (EXPH5)	-0,552	3,29E-05
NM_001123134	LIPA	-0,551	1,70E-03
A_72_P118726	-	-0,551	1,41E-04

TC525370	-	-0,550	6,04E-06
ENSSSCT00000006821	Tocopherol (alpha) transfer protein	-0,550	1,62E-04
TC599031	-	-0,549	2,64E-06
A_72_P010816	MMAA	-0,549	4,03E-04
TC629544	-	-0,548	1,00E-03
A_72_P206912	-	-0,547	1,09E-03
AJ961798	-	-0,547	1,24E-05
TC560803	-	-0,546	7,36E-05
ENSSSCT00000025705	Poly(ADP-ribose) polymerase family member 4 (PARP4)	-0,545	1,42E-05
TC531472	LOC108300678	-0,544	1,14E-03
ENSSSCT00000002527	MTHFD1	-0,544	9,21E-04
AK345540	-	-0,543	2,35E-04
AK345509	CLEC1A	-0,542	4,49E-04
TC546244	-	-0,541	1,32E-03
ENSSSCT00000010582	KCTD9	-0,540	1,13E-07
TC533090	-	-0,537	3,67E-04
XM_013978816	Annexin A3 (ANXA3)	-0,536	1,00E-04
NM_001111258	IFNGR2	-0,536	3,90E-06
BX924742	-	-0,536	6,38E-06
NM_214399	IL6	-0,533	1,84E-08
TC565249	-	-0,533	2,48E-06
TC608215	-	-0,533	1,95E-05
NM_001048187	DLK1	-0,529	3,90E-05
TC592842	-	-0,528	9,25E-04
TC531686	Zinc finger protein 26	-0,526	2,39E-04
ENSSSCT00000004450	RNASET2	-0,526	5,33E-04
TC528683	-	-0,525	9,49E-07
TC609759	-	-0,524	2,53E-08
ENSSSCT00000018493	STC2	-0,524	3,03E-05
NM_213851	Phosphoinositide-3-kinase, regulatory subunit 5 (PIK3R5)	-0,522	9,19E-04
TC576331	-	-0,521	1,22E-06
ENSSSCT00000031997	TMEM150C	-0,521	1,42E-03
TC583980	-	-0,521	3,73E-07
TC532501	-	-0,519	6,61E-04
AK236308	-	-0,518	2,58E-08
TC581829	-	-0,516	1,43E-08
TC539177	-	-0,515	3,54E-06
TC552040	-	-0,515	8,52E-06
TC564362	-	-0,509	8,25E-04
TC522007	-	-0,508	4,19E-06
TC546925	-	-0,507	8,10E-05
AK394207	LVRM10091F11	-0,507	1,69E-04
BX677249	-	-0,506	1,69E-03
TC614683	-	-0,504	1,22E-05
AK237396	SIK2	-0,503	5,10E-07

BX924155	-	-0,501	3,24E-04
ENSSSCT00000000994	SOCS2	-0,500	1,39E-03
**WI+CS24h-CMJ versus Ctl-CMJ** (Up-regulated genes)			
TC611155	-	3,000	2,56E-15
ENSSSCT00000002650	Proto-oncogene c-Fos (FOS)	2,846	7,04E-15
NM_001123113	Fos proto-oncogene, AP-1 transcription factor subunit	2,816	1,73E-14
TC529750	-	2,430	9,66E-17
BF444493	-	2,198	5,33E-04
NM_001190276	nuclear receptor subfamily 4, group A, member 2 (NR4A2)	2,076	2,39E-08
DR066068	-	1,979	4,32E-07
NM_001097505	BTG2	1,916	3,25E-09
TC611786	-	1,904	3,17E-07
JN899104	Small nucleolar RNA, C/D box 3 cluster (pSNORD3)	1,838	2,46E-08
AK234282	-	1,544	2,47E-05
NM_214069	Fucosyltransferase 2 (FUT2)	1,538	1,57E-09
NM_213868	Ficolin (collagen/fibrinogen domain containing lectin) 2 (FCN2)	1,490	1,17E-06
TC521842	-	1,455	4,39E-10
ENSSSCT00000025681	CRABP1	1,429	1,30E-04
TC552941	-	1,405	7,66E-20
TC575360	-	1,395	3,24E-07
TC557333	-	1,360	6,17E-04
NM_001130532	Par-6 family cell polarity regulator beta (PARD6B)	1,285	3,38E-06
TC564655	-	1,280	1,31E-06
AK347361	HLX	1,253	2,81E-06
ENSSSCT00000012922	B-cell lymphoma 6 protein (BCL6)	1,240	7,43E-06
TC577359	-	1,237	1,97E-05
TC570078	-	1,234	2,00E-10
NM_214105	Guanylate cyclase 2C (heat stable enterotoxin receptor) (GUCY2C)	1,228	7,29E-05
TC595819	-	1,225	4,15E-04
TC561142	-	1,205	6,76E-05
AK395517	-	1,171	2,77E-04
ENSSSCT00000015660	Early growth response protein 1 (EGR1)	1,163	7,40E-12
A_72_P443393	-	1,152	1,70E-03
ENSSSCT00000036290	Transcription factor AP-1 (JUN)	1,149	2,39E-06
ENSSSCT00000031521	LY6/PLAUR domain containing 6 (LYPD6)	1,133	5,26E-07
TC543988	-	1,115	6,73E-04
TC546352	-	1,109	4,07E-06
A_72_P431755	-	1,105	1,10E-03
TC556782	-	1,096	5,82E-06
TC566133	-	1,096	2,28E-07
TC605691	V-set domain containing T cell activation inhibitor 1 (VTCN1)	1,087	1,78E-04
CN161830	-	1,073	1,05E-03
TC525402	Pleckstrin homology-like domain family A member 2	1,066	5,78E-05
NM_214214	C-C motif chemokine 2 (CCL2)	1,055	7,70E-06

TC593464	-	1,036	4,02E-04
AK237109	PBL010107H06 in PBMC	1,036	1,15E-03
NM_214055	IL1B1	1,033	1,98E-05
NM_213880	Transcription factor AP-1 (JUN)	1,024	3,95E-05
NM_001113447	phytanoyl-CoA 2-hydroxylase (PHYH)	1,024	2,47E-12
ENSSSCT00000004344	Serine/threonine-protein kinase (PLK3)	1,022	2,41E-07
AK389182	FAM43B	1,022	7,50E-04
ENSSSCT00000010370	DIS3 homolog, exosome endoribonuclease and 3'-5' exoribonuclease	0,974	3,91E-04
ENSSSCT00000024134	phytanoyl-CoA 2-hydroxylase (PHYH)	0,959	2,50E-09
XM_001929359	ATP binding cassette subfamily C member 2 (ABCC2)	0,949	1,68E-10
XR_001305510	LOC106508566	0,946	1,23E-04
AK343913	AMP010080F05 macrophage	0,933	7,99E-06
ENSSSCT00000001267	Histone H2A type 1-like (HIST1H2AB)	0,930	3,25E-06
AJ660252	-	0,922	5,84E-07
AK351387	-	0,916	4,20E-12
NM_214277	ANPEP	0,907	1,42E-03
AK237533	SPL010057H01	0,895	6,69E-04
TC537380	-	0,888	4,91E-10
AK351795	CD83	0,875	2,91E-06
ENSSSCT00000027822	HSPA12A	0,872	1,08E-03
A_72_P139871	GPD1L (glycerol-3-phosphate dehydrogenase 1-like)	0,871	5,06E-04
TC618296	CCAAT/enhancer binding protein (C/EBP) (CEBPA)	0,868	1,51E-09
DY407595	-	0,867	7,05E-07
A_72_P773975	-	0,843	2,85E-04
TC523118	-	0,838	8,28E-08
NM_001044600	RGS2	0,835	3,61E-04
TC569507	-	0,821	1,86E-05
TC533770	Protein ENSP00000381892	0,810	3,46E-06
ENSSSCT00000022578	Mucolipin 3 (MCOLN3)	0,803	8,14E-04
TC525987	-	0,801	4,10E-04
TC546915	-	0,781	1,30E-03
AK348278	LOC100628052	0,777	5,68E-06
XM_003124543	amiloride-sensitive sodium channel subunit gamma-like (SCNN1G)	0,776	1,47E-04
TC574741	-	0,772	2,40E-10
ENSSSCT00000026137	Histone cluster 1. H2bo	0,759	2,38E-08
NM_001044567	Trans-acting T-cell-specific transcription factor GATA3	0,756	2,87E-04
NM_001161637	Claudin 4 (CLDN4)	0,756	1,00E-04
XM_001926022	pantothenate kinase 1 (PANK1)	0,746	1,54E-04
BI339629	-	0,745	1,70E-03
EW177256	-	0,744	6,73E-09
TC605264	-	0,733	3,07E-04
TC588713	-	0,731	1,01E-03
AK392332	-	0,730	3,74E-04
TC586066	-	0,721	6,32E-08
ENSSSCT00000012056	BAMBI	0,717	4,13E-05

A_72_P322198	Zinc finger and BTB domain containing 44 (ZBTB44)	0,717	6,48E-06
AJ648501	-	0,716	1,11E-03
NM_001031782	Krueppel-like factor 4 (KLF4)	0,713	1,11E-03
ENSSSCT00000001263	Histone cluster 1. H2bh	0,711	4,84E-11
ENSSSCT00000011534	ATP binding cassette subfamily C member 2 (ABCC2)	0,705	2,89E-05
NM_001243656	Histone cluster 1. H2bd (HIST1H2BD)	0,704	2,56E-10
TC548138	-	0,693	4,39E-04
NM_001037965	Inhibitor of DNA binding 2 (ID2)	0,692	4,49E-05
BX920940	-	0,685	4,44E-06
ENSSSCT00000014263	REST corepressor 2 (RCOR2)	0,680	2,00E-04
TC597684	-	0,676	4,08E-05
ENSSSCT00000001271	Histone H2A type 1-F-like	0,676	2,65E-04
A_72_P050011	-	0,673	3,58E-12
NM_001039746	fatty acid binding protein 5 (FABP5)	0,673	7,46E-04
ENSSSCT00000031509	Nestin (NES)	0,671	2,34E-04
NM_001128492	Cellular retinoic acid binding protein 1 (CRABP1)	0,663	1,66E-05
AJ964590	-	0,662	2,90E-05
ENSSSCT00000036588	KDR	0,660	1,13E-05
A_72_P218807	-	0,655	5,27E-04
ENSSSCT00000017147	Myomesin (M-protein) 2	0,655	2,53E-08
TC573765	-	0,654	4,96E-04
NM_213913	HSD11B2	0,653	3,59E-04
ENSSSCT00000033656	CLDN4	0,650	7,92E-04
AF245504	CYP24A1	0,650	7,19E-04
ENSSSCT00000025040	NAGS	0,642	4,21E-04
TC603080	-	0,639	6,58E-08
ENSSSCT00000026684	DDIT3	0,630	3,30E-04
TC617432	-	0,628	1,94E-05
XM_003126745	Lin-7 homolog A (LIN7A)	0,619	4,55E-04
TC601667	-	0,608	6,58E-04
AK394819	-	0,607	1,04E-05
TC532019	-	0,604	7,14E-04
TC531076	-	0,601	3,67E-06
TC559311	-	0,599	1,98E-07
EW350228	-	0,598	6,61E-04
ENSSSCT00000025080	E3 Ubiquitin ligase DTX2	0,595	2,61E-04
ENSSSCT00000023491	Histone H4	0,595	1,58E-05
ENSSSCT00000024917	K(lysine) acetyltransferase 2B (KAT2B)	0,593	2,83E-05
NM_214159	PIGR	0,592	5,74E-04
ENSSSCT00000036684	ENTPD1	0,590	3,18E-05
NM_213766	Heat shock protein 70 (HSP70)	0,582	6,60E-05
AK237461	NXF1	0,582	1,35E-03
EW461356	-	0,577	1,74E-04
NM_001134353	KLF6	0,575	5,86E-07
ENSSSCT00000017407	HOXD1	0,575	1,28E-03

A_72_P144706	-	0,569	9,25E-05
A_72_P321533	-	0,569	1,14E-03
AK392417	-	0,565	4,08E-07
FS677750	-	0,564	1,47E-07
TC534949	-	0,564	1,21E-04
TC548344	-	0,563	3,27E-04
NP276701	-	0,562	1,78E-04
TC590749	-	0,557	3,56E-05
TC548604	-	0,556	1,25E-05
TC521243	Proheparin-binding EGF-like growth factor precursor	0,552	1,12E-03
TC531386	-	0,552	3,26E-04
TC525538	Dual specificity tyrosine phosphorylation regulated kinase 2 (DYRK2)	0,550	6,93E-07
TC533491	-	0,549	1,88E-04
TC610889	-	0,548	1,65E-06
NM_213919	Aurora kinase B (AURKB)	0,546	5,47E-04
TC539874	-	0,546	9,76E-05
ENSSSCT00000019143	PPP1R9B	0,545	9,04E-06
AJ956332	-	0,544	6,61E-04
ENSSSCT00000006162	PBX3	0,544	2,14E-06
ENSSSCT00000033515	CD83	0,543	3,65E-05
AY610218	SCP2	0,541	9,21E-05
A_72_P104281	Integrator complex subunit 7 (INTS7)	0,540	7,82E-06
ENSSSCT00000007306	Histone cluster 2. H2ab	0,539	3,82E-09
NM_001243807	FKBP10	0,539	7,26E-04
AK400200	LOC102159136	0,535	9,95E-04
TC553832	-	0,533	2,90E-04
TC547779	-	0,526	2,55E-09
ENSSSCT00000003857	EPH receptor A2 (EPHA2)	0,525	1,19E-03
ENSSSCT00000030078	peptide YY (PYY)	0,525	1,11E-03
TC531463	-	0,523	1,52E-04
ENSSSCT00000018200	STK31	0,523	6,06E-04
TC623684	-	0,523	1,57E-04
ENSSSCT00000010394	RBM26	0,523	2,07E-11
TC560692	-	0,522	1,28E-03
AK343570	SP110	0,519	5,91E-07
ENSSSCT00000005460	Mothers against decapentaplegic homolog 6 (SMAD6)	0,518	9,49E-08
ENSSSCT00000019412	RAP1 GTPase activating protein 2	0,518	2,04E-04
NM_214299	Heparin-binding EGF-like growth factor (HBEGF)	0,513	1,26E-03
ENSSSCT00000012833	membrane metallo-endopeptidase (MME)	0,511	2,70E-04
NM_001244501	Glutamate dehydrogenase 1 (GLUD1)	0,510	4,42E-04
NM_001243629	cathepsin A (CTSA)	0,510	1,30E-03
ENSSSCT00000012034	LOC106504139	0,508	5,32E-04
TC556284	-	0,508	6,89E-04
ENSSSCT00000005197	Microtubule associated 1A MAP 1A	0,507	5,70E-04

A_72_P222142	FARP1	0,505	5,67E-04
ENSSSCT00000010405	dopachrome tautomerase (DCT)	0,505	1,14E-04
ENSSSCT00000007332	PDZK1	0,504	2,20E-06
ENSSSCT00000010315	ZC3H13	0,504	3,23E-04
NM_001244417	RHOV	0,501	2,11E-04
AK234955	-	0,501	1,54E-05
**WI+CS6h-CMJ versus WI-CMJ** (Down-regulated genes)			
NM_001167835	Cytochrome P450 2C42 (CYP2C42)	-2,828	5,31E-03
NM_214417	CYP2A19	-2,673	1,18E-02
ENSSSCT00000015537	Cysteine Dioxygenase Type 1 (CDO1)	-2,605	8,25E-09
A_72_P443884	-	-2,603	4,73E-03
NM_213845	Uteroferrin associated basic protein-2 (UABP-2)	-2,581	4,90E-02
A_72_P054101	-	-2,561	1,67E-03
NM_001159306	Pyruvate dehydrogenase kinase isozyme 4, mitochondrial (PDK4)	-2,495	7,94E-05
NM_213850	Glutathione S-transferase alpha 2 (GSTA2)	-2,423	1,65E-03
A_72_P773461	IGLV (IG Lambda chain V)	-2,227	1,59E-02
NM_214109	Chymotrypsin-like elastase family, member 2A (CELA2A)	-2,149	5,11E-03
TC613147	-	-2,048	4,69E-03
TC534148	-	-1,989	2,15E-06
KP735782	Isolate S1-V1 glutathione S-transferase A2 (GSTA2)	-1,739	2,60E-03
ENSSSCT00000029761	Immunoglobulin heavy constant Ig chain	-1,682	1,07E-02
ENSSSCT00000029761	IgA C alpha	-1,682	1,07E-02
NM_001078665	Chemokine ligand 26-like (CCL26) (MIP-4a, eotaxin-3)	-1,636	3,23E-07
JX092267	Adiponectin, C1Q And Collagen Domain Containing (ADIPOQ)	-1,630	3,66E-03
NM_214200	Perilipin 2 (PLIN2)	-1,592	2,12E-02
ENSSSCT00000026328	Vitamin D binding protein	-1,566	4,27E-02
NM_001134824	CYP3A46	-1,545	1,19E-02
EF601160	Adiponectin, C1Q And Collagen Domain Containing (ADIPOQ)	-1,469	2,24E-03
TC587527	-	-1,453	6,14E-04
NM_213962	Hydroxyacyl-CoA dehydrogenase trifunctional multienzyme complex subunit alpha (HADHA)	-1,402	1,65E-04
NM_214134	Erythropoietin (EPO)	-1,368	1,07E-02
A_72_P376423	-	-1,353	1,58E-04
NM_214422	CYP3A39	-1,345	2,77E-02
NM_001163696	Chromosome 13 open reading frame (C13H21orf62)	-1,308	4,82E-04
AK236120	CALHM2	-1,304	1,75E-03
NM_001044592	Coagulation factor X (F10)	-1,278	9,53E-04
NM_213988	SRD5A2	-1,258	4,01E-02
AK345473	-	-1,256	6,31E-04
EV864657	COL8A1	-1,242	1,82E-03
TC544200	-	-1,239	1,87E-02
NM_001114056	MHC class I antigen 5 (SLA-5)	-1,224	6,19E-04
AK238381	-	-1,184	5,20E-03
ENSSSCT00000019139	Collagen type I (COL1A1)	-1,182	2,95E-02

TC566172	-	-1,141	5,35E-04
EW262869	-	-1,140	3,61E-05
NM_001101027	CYP39A1	-1,132	2,29E-07
TC598771	-	-1,111	1,62E-04
TC569278	-	-1,102	9,53E-04
TC571068	-	-1,078	2,09E-05
AK392845	RNASET2	-1,067	8,39E-07
AK232564	-	-1,067	5,98E-03
TC604056	-	-1,062	2,63E-03
TC552284	-	-1,052	2,13E-02
NM_001122990	Neuronatin (NNAT)	-1,042	1,88E-04
ENSSSCT00000006766	Peptidase inhibitor 15 (PI15)	-1,041	1,17E-03
ENSSSCT00000035428	Arginase 2 (ARG2)	-1,023	1,42E-04
NM_214425	CYP4A21	-1,020	2,53E-07
ENSSSCT00000009671	RASL11B	-1,014	4,66E-04
NM_214228	prostaglandin D2 synthase (PTGDS)	-1,011	3,39E-05
NM_001315766	Carboxylesterase	-1,011	9,53E-03
NM_214337	protein phosphatase 1, regulatory (inhibitor) subunit 14A (PPP1R14A)	-1,003	7,70E-03
TC596995	-	-0,999	2,05E-04
NM_001097452	Complement component 8. gamma polypeptide (C8G)	-0,992	9,41E-05
TC541539	-	-0,987	8,31E-03
NM_001097431	SLA-1	-0,986	3,27E-02
NM_001315568	Glutathione S-transferase theta 1 (GSTT1)	-0,982	2,97E-02
TC595529	MGC148805 protein	-0,973	9,12E-03
NM_214246	Carboxylesterase 1 (CES1)	-0,969	2,04E-02
NM_001185143	SLA-DOA	-0,967	7,83E-06
TC590364	-	-0,962	1,53E-03
CV871207	-	-0,956	6,14E-04
NM_001083941	Angiotensin I converting enzyme (ACE)	-0,950	2,53E-02
NM_001113438	AQP7	-0,945	4,37E-02
NM_213932	LGALS9	-0,940	1,19E-02
AK393941	ABL2	-0,922	3,19E-02
ENSSSCT00000006875	CCAAT/enhancer binding protein (C/EBP), delta (CEBPD)	-0,914	2,18E-06
NM_001098605	PNPLA2	-0,897	5,66E-03
XR_001309264	LOC102158887	-0,897	4,13E-04
ENSSSCT00000001838	Peroxisomal biogenesis factor 6 (PEX6)	-0,895	1,59E-02
TC536661	-	-0,885	1,62E-04
NM_001048187	DLK1	-0,885	5,04E-06
ENSSSCT00000004450	RNASET2	-0,882	1,18E-04
BX914375	-	-0,874	5,25E-04
NM_001130243	troponin C type 1 (TNNC1)	-0,874	4,39E-02
AK235420	-	-0,873	1,99E-02
TC600327	-	-0,871	7,76E-03
NM_001244729	C3H2orf40	-0,871	2,45E-03

ENSSSCT00000010309	Leucine Rich Repeats And Calponin Homology Domain Containing 1 (LRCH1)	-0,871	2,26E-02
ENSSSCT00000035093	Angiotensin-converting enzyme 2 precursor (ACE)	-0,862	1,62E-02
TC548575	-	-0,858	2,96E-02
NM_001243452	DNA-damage-inducible transcript 4 (DDIT4)	-0,855	2,16E-03
NM_001134349	Kruppel-like factor 15 (KLF15)	-0,850	3,01E-05
ENSSSCT00000003289	Actinin, alpha 4 (ACTN4)	-0,848	1,40E-02
NM_214153	ENTPD1	-0,842	1,09E-04
EW034563	-	-0,838	1,49E-03
ENSSSCT00000006282	laminin. gamma 3	-0,835	1,43E-02
ENSSSCT00000010983	PIK3IP1	-0,832	1,40E-03
ENSSSCT00000002764	LOC100153899	-0,829	1,79E-06
TC560045	Dephospho-CoA kinase domain-containing protein	-0,826	2,18E-02
NM_001164652	Aggrecan (ACAN)	-0,821	1,38E-02
NM_001044573	Angiogenin, ribonuclease, RNase A family, 5 (ANG)	-0,820	4,74E-02
NM_001110172	Aquaporine 3 (AQP3)	-0,814	2,26E-03
A_72_P297874	Dysbindin domain containing 1 (DBNDD1)	-0,814	1,86E-03
TC543804	-	-0,812	2,63E-04
TC601797	Carnitine O-palmitoyltransferase 2. mitochondrial	-0,809	2,64E-03
AK348497	-	-0,809	2,97E-02
TC533634	-	-0,802	1,48E-06
NM_001143719	MAP kinase interacting serine/threonine kinase 1 (MKNK1)	-0,802	1,32E-04
CA779378	-	-0,800	2,59E-02
NM_001243304	CYP27A1	-0,799	1,40E-03
ENSSSCT00000024997	S100A2	-0,798	3,30E-02
NM_001246243	Carnitine palmitoyltransferase 2 (CPT2)	-0,792	2,02E-03
FD604545	-	-0,791	2,51E-02
TC610430	-	-0,788	3,53E-02
ENSSSCT00000017924	ZYX	-0,788	5,07E-03
TC531848	-	-0,774	1,67E-04
NM_001097506	AN1-type zinc finger protein 5 (ZFAND5)	-0,768	3,01E-05
ENSSSCT00000016639	FMOD	-0,764	8,38E-03
TC560156	-	-0,763	2,10E-05
ENSSSCT00000012586	Succinate-CoA ligase. GDP-forming. beta subunit	-0,762	1,25E-04
TC626203	-	-0,757	4,10E-02
NM_214099	IGFBP5	-0,757	4,16E-02
ENSSSCT00000013052	CCDC80	-0,756	3,00E-02
TC568621	-	-0,754	3,12E-03
ENSSSCT00000028877	Aminoacylproline aminopeptidase prolidase	-0,741	1,27E-03
ENSSSCT00000019531	Acyl-CoA dehydrogenase very long chain (ACADVL)	-0,740	1,07E-02
ENSSSCT00000027574	ID4	-0,732	2,63E-02
AK351603	-	-0,730	1,64E-02
AK391674	-	-0,730	4,22E-02
ENSSSCT00000007254	Tuftelin 1 (TUFT1)	-0,725	2,33E-03
TC519903	-	-0,723	2,15E-02

ENSSSCT00000023435	TNF receptor associated factor 3 (TRAF3)	-0,718	8,21E-04
NP276891	Major histocompatibility complex class I antigen	-0,717	1,46E-02
NM_001098590	HPS6	-0,713	1,67E-02
TC568311	-	-0,709	2,17E-02
NM_001145222	RBP7	-0,706	6,93E-03
ENSSSCT00000031997	TMEM150C	-0,702	4,30E-03
TC553534	-	-0,698	5,64E-03
AK393873	-	-0,696	1,19E-02
NM_001078670	Interferon regulatory factor 9 (IRF9)	-0,692	2,98E-02
NM_213833	CTGF	-0,690	3,46E-02
ENSSSCT00000017183	interferon regulatory factor 2 (IRF2)	-0,688	2,36E-03
TC627807	LOC106504276	-0,688	7,37E-03
ENSSSCT00000013085	ABI3BP	-0,687	2,25E-05
ENSSSCT00000033382	NT5E	-0,685	3,61E-02
M36927	Gelsolin (GSN)	-0,683	4,64E-02
ENSSSCT00000035772	IFITM1	-0,683	8,38E-03
AK351081	-	-0,681	1,37E-02
ENSSSCT00000030135	KV channel interacting protein 4 (KCNIP4)	-0,678	2,23E-02
NM_001243420	cold shock domain containing C2, RNA binding (CSDC2)	-0,678	4,69E-03
TC626843	-	-0,677	1,54E-02
TC598894	-	-0,675	3,41E-02
ENSSSCT00000015281	GUK1	-0,672	1,35E-02
ENSSSCT00000015179	Arrestin domain containing 2 (ARRDC2)	-0,671	4,00E-03
TC551834	TNF alpha-induced protein 8-like protein 3	-0,669	1,14E-02
NM_001160426	ARF3	-0,669	1,63E-02
NM_001190235	RING1	-0,667	5,29E-05
ENSSSCT00000004600	Mitogen-activated protein kinase kinase kinase 5 (MEKK5)(MAP3K5)	-0,666	1,85E-02
TC595220	-	-0,665	2,58E-03
NM_001243379	GSTA4	-0,662	7,94E-05
NM_214323	Junction plakoglobin (JUP)	-0,662	1,20E-02
AK395885	ACOX2	-0,660	7,93E-04
NM_214190	THRA	-0,658	4,20E-03
A_72_P206912	-	-0,657	1,00E-02
TC560351	-	-0,655	3,45E-02
ENSSSCT00000000994	SOCS2	-0,654	5,64E-03
ENSSSCT00000011130	Mitogen-activated protein kinase kinase kinase 21 (MLK4)	-0,653	1,68E-03
AK233683	C8G	-0,653	3,62E-05
NM_001163406	PROCR	-0,652	8,11E-04
TC531203	-	-0,651	2,04E-02
TC568198	-	-0,650	1,31E-04
AK346234	LOC100520861	-0,649	2,26E-02
ENSSSCT00000014662	Complement factor D (adipsin) (CFD)	-0,647	7,15E-03
AK346233	LOC100515741	-0,645	6,89E-05
ENSSSCT00000026507	Myosin 14 heavy chain	-0,645	4,64E-02

TC559094	-	-0,645	6,02E-03
AK234441	LOC100153598	-0,644	8,25E-03
NM_214031	ENG	-0,640	4,93E-02
TC560803	-	-0,640	1,24E-03
NM_001287412	Nuclear receptor subfamily 1H4 (NR1H4)	-0,638	4,16E-02
TC605742	Acyl-CoA-binding protein (ACBP)	-0,637	3,93E-02
TC629646	-	-0,635	8,73E-03
ENSSSCT00000018583	FAXDC2	-0,633	1,67E-03
NM_001114278	stearoyl-CoA desaturase 5 (SCD5)	-0,633	2,92E-05
NM_001101028	Acyl-CoA Oxidase 1 (ACOX1)	-0,633	9,53E-03
TC605852	-	-0,632	4,75E-02
TC551076	Protein C12orf60 homolog	-0,629	4,37E-02
TC621752	Complement factor D precursor	-0,629	3,28E-03
NM_001190184	DnaJ (Hsp40) homolog, subfamily C, member 3 (DNAJC3)	-0,628	4,78E-03
A_72_P158981	Lipopolysaccharide-induced TNF factor (LITAF)	-0,627	3,79E-03
AK351647	LOC100624410	-0,626	2,43E-02
DN125588	PLN	-0,625	1,20E-02
ENSSSCT00000025705	Poly(ADP-ribose) polymerase family member 4 (PARP4)	-0,625	4,44E-04
ENSSSCT00000013380	SRPX	-0,619	4,50E-02
NM_001244410	Lipocalin 2 (LCN2)	-0,617	2,23E-02
ENSSSCT00000029598	phosphatidylinositol glycan anchor biosynthesis, class H (PIGH)	-0,617	1,86E-03
ENSSSCT00000006838	FAM110B	-0,615	4,52E-03
NM_214438	Caveolin-1 (CAV1)	-0,615	3,53E-02
NM_001097501	LGALS3	-0,612	4,00E-03
NM_001136511	HUS1	-0,609	6,36E-03
NM_001123104	Cathepsin Z (CTSZ)	-0,604	1,17E-02
TC555339	-	-0,604	9,84E-03
ENSSSCT00000035147	Estrogen receptor	-0,603	1,97E-02
NM_001244095	Atypical chemokine receptor 1 (ACKR1)	-0,600	1,47E-02
TC590063	-	-0,600	1,46E-02
TC555701	-	-0,599	6,01E-03
ENSSSCT00000030211	Limbic system-associated membrane protein	-0,599	3,60E-02
TC537652	-	-0,598	2,53E-02
DN114475	-	-0,598	9,39E-04
EW200629	-	-0,597	1,49E-03
ENSSSCT00000023788	COL6A2	-0,596	4,10E-03
ENSSSCT00000023179	RAS homolog family member U (RHOU)	-0,595	5,96E-03
NM_001044588	FBXO32	-0,594	1,59E-02
A_72_P100126	RAS-like. family 11. member B (RASL11B)	-0,594	1,40E-03
AK349146	PTG010010B01	-0,593	9,12E-03
NM_213882	EDN1	-0,591	6,20E-05
ENSSSCT00000012858	CLDN11	-0,591	2,57E-03
ENSSSCT00000013104	COL8A1	-0,587	2,16E-02
AY609637	LOC100513611	-0,587	2,98E-02
NM_001122984	MFGE8	-0,583	3,09E-02

BX666762	-	-0,583	5,02E-04
EW374337	-	-0,582	5,13E-04
ENSSSCT00000004577	CITED2	-0,579	6,14E-04
NM_214271	NTRK3	-0,579	2,39E-03
ENSSSCT00000035115	Biglycan (BGN)	-0,578	3,09E-02
ENSSSCT00000008136	PDX1 C-terminal inhibiting factor 1	-0,577	4,05E-02
ENSSSCT00000010582	KCTD9	-0,576	2,97E-05
TC562329	-	-0,575	3,71E-03
TC555476	-	-0,571	3,08E-03
NM_001244629	PLBD1	-0,571	1,20E-02
ENSSSCT00000034119	LOC100516891	-0,569	3,01E-05
NM_001011505	Kruppel-like factor 13 (KLF13)	-0,567	8,31E-04
ENSSSCT00000008691	MGRN1	-0,566	1,23E-03
TC529862	-	-0,565	1,17E-02
NM_001024696	TNFSF10	-0,564	4,23E-02
ENSSSCT00000007278	FAM63A	-0,564	6,37E-04
TC609321	-	-0,563	6,42E-04
AK348520	RPL14	-0,563	2,63E-02
AK349805	-	-0,562	3,82E-02
BX671826	-	-0,562	2,85E-02
NM_001243435	Adenosine A3 receptor (ADORA3)	-0,562	2,97E-02
NM_001123189	RAS homolog family member B (RHOB)	-0,562	4,75E-03
NM_213972	calpain 1 (CAPN1)	-0,558	3,57E-02
NM_213785	coagulation factor III (tissue factor) (F3)	-0,557	3,41E-02
NM_001110420	SLC7A2	-0,557	4,69E-03
NM_214024	PPP2R1A	-0,556	9,53E-03
TC593742	-	-0,553	9,28E-04
TC601173	Neural cell adhesion molecule 1 (NCAM1)	-0,552	1,61E-02
NM_001123217	ZNF217	-0,552	8,57E-05
XM_003129835	Exophilin 5 (EXPH5)	-0,551	3,51E-03
TC523688	Protein CutA homolog	-0,551	4,01E-05
NM_214440	HYAL2	-0,549	7,61E-03
TC536717	Protease. serine. 23 isoform 2	-0,548	4,37E-02
ENSSSCT00000030935	LOC100513467	-0,548	5,45E-03
NM_214086	CD34	-0,548	3,68E-03
TC533090	-	-0,546	1,65E-02
TC575521	-	-0,544	3,37E-02
ENSSSCT00000010225	MEDAG	-0,539	1,30E-02
ENSSSCT00000006411	EDF1	-0,538	3,41E-02
DN118139	RHOG	-0,538	1,02E-02
TC601929	-	-0,536	1,18E-02
NM_001008692	LMCD1	-0,533	2,98E-02
NM_001123194	SERPING1	-0,533	3,79E-02
TC596311	-	-0,531	1,54E-02
NM_001243362	Lactamase, beta (LACTB)	-0,531	2,75E-02

ENSSSCT00000004670	Tumor protein D52-like 1 (TPD52L1)	-0,529	1,59E-02
BP446680	-	-0,527	3,28E-03
NM_214424	CYP4A24	-0,527	1,62E-05
BX677249	-	-0,527	4,10E-02
DN120432	-	-0,526	4,05E-02
ENSSSCT00000003153	PLEKHF1	-0,526	4,37E-02
AK389245	SLC25A39	-0,525	7,15E-03
NM_001097478	RPL14	-0,524	2,00E-03
A_72_P345793	-	-0,520	6,14E-04
ENSSSCT00000014394	CTNND1	-0,520	1,07E-02
AK237396	SIK2	-0,518	1,31E-04
EW172935	-	-0,518	2,65E-02
AK392187	TMEM158	-0,517	1,76E-03
NM_001123131	ITM2C	-0,516	2,67E-03
ENSSSCT00000028241	SMAD specific E3 ubiquitin protein ligase 1	-0,516	2,71E-02
ENSSSCT00000002527	MTHFD1	-0,516	4,89E-02
ENSSSCT00000014828	STXBP2	-0,516	2,53E-02
ENSSSCT00000013437	CCDC120	-0,513	1,07E-02
TC520959	-	-0,513	1,04E-03
NM_213986	MYOC	-0,512	1,08E-02
TC565249	-	-0,512	9,23E-04
EW030444	-	-0,511	2,93E-02
EW221235	-	-0,509	3,09E-02
TC608215	-	-0,509	3,83E-03
NM_214010	ABCG2	-0,509	4,27E-02
ENSSSCT00000024719	USP2	-0,508	1,94E-03
AJ651239	-	-0,507	2,18E-02
ENSSSCT00000008629	ATP-binding cassette. sub-family C1 (CFTR/MRP).	-0,507	3,88E-03
ENSSSCT00000012379	KIAA1143	-0,506	1,59E-04
TC581405	Plexin A1 (PLXNA1)	-0,506	1,95E-02
AK392875	-	-0,504	2,15E-02
NM_001123193	AZIN2	-0,502	4,05E-02
AK391669	CITED2	-0,501	2,88E-02
TC563657	-	-0,501	4,05E-02
**WI+CS6h-CMJ versus WI-CMJ** (Up-regulated genes)			
ENSSSCT00000016913	TOR3A	2,945	4,32E-02
TC611155	-	2,798	2,25E-10
TC529750	-	2,443	1,47E-12
BF444493	-	2,411	1,19E-02
TC611786	-	2,121	3,31E-05
TC521842	-	1,607	2,13E-07
TC605691	V-set domain containing T cell activation inhibitor 1 (VTCN1)	1,581	2,12E-04
NM_214069	Fucosyltransferase 2 (FUT2)	1,498	4,47E-06
AK398294	Creatine kinase, M-type (CKM)	1,438	3,08E-02

TC552941	-	1,412	2,33E-15
NM_001130532	Par-6 family cell polarity regulator beta (PARD6B)	1,367	3,68E-04
TC554916	-	1,355	2,25E-02
NM_001185171	FAM204A	1,350	6,08E-04
ENSSSCT00000019353	PIPOX	1,345	2,32E-02
ENSSSCT00000012922	B-cell lymphoma 6 protein (BCL6)	1,294	7,83E-04
TC561142	-	1,285	3,08E-03
TC575360	-	1,263	5,19E-04
XR_001305510	LOC106508566	1,263	4,72E-04
TC570078	-	1,253	4,13E-07
NM_214105	Guanylate cyclase 2C (heat stable enterotoxin receptor) (GUCY2C)	1,216	6,85E-03
TC577359	-	1,209	3,08E-03
TC593464	-	1,155	8,39E-03
TC543988	-	1,089	3,30E-02
ENSSSCT00000031521	LY6/PLAUR domain containing 6 (LYPD6)	1,070	4,38E-04
AK231086	-	1,054	4,36E-02
TC556782	-	1,020	2,26E-03
NM_001113447	phytanoyl-CoA 2-hydroxylase (PHYH)	1,001	3,23E-08
TC617990	LOC106507828	0,998	3,03E-02
ENSSSCT00000024134	phytanoyl-CoA 2-hydroxylase (PHYH)	0,996	1,99E-06
ENSSSCT00000022578	Mucolipin 3 (MCOLN3)	0,984	6,36E-03
TC523104	-	0,954	3,62E-05
DY407595	-	0,946	7,73E-05
TC569507	-	0,939	5,58E-04
TC566133	-	0,932	8,19E-04
XM_001929359	ATP binding cassette subfamily C member 2 (ABCC2)	0,920	8,39E-07
ENSSSCT00000031509	Nestin (NES)	0,898	7,93E-04
AK398217	SFXN4	0,881	3,53E-02
NM_001004027	Heme oxygenase 1 (HMOX1)	0,879	2,96E-02
TC612492	-	0,842	2,98E-02
AJ660252	-	0,840	6,60E-04
AJ964590	-	0,833	2,34E-04
TC574741	-	0,829	2,13E-07
ENSSSCT00000014263	REST corepressor 2 (RCOR2)	0,827	1,92E-03
TC566441	Tripartite motif containing 4 (TRIM4)	0,826	9,56E-03
TC575922	-	0,817	1,85E-02
TC544492	-	0,804	7,43E-05
XM_003124543	amiloride-sensitive sodium channel subunit gamma-like (SCNN1G)	0,798	8,11E-03
TC523118	-	0,797	1,06E-04
ENSSSCT00000018037	striatin interacting protein 2 (STRIP2)	0,792	1,34E-02
NM_001146126	Patatin-like phospholipase domain containing 3 (PNPLA3)	0,788	7,82E-03
NM_001246253	Translation initiation factor 4 gamma 1 (EIF4G1)	0,786	1,88E-04
A_72_P322198	ZBTB44 (zinc finger and BTB domain containing 44)	0,784	4,33E-04
AK240605	LOC100512960	0,783	2,55E-03
AK397918	CCDC17	0,779	1,07E-02

TC584067	-	0,774	2,54E-02
TC597684	-	0,767	1,08E-03
TC608650	-	0,766	4,61E-02
AK400868	AZGP1	0,764	3,94E-02
A_72_P125346	SAP30-like (SAP30L)	0,735	1,66E-04
A_72_P773975	-	0,733	4,30E-02
TC600965	-	0,730	4,67E-03
ENSSSCT00000024917	K(lysine) acetyltransferase 2B (KAT2B)	0,728	3,45E-04
ENSSSCT00000002223	receptor-interacting serine-threonine kinase 3 (RIPK3)	0,726	3,51E-02
TC557923	-	0,724	6,15E-03
TC618296	CCAAT/enhancer binding protein (C/EBP) (CEBPA)	0,720	5,53E-05
ENSSSCT00000000169	PR domain containing 4	0,715	1,04E-03
ENSSSCT00000026371	ADAM metallopeptidase domain 9 (ADAM9)	0,711	1,18E-04
ENSSSCT00000030542	Ubiquitin carboxyl terminal hydrolase 10	0,709	1,44E-03
TC617432	-	0,707	6,60E-04
ENSSSCT00000012056	BAMBI	0,702	5,09E-03
TC621227	-	0,694	3,23E-02
TC601571	-	0,690	2,25E-03
TC533770	ENSP00000381892	0,690	4,03E-03
AF245504	CYP24A1 (25-hydroxyvitamin D3-24-hydroxylase)	0,686	2,01E-02
TC537380	-	0,684	7,73E-05
ENSSSCT00000011534	ATP binding cassette subfamily C member 2 (ABCC2)	0,682	4,48E-03
ENSSSCT00000010315	ZC3H13	0,681	9,47E-04
XM_001926022	pantothenate kinase 1 (PANK1)	0,680	2,16E-02
TC524227	-	0,671	2,98E-02
ENSSSCT00000009301	striatin, calmodulin binding protein (STRN)	0,670	1,19E-02
AK343979	LOC100153768. AMP010096E10 macrophages	0,662	1,83E-03
BX916072	-	0,661	1,58E-04
TC548138	-	0,661	2,98E-02
NM_001044551	Creatine kinase. mitochondrial 2 (CKMT2)	0,652	1,26E-03
AK345441	-	0,646	5,62E-05
TC594690	-	0,641	3,08E-03
DN123882	-	0,641	2,10E-04
ENSSSCT00000025080	TES010103A05	0,640	8,29E-03
ENSSSCT00000025080	CG2256-PB	0,640	8,29E-03
TC539874	-	0,639	1,58E-03
EW177256	-	0,630	1,01E-04
TC532985	-	0,628	2,24E-04
TC601667	-	0,627	2,23E-02
ENSSSCT00000019021	IGFBP4	0,623	1,37E-02
BX667964	-	0,619	3,25E-02
ENSSSCT00000018200	STK31	0,617	6,93E-03
TC612783	-	0,617	2,61E-03
TC525538	Dual specificity tyrosine phosphorylation regulated kinase 2 (DYRK2)	0,617	5,30E-05

ENSSSCT00000033656	CLDN4	0,615	4,53E-02
ENSSSCT00000005485	UACA	0,613	1,29E-03
AK400200	LOC102159136	0,610	1,42E-02
ENSSSCT00000025092	Thiosulfate sulfurtransferase	0,608	1,12E-02
TC555221	-	0,605	4,75E-02
NM_214352	ATPase. Ca++ transporting (ATP2B1)	0,605	2,61E-03
ENSSSCT00000017147	Myomesin (M-protein) 2	0,602	7,73E-05
EW350228	-	0,600	2,75E-02
TC519002	-	0,598	1,53E-03
ENSSSCT00000002909	Transmembrane protein 14A (TMEM14A)	0,597	2,63E-02
TC555671	-	0,593	2,81E-02
ENSSSCT00000012635	SET domain containing 5	0,592	5,23E-03
TC566120	-	0,592	3,20E-03
ENSSSCT00000023491	Histone H4	0,589	2,32E-03
ENSSSCT00000011079	Histone cell cycle regulator (HIRA)	0,583	2,58E-03
TC590226	-	0,580	1,61E-02
BX920940	-	0,579	5,07E-03
TC572838	-	0,572	2,33E-03
BX921275	-	0,570	1,77E-02
DY417616	-	0,569	1,04E-04
NM_001197194	Dicer 1. ribonuclease type III (DICER1)	0,567	7,06E-04
NM_214067	Calpastatin (CAST)	0,563	8,07E-03
NM_001258352	TACC2	0,561	3,91E-02
ENSSSCT00000026831	AMP010004F01 from macrophage	0,559	1,67E-02
ENSSSCT00000032295	Ninein (GSK3B interacting protein)	0,557	1,62E-03
ENSSSCT00000010279	Alpha-1,2-mannosyltransferase (ALG11)	0,553	6,52E-05
ENSSSCT00000009614	Replication factor c subunit 1 activator 1	0,553	5,13E-04
ENSSSCT00000005108	LEO1	0,552	4,37E-03
A_72_P058501	Ryanodine receptor 2 (cardiac) (RYR2)	0,550	1,63E-02
TC616529	-	0,548	9,84E-03
ENSSSCT00000008564	general transcription factor IIIC (GTF3C1)	0,546	5,66E-04
ENSSSCT00000009769	CENPC	0,546	1,62E-05
ENSSSCT00000002715	TRIP11	0,544	3,92E-05
TC555988	-	0,541	3,06E-02
TC625344	-	0,540	1,40E-03
ENSSSCT00000024571	Dmx-like 1	0,537	5,43E-03
TC588425	-	0,536	1,65E-04
NM_001244501	Glutamate dehydrogenase 1 (GLUD1)	0,536	1,47E-02
ENSSSCT00000000843	KIF21A	0,536	5,51E-04
TC529269	-	0,534	3,26E-02
BF194504	-	0,533	2,79E-04
XM_003132024	N-acetylglucosaminyl-phosphatidylinositol de-N-acetylase-like (PIGL)	0,532	2,70E-03
ENSSSCT00000011497	Tolloid-like 2 (TLL2)	0,532	1,64E-02
ENSSSCT00000024936	ZFP30 zinc finger protein	0,528	1,56E-06
TC523260	-	0,527	1,52E-02

TC518652	-	0,527	2,79E-04
TC565811	-	0,526	2,02E-02
A_72_P050011	-	0,525	2,57E-06
ENSSSCT00000013899	ATPase. class VI. type 11C	0,523	5,10E-03
TC556751	-	0,523	1,76E-02
TC595337	-	0,522	1,41E-02
ENSSSCT00000030857	CCDC47	0,518	8,39E-03
A_72_P160346	Protein tyrosine phosphatase. non-receptor 21 (PTPN21)	0,518	6,08E-04
TC572525	-	0,516	4,24E-05
XM_003131126	RAS-related protein Rab-40B-like	0,516	2,58E-02
ENSSSCT00000002090	S-phase cyclin A-associated protein in the ER	0,513	2,33E-03
TC556284	-	0,513	2,71E-02
ENSSSCT00000006300	DDX31	0,513	2,17E-02
TC531386	-	0,511	3,05E-02
BX669300	-	0,510	9,23E-04
TC597524	-	0,509	4,05E-02
A_72_P015946	DGCR8 (DGCR8 microprocessor complex subunit)	0,508	5,94E-06
TC532904	-	0,507	7,59E-04
EW341358	-	0,505	3,47E-02
BX916806	-	0,504	3,62E-05
ENSSSCT00000002571	PLEKHD1	0,504	1,41E-02
ENSSSCT00000018581	GEMIN5	0,503	1,34E-02
ENSSSCT00000007486	SARS	0,502	4,22E-02
NM_001160297	FBXO9	0,500	4,18E-03
**WI+CS24h-CMJ versus WI-CMJ** (Down-regulated genes)			
NM_001167835	Cytochrome P450 2C42 (CYP2C42)	-2,893	4,55E-03
NM_214417	CYP2A19	-2,749	9,64E-03
NM_213845	Uteroferrin associated basic protein-2 (UABP-2)	-2,677	3,92E-02
A_72_P443884	-	-2,650	4,32E-03
A_72_P054101	-	-2,646	1,18E-03
NM_001159306	Pyruvate dehydrogenase kinase isozyme 4, mitochondrial (PDK4)	-2,501	7,43E-05
ENSSSCT00000015537	Cysteine Dioxygenase Type 1 (CDO1)	-2,465	2,86E-08
NM_213850	Glutathione S-transferase alpha 2 (GSTA2)	-2,380	2,09E-03
TC613147	-	-2,352	1,13E-03
A_72_P773461	IGLV (IG lambda chain v region)	-2,018	3,28E-02
NM_214109	Chymotrypsin-like elastase family, member 2A (CELA2A)	-2,013	9,96E-03
TC534148	-	-1,941	3,27E-06
NM_214200	Perilipin 2 (PLIN2)	-1,794	8,45E-03
ENSSSCT00000029761	IgA C alpha	-1,789	6,35E-03
NM_001134824	CYP3A46	-1,754	3,86E-03
JX092267	Adiponectin, C1Q And Collagen Domain Containing (ADIPOQ)	-1,667	3,09E-03
KP735782	Isolate S1-V1 glutathione S-transferase A2 (GSTA2)	-1,657	4,59E-03
NM_001078665	Chemokine ligand 26-like (CCL26) (MIP-4a, eotaxin-3)	-1,623	4,29E-07
ENSSSCT00000026328	Vitamin D binding protein	-1,618	3,43E-02

NM_213962	Hydroxyacyl-CoA dehydrogenase trifunctional multienzyme complex subunit alpha (HADHA)	-1,474	8,31E-05
EF601160	Adiponectin, C1Q And Collagen Domain Containing (ADIPOQ)	-1,412	3,59E-03
NM_214422	CYP3A39	-1,396	2,18E-02
NM_001044592	Coagulation factor X (F10)	-1,382	3,84E-04
NM_001038644	Angiopoietin like 4 (ANGPTL4)	-1,375	3,16E-02
NM_214134	Erythropoietin (EPO)	-1,309	1,56E-02
TC544200	-	-1,286	1,47E-02
NM_001122985	Coagulation factor II (thrombin) (F2)	-1,276	1,32E-02
NM_213988	SRD5A2	-1,260	3,95E-02
AK236120	CALHM2	-1,246	2,92E-03
NM_001161753	Phosphoenolpyruvate carboxykinase 2 (mitochondrial) (PCK2)	-1,241	2,69E-02
TC566172	-	-1,230	1,95E-04
NM_001163696	C13H21orf62	-1,219	1,05E-03
NM_001113438	AQP7	-1,214	6,52E-03
NM_001114056	MHC class I antigen 5 (SLA-5)	-1,192	8,76E-04
NM_214425	CYP4A21	-1,191	1,04E-08
NM_001083941	ACE	-1,179	4,36E-03
AK232564	-	-1,177	2,34E-03
AK345473	-	-1,176	1,33E-03
TC596995	-	-1,175	1,86E-05
AK238381	-	-1,165	6,40E-03
A_72_P376423	-	-1,151	1,13E-03
EW262869	-	-1,142	2,73E-05
TC548575	-	-1,125	3,05E-03
TC587527	-	-1,114	9,82E-03
TC569278	-	-1,112	9,14E-04
NM_001097452	Complement component 8 (C8G)	-1,111	1,72E-05
EV864657	COL8A1	-1,094	6,70E-03
NM_214246	Carboxylesterase 1 (CES1)	-1,091	8,05E-03
NM_001315766	Carboxylesterase	-1,073	5,61E-03
AK392845	RNASET2	-1,068	6,85E-07
NM_001101027	CYP39A1	-1,067	6,47E-07
TC571068	-	-1,065	1,86E-05
AK235420	-	-1,059	3,86E-03
NM_214228	prostaglandin D2 synthase (PTGDS)	-1,057	1,55E-05
TC598771	-	-1,042	3,60E-04
NM_001122990	Neuronatin (NNAT)	-1,030	2,20E-04
TC604056	-	-1,026	4,07E-03
ENSSSCT00000035428	Arginase 2 (ARG2)	-1,020	1,37E-04
ENSSSCT00000035093	ACE	-1,012	4,16E-03
NM_001130243	troponin C type 1 (TNNC1)	-1,009	1,66E-02
TC541539	-	-1,005	7,21E-03
NM_001246243	Carnitine palmitoyltransferase 2 (CPT2)	-1,005	1,14E-04

TC590364	-	-0,998	1,05E-03
NM_001098605	PNPLA2	-0,988	2,28E-03
NM_001185143	SLA-DOA	-0,980	5,38E-06
NM_001315568	Glutathione S-transferase theta 1 (GSTT1)	-0,975	3,18E-02
ENSSSCT00000004450	RNASET2	-0,957	3,08E-05
NM_214337	protein phosphatase 1, regulatory (inhibitor) subunit 14A (PPP1R14A)	-0,949	1,30E-02
FD604545	-	-0,934	6,64E-03
ENSSSCT00000006875	CCAAT/enhancer binding protein (C/EBP), delta (CEBPD)	-0,934	1,39E-06
ENSSSCT00000003289	Actinin, alpha 4 (ACTN4)	-0,931	6,40E-03
TC601797	Mitochondrial Carnitine O-palmitoyltransferase 2	-0,925	6,64E-04
BX914375	-	-0,925	2,38E-04
TC600327	-	-0,924	4,67E-03
CV871207	-	-0,916	1,04E-03
NM_001243452	DNA-damage-inducible transcript 4 (DDIT4)	-0,915	1,06E-03
AK393941	ABL2	-0,913	3,43E-02
TC595529	MGC148805 protein	-0,895	1,84E-02
ENSSSCT00000010309	Leucine Rich Repeats And Calponin Homology Domain Containing 1 (LRCH1)	-0,881	2,17E-02
TC536661	-	-0,879	1,71E-04
NM_001044573	Angiogenin. ribonuclease. RNase A family. 5 (ANG)	-0,876	3,13E-02
A_72_P297874	Dysbindin domain containing 1 (DBNDD1)	-0,870	9,39E-04
NM_001048187	DLK1	-0,867	6,05E-06
ENSSSCT00000009671	RASL11B	-0,864	2,67E-03
BF713827	-	-0,864	2,46E-02
ENSSSCT00000001838	Peroxisomal biogenesis factor 6 (PEX6)	-0,858	2,27E-02
A_72_P705517	-	-0,855	2,01E-02
ENSSSCT00000006766	Peptidase inhibitor 15 (PI15)	-0,845	9,95E-03
NM_001143719	MAP kinase interacting serine/threonine kinase 1 (MKNK1)	-0,845	6,09E-05
XR_001309264	LOC102158887	-0,845	8,22E-04
NM_214153	ENTPD1	-0,843	1,05E-04
NM_001134349	Kruppel-like factor 15 (KLF15)	-0,832	3,10E-05
NM_001243420	cold shock domain containing C2, RNA binding (CSDC2)	-0,830	5,40E-04
NM_001110172	Aquaporine 3 (AQP3)	-0,823	2,14E-03
ENSSSCT00000016639	FMOD	-0,819	4,51E-03
NM_001243304	CYP27A1	-0,819	1,07E-03
ENSSSCT00000028877	Aminoacylproline aminopeptidase prolidase	-0,818	4,04E-04
AK389171	GRAMD1B	-0,818	2,62E-02
TC610430	-	-0,817	2,80E-02
TC533634	-	-0,815	8,51E-07
ENSSSCT00000002764	LOC100153899	-0,813	2,40E-06
TC560045	Dephospho-CoA kinase domain-containing protein	-0,813	2,52E-02
NM_001244729	C3H2orf40	-0,812	5,28E-03
AK345001	MX2	-0,812	3,66E-02
AK343323	-	-0,809	9,02E-03
AK348497	-	-0,807	3,08E-02

CA779378	-	-0,805	2,51E-02
NM_213932	LGALS9	-0,804	3,80E-02
ENSSSCT00000015179	Arrestin domain containing 2 (ARRDC2)	-0,797	6,75E-04
AK395885	ACOX2	-0,793	7,43E-05
ENSSSCT00000019531	Acyl-CoA dehydrogenase very long chain (ACADVL)	-0,787	6,35E-03
AK343540	-	-0,787	3,64E-02
TC528319	-	-0,776	4,66E-02
NM_001101028	Acyl-CoA Oxidase 1 (ACOX1)	-0,771	1,32E-03
ENSSSCT00000006282	Laminin. gamma 3	-0,768	2,70E-02
ENSSSCT00000007615	WDR63	-0,766	2,27E-02
EW034563	-	-0,765	4,07E-03
TC627807	LOC106504276	-0,761	2,82E-03
TC531203	-	-0,758	5,82E-03
TC623045	-	-0,752	1,44E-02
NM_001164652	Aggrecan (ACAN)	-0,751	2,73E-02
TC531848	-	-0,751	2,43E-04
ENSSSCT00000010983	PIK3IP1	-0,750	4,42E-03
ENSSSCT00000011130	Mitogen-activated protein kinase kinase kinase 21 (MLK4)	-0,747	3,54E-04
AK233683	C8G	-0,738	4,43E-06
NM_001123135	Perilipin 5 (PLIN5)	-0,734	3,09E-02
NP276891	Major histocompatibility complex class I antigen	-0,734	1,28E-02
CF365587	-	-0,734	2,07E-02
ENSSSCT00000017924	ZYX	-0,729	1,10E-02
NM_001123104	Cathepsin Z (CTSZ)	-0,725	2,10E-03
TC553534	-	-0,714	4,89E-03
NM_001190235	RING1	-0,712	1,77E-05
ENSSSCT00000000040	Cytochrome b5 reductase 3	-0,710	1,83E-02
NM_001097506	AN1-type zinc finger protein 5 (ZFAND5)	-0,708	8,47E-05
TC559094	-	-0,705	2,61E-03
TC595220	-	-0,704	1,48E-03
AK351081	-	-0,704	1,08E-02
AK346234	LOC100520861	-0,702	1,33E-02
ENSSSCT00000012586	Succinate-CoA ligase. GDP-forming. beta subunit	-0,702	3,54E-04
ENSSSCT00000013085	ABI family member 3 binding protein	-0,700	1,40E-05
TC629646	-	-0,699	3,62E-03
NM_001243362	Lactamase, beta (LACTB)	-0,699	2,61E-03
NM_001190184	DnaJ (Hsp40) homolog, subfamily C, member 3 (DNAJC3)	-0,695	1,83E-03
TC568621	-	-0,693	7,62E-03
TC560156	-	-0,679	9,39E-05
ENSSSCT00000004600	Mitogen-activated protein kinase kinase kinase 5 (MEKK5)(MAP3K5)	-0,676	1,72E-02
ENSSSCT00000029598	phosphatidylinositol glycan anchor biosynthesis, class H (PIGH)	-0,675	7,37E-04
AK346233	LOC100515741	-0,673	3,08E-05
NM_214031	Endoglin (ENG)	-0,671	3,69E-02
ENSSSCT00000023435	TNF receptor associated factor 3 (TRAF3)	-0,670	1,85E-03

DN114475	-	-0,670	2,38E-04
NM_001204382	Proline rich 5 (renal) (PRR5)	-0,669	1,98E-02
TC568311	-	-0,668	3,32E-02
ENSSSCT00000030135	KV channel interacting protein 4 (KCNIP4)	-0,667	2,57E-02
ENSSSCT00000008691	MGRN1	-0,667	1,65E-04
AK234441	LOC100153598	-0,664	6,40E-03
ENSSSCT00000015281	GUK1	-0,663	1,55E-02
ENSSSCT00000000715	Ovostatin precursor	-0,661	3,51E-02
ENSSSCT00000011739	Carboxypeptidase X (M14 family)	-0,661	4,02E-02
ENSSSCT00000005761	KIAA0020	-0,655	2,69E-02
NM_214155	CD247	-0,651	2,70E-02
TC555701	-	-0,651	2,82E-03
NM_214424	CYP4A24	-0,650	4,29E-07
NM_001243379	GSTA4	-0,648	1,05E-04
ENSSSCT00000012072	Cubilin (intrinsic factor-cobalamin receptor)	-0,647	4,36E-02
NM_214190	THRA	-0,646	5,31E-03
NM_001287412	Nuclear receptor subfamily 1H4 (NR1H4)	-0,645	3,91E-02
TC555476	-	-0,644	9,01E-04
TC555339	-	-0,643	5,68E-03
ENSSSCT00000007254	Tuftelin 1 (TUFT1)	-0,642	8,18E-03
ENSSSCT00000035115	Biglycan (BGN)	-0,642	1,50E-02
TC626843	-	-0,641	2,42E-02
ENSSSCT00000014828	STXBP2	-0,639	4,42E-03
NM_214385	prostaglandin reductase 1 (PTGR1)	-0,637	1,76E-02
NM_214119	DBI	-0,637	3,71E-02
NM_001098590	HPS6	-0,635	3,86E-02
ENSSSCT00000025705	Poly(ADP-ribose) polymerase family member 4 (PARP4)	-0,632	3,54E-04
NM_001160426	ARF3	-0,630	2,63E-02
TC581405	Plexin A1 (PLXNA1)	-0,625	3,04E-03
AK348520	RPL14	-0,624	1,27E-02
TC543804	-	-0,622	5,28E-03
NM_001195115	SLC25A27	-0,621	1,96E-02
ENSSSCT00000000994	SOCS2	-0,620	9,73E-03
NM_214323	Junction plakoglobin (JUP)	-0,620	2,07E-02
ENSSSCT00000003153	PLEKHF1	-0,617	1,44E-02
ENSSSCT00000030935	LOC100513467	-0,616	1,76E-03
ENSSSCT00000017183	Interferon regulatory factor 2 (IRF2)	-0,615	7,60E-03
ENSSSCT00000008136	PDX1 C-terminal inhibiting factor 1	-0,612	2,73E-02
AK393873	-	-0,609	3,27E-02
NM_001184895	PMM1	-0,607	3,10E-02
NM_001145222	RBP7	-0,604	2,51E-02
NM_214440	HYAL2	-0,604	3,15E-03
NM_001163406	PROCR	-0,603	2,03E-03
NM_001024696	TNFSF10	-0,601	2,78E-02
ENSSSCT00000031997	TMEM150C	-0,600	1,79E-02

TC525635	-	-0,600	3,67E-02
ENSSSCT00000002527	MTHFD1	-0,599	1,80E-02
NM_001123189	RAS homolog family member B (RHOB)	-0,599	2,61E-03
AJ961798	-	-0,598	7,06E-04
ENSSSCT00000035772	IFITM1	-0,598	2,47E-02
TC568198	-	-0,596	3,91E-04
EW374337	-	-0,592	3,86E-04
A_72_P345793	-	-0,591	1,14E-04
ENSSSCT00000025029	GLTSCR2	-0,591	4,76E-02
BP446680	-	-0,587	1,07E-03
CJ029355	-	-0,583	2,66E-02
NM_213972	calpain 1 (CAPN1)	-0,582	2,73E-02
TC537652	-	-0,581	3,17E-02
TC548784	-	-0,581	4,58E-02
ENSSSCT00000034119	Family with sequence similarity 122C	-0,578	1,89E-05
ENSSSCT00000013104	COL8A1	-0,577	2,51E-02
ENSSSCT00000014662	CFD	-0,576	1,93E-02
NM_001011505	KLF13	-0,575	7,57E-04
AK349146	PTG010010B01	-0,572	1,29E-02
ENSSSCT00000006838	FAM110B	-0,570	9,73E-03
ENSSSCT00000018583	FAXDC2	-0,566	5,47E-03
AK398132	-	-0,563	8,40E-04
ENSSSCT00000028241	SMAD specific E3 ubiquitin protein ligase 1	-0,562	1,49E-02
NM_001136511	HUS1	-0,561	1,39E-02
EW172935	-	-0,558	1,60E-02
NM_001244629	PLBD1	-0,556	1,53E-02
TC529862	-	-0,555	1,41E-02
TC609321	-	-0,555	8,17E-04
ENSSSCT00000012858	CLDN11	-0,554	5,24E-03
AK349805	-	-0,554	4,23E-02
NM_001243435	Adenosine A3 receptor (ADORA3)	-0,553	3,32E-02
TC523544	Mitochondrial 39S ribosomal protein L49	-0,552	1,26E-02
TC563657	-	-0,549	2,17E-02
NM_001123217	ZNF217	-0,547	9,39E-05
NM_001122984	MFGE8	-0,547	4,85E-02
DN120432	-	-0,546	3,19E-02
ENSSSCT00000017757	Ubiquitin specific peptidase 40	-0,542	2,09E-02
TC621752	Complement factor D precursor	-0,541	1,42E-02
TC612381	Poly(RC)-binding protein 2 isoform b variant	-0,539	2,94E-02
A_72_P206912	-	-0,537	4,43E-02
NM_001244412	Heme oxygenase 2 (HMOX2)	-0,535	8,78E-03
TC601929	-	-0,533	1,29E-02
TC629544	-	-0,533	4,48E-02
NM_213882	Endothelin 1 (EDN1)	-0,533	2,34E-04
ENSSSCT00000023788	COL6A2	-0,532	1,25E-02

ENSSSCT00000004577	CITED2	-0,530	1,66E-03
AK396814	WDFY3	-0,528	1,98E-02
AK391669	CITED2	-0,528	2,05E-02
ENSSSCT00000007278	FAM63A	-0,527	1,38E-03
TC571424	-	-0,527	3,44E-06
NM_214154	TXNRD1	-0,527	1,44E-02
NM_001110420	SLC7A2	-0,526	8,47E-03
TC546925	-	-0,525	5,12E-03
NM_001195399	BMP2	-0,525	2,91E-02
ENSSSCT00000024719	Ubiquitin specific peptidase 2 (USP2)	-0,523	1,46E-03
DN118139	RHOG	-0,523	1,34E-02
TC618430	NCEH1 (neutral cholesterol ester hydrolase 1)	-0,523	3,03E-02
A_72_P100126	RASL11B	-0,522	5,47E-03
TC548865	-	-0,520	2,80E-02
NM_214024	PPP2R1A	-0,520	1,72E-02
TC590063	-	-0,519	4,14E-02
ENSSSCT00000032777	Guanine nucleotide exchange factor 3 (VAV3)	-0,519	1,73E-02
BX666762	-	-0,516	1,94E-03
AK235264	MLYCD	-0,516	1,26E-02
BX677249	-	-0,516	4,74E-02
ENSSSCT00000010582	KCTD9	-0,516	1,15E-04
CX064162	-	-0,514	1,40E-02
NM_001114278	stearoyl-CoA desaturase 5 (SCD5)	-0,513	4,35E-04
ENSSSCT00000008629	ATP-binding cassette. sub-family C1 (CFTR/MRP)	-0,510	3,93E-03
NM_001123222	NAGA	-0,509	1,18E-02
A_72_P158981	LITAF (lipopolysaccharide induced TNF factor)	-0,509	2,37E-02
TC520959	-	-0,508	1,18E-03
NM_001044588	FBXO32	-0,507	4,93E-02
TC565249	-	-0,507	1,06E-03
DB804246	-	-0,506	1,75E-03
XM_001928852	PXDC1	-0,506	4,14E-02
ENSSSCT00000010225	MEDAG	-0,506	2,17E-02
TC523688	Protein CutA homolog	-0,505	1,22E-04
ENSSSCT00000003430	IRF2BP1	-0,502	2,51E-02
TC618316	-	-0,502	1,76E-02
EW200629	-	-0,502	8,79E-03
NM_214064	FMO1	-0,501	2,33E-03
ENSSSCT00000031688	ZMAT3	-0,501	4,48E-02
TC547188	-	-0,501	1,93E-05
AK392875	-	-0,500	2,33E-02
A_72_P320493	-	-0,500	2,89E-04
**WI+CS24h-CMJ versus WI-CMJ** (Up-regulated genes)			
TC611155	-	2,944	5,29E-11
TC529750	-	2,501	7,34E-13

BF444493	-	2,338	1,60E-02
TC611786	-	1,982	7,66E-05
TC605691	V-set domain containing T cell activation inhibitor 1 (VTCN1)	1,523	3,45E-04
NM_214069	Fucosyltransferase 2 (FUT2)	1,514	3,39E-06
AK398294	Creatine kinase, M-type (CKM)	1,507	2,28E-02
TC521842	-	1,485	6,85E-07
TC552941	-	1,361	7,45E-15
TC561142	-	1,304	2,83E-03
ENSSSCT00000012922	B-cell lymphoma 6 protein (BCL6)	1,290	8,50E-04
NM_001185171	FAM204A	1,282	1,06E-03
TC577359	-	1,265	2,07E-03
TC575360	-	1,259	5,12E-04
TC554916	-	1,258	3,84E-02
NM_001130532	Par-6 family cell polarity regulator beta (PARD6B)	1,248	1,05E-03
AK237109	LOC100627089	1,218	1,31E-02
TC569507	-	1,204	1,72E-05
NM_214105	Guanylate cyclase 2C (heat stable enterotoxin receptor) (GUCY2C)	1,184	9,18E-03
XR_001305510	LOC106508566	1,128	1,66E-03
TC556782	-	1,118	8,76E-04
TC570078	-	1,107	3,49E-06
ENSSSCT00000031521	LY6/PLAUR domain containing 6 (LYPD6)	1,097	2,85E-04
TC543988	-	1,093	3,27E-02
NM_001190276	nuclear receptor subfamily 4, group A, member 2 (NR4A2)	1,067	3,09E-02
TC593464	-	1,013	2,45E-02
NM_001129949	CKM	1,004	3,58E-02
NM_001113447	phytanoyl-CoA 2-hydroxylase (PHYH)	0,989	3,68E-08
AK398217	SFXN4	0,981	1,72E-02
ENSSSCT00000024134	phytanoyl-CoA 2-hydroxylase (PHYH)	0,974	2,91E-06
DY407595	-	0,952	6,51E-05
ENSSSCT00000031509	Nestin (NES)	0,927	5,48E-04
XM_003124543	amiloride-sensitive sodium channel subunit gamma-like (SCNN1G)	0,918	2,10E-03
XM_001929359	ATP binding cassette subfamily C member 2 (ABCC2)	0,911	8,51E-07
TC612492	-	0,887	2,12E-02
TC523118	-	0,879	2,07E-05
TC548959	-	0,879	3,92E-02
ENSSSCT00000014263	REST corepressor 2 (RCOR2)	0,876	1,06E-03
AJ660252	-	0,868	4,46E-04
ENSSSCT00000022578	Mucolipin 3 (MCOLN3)	0,848	2,27E-02
A_72_P322198	ZBTB44 (zinc finger and BTB domain containing 44)	0,843	1,53E-04
TC521714	-	0,836	2,97E-02
TC523777	-	0,833	3,53E-02
TC523104	-	0,826	2,41E-04
TC566133	-	0,816	3,62E-03
TC608650	-	0,809	3,27E-02
TC566441	Tripartite motif containing 4 (TRIM4)	0,793	1,40E-02

TC617432	-	0,788	1,65E-04
AK231785	AP1S3	0,783	4,43E-02
ENSSSCT00000012056	BAMBI	0,779	1,86E-03
A_72_P773975	-	0,773	3,08E-02
ENSSSCT00000033656	CLDN4	0,770	9,02E-03
TC574741	-	0,762	6,85E-07
AK240605	LOC100512960	0,762	3,62E-03
NM_001038694	Acyl-CoA synthetase long-chain family member 4 (ACSL4)	0,753	4,24E-02
TC537380	-	0,742	1,94E-05
TC546352	-	0,742	3,29E-02
AJ964590	-	0,740	1,04E-03
TC544492	-	0,727	2,54E-04
TC618296	CCAAT/enhancer binding protein (C/EBP) (CEBPA)	0,723	4,40E-05
EW350228	-	0,719	6,50E-03
TC601571	-	0,715	1,60E-03
AK400200	LOC102159136	0,712	3,62E-03
ENSSSCT00000026371	ADAM metallopeptidase domain 9 (ADAM9)	0,706	1,19E-04
TC533770	ENSP00000381892	0,702	3,62E-03
ENSSSCT00000024917	K(lysine) acetyltransferase 2B (KAT2B)	0,702	5,02E-04
NM_001146126	Patatin-like phospholipase domain containing 3 (PNPLA3)	0,702	2,08E-02
ENSSSCT00000010315	ZC3H13	0,699	7,48E-04
BX667964	-	0,697	1,43E-02
TC597684	-	0,696	3,22E-03
A_72_P125346	SAP30-like (SAP30L)	0,690	3,75E-04
ENSSSCT00000017407	HOXD1	0,688	1,25E-02
ENSSSCT00000002909	Transmembrane protein 14A (TMEM14A)	0,686	9,23E-03
TC573765	-	0,679	1,82E-02
AK397918	CCDC17	0,670	3,32E-02
TC586066	-	0,665	1,24E-04
ENSSSCT00000009301	striatin, calmodulin binding protein (STRN)	0,656	1,47E-02
XM_001926022	pantothenate kinase 1 (PANK1)	0,653	2,95E-02
ENSSSCT00000032295	Ninein (GSK3B interacting protein)	0,652	2,41E-04
TC524227	-	0,651	3,71E-02
NM_001258352	TACC2	0,648	1,42E-02
ENSSSCT00000017147	Myomesin (M-protein) 2	0,636	2,95E-05
TC548138	-	0,632	4,08E-02
ENSSSCT00000023491	Histone H4	0,628	1,20E-03
TC555671	-	0,627	1,92E-02
TC590749	-	0,617	1,31E-03
TC532985	-	0,616	2,75E-04
ENSSSCT00000025080	E3 Ubiquitin ligase DTX2	0,615	1,23E-02
EW177256	-	0,612	1,39E-04
TC557923	-	0,607	2,66E-02
TC600965	-	0,606	2,34E-02
NM_001044551	CKMT2	0,601	3,09E-03

ENSSSCT00000012635	SET domain containing 5	0,596	5,23E-03
A_72_P218807	-	0,595	4,69E-02
TC529900	-	0,595	3,37E-02
TC594690	-	0,594	6,89E-03
ENSSSCT00000018200	STK31	0,593	1,04E-02
DN123882	-	0,588	6,35E-04
NM_001243656	HIST1H2BD	0,586	1,56E-05
ENSSSCT00000006169	SLC2A8	0,586	2,46E-02
ENSSSCT00000009295	CCAAT/enhancer binding protein (C/EBP), zeta (CEBPZ)	0,584	3,38E-02
ENSSSCT00000032409	SRP19	0,579	6,39E-03
NM_001246253	EIF4G1	0,575	6,40E-03
ENSSSCT00000034652	Immediate Early Response 3 (IER3)	0,574	9,01E-03
TC550342	Purinergic receptor P2Y. G-protein coupled. 1 (P2RY1)	0,574	4,83E-04
A_72_P050011	-	0,572	6,47E-07
ENSSSCT00000024936	ZFP30 zinc finger protein	0,570	4,29E-07
TC532019	-	0,570	4,42E-02
ENSSSCT00000008564	general transcription factor IIIC (GTF3C1)	0,569	3,19E-04
DY417616	-	0,567	1,05E-04
TC612783	-	0,566	6,49E-03
TC539874	-	0,566	5,67E-03
A_72_P247197	LOC102158270	0,564	2,01E-04
NM_214067	Calpastatin (CAST)	0,564	8,16E-03
AK351387	-	0,564	1,14E-04
TC519002	-	0,562	3,09E-03
NM_001037965	Inhibitor of DNA binding 2 (ID2)	0,558	2,73E-02
TC525538	Dual specificity tyrosine phosphorylation regulated kinase 2 (DYRK2)	0,557	2,00E-04
AK343979	LOC100153768	0,556	1,06E-02
NM_214352	ATPase. Ca++ transporting (ATP2B1)	0,551	7,02E-03
AK345441	-	0,550	4,47E-04
TC590226	-	0,549	2,48E-02
BX920940	-	0,546	9,18E-03
ENSSSCT00000009614	Replication factor c subunit 1 activator 1	0,546	5,68E-04
A_72_P443589	HISTONE H4	0,545	2,72E-03
DN132993	-	0,545	4,87E-03
XM_003133082	Zinc finger protein 503-like	0,544	2,34E-04
ENSSSCT00000001263	Histone cluster 1. H2bh	0,544	1,86E-05
NM_214339	DNAJA4	0,543	7,47E-04
ENSSSCT00000011534	ATP binding cassette subfamily C member 2 (ABCC2)	0,541	3,09E-02
XM_003132024	N-acetylglucosaminyl-phosphatidylinositol de-N-acetylase-like (PIGL)	0,540	2,55E-03
ENSSSCT00000025092	Thiosulfate sulfurtransferase	0,540	2,74E-02
ENSSSCT00000004227	USP1	0,538	1,21E-03
ENSSSCT00000010405	Dopachrome tautomerase (DCT)	0,534	5,28E-03
TC586031	LOC522928 protein	0,533	2,40E-02
ENSSSCT00000003857	EPH receptor A2 (EPHA2)	0,533	3,80E-02

ENSSSCT00000005485	UACA	0,533	5,73E-03
TC597524	-	0,528	3,19E-02
ENSSSCT00000026137	Histone cluster 1. H2bo	0,528	2,28E-03
ENSSSCT00000030542	SPLT10089G01	0,526	2,31E-02
ENSSSCT00000009769	CENPC	0,526	2,07E-05
ENSSSCT00000000169	PR domain containing 4	0,524	2,08E-02
BX916072	-	0,524	2,50E-03
AK233798	-	0,519	2,22E-04
TC572525	-	0,517	3,39E-05
XM_003131126	RAS-related protein Rab-40B-like	0,517	2,58E-02
ENSSSCT00000029351	Topoisomerase (DNA) I (TOP1)	0,517	2,45E-04
ENSSSCT00000012034	MGC152506 protein	0,516	2,23E-02
A_72_P015946	DGCR8 (DGCR8 microprocessor complex subunit)	0,514	4,43E-06
ENSSSCT00000002571	PLEKHD1	0,513	1,28E-02
TC565811	-	0,513	2,46E-02
NM_001128492	Cellular retinoic acid binding protein 1 (CRABP1)	0,511	2,34E-02
AK348278	LOC100628052	0,510	4,35E-02
TC621005	-	0,506	2,97E-02
BX669300	-	0,505	1,06E-03
TC566120	-	0,504	1,49E-02
ENSSSCT00000004730	Regulating synaptic membrane exocytosis 1	0,503	2,82E-05
A_72_P058501	-	0,503	3,23E-02
ENSSSCT00000002715	TRIP11	0,501	1,15E-04
ENSSSCT00000019100	ZNF652	0,501	1,84E-02

Table S3: Functionnal Enrichment analysis of Gene-Ontology Biological-Process terms for differentially expressed (DE) genes in cortex tissues.

The table provided represents the enrichment analysis of Gene Ontology Biological Process (GOBP) terms for differentially expressed genes up and down-regulated in cortex compared conditions. The list of regulated genes, where genes without Entrez Gene ID have been removed, was used to perform the hypergeometrical statistical test, using GOstats package. (GOBPID: Gene Ontology Biological Process Identification number; p value: p value given by the hypergeometric test ($p<0.05$); Term: Gene Ontology Biological Process description term; and differentially expressed genes in each family: Name of gene differentially expressed in the microarray, included in the Gene Ontology family.)

GOBP-ID	p value	Terms	differentially expressed genes in each family
**WI-C versus Ctl-C** (Down-regulated genes)			
GO:1901029	0.0003	negative regulation of mitochondrial membrane permeabilization (apoptotisis)	IER3 (immediate early response 3)
GO:0045820	0.0009	negative regulation of glycolytic process	IER3
GO:0051195	0.0011	negative regulation of cofactor metabolic process	IER3
GO:0045978	0.0014	negative regulation of nucleoside metabolic process	IER3
GO:0007095	0.0017	mitotic G2 DNA damage checkpoint	IER3
GO:1902110	0.0017	positive regulation of mitochondrial membrane permeability involved in apoptosis	IER3
GO:0001562	0.0020	response to protozoan	IER3
GO:0010972	0.0020	negative regulation of G2/M transition of mitotic cell cycle	IER3
GO:0030811	0.0023	regulation of nucleotide catabolic process	IER3
GO:0035794	0.0023	positive regulation of mitochondrial membrane permeability	IER3
GO:0003085	0.0026	negative regulation of systemic arterial blood pressure	IER3

GO:1900543	0.0026	negative regulation of purine nucleotide metabolic process	IER3
GO:0006282	0.0031	regulation of DNA repair	IER3
GO:0043471	0.0031	regulation of cellular carbohydrate catabolic process	IER3
GO:0051196	0.0031	regulation of coenzyme metabolic process	IER3
GO:0090559	0.0031	regulation of membrane permeability	IER3
GO:1902749	0.0031	regulation of cell cycle G2/M phase transition	IER3
GO:0045912	0.0034	negative regulation of carbohydrate metabolic process	IER3
GO:0007006	0.0037	mitochondrial membrane organization	IER3
GO:1903578	0.0037	regulation of ATP metabolic process	IER3
GO:0044774	0.0040	mitotic DNA integrity checkpoint	IER3
GO:1901292	0.0040	nucleoside phosphate catabolic process	IER3
GO:0043467	0.0048	regulation of generation of precursor metabolites and energy	IER3
GO:0006757	0.0054	ATP generation from ADP	IER3
GO:1901988	0.0054	negative regulation of cell cycle phase transition	IER3
GO:0008637	0.0057	apoptotic mitochondrial changes	IER3
GO:0046939	0.0057	nucleotide phosphorylation	IER3
GO:0008630	0.0062	intrinsic apoptotic signaling pathway in response to DNA damage	IER3
GO:0050728	0.0062	negative regulation of inflammatory response	IER3
GO:0009179	0.0065	purine ribonucleoside diphosphate metabolic process	IER3
GO:0009132	0.0068	nucleoside diphosphate metabolic process	IER3
GO:0000077	0.0071	DNA damage checkpoint	IER3
GO:0006090	0.0071	pyruvate metabolic process	IER3
GO:0046496	0.0071	nicotinamide nucleotide metabolic process	IER3
GO:0072524	0.0074	pyridine-containing compound metabolic process	IER3
GO:0010821	0.0077	regulation of mitochondrion organization	IER3
GO:0044724	0.0077	single-organism carbohydrate catabolic process	IER3
GO:0006733	0.0082	oxidoreduction coenzyme metabolic process	IER3
GO:2000377	0.0096	regulation of reactive oxygen species metabolic process	IER3
GO:0045930	0.0099	negative regulation of mitotic cell cycle	IER3
**WI-C versus Ctl-C** (Up- regulated genes)			

GO:0006927	0.0026	transformed cell apoptotic process	RHOB
GO:0032713	0.0026	negative regulation of interleukin-4 production	CD83
GO:0032733	0.0034	positive regulation of interleukin-10 production	CD83
GO:0008333	0.0051	endosome to lysosome transport	RHOB
GO:0032743	0.0059	positive regulation of interleukin-2 production	CD83
GO:0043372	0.0059	positive regulation of CD4-positive. alpha-beta T cell differentiation	CD83
GO:0006342	0.0076	chromatin silencing	LOC100154071
GO:0071479	0.0085	cellular response to ionizing radiation	RHOB
GO:2000514	0.0093	regulation of CD4-positive. alpha-beta T cell activation	CD83
**WI+CS6h-C versus Ctl-C** (Down-regulated genes)			
GO:0071941	0.0004	nitrogen cycle metabolic process	ARG2, NR1H4
GO:0043436	0.0011	oxoacid metabolic process	MTHFD1, HAO2, CDO1, ARG2, PDK4, NR1H4
GO:0009396	0.0054	folic acid-containing compound biosynthetic process	MTHFD1
GO:0034255	0.0054	regulation of urea metabolic process	NR1H4
GO:0038185	0.0054	intracellular bile acid receptor signaling pathway	NR1H4
GO:0046439	0.0054	L-cysteine metabolic process	CDO1
GO:1900094	0.0054	regulation of transcription from RNA polymerase II promoter involved in determination of left/right symmetry	CITED2
GO:1900164	0.0054	nodal signaling pathway involved in determination of lateral mesoderm left/right asymmetry	CITED2
GO:1900260	0.0054	negative regulation of RNA-directed RNA polymerase activity	EIF4A2
GO:1903413	0.0054	cellular response to bile acid	NR1H4
GO:2001250	0.0054	positive regulation of ammonia assimilation cycle	NR1H4
GO:0046394	0.0063	carboxylic acid biosynthetic process	MTHFD1, PDK4, NR1H4
GO:0019395	0.0065	fatty acid oxidation	HAO2, PDK4
GO:0009064	0.0077	glutamine family amino acid metabolic process	ARG2, NR1H4
GO:0016070	0.0092	RNA metabolic process	TRMT6, LSM6, LOC100156195, EIF4A2, CITED2, CEBPD, PAXBP1, ZNF217, NR1H4
GO:1901605	0.0095	alpha-amino acid metabolic process	MTHFD1, CDO1

**WI+CS6h-C versus Ctl-C** (Up-regulated genes)			
GO:2000113	0.0001	negative regulation of cellular macromolecule biosynthetic process	LOC100154071, LOC100154508, PLK3, BCL6, ENC1, EGR1, ID2, AURKB
GO:0043066	0.0001	negative regulation of apoptotic process	PLK3, CYR61, BCL6, PTGFR, NR4A2, AURKB, CCL2
GO:0010941	0.0002	regulation of cell death	PLK3, CYR61, BCL6, EGR1, PTGFR, NR4A2, DCPS, AURKB, CCL2
GO:0051172	0.0002	negative regulation of nitrogen compound metabolic process	LOC100154071, LOC100154508, PLK3, BCL6, ENC1, EGR1, ID2, AURKB
GO:0009890	0.0002	negative regulation of biosynthetic process	LOC100154071, LOC100154508, PLK3, BCL6, ENC1, EGR1, ID2, AURKB
GO:0010629	0.0002	negative regulation of gene expression	LOC100154071, LOC100154508, PLK3, BCL6, ENC1, EGR1, ID2, AURKB
GO:1903507	0.0004	negative regulation of nucleic acid-templated transcription	LOC100154071, LOC100154508, PLK3, BCL6, EGR1, ID2, AURKB
GO:0051253	0.0005	negative regulation of RNA metabolic process	LOC100154071, LOC100154508, PLK3, BCL6, EGR1, ID2, AURKB
GO:0016265	0.0007	death	PLK3, CYR61, BCL6, EGR1, PTGFR, NR4A2, DCPS, AURKB, CCL2
GO:0008284	0.0009	positive regulation of cell proliferation	CYR61, DCT, BCL6, PTGFR, ID2, HBEGF
GO:0000122	0.0013	negative regulation of transcription from RNA polymerase II promoter	PLK3, BCL6, EGR1, ID2, AURKB
GO:0009314	0.0013	response to radiation	PLK3, EGR1, ID2, AURKB
GO:0001666	0.0016	response to hypoxia	PLK3, EGR1, NR4A2
GO:0006342	0.0017	chromatin silencing	LOC100154071, LOC100154508
GO:0070482	0.0018	response to oxygen levels	PLK3, EGR1, NR4A2
GO:0048519	0.0020	negative regulation of biological process	LOC100154071, LOC100154508, PLK3, CYR61, BCL6, ENC1, EGR1, PTGFR, ID2, NR4A2, DCPS, AURKB, CCL2
GO:0051276	0.0023	chromosome organization	LOC100154071, LOC100154508, BCL6, HIST1H2BD, AURKB
GO:0046189	0.0026	phenol-containing compound biosynthetic process	DCT, NR4A2
GO:1901700	0.0034	response to oxygen-containing compound	PLK3, EGR1, PTGFR, NR4A2, DCPS, CCL2

GO:0071347	0.0036	cellular response to interleukin-1	EGR1, CCL2
GO:0090398	0.0042	cellular senescence	BCL6, ID2
GO:1902106	0.0042	negative regulation of leukocyte differentiation	BCL6, ID2
GO:0036294	0.0048	cellular response to decreased oxygen levels	PLK3, EGR1
GO:0031324	0.0051	negative regulation of cellular metabolic process	LOC100154071, LOC100154508, PLK3, BCL6, ENC1, EGR1, ID2, AURKB
GO:0043069	0.0054	negative regulation of programmed cell death	DCPS
GO:0010556	0.0060	regulation of macromolecule biosynthetic process	LOC100154071, LOC100154508, PLK3, CYR61, BCL6, ENC1, EGR1, ID2, AURKB, CCL2
GO:2000177	0.0061	regulation of neural precursor cell proliferation	DCT, ID2
GO:0050769	0.0065	positive regulation of neurogenesis	DCT, BCL6, ID2
GO:0000290	0.0071	deadenylation-dependent decapping of nuclear-transcribed mRNA	DCPS
GO:0002041	0.0071	intussusceptive angiogenesis	CYR61
GO:0002434	0.0071	immune complex clearance	CCL2
GO:0003278	0.0071	apoptotic process involved in heart morphogenesis	CYR61
GO:0006583	0.0071	melanin biosynthetic process from tyrosine	DCT
GO:0009838	0.0071	abscission	AURKB
GO:0021847	0.0071	ventricular zone neuroblast division	DCT
GO:0021986	0.0071	habenula development	NR4A2
GO:0032764	0.0071	negative regulation of mast cell cytokine production	BCL6
GO:0035684	0.0071	helper T cell extravasation	CCL2
GO:0036245	0.0071	cellular response to menadione	DCPS
GO:0043380	0.0071	regulation of memory T cell differentiation	BCL6
GO:0043615	0.0071	astrocyte cell migration	CCL2
GO:0043988	0.0071	histone H3-S28 phosphorylation	AURKB
GO:0045578	0.0071	negative regulation of B cell differentiation	ID2
GO:0045910	0.0071	negative regulation of DNA recombination	BCL6
GO:0048294	0.0071	negative regulation of isotype switching to IgE isotypes	BCL6
GO:0048541	0.0071	Peyer's patch development	ID2

GO:0051256	0.0071	mitotic spindle midzone assembly	AURKB
GO:0051866	0.0071	general adaptation syndrome	NR4A2
GO:0061030	0.0071	epithelial cell differentiation involved in mammary gland alveolus development	ID2
GO:0090166	0.0071	Golgi disassembly	PLK3
GO:0090265	0.0071	positive regulation of immune complex clearance by monocytes and macrophages	CCL2
GO:2000304	0.0071	positive regulation of ceramide biosynthetic process	CYR61
GO:2000502	0.0071	negative regulation of natural killer cell chemotaxis	CCL2
GO:2000777	0.0071	positive regulation of proteasomal ubiquitin-dependent protein catabolic process involved in cellular response to hypoxia	PLK3
GO:0030900	0.0075	forebrain development	DCT, ID2, NR4A2
GO:0003281	0.0077	ventricular septum development	CYR61, ID2
GO:1901576	0.0080	organic substance biosynthetic process	LOC100154071, LOC100154508, PLK3, CYR61, DCT, BCL6, ENC1, EGR1, ID2, FABP5, NR4A2, AURKB, FUT2, CCL2
GO:0051250	0.0085	negative regulation of lymphocyte activation	BCL6, ID2
**WI+CS24h-C versus Ctl-C** (Down-regulated genes)			
GO:0030258	0.0003	lipid modification	HAO2, PDK4, INPP1, HADHA
GO:0019395	0.0007	fatty acid oxidation	HAO2, PDK4, HADHA
GO:1901605	0.0009	alpha-amino acid metabolic process	MTHFD1, CDO1, ARG2, NR1H4
GO:0071941	0.0009	nitrogen cycle metabolic process	ARG2, NR1H4
GO:0019752	0.0015	carboxylic acid metabolic process	MTHFD1, HAO2, CDO1, ARG2, PDK4, NR1H4, HADHA
GO:0006082	0.0021	organic acid metabolic process	MTHFD1, HAO2, CDO1, ARG2, PDK4, NR1H4, HADHA
GO:0006290	0.0079	pyrimidine dimer repair	POLH
GO:0009396	0.0079	folic acid-containing compound biosynthetic process	MTHFD1
GO:0034255	0.0079	regulation of urea metabolic process	NR1H4
GO:0038185	0.0079	intracellular bile acid receptor signaling pathway	NR1H4
GO:0046439	0.0079	L-cysteine metabolic process	CDO1

GO:0089709	0.0079	L-histidine transmembrane transport	SLC38A1
GO:1900094	0.0079	regulation of transcription from RNA polymerase II promoter involved in determination of left/right symmetry	CITED2
GO:1900164	0.0079	nodal signaling pathway involved in determination of lateral mesoderm left/right asymmetry	CITED2
GO:1903413	0.0079	cellular response to bile acid	NR1H4
GO:2001250	0.0079	positive regulation of ammonia assimilation cycle	NR1H4
**WI+CS24h-C versus Ctl-C** (Up-regulated genes)			
GO:0043066	0.0000	negative regulation of apoptotic process	PLK3, CYR61, BCL6, CRYAB, KLF4, NR4A2, AURKB, CAST, CCL2
GO:0060548	0.0001	negative regulation of cell death	PLK3, CYR61, BCL6, CRYAB, KLF4, NR4A2, AURKB, CAST, CCL2
GO:1903507	0.0001	negative regulation of nucleic acid-templated transcription	Hist1h2af, PLK3, BCL6, EGR1, JUN, KLF4, ID2, AURKB
GO:0010629	0.0001	negative regulation of gene expression	Hist1h2af, PLK3, BCL6, EGR1, CRYAB, JUN, KLF4, ID2, AURKB
GO:0051253	0.0002	negative regulation of RNA metabolic process	Hist1h2af, PLK3, BCL6, EGR1, JUN, KLF4, ID2, AURKB
GO:0016265	0.0002	death	EPHA2, PLK3, CYR61, BCL6, EGR1, CRYAB, JUN, KLF4, NR4A2, AURKB, CAST, CCL2
GO:2000113	0.0003	negative regulation of cellular macromolecule biosynthetic process	Hist1h2af, PLK3, BCL6, EGR1, JUN, KLF4, ID2, AURKB
GO:0071480	0.0003	cellular response to gamma radiation	EGR1, CRYAB
GO:0036293	0.0003	response to decreased oxygen levels	PLK3, EGR1, CRYAB, NR4A2
GO:0043067	0.0005	regulation of programmed cell death	PLK3, CYR61, BCL6, EGR1, CRYAB, KLF4, NR4A2, AURKB, CAST, CCL2
GO:0050869	0.0006	negative regulation of B cell activation	BCL6, ID2
GO:0051172	0.0007	negative regulation of nitrogen compound metabolic process	Hist1h2af, PLK3, BCL6, EGR1, JUN, KLF4, ID2, AURKB
GO:0009890	0.0007	negative regulation of biosynthetic process	histone H2A type 1-like, PLK3, BCL6, EGR1, JUN, KLF4, ID2, AURKB
GO:1903707	0.0010	negative regulation of hemopoiesis	LEO1, BCL6, ID2

GO:0001779	0.0010	natural killer cell differentiation	ID2, IL15
GO:0000122	0.0012	negative regulation of transcription from RNA polymerase II promoter	PLK3, BCL6, EGR1, JUN, ID2, AURKB
GO:0048534	0.0015	hematopoietic or lymphoid organ development	EPHA2, LEO1, BCL6, EGR1, KLF4, ID2, IL15
GO:0002376	0.0015	immune system process	EPHA2, LEO1, BCL6, EGR1, JUN, CCL28, KLF4, ID2, FCN2, GGT1, IL1B1, CCL2, IL15
GO:0009791	0.0015	post-embryonic development	RAB3A, KLF4, NR4A2
GO:0031324	0.0016	negative regulation of cellular metabolic process	Sus scrofa Hist1h2af, PLK3, BCL6, EGR1, CRYAB, JUN, KLF4, ID2, AURKB, CAST
GO:0071478	0.0022	cellular response to radiation	EGR1, CRYAB, AURKB
GO:0051276	0.0022	chromosome organization	Hist1h2af, LEO1, BCL6, HIST1H2BD, AURKB
GO:0019538	0.0024	protein metabolic process	EPHA2, PLK3, LEO1, CYR61, LOC100511833, BCL6, EGR1, CRYAB, FKBP14, KLF4, FCN2, AURKB, GGT1, CAST, FUT2, CCL2, TGFA, IL15
GO:0006950	0.0024	response to stress	CKM, EPHA2, PLK3, CYR61, BCL6, EGR1, CRYAB, KLF4, ID2, CKM, NR4A2, FCN2, GGT1, IL1B1, CCL2, IL15
GO:0045620	0.0027	negative regulation of lymphocyte differentiation	BCL6, ID2
GO:0060561	0.0027	apoptotic process involved in morphogenesis	CYR61, CRYAB
GO:0080090	0.0029	regulation of primary metabolic process	Hist1h2af, EPHA2, PLK3, LEO1, CYR61, BCL6, EGR1, CRYAB, JUN, KLF4, ID2, NR4A2, AURKB, CAST, CCL2, TGFA, IL15
GO:0050790	0.0031	regulation of catalytic activity	EPHA2, CYR61, BCL6, CRYAB, KLF4, NR4A2, CAST, CCL2, TGFA
GO:0006342	0.0035	chromatin silencing	Sus scrofa histone H2A type 1-like
GO:0033598	0.0035	mammary gland epithelial cell proliferation	EPHA2, ID2
GO:0051781	0.0040	positive regulation of cell division	AURKB, IL1B1, TGFA
GO:0002521	0.0040	leukocyte differentiation	EPHA2, BCL6, EGR1, ID2, IL15
GO:0032268	0.0042	regulation of cellular protein metabolic process	EPHA2, PLK3, CYR61, BCL6, EGR1, CRYAB, KLF4, CAST, CCL2, TGFA, IL15
GO:0021700	0.0042	developmental maturation	RAB3A, ID2, NR4A2, IL15
GO:0070848	0.0047	response to growth factor	EPHA2, CYR61, EGR1, KLF4, CCL2

GO:0098602	0.0048	single organism cell adhesion	CYR61, BCL6, EGR1, KLF4, CCL2, IL15
GO:0048519	0.0049	negative regulation of biological process	Hist1h2af, EPHA2, PLK3, LEO1, CYR61, BCL6, EGR1, CRYAB, JUN, KLF4, ID2, NR4A2, AURKB, CAST, CCL2
GO:0010466	0.0051	negative regulation of peptidase activity	CRYAB, KLF4, CAST
GO:0010556	0.0052	regulation of macromolecule biosynthetic process	Hist1h2af, PLK3, LEO1, CYR61, BCL6, EGR1, JUN, KLF4, ID2, AURKB, CCL2, IL15
GO:0007585	0.0053	respiratory gaseous exchange	RAB3A, NR4A2
GO:1901700	0.0056	response to oxygen-containing compound	PLK3, EGR1, CRYAB, JUN, KLF4, NR4A2, CCL2
GO:0016043	0.0059	cellular component organization	RAB3A, LOC100154071, LOC100154508, EPHA2, PLK3, LEO1, CYR61, BCL6, CRYAB, KLF4, PARD6B, CLDN19, NR4A2, HIST1H2BD, AURKB, CCL2, TGFA
GO:0007369	0.0060	gastrulation	EPHA2, LEO1, KLF4
GO:0070887	0.0061	cellular response to chemical stimulus	EPHA2, PLK3, CYR61, EGR1, JUN, CCL28, KLF4, ID2, NR4A2, IL1B1, CCL2
GO:0043535	0.0063	regulation of blood vessel endothelial cell migration	EPHA2, KLF4
GO:0098609	0.0063	cell-cell adhesion	CYR61, BCL6, EGR1, KLF4, CCL2, IL15
GO:0008284	0.0067	positive regulation of cell proliferation	CYR61, BCL6, ID2, TGFA, HBEGF, IL15
GO:0051249	0.0073	regulation of lymphocyte activation	BCL6, ID2, CCL2, IL15
GO:0002088	0.0074	lens development in camera-type eye	EPHA2, CRYAB
GO:0071347	0.0074	cellular response to interleukin-1	EGR1, CCL2
GO:0043281	0.0074	regulation of cysteine-type endopeptidase activity involved in apoptotic process	CYR61, CRYAB, KLF4
GO:0009314	0.0078	response to radiation	PLK3, JUN, ID2
GO:0030162	0.0081	regulation of proteolysis	PLK3, CYR61, CRYAB, KLF4, CAST
GO:0090398	0.0085	cellular senescence	BCL6, ID2
GO:0001649	0.0091	osteoblast differentiation	EPHA2, CYR61, ID2
GO:0043087	0.0091	regulation of GTPase activity	EPHA2, BCL6, CCL2
GO:0009605	0.0094	response to external stimulus	EPHA2, BCL6, JUN, CCL28, KLF4, ID2,

			NR4A2, GGT1, CCL2, IL15
GO:0022008	0.0094	neurogenesis	RAB3A, EPHA2, BCL6, KLF4, ID2, NR4A2, CCL2
GO:0022407	0.0095	regulation of cell-cell adhesion	BCL6, KLF4, CCL2, IL15
GO:0030099	0.0095	myeloid cell differentiation	EPHA2, LEO1, BCL6, ID2
GO:0016525	0.0098	negative regulation of angiogenesis	KLF4, CCL2
GO:0071456	0.0098	cellular response to hypoxia	PLK3, EGR1
GO:1903034	0.0099	regulation of response to wounding	BCL6, KLF4, GGT1, CCL2
**WI+CS6h-C versus WI-C** (Down-regulated genes)			
GO:0019395	0.0005	fatty acid oxidation	HAO2, ACOX1, PDK4, HADHA
GO:0070295	0.0008	renal water absorption	AQP3, AQP7
GO:0030104	0.0014	water homeostasis	AQP3, AQP7, CELA2A
GO:0071918	0.0016	urea transmembrane transport	AQP3, AQP7
GO:0003091	0.0027	renal water homeostasis	AQP3, AQP7
GO:0019627	0.0040	urea metabolic process	ARG2, NR1H4
GO:0019755	0.0055	one-carbon compound transport	AQP3, AQP7
GO:0030258	0.0058	lipid modification	HAO2, ACOX1, PDK4, HADHA
GO:0006629	0.0079	lipid metabolic process	SF1, HAO2, ANG, PNPLA2, ACOX1, PDK4, NR1H4, PIK3R5, HADHA, DBI, HSD17B4
GO:0006833	0.0092	water transport	AQP3, AQP7
**WI+CS6h-C versus WI-C** (Up-regulated genes)			
GO:0060548	0.0000	negative regulation of cell death	SMAD6, BCL6, IER3, HMOX1, KLF4, GATA3, NR4A2, ID1, CAST, CCL2
GO:0046189	0.0002	phenol-containing compound biosynthetic process	DCT, GATA3, NR4A2
GO:0045444	0.0003	fat cell differentiation	SMAD6, KLF4, ID2, GATA3, NR4A2
GO:0043066	0.0004	negative regulation of apoptotic process	SMAD6, BCL6, IER3, HMOX1, KLF4, NR4A2, ID1, CCL2
GO:0030182	0.0004	neuron differentiation	RAB3A, EPHA2, BCL6, HOXD1, KLF4, ID2, ACSL4, NR4A2, EIF4G1
GO:0043067	0.0005	regulation of programmed cell death	SMAD6, BCL6, EGR1, IER3, HMOX1, KLF4, GATA3, NR4A2, ID1, CAST, CCL2

GO:0008285	0.0008	negative regulation of cell proliferation	SMAD6, STRN, BCL6, HMOX1, KLF4, ID2, GATA3
GO:0034616	0.0008	response to laminar fluid shear stress	SMAD6, KLF4
GO:0050869	0.0008	negative regulation of B cell activation	BCL6, ID2
GO:0016265	0.0010	death	EPHA2, SMAD6, BCL6, EGR1, IER3, HMOX1, KLF4, GATA3, NR4A2, ID1, CAST, CCL2
GO:0009968	0.0013	negative regulation of signal transduction	EPHA2, SMAD6, BAMBI, BCL6, EGR1, IER3, HMOX1, GATA3
GO:0003281	0.0013	ventricular septum development	SMAD6, ID2, GATA3
GO:2000679	0.0014	positive regulation of transcription regulatory region DNA binding	KLF4, GATA3
GO:0060070	0.0014	canonical Wnt signaling pathway	BAMBI, EGR1, KLF4, GATA3
GO:1903707	0.0016	negative regulation of hemopoiesis	LEO1, BCL6, ID2
GO:0090288	0.0018	negative regulation of cellular response to growth factor stimulus	SMAD6, BAMBI, GATA3
GO:0070848	0.0018	response to growth factor	EPHA2, BAMBI, KLF4, GATA3, CCL2
GO:0048869	0.0019	cellular developmental process	CYP24A1, RAB3A, EPHA2, LEO1, SMAD6, DCT, BCL6, EGR1, HOXD1, KLF4, ID2, CSL4, GATA3, NR4A2, ID1, EIF4G1, CCL2
GO:0009790	0.0021	embryo development	EPHA2, LEO1, SMAD6, HOXD1, KLF4, ID2, ACSL4, GATA3
GO:0045765	0.0023	regulation of angiogenesis	EPHA2, KLF4, ID1, CCL2,
GO:0009791	0.0024	post-embryonic development	RAB3A, KLF4, NR4A2
GO:0042423	0.0028	catecholamine biosynthetic process	GATA3, NR4A2
GO:0045668	0.0028	negative regulation of osteoblast differentiation	ID2, ID1
GO:2000026	0.0029	regulation of multicellular organismal development	EPHA2, LEO1, DCT, BCL6, KLF4, ID2, GATA3, ID1, EIF4G1, CCL2
GO:0044710	0.0031	single-organism metabolic process	CYP24A1, EPHA2, USP1, LEO1, PPP1R3C, BCL6, NAGS, IER3, ID2, ACSL4, FABP5, GATA3, PNPLA3, GLUD1, ID1, FCN2, FUT2, GUCY2C, CCL2, LPL
GO:0022008	0.0034	neurogenesis	RAB3A, EPHA2, HOXD1, KLF4, ACSL4, NR4A2, CCL2
GO:0048534	0.0037	hematopoietic or lymphoid organ development	EPHA2, LEO1, BCL6, EGR1, KLF4, ID2,

			GATA3
GO:0045620	0.0037	negative regulation of lymphocyte differentiation	BCL6, ID2
GO:1904036	0.0037	negative regulation of epithelial cell apoptotic process	GATA3, CAST
GO:0050769	0.0038	positive regulation of neurogenesis	DCT, BCL6, ID2, EIF4G1
GO:0030509	0.0042	BMP signaling pathway	SMAD6, EGR1, ID1
GO:0043433	0.0046	negative regulation of sequence-specific DNA binding transcription factor activity	KLF4, ID2, ID1
GO:0071772	0.0046	response to BMP	SMAD6, EGR1, ID1
GO:0030512	0.0047	negative regulation of transforming growth factor beta receptor signaling pathway	SMAD6, BAMBI
GO:0033598	0.0047	mammary gland epithelial cell proliferation	EPHA2, ID2
GO:0050727	0.0049	regulation of inflammatory response	BCL6, IER3, GATA3, CCL2
GO:0045934	0.0049	negative regulation of nucleobase-containing compound metabolic process	BCL6, EGR1, IER3, HMOX1, KLF4, ID2, GATA3, ID1
GO:0043491	0.0056	protein kinase B signaling	EPHA2, GATA3, CCL2
GO:0043542	0.0056	endothelial cell migration	EPHA2, KLF4, GATA3
GO:0033238	0.0059	regulation of cellular amine metabolic process	GATA3, NR4A2
GO:0071479	0.0059	cellular response to ionizing radiation	EGR1, GATA3
GO:0051098	0.0059	regulation of binding	KLF4, ID2, GATA3, ID1
GO:0000902	0.0062	cell morphogenesis	RAB3A, EPHA2, BCL6, GATA3, NR4A2, ID1, CCL2
GO:0006282	0.0071	regulation of DNA repair	USP1, IER3
GO:0007585	0.0071	respiratory gaseous exchange	RAB3A, NR4A2
GO:0009712	0.0071	catechol-containing compound metabolic process	GATA3, NR4A2
GO:0048661	0.0071	positive regulation of smooth muscle cell proliferation	HMOX1, ID2
GO:0035295	0.0072	tube development	RAB3A, EPHA2, SMAD6, ID2, GATA3, ID1
GO:0009308	0.0073	amine metabolic process	FABP5, GATA3, NR4A2
GO:0034101	0.0073	erythrocyte homeostasis	BCL6, HMOX1, ID2
GO:0009611	0.0074	response to wounding	BCL6, IER3, HMOX1, KLF4, GATA3, CCL2
GO:0002521	0.0078	leukocyte differentiation	EPHA2, BCL6, EGR1, ID2, GATA3

GO:0042440	0.0085	pigment metabolic process	DCT, HMOX1
GO:0043535	0.0085	regulation of blood vessel endothelial cell migration	EPHA2, KLF4
GO:0072358	0.0086	cardiovascular system development	EPHA2, SMAD6, KLF4, ID2, GATA3, ID1, CCL2
GO:0009314	0.0090	response to radiation	USP1, EGR1, ID2, GATA3
GO:0003205	0.0092	cardiac chamber development	SMAD6, ID2, GATA3
GO:0007369	0.0092	gastrulation	EPHA2, LEO1, KLF4
GO:0010632	0.0092	regulation of epithelial cell migration	EPHA2, KLF4, GATA3
GO:2000146	0.0092	negative regulation of cell motility	KLF4, GATA3, CCL2
GO:1902679	0.0098	negative regulation of RNA biosynthetic process	BCL6, EGR1, HMOX1, KLF4, ID2, GATA3, ID1
GO:0003382	0.0099	epithelial cell morphogenesis	GATA3, ID1
GO:0043392	0.0099	negative regulation of DNA binding	ID2, ID1
GO:0071347	0.0099	cellular response to interleukin-1	EGR1, CCL2
**WI+CS24h-C versus WI-C** (Down-regulated genes)			
GO:0019395	0.0001	fatty acid oxidation	HAO2, ACOX1, PDK4, HADHA
GO:0030258	0.0017	lipid modification	HAO2, ACOX1, PDK4, HADHA
GO:0019627	0.0020	urea metabolic process	ARG2, NR1H4
GO:0055114	0.0022	oxidation-reduction process	HAO2, PDK4, HMOX2, HADHA, HSD17B4
GO:0001101	0.0061	response to acid chemical	PDK4, NR1H4, AQP3
GO:0050891	0.0085	multicellular organismal water homeostasis	AQP3, CELA2A
**WI+CS24h-C versus WI-C** (Up-regulated genes)			
GO:0022008	0.0000	neurogenesis	RAB3A, KCNJ10, EPHA2, BCL6, HOXD1, KLF4, ID2, ACSL4, NR4A2, EIF4G1
GO:0009314	0.0004	response to radiation	USP1, EGR1, ID2, GATA3, AURKB
GO:0048731	0.0005	system development	RAB3A, KCNJ10, EPHA2, USP1, LEO1, BCL6, EGR1, HOXD1, DOK4, CLDN4, KLF4, ID2, ACSL4, GATA3, CLDN4, NR4A2, EIF4G1
GO:2000679	0.0008	positive regulation of transcription regulatory region DNA binding	KLF4, GATA3
GO:0048534	0.0008	hematopoietic or lymphoid organ development	EPHA2, LEO1, BCL6, EGR1, KLF4, ID2,

			GATA3
GO:0043066	0.0009	negative regulation of apoptotic process	BCL6, IER3, KLF4, GATA3, NR4A2, AURKB, CAST
GO:0045444	0.0011	fat cell differentiation	ID2, GATA3, KLF4, NR4A2
GO:0009791	0.0012	post-embryonic development	KLF4, NR4A2, RAB3A
GO:0014003	0.0012	oligodendrocyte development	KCNJ10, ID2
GO:0060548	0.0013	negative regulation of cell death	BCL6, IER3, KLF4, GATA3, NR4A2, AURKB, CAST
GO:0009713	0.0017	catechol-containing compound biosynthetic process	GATA3, NR4A2
GO:0051101	0.0019	regulation of DNA binding	ID2, GATA3, KLF4
GO:0030182	0.0020	neuron differentiation	RAB3A, EPHA2, HOXD1, ACSL4, NR4A2
GO:0045619	0.0026	regulation of lymphocyte differentiation	ID2, GATA3, BCL6
GO:0043542	0.0028	endothelial cell migration	EPHA2, KLF4, GATA3
GO:0033598	0.0029	mammary gland epithelial cell proliferation	EPHA2, ID2
GO:0016055	0.0030	Wnt signaling pathway	STRN, EGR1, KLF4, GATA3
GO:0044708	0.0030	single-organism behavior	ID2, NR4A2, EIF4G1, KCNJ10
GO:1903706	0.0030	regulation of hemopoiesis	LEO1, BCL6, ID2, GATA3
GO:0033238	0.0036	regulation of cellular amine metabolic process	GATA3, NR4A2
GO:0071479	0.0036	cellular response to ionizing radiation	EGR1, GATA3
GO:0009308	0.0037	amine metabolic process	FABP5, GATA3, NR4A2
GO:0006282	0.0044	regulation of DNA repair	USP1, IER3
GO:0006584	0.0044	catecholamine metabolic process	GATA3, NR4A2
GO:0007585	0.0044	respiratory gaseous exchange	RAB3A,NR4A2
GO:0045934	0.0045	negative regulation of nucleobase-containing compound metabolic process	BCL6, EGR1, IER3, KLF4, ID2, GATA3, AURKB
GO:0000122	0.0046	negative regulation of transcription from RNA polymerase II promoter	BCL6, EGR1, ID2, GATA3, AURKB
GO:0010632	0.0047	regulation of epithelial cell migration	KLF4, GATA3, EPHA2
GO:0043067	0.0048	regulation of programmed cell death	BCL6, EGR1, IER3, KLF4, GATA3, NR4A2, AURKB, CAST
GO:0043535	0.0053	regulation of blood vessel endothelial cell migration	EPHA2, KLF4

GO:0048598	0.0055	embryonic morphogenesis	EPHA2, LEO1, KLF4, ID2, GATA3
GO:0016265	0.0060	death	EPHA2, BCL6, EGR1, IER3, KLF4, GATA3, NR4A2, AURKB, CAST
GO:0001558	0.0061	regulation of cell growth	IGFBP6, BCL6, IGFBP4, ACSL4
GO:0008344	0.0062	adult locomotory behavior	KCNJ10, NR4A2
GO:1904035	0.0062	regulation of epithelial cell apoptotic process	GATA3, CAST
GO:0030099	0.0069	myeloid cell differentiation	EPHA2, LEO1, BCL6, ID2
GO:0008285	0.0069	negative regulation of cell proliferation	STRN, BCL6, KLF4, ID2, GATA3
GO:0090398	0.0072	cellular senescence	BCL6, ID2
GO:1902106	0.0072	negative regulation of leukocyte differentiation	BCL6, ID2
GO:0048869	0.0082	cellular developmental process	RAB3A, KCNJ10, EPHA2, LEO1, BCL6, EGR1, HOXD1, KLF4, ID2, ACSL4, GATA3, NR4A2, EIF4G1
GO:0045786	0.0089	negative regulation of cell cycle	IER3, ID2, GATA3, AURKB
GO:0045664	0.0092	regulation of neuron differentiation	KLF4, ID2, EIF4G1, BCL6
GO:0003180	0.0094	aortic valve morphogenesis	GATA3
GO:0009838	0.0094	abscission	AURKB
GO:0014028	0.0094	notochord formation	EPHA2
GO:0016115	0.0094	terpenoid catabolic process	CRABP1
GO:0021986	0.0094	habenula development	NR4A2
GO:0031630	0.0094	regulation of synaptic vesicle fusion to presynaptic membrane	RAB3A
GO:0032307	0.0094	negative regulation of prostaglandin secretion	ACSL4
GO:0032764	0.0094	negative regulation of mast cell cytokine production	BCL6
GO:0033523	0.0094	histone H2B ubiquitination	LEO1
GO:0034115	0.0094	negative regulation of heterotypic cell-cell adhesion	KLF4
GO:0034653	0.0094	retinoic acid catabolic process	CRABP1
GO:0035860	0.0094	glial cell-derived neurotrophic factor receptor signaling pathway	GATA3
GO:0042421	0.0094	norepinephrine biosynthetic process	GATA3
GO:0043380	0.0094	regulation of memory T cell differentiation	BCL6
GO:0043988	0.0094	histone H3-S28 phosphorylation	AURKB

GO:0045415	0.0094	negative regulation of interleukin-8 biosynthetic process	KLF4
GO:0045578	0.0094	negative regulation of B cell differentiation	ID2
GO:0045910	0.0094	negative regulation of DNA recombination	BCL6
GO:0046985	0.0094	positive regulation of hemoglobin biosynthetic process	KLF4
GO:0048172	0.0094	regulation of short-term neuronal synaptic plasticity	RAB3A
GO:0048294	0.0094	negative regulation of isotype switching to IgE isotypes	BCL6
GO:0048318	0.0094	axial mesoderm development	EPHA2
GO:0048320	0.0094	axial mesoderm formation	EPHA2
GO:0048541	0.0094	Peyer's patch development	ID2
GO:0048679	0.0094	regulation of axon regeneration	KLF4
GO:0048790	0.0094	maintenance of presynaptic active zone structure	RAB3A
GO:0051256	0.0094	mitotic spindle midzone assembly	AURKB
GO:0051866	0.0094	general adaptation syndrome	NR4A2
GO:0060035	0.0094	notochord cell development	EPHA2
GO:0060995	0.0094	cell-cell signaling involved in kidney development	GATA3
GO:0061030	0.0094	epithelial cell differentiation involved in mammary gland alveolus development	ID2
GO:0061290	0.0094	canonical Wnt signaling pathway involved in metanephric kidney development	GATA3
GO:0061670	0.0094	evoked neurotransmitter secretion	RAB3A
GO:0070586	0.0094	cell-cell adhesion involved in gastrulation	KLF4
GO:1901029	0.0094	negative regulation of mitochondrial outer membrane permeabilization involved in apoptotic signaling pathway	IER3
GO:2000342	0.0094	negative regulation of chemokine (C-X-C motif) ligand 2 production	KLF4
GO:2000607	0.0094	negative regulation of cell proliferation involved in mesonephros development	GATA3
GO:2000611	0.0094	positive regulation of thyroid hormone generation	GATA3
GO:2000675	0.0094	negative regulation of type B pancreatic cell apoptotic process	CAST
GO:2000683	0.0094	regulation of cellular response to X-ray	GATA3
GO:2000703	0.0094	negative regulation of fibroblast growth factor receptor signaling pathway involved in ureteric bud formation	GATA3

GO:2000734	0.0094	negative regulation of glial cell-derived neurotrophic factor receptor signaling pathway involved in ureteric bud formation	GATA3
GO:0032502	0.0094	developmental process	RAB3A, KCNJ10, EPHA2, USP1, LEO1, BCL6, EGR1, HOXD1, DOK4, CLDN4, KLF4, ID2, ACSL4, GATA3, CLDN4, NR4A2, EIF4G1
GO:0097285	0.0096	cell-type specific apoptotic process	GATA3, NR4A2, AURKB, CAST
GO:1903507	0.0097	negative regulation of nucleic acid-templated transcription	BCL6, EGR1, KLF4, ID2, GATA3, AURKB
GO:0071214	0.0097	cellular response to abiotic stimulus	EGR1, GATA3, AURKB
GO:0090132	0.0097	epithelium migration	KLF4, GATA3, EPHA2

Table S4: Functionnal Enrichment analysis of Gene-Ontology Biological-Process terms for differentially expressed (DE) genes in corticomedullary junction tissues.

The table provided represents the enrichment analysis of Gene Ontology Biological Process (GOBP) terms for differentially expressed genes up and down-regulated in corticomedullary junction (CMJ) compared conditions. The list of regulated genes, where genes without Entrez Gene ID have been removed, was used to perform the hypergeometrical statistical test, using GOstats package. (GOBPID: Gene Ontology Biological Process Identification number; p value: p value given by the hypergeometric test ($p<0.05$); Term: Gene Ontology Biological Process description term; and differentially expressed genes in each family: Name of gene differentially expressed in the microarray, included in the Gene Ontology family.)

GOBP-ID	p value	Terms	differentially expressed genes in each family
**WI-CMJ versus Ctl-CMJ** (Down-regulated genes)			
GO:1901029	0.0009	negative regulation of mitochondrial outer membrane permeabilization involved in apoptotic signaling pathway	IER3
GO:0045820	0.0026	negative regulation of glycolytic process	IER3
GO:0045475	0.0034	locomotor rhythm	LOC100156195
GO:0051195	0.0034	negative regulation of cofactor metabolic process	IER3
GO:0045978	0.0042	negative regulation of nucleoside metabolic process	IER3
GO:0007095	0.0051	mitotic G2 DNA damage checkpoint	IER3
GO:1902110	0.0051	positive regulation of mitochondrial membrane permeability involved in apoptotic process	IER3

GO:0001562	0.0059	response to protozoan	IER3
GO:0010972	0.0059	negative regulation of G2/M transition of mitotic cell cycle	IER3
GO:0030811	0.0068	regulation of nucleotide catabolic process	IER3
GO:0035794	0.0068	positive regulation of mitochondrial membrane permeability	IER3
GO:0003085	0.0076	negative regulation of systemic arterial blood pressure	IER3
GO:0007622	0.0076	rhythmic behavior	LOC100156195
GO:1900543	0.0076	negative regulation of purine nucleotide metabolic process	IER3
GO:0006282	0.0093	regulation of DNA repair	IER3
GO:0043471	0.0093	regulation of cellular carbohydrate catabolic process	IER3
GO:0051196	0.0093	regulation of coenzyme metabolic process	IER3
GO:0090559	0.0093	regulation of membrane permeability	IER3
GO:1902749	0.0093	regulation of cell cycle G2/M phase transition	IER3
**WI-CMJ versus Ctl-CMJ** (Up- regulated genes)			
GO:0022610	0.0003	biological adhesion	CD83, CYR61, EGR1, CD83, COL8A1, KLF4, RHOB, ACAN, MYOC, JUP
GO:1903115	0.0019	regulation of actin filament-based movement	TNNC1, JUP
GO:0009888	0.0024	tissue development	CYR61, EGR1, COL8A1, KLF4, CTSZ, TNNC1, ACAN, ORAI1, PI16
GO:0009314	0.0026	response to radiation	PLK3, EGR1, JUN, RHOB
GO:0030155	0.0028	regulation of cell adhesion	CYR61, CD83, COL8A1, KLF4, MYOC
GO:0051239	0.0028	regulation of multicellular organismal process	CD83, CYR61, KLF4, AGPAT1, RHOB, TNNC1, PI16, ACKR1, PPP2R1A, IL1B1, JUP
GO:0043502	0.0030	regulation of muscle adaptation	TNNC1, PI16
GO:0071479	0.0030	cellular response to ionizing radiation	EGR1, RHOB
GO:0043388	0.0037	positive regulation of DNA binding	EDF1, KLF4
GO:0070887	0.0044	cellular response to chemical stimulus	PLK3, CYR61, EGR1, JUN, CCL3L1, KLF4, RHOB, ACKR1, IL1B1, JUP
GO:0034097	0.0055	response to cytokine	EGR1, KLF4, ACKR1, JUN, IL1B1
GO:0072358	0.0060	cardiovascular system development	CYR61, KLF4, RHOB, TNNC1, ACAN, PI16
GO:0070252	0.0060	actin-mediated cell contraction	TNNC1, JUP

GO:0048513	0.0062	animal organ development	CD83, CYR61, EGR1, COL8A1, KLF4, CTSZ, TNNC1, ACAN, ORAI1, PI16, JUP
GO:0036294	0.0069	cellular response to decreased oxygen levels	PLK3, EGR1
GO:0032879	0.0072	regulation of localization	PLK3, CYR61, KLF4, MFGE8, TNNC1, ORAI1, MYOC, IL1B1, JUP
GO:0010811	0.0078	positive regulation of cell-substrate adhesion	CYR61, COL8A1
GO:0051050	0.0081	positive regulation of transport	PLK3, MFGE8, ORAI1, IL1B1, JUP
GO:0002041	0.0085	intussusceptive angiogenesis	CYR61
GO:0002159	0.0085	desmosome assembly	JUP
GO:0003278	0.0085	apoptotic process involved in heart morphogenesis	CYR61
GO:0003301	0.0085	physiological cardiac muscle hypertrophy	PI16
GO:0032972	0.0085	regulation of muscle filament sliding speed	TNNC1
GO:0034115	0.0085	negative regulation of heterotypic cell-cell adhesion	KLF4
GO:0045415	0.0085	negative regulation of interleukin-8 biosynthetic process	KLF4
GO:0046985	0.0085	positive regulation of hemoglobin biosynthetic process	KLF4
GO:0048679	0.0085	regulation of axon regeneration	KLF4
GO:0051232	0.0085	meiotic spindle elongation	PPP2R1A
GO:0051754	0.0085	meiotic sister chromatid cohesion. centromeric	PPP2R1A
GO:0061052	0.0085	negative regulation of cell growth involved in cardiac muscle cell development	PI16
GO:0070262	0.0085	peptidyl-serine dephosphorylation	PPP2R1A
GO:0070586	0.0085	cell-cell adhesion involved in gastrulation	KLF4
GO:0086073	0.0085	bundle of His cell-Purkinje myocyte adhesion involved in cell communication	JUP
GO:0090166	0.0085	Golgi disassembly	PLK3
GO:2000304	0.0085	positive regulation of ceramide biosynthetic process	CYR61
GO:2000342	0.0085	negative regulation of chemokine (C-X-C motif) ligand 2 production	KLF4
GO:2000777	0.0085	positive regulation of proteasomal ubiquitin-dependent protein catabolic process involved in cellular response to hypoxia	PLK3
GO:0032642	0.0088	regulation of chemokine production	KLF4, ACKR1
GO:1901700	0.0088	response to oxygen-containing compound	PLK3, EGR1, JUN, KLF4, RHOB, JUP

GO:0098602	0.0100	single organism cell adhesion	CYR61, EGR1, CD83, KLF4, JUP
**WI+CS6h-CMJ versus Ctl-CMJ** (Down-regulated genes)			
GO:0019627	0.0019	urea metabolic process	ARG2, NR1H4
GO:0048512	0.0035	circadian behavior	LOC100156195, PTGDS
GO:1901605	0.0037	alpha-amino acid metabolic process	MTHFD1, ARG2, CDO1, NR1H4
GO:0032868	0.0074	response to insulin	SOCS2, PDK4, HADHA
GO:0046394	0.0091	carboxylic acid biosynthetic process	MTHFD1, PDK4, NR1H4, PTGDS
**WI+CS6h-CMJ versus Ctl-CMJ** (Up-regulated genes)			
GO:0051253	0.0000	negative regulation of RNA metabolic process	LOC100154071, LOC100154508, PLK3, PRNP, BCL6, EGR1, JUN, HMOX1, ID2, GATA3, JUN, AURKB
GO:1903507	0.0000	negative regulation of nucleic acid-templated transcription	LOC100154071, LOC100154508, PLK3, PRNP, BCL6, EGR1, JUN, ID2, GATA3, JUN, AURKB
GO:0051172	0.0000	negative regulation of nitrogen compound metabolic process	LOC100154071, LOC100154508, PLK3, PRNP, BCL6, EGR1, JUN, HMOX1, ID2, GATA3, AURKB
GO:0009890	0.0000	negative regulation of biosynthetic process	LOC100154071, LOC100154508, PLK3, PRNP, BCL6, EGR1, JUN, HMOX1, ID2, GATA3, AURKB
GO:0010629	0.0000	negative regulation of gene expression	LOC100154071, LOC100154508, PLK3, PRNP, BCL6, EGR1, JUN, HMOX1, ID2, GATA3, AURKB
GO:2000113	0.0000	negative regulation of cellular macromolecule biosynthetic process	LOC100154071, LOC100154508, PLK3, PRNP, BCL6, EGR1, JUN, ID2, GATA3, JUN, AURKB
GO:0009314	0.0001	response to radiation	PLK3, EGR1, JUN, ID2, GATA3, AURKB
GO:0000122	0.0001	negative regulation of transcription from RNA polymerase II promoter	PLK3, BCL6, EGR1, JUN, ID2, GATA3, AURKB
GO:0045619	0.0002	regulation of lymphocyte differentiation	BCL6, CD83, ID2, GATA3
GO:0046649	0.0002	lymphocyte activation	PRNP, BCL6, EGR1, CD83, ID2, GATA3, CCL2
GO:0043066	0.0002	negative regulation of apoptotic process	PLK3, PRNP, BCL6, HMOX1, GATA3, NR4A2, AURKB, CCL2

GO:0050865	0.0003	regulation of cell activation	PRNP, BCL6, CD83, ID2, GATA3, CCL2
GO:0031324	0.0003	negative regulation of cellular metabolic process	LOC100154071, LOC100154508, PLK3, PRNP, BCL6, EGR1, JUN, HMOX1, ID2, GATA3, RGS2, JUN, AURKB
GO:0060548	0.0004	negative regulation of cell death	PLK3, PRNP, BCL6, HMOX1, GATA3, NR4A2, AURKB, CCL2
GO:0034110	0.0004	regulation of homotypic cell-cell adhesion	PRNP, BCL6, CD83, GATA3, CCL2
GO:0070489	0.0004	T cell aggregation	PRNP, BCL6, EGR1, CD83, GATA3, CCL2
GO:1903037	0.0004	regulation of leukocyte cell-cell adhesion	PRNP, BCL6, CD83, GATA3, CCL2
GO:0070486	0.0005	leukocyte aggregation	PRNP, BCL6, EGR1, CD83, GATA3, CCL2
GO:0043252	0.0006	sodium-independent organic anion transport	SLC22A6
GO:0043576	0.0006	regulation of respiratory gaseous exchange	PBX3, NR4A2
GO:0045444	0.0014	fat cell differentiation	ID2, GATA3, RGS2, NR4A2
GO:0032673	0.0014	regulation of interleukin-4 production	CD83, GATA3
GO:0070887	0.0014	cellular response to chemical stimulus	PLK3, PRNP, BAMBI, EGR1, JUN, CXCL2, HMOX1, ID2, GATA3, NR4A2, IL1B1, CCL2
GO:0030217	0.0015	T cell differentiation	BCL6, EGR1, CD83, GATA3
GO:0002695	0.0018	negative regulation of leukocyte activation	PRNP, BCL6, ID2
GO:0043067	0.0018	regulation of programmed cell death	PLK3, PRNP, BCL6, EGR1, HMOX1, GATA3, NR4A2, AURKB, CCL2
GO:0009713	0.0019	catechol-containing compound biosynthetic process	GATA3, NR4A2
GO:0071248	0.0020	cellular response to metal ion	PRNP, HMOX1, ID2
GO:0050852	0.0026	T cell receptor signaling pathway	PRNP, GATA3
GO:0016265	0.0026	death	PLK3, PRNP, BCL6, EGR1, JUN, HMOX1, GATA3, NR4A2, AURKB, CCL2
GO:0002696	0.0028	positive regulation of leukocyte activation	BCL6, CD83, GATA3, CCL2
GO:0006342	0.0033	chromatin silencing	LOC100154071, LOC100154508
GO:0016337	0.0034	single organismal cell-cell adhesion	PRNP, BCL6, EGR1, CD83, GATA3, CCL2
GO:0048519	0.0035	negative regulation of biological process	CD83, LOC100154071, LOC100154508, PLK3, PRNP, BAMBI, BCL6, EGR1, CD83, JUN, HMOX1, ID2, GATA3, RGS2, NR4A2,

			AURKB, CCL2
GO:0002376	0.0037	immune system process	CD83, PRNP, BCL6, EGR1, CD83, JUN, CXCL2, HMOX1, ID2, GATA3, FCN2, IL1B1, CCL2
GO:1903706	0.0038	regulation of hemopoiesis	BCL6, CD83, ID2, GATA3
GO:0043933	0.0038	macromolecular complex subunit organization	LOC100154071, LOC100154508, PLK3, PRNP, BCL6, ATP6V0A1, HMOX1, PARD6B, HIST1H2BD, AURKB
GO:0010556	0.0039	regulation of macromolecule biosynthetic process	LOC100154071, LOC100154508, PLK3, PRNP, BCL6, EGR1, JUN, HMOX1, ID2, GATA3, RGS2, AURKB, CCL2
GO:1902107	0.0040	positive regulation of leukocyte differentiation	CD83, ID2, GATA3
GO:0006979	0.0040	response to oxidative stress	PLK3, PRNP, HMOX1, NR4A2
GO:0033238	0.0041	regulation of cellular amine metabolic process	GATA3, NR4A2
GO:0071479	0.0041	cellular response to ionizing radiation	EGR1, GATA3
GO:0018130	0.0041	heterocycle biosynthetic process	LOC100154071, LOC100154508, PLK3, PRNP, BCL6, EGR1, ATP6V0A1, JUN, HMOX1, ID2, GATA3, NR4A2, AURKB, GUCY2C
GO:0019438	0.0043	aromatic compound biosynthetic process	LOC100154071, LOC100154508, PLK3, PRNP, BCL6, EGR1, ATP6V0A1, JUN, HMOX1, ID2, GATA3, NR4A2, AURKB, GUCY2C
GO:0001666	0.0043	response to hypoxia	PLK3, EGR1, NR4A2
GO:0034101	0.0043	erythrocyte homeostasis	BCL6, HMOX1, ID2
GO:0070482	0.0047	response to oxygen levels	PLK3, EGR1, NR4A2
GO:0006584	0.0050	catecholamine metabolic process	GATA3, NR4A2
GO:0032663	0.0050	regulation of interleukin-2 production	CD83, PRNP
GO:0043370	0.0050	regulation of CD4-positive. alpha-beta T cell differentiation	CD83, BCL6
GO:0048661	0.0050	positive regulation of smooth muscle cell proliferation	HMOX1, ID2
GO:0030155	0.0056	regulation of cell adhesion	PRNP, BCL6, CD83, GATA3, CCL2
GO:1901362	0.0063	organic cyclic compound biosynthetic process	LOC100154071, LOC100154508, PLK3, PRNP, BCL6, EGR1, ATP6V0A1, JUN, HMOX1, ID2, GATA3, NR4A2, AURKB, GUCY2C

GO:0008344	0.0070	adult locomotory behavior	PBX3, NR4A2
GO:0071347	0.0070	cellular response to interleukin-1	EGR1, CCL2
GO:0009968	0.0076	negative regulation of signal transduction	PRNP, BAMBI, BCL6, EGR1, HMOX1, RGS2
GO:0090398	0.0081	cellular senescence	BCL6, ID2
GO:1902106	0.0081	negative regulation of leukocyte differentiation	BCL6, ID2
GO:0032774	0.0084	RNA biosynthetic process	LOC100154071, LOC100154508, PLK3, PRNP, BCL6, EGR1, JUN, HMOX1, ID2, GATA3, NR4A2, AURKB
GO:0001818	0.0084	negative regulation of cytokine production	CD83, PRNP, BCL6
GO:0043087	0.0084	regulation of GTPase activity	BCL6, RGS2, CCL2
GO:0050870	0.0084	positive regulation of T cell activation	CD83, GATA3, CCL2
GO:0044271	0.0089	cellular nitrogen compound biosynthetic process	LOC100154071, LOC100154508, PLK3, PRNP, BCL6, EGR1, ATP6V0A1, JUN, HMOX1, ID2, GATA3, RGS2, NR4A2, AURKB, GUCY2C
GO:0008285	0.0089	negative regulation of cell proliferation	PRNP, BCL6, HMOX1, ID2, GATA3
GO:0014070	0.0089	response to organic cyclic compound	CYP24A1, CD83, HMOX1, JUN, CCL2
GO:0036294	0.0093	cellular response to decreased oxygen levels	PLK3, EGR1
GO:0050868	0.0093	negative regulation of T cell activation	PRNP, BCL6
GO:0002434	0.0099	immune complex clearance	CCL2
GO:0003180	0.0099	aortic valve morphogenesis	GATA3
GO:0009838	0.0099	abscission	AURKB
GO:0010519	0.0099	negative regulation of phospholipase activity	RGS2
GO:0015742	0.0099	alpha-ketoglutarate transport	SLC22A6
GO:0021986	0.0099	habenula development	NR4A2
GO:0032764	0.0099	negative regulation of mast cell cytokine production	BCL6
GO:0034395	0.0099	regulation of transcription from RNA polymerase II promoter in response to iron	HMOX1
GO:0035684	0.0099	helper T cell extravasation	CCL2
GO:0035860	0.0099	glial cell-derived neurotrophic factor receptor signaling pathway	GATA3
GO:0042167	0.0099	heme catabolic process	HMOX1

GO:0042421	0.0099	norepinephrine biosynthetic process	GATA3
GO:0043380	0.0099	regulation of memory T cell differentiation	BCL6
GO:0043615	0.0099	astrocyte cell migration	CCL2
GO:0043988	0.0099	histone H3-S28 phosphorylation	AURKB
GO:0045578	0.0099	negative regulation of B cell differentiation	ID2
GO:0045910	0.0099	negative regulation of DNA recombination	BCL6
GO:0048294	0.0099	negative regulation of isotype switching to IgE isotypes	BCL6
GO:0048541	0.0099	Peyer's patch development	ID2
GO:0051256	0.0099	mitotic spindle midzone assembly	AURKB
GO:0051866	0.0099	general adaptation syndrome	NR4A2
GO:0060995	0.0099	cell-cell signaling involved in kidney development	GATA3
GO:0061030	0.0099	epithelial cell differentiation involved in mammary gland alveolus development	ID2
GO:0061290	0.0099	canonical Wnt signaling pathway involved in metanephric kidney development	GATA3
GO:0070885	0.0099	negative regulation of calcineurin-NFAT signaling cascade	PRNP
GO:0090166	0.0099	Golgi disassembly	PLK3
GO:0090265	0.0099	positive regulation of immune complex clearance by monocytes and macrophages	CCL2
GO:0097254	0.0099	renal tubular secretion	SLC22A6
GO:2000502	0.0099	negative regulation of natural killer cell chemotaxis	CCL2
GO:2000607	0.0099	negative regulation of cell proliferation involved in mesonephros development	GATA3
GO:2000611	0.0099	positive regulation of thyroid hormone generation	GATA3
GO:2000683	0.0099	regulation of cellular response to X-ray	GATA3
GO:2000703	0.0099	negative regulation of fibroblast growth factor receptor signaling pathway involved in ureteric bud formation	GATA3
GO:2000734	0.0099	negative regulation of glial cell-derived neurotrophic factor receptor signaling pathway involved in ureteric bud formation	GATA3
GO:2000777	0.0099	positive regulation of proteasomal ubiquitin-dependent protein catabolic process involved in cellular response to hypoxia	PLK3

**WI+CS24h-CMJ versus Ctl-CMJ** (Down-regulated genes)			
GO:0019627	0.0018	urea metabolic process	ARG2, NR1H4
GO:0048512	0.0034	circadian behavior	LOC100156195, PTGDS
GO:1901605	0.0034	alpha-amino acid metabolic process	MTHFD1, ARG2, NR1H4, CDO1
GO:0032868	0.0069	response to insulin	PDK4, HADHA, SOCS2
GO:0046394	0.0084	carboxylic acid biosynthetic process	MTHFD1, PDK4, NR1H4, PTGDS
**WI+CS24h-CMJ versus Ctl-CMJ** (Up-regulated genes)			
GO:1903507	0.0000	negative regulation of nucleic acid-templated transcription	PLK3, LOC106504139, BCL6, EGR1, DDIT3, JUN, KLF4, ID2, GATA3, AURKB, histone H2A like 1, histone H2A like 1F, H2ab
GO:0051253	0.0000	negative regulation of RNA metabolic process	PLK3, LOC106504139, BCL6, EGR1, DDIT3, JUN, KLF4, ID2, GATA3, AURKB, histone H2A like 1, histone H2A like 1F, H2ab
GO:2000113	0.0000	negative regulation of cellular macromolecule biosynthetic process	PLK3, LOC106504139, BCL6, EGR1, DDIT3, JUN, KLF4, ID2, GATA3, AURKB, histone H2A like 1, histone H2A like 1F, H2ab
GO:0051172	0.0000	negative regulation of nitrogen compound metabolic process	PLK3, LOC106504139, BCL6, EGR1, DDIT3, JUN, KLF4, ID2, GATA3, AURKB, histone H2A like 1, histone H2A like 1F, H2ab
GO:0009890	0.0000	negative regulation of biosynthetic process	PLK3, LOC106504139, BCL6, EGR1, DDIT3, JUN, KLF4, ID2, GATA3, AURKB, histone H2A like 1, histone H2A like 1F, H2ab
GO:0010629	0.0000	negative regulation of gene expression	PLK3, LOC106504139, BCL6, EGR1, DDIT3, JUN, KLF4, ID2, GATA3, AURKB, histone H2A like 1, histone H2A like 1F, H2ab
GO:0000122	0.0000	negative regulation of transcription from RNA polymerase II promoter	PLK3, LOC106504139, BCL6, EGR1, DDIT3, JUN, ID2, GATA3, JUN, AURKB
GO:0009314	0.0000	response to radiation	PLK3, MME, EGR1, JUN, ID2, GATA3, AURKB
GO:0031324	0.0001	negative regulation of cellular metabolic process	PLK3, SMAD6, LOC106504139, BCL6, EGR1, DDIT3, JUN, KLF4, ID2, GATA3, RGS2, AURKB, histone H2A like 1, histone H2A like

			1F, H2ab
GO:0045444	0.0001	fat cell differentiation	SMAD6, KLF4, ID2, RGS2, NR4A2
GO:0006342	0.0001	chromatin silencing	histone H2A like 1, histone H2A like 1F, H2ab
GO:0046189	0.0003	phenol-containing compound biosynthetic process	DCT, GATA3, NR4A2
GO:0051101	0.0003	regulation of DNA binding	DDIT3, KLF4, ID2, GATA3
GO:0070848	0.0003	response to growth factor	EPHA2, SMAD6, BAMBI, EGR1, KLF4, GATA3, CCL2
GO:0048869	0.0004	cellular developmental process	CYP24A1, CD83, EPHA2, SMAD6, PBX3, DCT, BCL6, EGR1, HOXD1, PPP1R9B, DDIT3, CD83, JUN, KLF4, ID2, GATA3, RGS2, NR4A2, CCL2, ANPEP
GO:0022008	0.0006	neurogenesis	EPHA2, PBX3, DCT, BCL6, HOXD1, PPP1R9B, KLF4, ID2, NR4A2, CCL2
GO:0034616	0.0009	response to laminar fluid shear stress	SMAD6, KLF4
GO:0043576	0.0009	regulation of respiratory gaseous exchange	PBX3, NR4A2
GO:0050869	0.0009	negative regulation of B cell activation	BCL6, ID2
GO:0006355	0.0011	regulation of transcription. DNA-templated	PLK3, SMAD6, LOC106504139, BCL6, EGR1, HOXD1, DDIT3, JUN, KLF4, ID2, GATA3, AURKB, histone H2A like 1, histone H2A like 1F, H2ab
GO:0043066	0.0011	negative regulation of apoptotic process	PLK3, SMAD6, BCL6, KLF4, GATA3, NR4A2, AURKB, CCL2
GO:2001141	0.0012	regulation of RNA biosynthetic process	PLK3, SMAD6, LOC106504139, BCL6, EGR1, HOXD1, DDIT3, JUN, KLF4, ID2, GATA3, AURKB, histone H2A like 1, histone H2A like 1F, H2ab
GO:2000679	0.0015	positive regulation of transcription regulatory region DNA binding	KLF4, GATA3
GO:0003281	0.0015	ventricular septum development	SMAD6, ID2, GATA3
GO:0016265	0.0016	death	EPHA2, PLK3, SMAD6, BCL6, EGR1, DDIT3, JUN, KLF4, GATA3,NR4A2, AURKB, CCL2
GO:0071310	0.0017	cellular response to organic substance	SMAD6, BAMBI, MME, EGR1, PPP1R9B, DDIT3, JUN, KLF4, GATA3, NR4A2, IL1B1,

			CCL2
GO:0042221	0.0018	response to chemical	CYP24A1, CD83, EPHA2, PLK3, SMAD6, BAMBI, MME, EGR1, PPP1R9B, DDIT3, CD83, JUN, KLF4, ID2, GATA3, NR4A2, IL1B1, CCL2
GO:0009968	0.0018	negative regulation of signal transduction	EPHA2, SMAD6, BAMBI, BCL6, EGR1, DDIT3, GATA3, RGS2
GO:0060548	0.0018	negative regulation of cell death	PLK3, SMAD6, BCL6, KLF4, GATA3, NR4A2, AURKB, CCL2
GO:0007569	0.0018	cell aging	MME, BCL6, ID2
GO:0034654	0.0020	nucleobase-containing compound biosynthetic process	PLK3, SMAD6, LOC106504139, BCL6, EGR1, HOXD1, DDIT3, JUN, KLF4, ID2, GATA3, NR4A2, AURKB, GUCY2C, histone H2A like 1, histone H2A like 1F, H2ab
GO:0090288	0.0021	negative regulation of cellular response to growth factor stimulus	SMAD6, BAMBI, GATA3
GO:0032673	0.0022	regulation of interleukin-4 production	CD83, GATA3
GO:0032502	0.0025	developmental process	CYP24A1, CD83, EPHA2, SMAD6, PBX3, DCT, MME, BCL6, EGR1, HOXD1, PPP1R9B, DDIT3, CD83, CLDN4, JUN, KLF4, ID2, GATA3, RGS2, CLDN4, NR4A2, AURKB, CCL2, ANPEP
GO:0040029	0.0027	regulation of gene expression. epigenetic	histone H2A like 1, histone H2A like 1F, H2ab
GO:0071214	0.0028	cellular response to abiotic stimulus	MME, EGR1, GATA3, AURKB
GO:0043067	0.0028	regulation of programmed cell death	PLK3, SMAD6, BCL6, EGR1, DDIT3, KLF4, GATA3, NR4A2, AURKB, CCL2
GO:0030182	0.0028	neuron differentiation	EPHA2, PBX3, BCL6, HOXD1, PPP1R9B, KLF4, ID2, NR4A2
GO:0072358	0.0029	cardiovascular system development	EPHA2, SMAD6, DDIT3, KLF4, ID2, GATA3, CCL2, ANPEP
GO:0045580	0.0031	regulation of T cell differentiation	CD83, BCL6, CD83, GATA3
GO:0042423	0.0031	catecholamine biosynthetic process	GATA3, NR4A2
GO:0030155	0.0031	regulation of cell adhesion	CD83, EPHA2, BCL6, CD83, KLF4, GATA3,

			CCL2
GO:0007275	0.0035	multicellular organism development	CD83, EPHA2, SMAD6, PBX3, DCT, MME, BCL6, EGR1, HOXD1, PPP1R9B, DDIT3, CD83, CLDN4, KLF4, ID2, GATA3, CLDN4, NR4A2, AURKB, CCL2, ANPEP
GO:0051241	0.0040	negative regulation of multicellular organismal process	CD83, BCL6, DDIT3, CD83, KLF4, ID2, GATA3, RGS2, CCL2
GO:0045620	0.0041	negative regulation of lymphocyte differentiation	BCL6, ID2
GO:0009059	0.0041	macromolecule biosynthetic process	PLK3, LOC106504139, SMAD6, BCL6, EGR1, HOXD1, DDIT3, JUN, KLF4, ID2, GATA3, RGS2, NR4A2, AURKB, FUT2, CCL2, histone H2A like 1, histone H2A like 1F, H2ab
GO:0001822	0.0045	kidney development	SMAD6, MME, ID2, GATA3
GO:0051251	0.0045	positive regulation of lymphocyte activation	CD83, BCL6, GATA3, CCL2
GO:0044249	0.0046	cellular biosynthetic process	PLK3, SMAD6, DCT, LOC106504139, BCL6, EGR1, HOXD1, NAGS, NAGS, DDIT3, JUN, KLF4, ID2, FABP5, GATA3, RGS2, NR4A2, AURKB, FUT2, GUCY2C, histone H2A like 1, histone H2A like 1F, H2ab
GO:0071495	0.0047	cellular response to endogenous stimulus	SMAD6, BAMBI, EGR1, PPP1R9B, JUN, GATA3, NR4A2, JUN, CCL2
GO:0002694	0.0048	regulation of leukocyte activation	CD83, BCL6, ID2, GATA3, CCL2
GO:0048534	0.0048	hematopoietic or lymphoid organ development	CD83, EPHA2, BCL6, EGR1, CD83, KLF4, ID2, GATA3
GO:0097285	0.0050	cell-type specific apoptotic process	DDIT3, GATA3, NR4A2, AURKB, CCL2
GO:0019219	0.0051	regulation of nucleobase-containing compound metabolic process	PLK3, SMAD6, LOC106504139, BCL6, EGR1, HOXD1, DDIT3, JUN, KLF4, ID2, GATA3, AURKB, histone H2A like 1, histone H2A like 1F, H2ab
GO:0030512	0.0052	negative regulation of transforming growth factor beta receptor signaling pathway	SMAD6, BAMBI
GO:0033598	0.0052	mammary gland epithelial cell proliferation	EPHA2, ID2

GO:0051898	0.0052	negative regulation of protein kinase B signaling	EPHA2, DDIT3
GO:0043433	0.0053	negative regulation of sequence-specific DNA binding transcription factor activity	KLF4, ID2, DDIT3
GO:0060828	0.0053	regulation of canonical Wnt signaling pathway	BAMBI, EGR1, DDIT3
GO:0007626	0.0058	locomotory behavior	PBX3, ID2, NR4A2
GO:0051276	0.0062	chromosome organization	BCL6, HIST1H2BD, AURKB, histone H2A like 1, histone H2A like 1F, H2ab
GO:0043542	0.0064	endothelial cell migration	EPHA2, KLF4, GATA3
GO:0033238	0.0064	regulation of cellular amine metabolic process	GATA3, NR4A2
GO:0071479	0.0064	cellular response to ionizing radiation	EGR1, GATA3
GO:0001568	0.0064	blood vessel development	EPHA2, SMAD6, DDIT3, KLF4, CCL2, ANPEP
GO:0034097	0.0067	response to cytokine	MME, EGR1, JUN, KLF4, IL1B1, CCL2
GO:0001816	0.0069	cytokine production	CD83, BCL6, DDIT3, CD83, KLF4, GATA3, CCL2
GO:0043523	0.0070	regulation of neuron apoptotic process	DDIT3, NR4A2, CCL2
GO:0050867	0.0074	positive regulation of cell activation	CD83, BCL6, CD83, GATA3, CCL2
GO:1902107	0.0076	positive regulation of leukocyte differentiation	CD83, ID2, GATA3
GO:0034110	0.0078	regulation of homotypic cell-cell adhesion	CD83, BCL6, GATA3, CCL2
GO:0006775	0.0078	fat-soluble vitamin metabolic process	CYP24A1, CRABP1
GO:0009712	0.0078	catechol-containing compound metabolic process	GATA3, NR4A2
GO:0043370	0.0078	regulation of CD4-positive. alpha-beta T cell differentiation	CD83, BCL6
GO:0042110	0.0078	T cell activation	CD83, BCL6, EGR1, CD83, GATA3, CCL2
GO:0071593	0.0078	lymphocyte aggregation	CD83, BCL6, EGR1, CD83, GATA3, CCL2
GO:0001666	0.0083	response to hypoxia	PLK3, EGR1, NR4A2
GO:0009308	0.0083	amine metabolic process	FABP5, GATA3, NR4A2
GO:0043547	0.0083	positive regulation of GTPase activity	EPHA2, RGS2, CCL2
GO:1903037	0.0087	regulation of leukocyte cell-cell adhesion	CD83, BCL6, GATA3, CCL2
GO:1903706	0.0087	regulation of hemopoiesis	CD83, BCL6, ID2, GATA3
GO:0070482	0.0090	response to oxygen levels	PLK3, EGR1, NR4A2
GO:0043535	0.0093	regulation of blood vessel endothelial cell migration	EPHA2, KLF4

GO:0045599	0.0093	negative regulation of fat cell differentiation	DDIT3, GATA3
GO:0071704	0.0096	organic substance metabolic process	CYP24A1, NXF1, EPHA2, PLK3, SMAD6, DCT, LOC106504139, MME, BCL6, EGR1, HOXD1, PPP1R9B, NAGS, CRABP1, DDIT3, JUN, ENTPD1, KLF4, ID2, FABP5, GATA3, RGS2, CRABP1, NR4A2, GLUD1, FCN2, AURKB, FUT2, GUCY2C, CCL2, ANPEP, histone H2A like 1, histone H2A like 1F, H2ab
GO:0060070	0.0096	canonical Wnt signaling pathway	KLF4, GATA3
**WI+CS6h-CMJ versus WI-CMJ** (Down-regulated genes)			
GO:0070295	0.0000	renal water absorption	AQP3, AQP7, HYAL2
GO:1903115	0.0000	regulation of actin filament-based movement	PLN, TNNC1, JUP, CAV1
GO:0003091	0.0002	renal water homeostasis	AQP3, AQP7, HYAL2
GO:0050878	0.0002	regulation of body fluid levels	F10, HPS6, AQP3, AQP7, F3, EDN1, CELA2A, CAV1, HYAL2
GO:0030104	0.0003	water homeostasis	AQP3, AQP7, CELA2A, HYAL2
GO:2001238	0.0003	positive regulation of extrinsic apoptotic signaling pathway	ITM2C, PPP2R1A, CAV1, HYAL2
GO:0006941	0.0008	striated muscle contraction	PLN, ARG2, ARG2, TNNC1, JUP, CAV1
GO:0006833	0.0012	water transport	AQP3, AQP7, HYAL2
GO:0072330	0.0014	monocarboxylic acid biosynthetic process	FAXDC2, SCD5, PDK4, NR1H4, EDN1, PTGDS
GO:0006631	0.0015	fatty acid metabolic process	FAXDC2, ACOX1, SCD5, PDK4, EDN1, HADHA, PTGDS, CAV1
GO:0086003	0.0017	cardiac muscle cell contraction	PLN, JUP, CAV1
GO:0035810	0.0019	positive regulation of urine volume	EDN1, HYAL2
GO:0098911	0.0019	regulation of ventricular cardiac muscle cell action potential	JUP, CAV1
GO:0016053	0.0021	organic acid biosynthetic process	MTHFD1, FAXDC2, SCD5, PDK4, NR1H4, EDN1, PTGDS
GO:0003012	0.0025	muscle system process	PLN, ARG2, LMCD1, TNNC1, EDN1, JUP, CAV1
GO:0007155	0.0026	cell adhesion	CITED2, ABL2, CITED2, CLDN11, CCDC80, COL8A1, CTNND1, NT5E, COL8A

			,LGALS3,RHOB, ACAN, MYOC, ENG, CD34, JUP, CAV1
GO:0060047	0.0035	heart contraction	PLN, TNNC1, EDN1, JUP, CAV1
GO:0043502	0.0036	regulation of muscle adaptation	FBXO32, TNNC1
GO:0071918	0.0037	urea transmembrane transport	AQP3, AQP7
GO:0086103	0.0037	G-protein coupled receptor signaling pathway involved in heart process	PLN, CAV1
GO:2000811	0.0037	negative regulation of anoikis	PDK4, CAV1
GO:0055117	0.0038	regulation of cardiac muscle contraction	PLN, JUP, CAV1
GO:0007204	0.0039	positive regulation of cytosolic calcium ion concentration	ABL2, PLN, EDN1, EPO, CAV1
GO:0071560	0.0039	cellular response to transforming growth factor beta stimulus	CITED2, RASL11B, CAV1, ENG, HYAL2
GO:2001233	0.0042	regulation of apoptotic signaling pathway	PLEKHF1, LGALS3, ITM2C, PPP2R1A, EPO, CAV1, HYAL2
GO:0009887	0.0051	organ morphogenesis	CITED2, ABL2, CITED2, COL8A1, AQP3, CTSZ, TNNC1, ACAN, RING1, EDN1, CAPN1, CAV1, HYAL2
GO:0006636	0.0059	unsaturated fatty acid biosynthetic process	SCD5, PTGDS, EDN1
GO:0035637	0.0059	multicellular organismal signaling	PLN, JUP, CAV1
GO:1904063	0.0059	negative regulation of cation transmembrane transport	PLN, EPO, CAV1
GO:0051926	0.0061	negative regulation of calcium ion transport	PLN, EPO
GO:0060416	0.0061	response to growth hormone	SOCS2, IGFBP5
GO:0061099	0.0061	negative regulation of protein tyrosine kinase activity	CAV1, HYAL2
GO:0086065	0.0061	cell communication involved in cardiac conduction	JUP, CAV1
GO:0051341	0.0071	regulation of oxidoreductase activity	ABL2, CAV1, EDN1
GO:0007166	0.0080	cell surface receptor signaling pathway	CITED2, ABL2, PLN, SOCS2, CITED2, RASL11B, CTNND1, LGALS3, ITM2C, MKNK1, PDK4, ACKR1, LCN2, NR1H4, F3, EDN1, PPP2R1A, ENG, IGFBP5, NTRK3, CAV1, HYAL2
GO:0001101	0.0083	response to acid chemical	ABL2, AQP3, PDK4, NR1H4
GO:0050896	0.0083	response to stimulus	CITED2, ABL2, RHOG, PLN, SOCS2, PLEKHF1, CITED2, RASL11B, PIK3IP1,

			CTNND1, CFD, USP2, NT5E, IFITM1, LMCD1, TNFSF10,ANG, FBXO32, F10, LOC780409, SLA-1, LGALS3, HPS6, AQP3, SLA-5, ITM2C, RHOB, SERPING1, ZNF217, TNNC1, HUS1 MKNK1, PDK4,ARF3, PROCR, SLA-DOA, ACKR1, LCN2, NR1H4, F3, EDN1, HADHA, ABCG2, PPP2R1A, ENG, IGFBP5, EPO, THRA, PTGDS, NTRK3, JUP, CAV1, HYAL2
GO:0010522	0.0084	regulation of calcium ion transport into cytosol	PLN, EPO,CAV1
GO:0017015	0.0084	regulation of transforming growth factor beta receptor signaling pathway	CITED2, RASL11B, CAV1
GO:2001257	0.0084	regulation of cation channel activity	PLN, EPO, CAV1
GO:0002026	0.0090	regulation of the force of heart contraction	PLN, CAV1
GO:0010611	0.0090	regulation of cardiac muscle hypertrophy	LMCD1, EDN1
GO:0019627	0.0090	urea metabolic process	ARG2, NR1H4
GO:0060056	0.0090	mammary gland involution	CAPN1, CV1
GO:0086091	0.0090	regulation of heart rate by cardiac conduction	JUP, CAV1
**WI+CS6h-CMJ versus WI-CMJ** (Up-regulated genes)			
GO:0006434	0.0065	seryl-tRNA aminoacylation	SARS
GO:0031643	0.0065	positive regulation of myelination	DICER1
GO:0032764	0.0065	negative regulation of mast cell cytokine production	BCL6
GO:0033168	0.0065	conversion of ds siRNA to ss siRNA involved in RNA interference	DICER1
GO:0033523	0.0065	histone H2B ubiquitination	LEO1
GO:0034395	0.0065	regulation of transcription from RNA polymerase II promoter in response to iron	HMOX1
GO:0035280	0.0065	miRNA loading onto RISC involved in gene silencing by miRNA	DICER1
GO:0042167	0.0065	heme catabolic process	HMOX1
GO:0043380	0.0065	regulation of memory T cell differentiation	BCL6
GO:0045910	0.0065	negative regulation of DNA recombination	BCL6
GO:0048294	0.0065	negative regulation of isotype switching to IgE isotypes	BCL6
GO:0060253	0.0065	negative regulation of glial cell proliferation	DICER1

GO:0070173	0.0065	regulation of enamel mineralization	DICER1
GO:2000630	0.0065	positive regulation of miRNA metabolic process	DICER1
GO:2000675	0.0065	negative regulation of type B pancreatic cell apoptotic process	CAST
GO:1903707	0.0072	negative regulation of hemopoiesis	LEO1, BCL6
GO:0009791	0.0095	post-embryonic development	CCDC47, DICER1
**WI+CS24h-CMJ versus WI-CMJ** (Down-regulated genes)			
GO:0070295	0.0000	renal water absorption	AQP3, AQP7, HYAL2
GO:0003091	0.0001	renal water homeostasis	AQP3, AQP7, HYAL2
GO:0030104	0.0002	water homeostasis	AQP3, AQP7, CELA2A, HYAL2
GO:0050878	0.0005	regulation of body fluid levels	F10, HPS6, AQP3, AQP7, F2, EDN1, CELA2A, HYAL2
GO:0072330	0.0007	monocarboxylic acid biosynthetic process	FAXDC2, SCD5, PDK4, NR1H4, EDN1, PTGDS
GO:0006833	0.0009	water transport	AQP3, AQP7, HYAL2
GO:0016053	0.0011	organic acid biosynthetic process	MTHFD1, FAXDC2, SCD5, PDK4, NR1H4, EDN1, PTGDS
GO:0043502	0.0012	regulation of muscle adaptation	FBXO32, TNNC1, EDN1
GO:0035810	0.0015	positive regulation of urine volume	EDN1, HYAL2
GO:0071918	0.0030	urea transmembrane transport	AQP3, AQP7
GO:0006631	0.0032	fatty acid metabolic process	FAXDC2, ACOX1, SCD5, PDK4, EDN1, HADHA, PTGDS
GO:0006636	0.0042	unsaturated fatty acid biosynthetic process	SCD5, EDN1, PTGDS
GO:0009887	0.0057	organ morphogenesis	CITED2, ABL2, COL8A1, AQP3, CTSZ, TNNC1, ACAN, RING1, EDN1, CAPN1, HYAL2
GO:0019627	0.0072	urea metabolic process	ARG2, NR1H4
GO:0071229	0.0097	cellular response to acid chemical	ABL2, PDK4, NR1H4
GO:0010863	0.0099	positive regulation of phospholipase C activity	ABL2, ANG
GO:0019755	0.0099	one-carbon compound transport	AQP3, AQP7
GO:2000352	0.0099	negative regulation of endothelial cell apoptotic process	ABL2, ANGPTL4
**WI+CS24h-CMJ versus WI-CMJ** (Up-regulated genes)			

GO:0030182	0.0006	neuron differentiation	EPHA2, BCL6, HOXD1, ID2, ACSL4, NR4A2, EIF4G1
GO:0050769	0.0007	positive regulation of neurogenesis	DCT, BCL6, ID2, EIF4G1
GO:0033598	0.0020	mammary gland epithelial cell proliferation	EPHA2, ID2
GO:0006282	0.0030	regulation of DNA repair	USP1, IER3
GO:0046189	0.0030	phenol-containing compound biosynthetic process	DCT, NR4A2
GO:0090398	0.0049	cellular senescence	BCL6, ID2
GO:1902106	0.0049	negative regulation of leukocyte differentiation	BCL6, ID2
GO:0009628	0.0049	response to abiotic stimulus	CKM, USP1, ID2, NR4A2, DNAJA4
GO:0009408	0.0056	response to heat	CKM, DNAJA4
GO:2000177	0.0072	regulation of neural precursor cell proliferation	DCT, ID2
GO:0006583	0.0077	melanin biosynthetic process from tyrosine	DCT
GO:0014028	0.0077	notochord formation	EPHA2
GO:0016115	0.0077	terpenoid catabolic process	CRABP1
GO:0021847	0.0077	ventricular zone neuroblast division	DCT
GO:0021986	0.0077	habenula development	NR4A2
GO:0032307	0.0077	negative regulation of prostaglandin secretion	ACSL4
GO:0032764	0.0077	negative regulation of mast cell cytokine production	BCL6
GO:0034653	0.0077	retinoic acid catabolic process	CRABP1
GO:0043380	0.0077	regulation of memory T cell differentiation	BCL6
GO:0045578	0.0077	negative regulation of B cell differentiation	ID2
GO:0045910	0.0077	negative regulation of DNA recombination	BCL6
GO:0048294	0.0077	negative regulation of isotype switching to IgE isotypes	BCL6
GO:0048318	0.0077	axial mesoderm development	EPHA2
GO:0048320	0.0077	axial mesoderm formation	EPHA2
GO:0048541	0.0077	Peyer's patch development	ID2
GO:0051866	0.0077	general adaptation syndrome	NR4A2
GO:0060035	0.0077	notochord cell development	EPHA2
GO:0061030	0.0077	epithelial cell differentiation involved in mammary gland alveolus development	ID2

GO:1901029	0.0077	negative regulation of mitochondrial outer membrane permeabilization involved in apoptotic signaling pathway	IER3
GO:2000675	0.0077	negative regulation of type B pancreatic cell apoptotic process	CAST
GO:0030900	0.0093	forebrain development	DCT, ID2, NR4A2
GO:0007399	0.0098	nervous system development	DCT, BCL6, HOXD1, ID2, ACSL4, NR4A2, EIF4G1
GO:0051250	0.0099	negative regulation of lymphocyte activation	BCL6, ID2

Table S5: Functionnal Enrichment analysis of Gene-Ontology Molecular Function terms for differentially expressed (DE) genes in cortex tissues.

The table provided represents the enrichment analysis of Gene Ontology Molecular Function (GOMF) terms for differentially expressed genes up and down-regulated in cortex compared conditions. The list of regulated genes, where genes without Entrez Gene ID have been removed, was used to perform the hypergeometrical statistical test, using GOstats package. (GOMFID: Gene Ontology Molecular Function Identification number; p value: p value given by the hypergeometric test ($p<0.05$); Term: Gene Ontology Molecular Function description term; and differentially expressed genes in each family: Name of gene differentially expressed in the microarray, included in the Gene Ontology family).

GOMF-ID	p value	Terms	differentially expressed genes in each family
**WI-C versus Ctl-C** (Down-regulated genes)			
-	-	-	-
**WI-C versus Ctl-C (Up- regulated genes)**			
GO:0019003	0.0119	GDP binding	RHOB
GO:0003677	0.0400	DNA binding	HIST1H2AB, FOS
**WI+CS6h-C versus Ctl-C (Down-regulated genes)**			
GO:1901363	0.0003	heterocyclic compound binding	MX2, TRMT6, LSM6, MTHFD1, LOC100156195, EIF4A2, CEBPD, HAO2, PAXBP1, ZFAND5, CYP39A1, ZNF217, GBP2, KLF15, MKNK1, PDK4, CYP2C42, NR1H4, CYP4A21
GO:0097159	0.0004	organic cyclic compound binding	MX2, TRMT6, LSM6, MTHFD1,

			LOC100156195, EIF4A2, CEBPD, HAO2, PAXBP1, ZFAND5, CYP39A1, ZNF217, GBP2, KLF15, MKNK1, PDK4, CYP2C42, NR1H4, CYP4A21
GO:0043167	0.0010	ion binding	MX2, MTHFD1, EIF4A2, HAO2, ZNF512, ADAMDEC1, CDO1, ARG2, ZFAND5, CYP39A1, ZNF217, GBP2, KLF15, MKNK1, PDK4, CYP2C42, NR1H4, CYP4A21
GO:0005506	0.0011	iron ion binding	CDO1, CYP39A1, CYP2C42, CYP4A21
GO:0001190	0.0033	transcriptional activator activity. RNA polymerase II transcription factor binding	CITED2, NR1H4
GO:0004497	0.0047	monooxygenase activity	CYP39A1, CYP2C42, CYP4A21
GO:0004329	0.0074	formate-tetrahydrofolate ligase activity	MTHFD1
GO:0004488	0.0074	methylenetetrahydrofolate dehydrogenase (NADP+) activity	MTHFD1
GO:0017172	0.0074	cysteine dioxygenase activity	CDO1
GO:0033780	0.0074	taurochenodeoxycholate 6alpha-hydroxylase activity	CYP4A21
GO:0052853	0.0074	long-chain-(S)-2-hydroxy-long-chain-acid oxidase activity	HAO2
GO:1902122	0.0074	chenodeoxycholic acid binding	NR1H4
GO:0016705	0.0077	oxidoreductase activity. acting on paired donors. with incorporation or reduction of molecular oxygen	CYP39A1, CYP2C42, CYP4A21
GO:0001191	0.0092	transcriptional repressor activity. RNA polymerase II transcription factor binding	CITED2, NR1H4
GO:0020037	0.0120	heme binding	CYP39A1, CYP2C42, CYP4A21
GO:0004053	0.0147	arginase activity	ARG2
GO:0004887	0.0147	thyroid hormone receptor activity	NR1H4
GO:0016899	0.0147	oxidoreductase activity. acting on the CH-OH group of donors. oxygen as acceptor	HAO2
GO:0038181	0.0147	bile acid receptor activity	NR1H4
GO:0000976	0.0153	transcription regulatory region sequence-specific DNA binding	LOC100156195, PAXBP1, ZNF217, NR1H4
GO:0009931	0.0220	calcium-dependent protein serine/threonine kinase activity	MKNK1
GO:0003690	0.0233	double-stranded DNA binding	LOC100156195, PAXBP1, ZNF217, NR1H4

GO:0043565	0.0234	sequence-specific DNA binding	LOC100156195, CEBPD, PAXBP1, ZNF217, NR1H4
GO:0046872	0.0255	metal ion binding	ZNF512, ADAMDEC1, CDO1, ARG2, ZFAND5, CYP39A1, ZNF217, KLF15, CYP2C42, NR1H4, CYP4A21
GO:0070888	0.0292	E-box binding	LOC100156195
GO:0000975	0.0317	regulatory region DNA binding	LOC100156195, PAXBP1, ZNF217, NR1H4
GO:0003700	0.0338	transcription factor activity. sequence-specific DNA binding	CITED2, CEBPD, PAXBP1, ZNF217, NR1H4
GO:0005516	0.0436	calmodulin binding	MKNK1
GO:0010181	0.0436	FMN binding	HAO2
GO:0016645	0.0436	oxidoreductase activity. acting on the CH-NH group of donors	MTHFD1
GO:0003924	0.0445	GTPase activity	MX2, GBP2
**WI+CS6h-C versus Ctl-C (Up-regulated genes)**			
GO:0050072	0.0077	m7G(5')pppN diphosphatase activity	DCPS
GO:0000977	0.0106	RNA polymerase II regulatory region sequence-specific DNA binding	BCL6, EGR1, FOS, NR4A2
GO:0000340	0.0153	RNA 7-methylguanosine cap binding	DCPS
GO:0004167	0.0153	dopachrome isomerase activity	DCT
GO:0004958	0.0153	prostaglandin F receptor activity	PTGFR
GO:0008107	0.0153	galactoside 2-alpha-L-fucosyltransferase activity	FUT2
GO:0000982	0.0169	transcription factor activity. RNA polymerase II core promoter proximal region sequence-specific binding	EGR1, FOS, NR4A2
GO:1990837	0.0188	sequence-specific double-stranded DNA binding	BCL6, EGR1, FOS, NR4A2
GO:0001161	0.0228	intronic transcription regulatory region sequence-specific DNA binding	BCL6
GO:0005154	0.0228	epidermal growth factor receptor binding	HBEGF
GO:0035174	0.0228	histone serine kinase activity	AURKB
GO:0008201	0.0289	heparin binding	CYR61, HBEGF
GO:0004712	0.0304	protein serine/threonine/tyrosine kinase activity	AURKB
GO:0048020	0.0304	CCR chemokine receptor binding	CCL2
GO:0044212	0.0339	transcription regulatory region DNA binding	BCL6, EGR1, FOS, NR4A2
GO:0001067	0.0359	regulatory region nucleic acid binding	BCL6, EGR1, FOS, NR4A2

GO:0003677	0.0364	DNA binding	LOC100154071, LOC100154508, BCL6, EGR1, FOS, NR4A2, HIST1H2BD
GO:0005178	0.0378	integrin binding	CYR61
GO:0004954	0.0452	prostanoid receptor activity	PTGFR
GO:0008417	0.0452	fucosyltransferase activity	FUT2
**WI+CS24h-C versus Ctl-C (Down-regulated genes)**			
GO:0005506	0.0031	iron ion binding	CDO1, CYP39A1, CYP2C42, CYP4A21
GO:1901363	0.0037	heterocyclic compound binding	MX2, TRMT6, LSM6, MTHFD1, LOC100156195, HIVEP1, PEX6, POLH, CEBPD, HAO2, PAXBP1, ZFAND5, CYP39A1, ZNF217, GBP2, KLF15, MKNK1, PDK4, CYP2C42, NR1H4, CYP4A21
GO:0097159	0.0050	organic cyclic compound binding	MX2, TRMT6, LSM6, MX2, MTHFD1, LOC100156195, HIVEP1, PEX6, POLH, CEBPD, HAO2, PAXBP1, MX2, ZFAND5, CYP39A1, ZNF217, GBP2, KLF15, MKNK1, PDK4, CYP2C42, NR1H4, CYP4A21
GO:0001190	0.0056	transcriptional activator activity. RNA polymerase II transcription factor binding	CITED2, NR1H4
GO:0000976	0.0082	transcription regulatory region sequence-specific DNA binding	LOC100156195, HIVEP1, PAXBP1, ZNF217, NR1H4
GO:0000980	0.0088	RNA polymerase II distal enhancer sequence-specific DNA binding	HIVEP1, NR1H4
GO:0043167	0.0089	ion binding	MX2, MTHFD1, HIVEP1, PEX6, HAO2, ZNF512, ADAMDEC1, CDO1, ARG2, MX2, ZFAND5, CYP39A1, ZNF217, GBP2, KLF15, MKNK1, PDK4, CYP2C42, INPP1, NR1H4, CYP4A21
GO:0004300	0.0097	enoyl-CoA hydratase activity	HADHA
GO:0004329	0.0097	formate-tetrahydrofolate ligase activity	MTHFD1
GO:0004441	0.0097	inositol-1.4-bisphosphate 1-phosphatase activity	INPP1
GO:0004488	0.0097	methylenetetrahydrofolate dehydrogenase (NADP+) activity	MTHFD1
GO:0016509	0.0097	long-chain-3-hydroxyacyl-CoA dehydrogenase activity	HADHA

GO:0017172	0.0097	cysteine dioxygenase activity	CDO1
GO:0033780	0.0097	taurochenodeoxycholate 6alpha-hydroxylase activity	CYP4A21
GO:0052853	0.0097	long-chain-(S)-2-hydroxy-long-chain-acid oxidase activity	HAO2
GO:1902122	0.0097	chenodeoxycholic acid binding	NR1H4
**WI+CS24h-C versus Ctl-C (Up-regulated genes)**			
GO:0005154	0.0004	epidermal growth factor receptor binding	TGFA, HBEGF
GO:0005488	0.0009	binding	RAB3A, CKM, LOC100154071, LOC100154508, FOS, EPHA2, PLK3, LEO1, CYR61, ZC3H13, ACTR1A, BCL6, RCOR2, EGR1, CRYAB, FKBP14, CRABP1, JUN, KLF4, FABP5, CRYZL1, CHORDC1, CRABP1, KLF6, NR4A2, HIST1H2BD, FCN2, AURKB, IL1B1, CAST, GUCY2C, CCL2, TGFA, HBEGF, IL15
GO:0008083	0.0024	growth factor activity	TGFA, HBEGF
GO:1990837	0.0034	sequence-specific double-stranded DNA binding	BCL6, EGR1, JUN, KLF4, FOS, NR4A2
GO:0000982	0.0077	transcription factor activity. RNA polymerase II core promoter proximal region sequence-specific binding	EGR1, KLF4, FOS, NR4A2
GO:0000977	0.0081	RNA polymerase II regulatory region sequence-specific DNA binding	BCL6, EGR1, JUN, FOS, NR4A2
GO:0044212	0.0082	transcription regulatory region DNA binding	BCL6, EGR1, JUN, KLF4, FOS, NR4A2
GO:0001067	0.0090	regulatory region nucleic acid binding	BCL6, EGR1, JUN, KLF4, FOS, NR4A2
**WI+CS6h-C versus WI-C (Down-regulated genes)**			
GO:0005506	0.0002	iron ion binding	CDO1, LOC100737768, CYP39A1, CYP3A46, CYP2C42, CYP3A39, CYP4A21
GO:0016705	0.0006	oxidoreductase activity. acting on paired donors. with incorporation or reduction of molecular oxygen	CYP39A1, CYP3A46, CYP2C42, HMOX2, CYP3A39, CYP4A21
GO:0020037	0.0014	heme binding	LOC100737768, CYP39A1, CYP3A46, CYP2C42, CYP3A39, CYP4A21
GO:0004497	0.0018	monooxygenase activity	CYP39A1, CYP3A46, CYP2C42, CYP3A39, CYP4A21
GO:0015204	0.0021	urea transmembrane transporter activity	AQP3, AQP7
GO:0005548	0.0034	phospholipid transporter activity	MFSD2A, MTTP

GO:0016614	0.0092	oxidoreductase activity. acting on CH-OH group of donors	HAO2, HADHA, HSD17B4, PTGR1
GO:0015166	0.0093	polyol transmembrane transporter activity	AQP3, AQP7
GO:0015250	0.0093	water channel activity	AQP3, AQP7
GO:0015254	0.0093	glycerol channel activity	AQP3, AQP7
**WI+CS6h-C versus WI-C (Up-regulated genes)**			
GO:1990837	0.0002	sequence-specific double-stranded DNA binding	SMAD6, CEBPZ, BCL6, EGR1, KLF4, GATA3, FOS, NR4A2
GO:0044212	0.0008	transcription regulatory region DNA binding	SMAD6, CEBPZ, BCL6, EGR1, KLF4, GATA3, FOS, NR4A2
GO:0001067	0.0009	regulatory region nucleic acid binding	SMAD6, CEBPZ, BCL6, EGR1, KLF4, GATA3, FOS, NR4A2
GO:0000987	0.0012	core promoter proximal region sequence-specific DNA binding	SMAD6, CEBPZ, KLF4, GATA3, FOS
GO:0000981	0.0013	RNA polymerase II transcription factor activity. sequence-specific DNA binding	CEBPZ, BCL6, EGR1, KLF4, GATA3, FOS, NR4A2
GO:0000977	0.0027	RNA polymerase II regulatory region sequence-specific DNA binding	SMAD6, CEBPZ, BCL6, EGR1, FOS, NR4A2
GO:0001071	0.0032	nucleic acid binding transcription factor activity	SMAD6, CEBPZ, BCL6, EGR1, KLF4, GATA3, FOS, NR4A2, ID1
GO:0001077	0.0037	transcriptional activator activity. RNA polymerase II core promoter proximal region sequence-specific binding	CEBPZ, EGR1, KLF4, NR4A2
GO:0043167	0.0067	ion binding	CYP24A1, RAB3A, EPHA2, LEO1, ZC3H13, DCT, BCL6, EGR1, MAP2K6, ENTPD1, HMOX1, KLF4, GATA3, HSPH1, PEG10, CHORDC1, CKM, PVALB, NR4A2, RHOV, AWN, FCN2, GUCY2C, LPL, ATP2B1
GO:1901363	0.0076	heterocyclic compound binding	CYP24A1, RAB3A, EPHA2, SMAD6, CEBPZ, ZC3H13, BCL6, RCOR2, EGR1, HOXD1, MAP2K6, ENTPD1, HMOX1, KLF4, GATA3, HSPH1, PEG10, FOS, CKM, NR4A2, RHOV, EIF4G1, CAST, GUCY2C, ATP2B1
**WI+CS24h-C versus WI-C (Down-regulated genes)**			
GO:0005506	0.0000	iron ion binding	CDO1, CYP39A1, CYP3A46, CYP2C42, CYP3A39, CYP4A21

GO:0020037	0.0003	heme binding	CYP39A1, CYP3A46, CYP2C42, CYP3A39, CYP4A24, CYP4A21
GO:0016705	0.0003	oxidoreductase activity. acting on paired donors. with incorporation or reduction of molecular oxygen	CYP39A1, CYP2C42, HMOX2, CYP4A24, CYP4A21
GO:0004497	0.0012	monooxygenase activity	CYP39A1, CYP2C42, CYP4A24, CYP4A21
GO:0016616	0.0019	oxidoreductase activity. acting on the CH-OH group of donors. NAD or NADP as acceptor	HADHA, ENTPD1, HSD17B4, PTGR1
GO:0016491	0.0023	oxidoreductase activity	HAO2, CDO1, ACOX1, HADHA, ENTPD1, TXNRD1, HSD17B4, PTGR1
GO:0043167	0.0086	ion binding	MX2, PEX6, HAO2, ADAMDEC1, CDO1, KLF9, ARG2, ZFAND5, CYP39A1, ACOX1, ZNF217, GBP2, KLF15, CYP3A46, MKNK1, PDK4, CYP2C42, RING1, DHX58, NR1H4, DBI, ENTPD1,TXNRD1, PTGR1, CYP3A39, CYP4A24, YP4A21
GO:0050662	0.0091	coenzyme binding	HAO2, ACOX1, DBI, TXNRD1
GO:0016712	0.0096	oxidoreductase activity. acting on paired donors. with incorporation or reduction of molecular oxygen. reduced flavin or flavoprotein as one donor. and incorporation of one atom of oxygen	CYP3A46, CYP3A39
GO:1901363	0.0097	heterocyclic compound binding	MX2, NFKB2, PEX6, CEBPD, HAO2, PDK4, CYP2C42, SPDEF, DHX58, CSDC2, NR1H4, ENTPD1, TXNRD1, CYP3A39, CYP4A24, CYP4A21, IRF2, KLF9, IRF9, ZFAND5, CYP39A1, ACOX1, ZNF217, GBP2, KLF15, CYP3A46, MKNK1
**WI+CS24h-C versus WI-C (Up-regulated genes)**			
GO:1990837	0.0003	sequence-specific double-stranded DNA binding	CEBPZ, BCL6, EGR1, KLF4, GATA3, FOS, NR4A2
GO:0000981	0.0003	RNA polymerase II transcription factor activity. sequence-specific DNA binding	CEBPZ, BCL6, EGR1, KLF4, GATA3, FOS, NR4A2
GO:0044212	0.0009	transcription regulatory region DNA binding	CEBPZ, BCL6, EGR1, KLF4, GATA3, FOS, NR4A2
GO:0001067	0.0010	regulatory region nucleic acid binding	CEBPZ, BCL6, EGR1, KLF4, GATA3, FOS,

			NR4A2
GO:0001077	0.0016	transcriptional activator activity. RNA polymerase II core promoter proximal region sequence-specific binding	CEBPZ, EGR1, KLF4, NR4A2
GO:0000987	0.0038	core promoter proximal region sequence-specific DNA binding	FOS, CEBPZ, KLF4, GATA3
GO:0000977	0.0051	RNA polymerase II regulatory region sequence-specific DNA binding	CEBPZ, BCL6, EGR1, FOS, NR4A2
GO:1901363	0.0061	heterocyclic compound binding	RAB3A, CKM, FOS, EPHA2, CEBPZ, ZC3H13, BCL6, RCOR2, EGR1, HOXD1, MAP2K6, ENTPD1, KLF4, GATA3, PEG10, FOS, CKM, NR4A2, EIF4G1, AURKB, CAST, GUCY2C, ATP2B1
GO:0097159	0.0081	organic cyclic compound binding	RAB3A, CKM, FOS, EPHA2, CEBPZ, ZC3H13, BCL6, RCOR2, EGR1, HOXD1, MAP2K6, ENTPD1, KLF4, GATA3, PEG10, FOS, CKM, NR4A2, EIF4G1, AURKB, CAST, GUCY2C, ATP2B1
GO:0001010	0.0099	transcription factor activity. sequence-specific DNA binding transcription factor recruiting	KLF4
GO:0008190	0.0099	eukaryotic initiation factor 4E binding	EIF4G1
GO:0045309	0.0099	protein phosphorylated amino acid binding	LEO1
GO:0047676	0.0099	arachidonate-CoA ligase activity	ACSL4
GO:1990269	0.0099	RNA polymerase II C-terminal domain phosphoserine binding	LEO1

Table S6: Functionnal Enrichment analysis of Gene-Ontology Molecular Function terms for differentially expressed (DE) genes in corticomedullary junction tissues.

The table provided represents the enrichment analysis of Gene Ontology Molecular Function (GOMF) terms for differentially expressed genes up and down-regulated in corticomedullary junction compared conditions. The list of regulated genes, where genes without Entrez Gene ID have been removed, was used to perform the hypergeometrical statistical test, using GOstats package. (GOMFID: Gene Ontology Molecular Function Identification number; p value: p value given by the hypergeometric test ($p<0.05$); Term: Gene Ontology Molecular Function description term; and differentially expressed genes in each family: Name of gene differentially expressed in the microarray, included in the Gene Ontology family).

GOMF-ID	p value	Terms	differentially expressed genes in each family
**WI-CMJ versus Ctl-CMJ** (Down-regulated genes)			
GO:0004032	0.0006	alditol:NADP+ 1-oxidoreductase activity	AKR1B1
GO:0004033	0.0011	aldo-keto reductase (NADP) activity	AKR1B1
GO:0070888	0.0023	E-box binding	LOC100156195
GO:0001046	0.0125	core promoter sequence-specific DNA binding	LOC100156195
GO:0016614	0.0249	oxidoreductase activity. acting on CH-OH group of donors	AKR1B1
**WI-CMJ versus Ctl-CMJ** (Up- regulated genes)			
GO:0000981	0.0007	RNA polymerase II transcription factor activity. sequence-specific DNA binding	EGR1, AEBP1, JUN, KLF4, FOS, THRA
GO:0000988	0.0011	transcription factor activity. protein binding	EDF1, AEBP1, JUN, KLF4, JUP
GO:0043565	0.0020	sequence-specific DNA binding	EDF1, EGR1, AEBP1, JUN, KLF4, FOS, THRA

GO:0050839	0.0034	cell adhesion molecule binding	CYR61, JUP
GO:0000976	0.0041	transcription regulatory region sequence-specific DNA binding	EGR1, AEBP1, JUN, KLF4, FOS
GO:0003712	0.0058	transcription cofactor activity	EDF1, AEBP1, JUN, JUP
GO:0003690	0.0070	double-stranded DNA binding	EGR1, AEBP1, JUN, KLF4, FOS
GO:0001010	0.0082	transcription factor activity. sequence-specific DNA binding transcription factor recruiting	KLF4
GO:0019957	0.0082	C-C chemokine binding	ACKR1
**WI+CS6h-CMJ versus Ctl-CMJ** (Down-regulated genes)			
GO:0005506	0.0013	iron ion binding	CDO1, CYP39A1, CYP3A46, CYP2C42, CYP4A21
GO:0004497	0.0030	monooxygenase activity	CYP39A1, CYP3A46, CYP2C42, CYP4A21
GO:0016705	0.0057	oxidoreductase activity. acting on paired donors. with incorporation or reduction of molecular oxygen	CYP39A1, CYP3A46, CYP2C42 CYP4A21
GO:0005501	0.0085	retinoid binding	RBP7, PTGDS
GO:0001190	0.0102	transcriptional activator activity. RNA polymerase II transcription factor binding	CITED2, NR1H4
GO:0020037	0.0102	heme binding	CYP39A1, CYP3A46, CYP2C42, CYP4A21
GO:0046872	0.0123	metal ion binding	ZSCAN21, ZNF512, CDO1, S100A4, ARG2, S100A6, ANG, DLK1, ZFAND5, CYP39A1, TES, ZNF217, KLF15, CYP3A46, CYP2C42, INPP1, NR1H4, CYP4A21
GO:0004032	0.0131	alditol:NADP+ 1-oxidoreductase activity	AKR1B1
GO:0004300	0.0131	enoyl-CoA hydratase activity	HADHA
GO:0004329	0.0131	formate-tetrahydrofolate ligase activity	MTHFD1
GO:0004441	0.0131	inositol-1.4-bisphosphate 1-phosphatase activity	INPP1
GO:0004488	0.0131	methylenetetrahydrofolate dehydrogenase (NADP+) activity	MTHFD1
GO:0004667	0.0131	prostaglandin-D synthase activity	PTGDS
GO:0016509	0.0131	long-chain-3-hydroxyacyl-CoA dehydrogenase activity	HADHA
GO:0017172	0.0131	cysteine dioxygenase activity	CDO1
GO:0033780	0.0131	taurochenodeoxycholate 6alpha-hydroxylase activity	CYP4A21
GO:1902122	0.0131	chenodeoxycholic acid binding	NR1H4

GO:0043167	0.0140	ion binding	ZSCAN21, MX2, MTHFD1, PEX6, ARG2, ZNF512, RASL11B, CDO1, S100A4, ARG2, S100A6, ANG, DLK1, MX2, ZFAND5, CYP39A1, TES, ZNF217, GBP2, KLF15, CYP3A46, MKNK1, PDK4, CYP2C42, INPP1, NR1H4, CYP4A21
GO:0004033	0.0260	aldo-keto reductase (NADP) activity	AKR1B1
GO:0004053	0.0260	arginase activity	ARG2
GO:0004887	0.0260	thyroid hormone receptor activity	NR1H4
GO:0008022	0.0260	protein C-terminus binding	PEX6
GO:0038181	0.0260	bile acid receptor activity	NR1H4
GO:0042030	0.0260	ATPase inhibitor activity	PLN
GO:0046935	0.0260	1-phosphatidylinositol-3-kinase regulator activity	PIK3R5
GO:0001191	0.0274	transcriptional repressor activity. RNA polymerase II transcription factor binding	CITED2, NR1H4
GO:0005246	0.0387	calcium channel regulator activity	PLN
GO:0009931	0.0387	calcium-dependent protein serine/threonine kinase activity	MKNK1
GO:0052745	0.0387	inositol phosphate phosphatase activity	INPP1
**WI+CS6h-CMJ versus Ctl-CMJ** (Up-regulated genes)			
GO:0015347	0.0003	sodium-independent organic anion transmembrane transporter activity	SLC22A6, SLC22A8
GO:1990837	0.0003	sequence-specific double-stranded DNA binding	FOS, PBX3, BCL6, EGR1, JUN, NR4A2, GATA3
GO:0005452	0.0006	inorganic anion exchanger activity	SLC22A6
GO:0000977	0.0008	RNA polymerase II regulatory region sequence-specific DNA binding	FOS, PBX3, BCL6, EGR1, JUN, NR4A2
GO:0044212	0.0009	transcription regulatory region DNA binding	FOS, PBX3, BCL6, EGR1, JUN, NR4A2
GO:0001067	0.0010	regulatory region nucleic acid binding	FOS, PBX3, BCL6, EGR1, JUN, NR4A2, GATA3
GO:0003677	0.0019	DNA binding	LOC100154508, FOS, PBX3, BCL6, RCOR2, EGR1, JUN, GATA3, NAR4A2, HIST1H2BD
GO:0000982	0.0052	transcription factor activity. RNA polymerase II core promoter proximal region sequence-specific binding	FOS, EGR1, GATA3, NR4A2

GO:0070851	0.0070	growth factor receptor binding	HBEGF, IL1B1
GO:0099516	0.0081	ion antiporter activity	SLC22A6, SLC22A8
GO:0003991	0.0099	acetylglutamate kinase activity	NAGS
GO:0008403	0.0099	25-hydroxycholecalciferol-24-hydroxylase activity	CYP24A1
GO:0030342	0.0099	1-alpha.25-dihydroxyvitamin D3 24-hydroxylase activity	CYP24A1
**WI+CS24h-CMJ versus Ctl-CMJ** (Down-regulated genes)			
GO:0005506	0.0014	iron ion binding	CDO1, CYP39A1, CYP3A46, CYP2C42, CYP4A21
GO:0004497	0.0033	monooxygenase activity	CYP39A1, CYP3A46, CYP2C42, CYP4A21
GO:0016705	0.0062	oxidoreductase activity. acting on paired donors. with incorporation or reduction of molecular oxygen	CYP39A1, CYP3A46, CYP2C42, CYP4A21
GO:0046872	0.0069	metal ion binding	ZSCAN21, S100A4, ARG2, CDO1, S100A4, ARG2, ANG, F10, DLK1, ZFAND5, CYP39A1, TES, ZNF217, KLF15, CYP3A46, CYP2C42, INPP1, NR1H4, PTGR1, CYP4A21, LIMK2
GO:0005501	0.0089	retinoid binding	RBP7, PTGDS
GO:0043167	0.0092	ion binding	ZSCAN21, MX2, MTHFD1, S100A4, PEX6, MTHFD1, ARG2, ARG2, RASL11B, CDO1, S100A4, ARG2, ANG, F10, DLK1, MX2, ZFAND5, CYP39A1, TES, ZNF217, GBP2, KLF15, CYP3A46, MKNK1, PDK4, CYP2C42, INPP1, NR1H4, PTGR1, CYP4A21, LIMK2
**WI+CS24h-CMJ versus Ctl-CMJ** (Up-regulated genes)			
GO:1990837	0.0000	sequence-specific double-stranded DNA binding	FOS, SMAD6, PBX3, LOC106504139, BCL6, EGR1, DDIT3, JUN, KLF4, GATA3, NR4A2
GO:0044212	0.0000	transcription regulatory region DNA binding	FOS, SMAD6, PBX3, LOC106504139, BCL6, EGR1, DDIT3, JUN, KLF4, GATA3, NR4A2
GO:0001067	0.0000	regulatory region nucleic acid binding	FOS, SMAD6, PBX3, LOC106504139, BCL6, EGR1, DDIT3, JUN, KLF4, GATA3, NR4A2
GO:0003677	0.0000	DNA binding	LOC100154071, LOC100154508, FOS, SMAD6, PBX3, LOC100154181, LOC106504139, BCL6, RCOR2, EGR1, HOXD1, DDIT3, JUN, KLF4,

			GATA3, NR4A2, HIST1H2BD
GO:1901363	0.0002	heterocyclic compound binding	CYP24A1, NXF1, LOC100154071, LOC100154508, FOS, EPHA2, PLK3, SMAD6, PBX3, LOC100154181, ZC3H13, LOC106504139, BCL6, RCOR2, EGR1, HOXD1, STK31, DDIT3, JUN, KDR, ENTPD1, KLF4, GATA3, KLF6, NR4A2, HIST1H2BD, RHOV, HSP70,2, AURKB, GUCY2C
GO:0003700	0.0003	transcription factor activity. sequence-specific DNA binding	FOS, SMAD6, PBX3, LOC106504139, BCL6, EGR1, DDIT3, JUN, KLF4, GATA3, NR4A2
GO:0097159	0.0004	organic cyclic compound binding	CYP24A1, NXF1, LOC100154071, LOC100154508, FOS, EPHA2, PLK3, SMAD6, PBX3, LOC100154181, ZC3H13, LOC106504139, BCL6, RCOR2, EGR1, HOXD1, STK31, DDIT3, JUN, KDR, ENTPD1, KLF4, GATA3, KLF6, NR4A2, HIST1H2BD, RHOV, HSP70,2, AURKB, GUCY2C
GO:0000977	0.0005	RNA polymerase II regulatory region sequence-specific DNA binding	PBX3, LOC106504139, BCL6, EGR1, NR4A2
GO:0000982	0.0017	transcription factor activity. RNA polymerase II core promoter proximal region sequence-specific binding	FOS, LOC106504139, GATA3
GO:0000987	0.0022	core promoter proximal region sequence-specific DNA binding	KLF4, GATA3
GO:0001077	0.0047	transcriptional activator activity. RNA polymerase II core promoter proximal region sequence-specific binding	EGR1, DDIT3, KLF4, NR4A2
GO:0001227	0.0064	transcriptional repressor activity. RNA polymerase II transcription regulatory region sequence-specific binding	LOC106504139, BCL6, GATA3
GO:0000978	0.0079	RNA polymerase II core promoter proximal region sequence-specific DNA binding	FOS, SMAD6, DDIT3, JUN
**WI+CS6h-CMJ versus WI-CMJ** (Down-regulated genes)			
GO:0005506	0.0000	iron ion binding	CDO1, FAXDC2, CYP39A1, CYP2C42, LCN2, CYP2A19, CYP3A39, CYP4A24
GO:0004887	0.0007	thyroid hormone receptor activity	NR1H4, THRA

GO:0020037	0.0017	heme binding	CYP39A1, CYP3A46, CYP2C42, CYP2A19, CYP3A39, CYP4A24, CYP4A21
GO:0016712	0.0026	oxidoreductase activity. acting on paired donors. with incorporation or reduction of molecular oxygen. reduced flavin or flavoprotein as one donor. and incorporation of one atom of oxygen	CYP3A46, CYP2A19, CYP3A39
GO:0001190	0.0034	transcriptional activator activity. RNA polymerase II transcription factor binding	CITED2, NR1H4, HYAL2
GO:0015204	0.0041	urea transmembrane transporter activity	AQP7, AQP3
GO:0004364	0.0043	glutathione transferase activity	GSTA4, LOC100153094, GSTA2
GO:0016705	0.0057	oxidoreductase activity. acting on paired donors. with incorporation or reduction of molecular oxygen	CYP39A1, SCD5, CYP2C42, CYP4A24, CYP4A21
GO:0042277	0.0058	peptide binding	TMEM158, ANG, SLA-1, SLA-5, ITM2C
GO:0030234	0.0065	enzyme regulator activity	PLN, SOCS2, LOC100153899, AZIN2, SERPING1, RING1, UABP-2, PPP2R1A, EPO, PPP1R14A, CAV1, HYAL2
GO:0005540	0.0067	hyaluronic acid binding	ACAN, HYAL2
**WI+CS6h-CMJ versus WI-CMJ** (Up-regulated genes)			
GO:1901363	0.0019	heterocyclic compound binding	CYP24A1, CKM, SARS, ZC3H13, BCL6, RCOR2, STK31, LOC100739425, CCDC47, HMOX1, CKMT2, DICER1, EIF4G1, CAST, GUCY2C, ATP2B1
GO:0097159	0.0025	organic cyclic compound binding	CYP24A1, CKM, SARS, ZC3H13, BCL6, RCOR2, STK31, LOC100739425, CCDC47, HMOX1, CKMT2, DICER1, EIF4G1, CAST, GUCY2C, ATP2B1
GO:0005524	0.0038	ATP binding	CKM, SARS, STK31, CKMT2, DICER1, GUCY2C, ATP2B1
GO:0030554	0.0043	adenyl nucleotide binding	CKM, SARS, STK31, CKMT2, DICER1, GUCY2C, ATP2B1
GO:0004828	0.0065	serine-tRNA ligase activity	SARS
GO:0008190	0.0065	eukaryotic initiation factor 4E binding	EIF4G1
GO:0008403	0.0065	25-hydroxycholecalciferol-24-hydroxylase activity	CYP24A1

GO:0030342	0.0065	1-alpha.25-dihydroxyvitamin D3 24-hydroxylase activity	CYP24A1
GO:0045309	0.0065	protein phosphorylated amino acid binding	LEO1
GO:1990269	0.0065	RNA polymerase II C-terminal domain phosphoserine binding	LEO1
**WI+CS24h-CMJ versus WI-CMJ** (Down-regulated genes)			
GO:0005506	0.0000	iron ion binding	CDO1, FAXDC2, CYP39A1, CYP3A46, CYP2C42, CYP2A19, CYP3A39, CYP4A24, CYP4A21,
GO:0016705	0.0001	oxidoreductase activity. acting on paired donors. with incorporation or reduction of molecular oxygen	CYP39A1, SCD5, CYP2C42, HMOX2, FMO1, CYP4A24, CYP4A21
GO:0004887	0.0007	thyroid hormone receptor activity	NR1H4, THRA
GO:0020037	0.0014	heme binding	CYP39A1, CYP3A46, CYP2C42, CYP2A19, CYP3A39, CYP4A24, CYP4A21
GO:0004497	0.0018	monooxygenase activity	CYP39A1, CYP2C42, FMO1, CYP4A24, CYP4A21
GO:0016712	0.0024	oxidoreductase activity. acting on paired donors. with incorporation or reduction of molecular oxygen. reduced flavin or flavoprotein as one donor. and incorporation of one atom of oxygen	CYP3A46, CYP2A19, CYP3A39
GO:0001190	0.0031	transcriptional activator activity. RNA polymerase II transcription factor binding	CITED2, NR1H4, HYAL2
GO:0043167	0.0036	ion binding	MX2, ABL2, RHOG, PEX6, MTHFD1, PLEKHF1, IRF2BP1, RASL11B, CDO1, ZYX, FAXDC2, ZMAT3, ACE, ARG2, KLF13, ANG, F10, DLK1, ZFAND5, CYP39A1, ACOX1, MFGE8, F2, RHOB, ZNF217, TNNC1, KLF15, CYP3A46, MKNK1, PDK4, ARF3, PCK2, ACAN, CYP2C42, RING1, NR1H4, CAPN1, FMO1, DBI, ENTPD1, TXNRD1, THRA, PTGR1, CYP2A19, CYP3A39, CYP4A24, CYP4A21
GO:0015204	0.0038	urea transmembrane transporter activity	AQP3, AQP7
GO:0004364	0.0040	glutathione transferase activity	GSTA4, LOC100153094, GSTA2
GO:0005540	0.0063	hyaluronic acid binding	ACAN, HYAL2

GO:0004252	0.0067	serine-type endopeptidase activity	PIK3IP1, CFD, F10, F2, CELA2A
GO:0016627	0.0073	oxidoreductase activity. acting on the CH-CH group of donors	ACOX1, SRD5A2, PTGR1
**WI+CS24h-CMJ versus WI-CMJ** (Up-regulated genes)			
GO:0003676	0.0046	nucleic acid binding	CEBPZ, ZC3H13, LOC106504139, BCL6, RCOR2, HOXD1, ZNF652, LOC100739425, SRP19, NR4A2, HIST1H2BD, EIF4G1, CAST
GO:0008190	0.0080	eukaryotic initiation factor 4E binding	EIF4G1
GO:0047676	0.0080	arachidonate-CoA ligase activity	ACSL4

Table S7: RT-qPCR confirmation of selected genes issues from microarray data.

The table provided the expression of mRNA detected by RT-PCR, in renal cortex and renal corticomedullary junction (CMJ) tissue, at the end of warm ischemia period (WI), at the end of WI + 6h Cold storage (WI+CS6h) and WI+CS24h (n=5 per group). Results, mean ± standard error mean, were expressed in relative fold change to control group (Ctl). Statistical analyses were performed with Kruskal-Wallis Multiple-Comparison Dunn's Test, NCSS software.

Gene	Tissue	WI	WI+CS6h	WI+CS24h
CCL2	Cortex	1.17±0.26	4.38±0.41<br>*p<0.05 vs WI*	4.68±0.43<br>*p<0.05: vs WI, vs Ctl*
CCL2	CMJ	1.41±0.16	3.65±0.43<br>*p<0.05: vs WI, vs Ctl*	5.92±0.68<br>*p<0.05: vs WI, vs Ctl*
GSTA2	Cortex	2.19±0.84<br>*p<0.05 vs Ctl*	0.97±0.31	0.74±0.14
GSTA2	CMJ	1.45±0.52	0.50±0.14	0.58±0.14
EXPH5	Cortex	0.73±0.18	0.50±0.22	0.35±0.19<br>*p<0.05 vs Ctl*
EXPH5	CMJ	1.39±0.20	0.88±0.19	0.38±0.13<br>*p<0.05: vs WI, vs Ctl*
MCOLM3	Cortex	0.77±0.15	2.38±0.35<br>*p<0.05: vs WI, vs Ctl*	1.99±0.16<br>*p<0.05 vs WI*
MCOLM3	CMJ	0.73±0.16	1.41±0.23	1.70±0.29<br>*p<0.05 vs WI*
CKM	Cortex	1.05±0.21	6.01±1.52<br>*p<0.05: vs WI, vs Ctl*	4.31±1.24<br>*p<0.05: vs WI, vs Ctl*
CKM	CMJ	0.62±0.06	3.73±0.84<br>*p<0.05: vs WI, vs Ctl*	5.65±1.46<br>*p<0.05: vs WI, vs Ctl*
PDK4	Cortex	1.84±0.35	0.23±0.06<br>*p<0.05: vs WI, vs Ctl*	0.17±0.05<br>*p<0.05: vs WI, vs Ctl*
PDK4	CMJ	1.35±0.14	0.15±0.04<br>*p<0.05: vs WI, vs Ctl*	0.11±0.03<br>*p<0.05: vs WI, vs Ctl*

CDO1	Cortex	1.57±0.34	0.65±0.24	0.37±0.09<br>*p<0.05 vs WI*
CDO1	CMJ	1.69±0.39	0.28±0.08<br>*p<0.05 vs WI*	0.37±0.21<br>*p<0.05 vs WI*
FOS	Cortex	4.76±1.42<br>*p<0.05 vs Ctl*	3.56±0.45<br>*p<0.05 vs Ctl*	3.35±0.34<br>*p<0.05 vs Ctl*
FOS	CMJ	14.35±2.95<br>*p<0.05 vs Ctl*	8.09±0.96<br>*p<0.05 vs Ctl*	6.69±0.45<br>*p<0.05 vs Ctl*
HIST1H2AB	Cortex	2.04±0.55	2.20±0.86	2.39±0.81
HIST1H2AB	CMJ	1.51±0.40	1.02±0.33	1.47±0.44
CYP2C42	Cortex	2.76±1.3	0.55±0.15	0.37±0.09<br>*p=0.05 vs WI*
CYP2C42	CMJ	2.48±0.98	0.43±0.15	0.36±0.06
FUT2	Cortex	1.07±0.06	25.25±2.04<br>*p<0.05: vs WI, vs Ctl*	22.50±3.97<br>*p<0.05: vs WI, vs Ctl*
FUT2	CMJ	0.94±0.15	14.95±2.19<br>*p<0.05: vs WI, vs Ctl*	18.91±2.69<br>*p<0.05: vs WI, vs Ctl*
ARG2	Cortex	1.01±0.06	0.39±0.10<br>*p<0.05: vs WI, vs Ctl*	0.34±0.08<br>*p<0.05: vs WI, vs Ctl*
ARG2	CMJ	1.09±0.11	0.32±0.09<br>*p<0.05: vs WI, vs Ctl*	0.25±0.06<br>*p<0.05: vs WI, vs Ctl*
PLK3	Cortex	1.40±0.18	1.46±0.12<br>*p<0.05 vs Ctl*	1.46±0.23
PLK3	CMJ	2.66±0.20<br>*p<0.05 vs Ctl*	2.04±0.30<br>*p<0.05 vs Ctl*	1.64±0.22<br>*p<0.05 vs WI*
VAV3	Cortex	1.26±0.18	0.85±0.10<br>*p<0.05 vs WI*	0.79±0.11<br>*p<0.05 vs WI*
VAV3	CMJ	1.38±0.35	0.91±0.12	0.92±0.12
JUN	Cortex	1.40±0.18	2.67±0.41<br>*p<0.05 vs Ctl*	2.31±0.44<br>*p<0.05 vs Ctl*
JUN	CMJ	1.94±0.26<br>*p<0.05 vs Ctl*	2.32±0.35<br>*p<0.05 vs Ctl*	2.76±0.38<br>*p<0.05 vs Ctl*
EGR1	Cortex	1.17±0.15	2.22±0.76	1.98±0.51<br>*p<0.05 vs Ctl*

EGR1	CMJ	3.44±0.70<br>*p<0.05 vs Ctl*	4.92±1.39<br>*p<0.05 vs Ctl*	3.76±1.11<br>*p<0.05 vs Ctl*
KLF4	Cortex	1.47±0.28	8.39±2.90<br>*p<0.05: vs WI, vs Ctl*	8.49±2.94<br>*p<0.05: vs WI, vs Ctl*
KLF4	CMJ	1.09±0.28	2.12±0.49	3.06±1.05
HSP70	Cortex	1.10±0.03	2.15±0.37<br>*p<0.05: vs WI, vs Ctl*	1.78±0.23<br>*p<0.05 vs Ctl*
HSP70	CMJ	0.96±0.06	1.05±0.16	1.39±0.29

Table S8: Primer sequences used for RT-qPCR analysis

**Target**	**Forward primer**	**Reverse primer**
B-Actin	GATCGTGCGGGACATCAAG	GCCATCTCCTGCTCGAAGTC
RPLP0	AGAAACTGTTGCCTCACATCC	CCTTATTGGCCAGCAGCA
SDHA	GAGTTCGTGCAGTTCCACCCTA	CCTCTCACCCTGGCTGTTGATA
Cyclophilin A (CYA62)	AAGACTGAGTGGTTGGATGG	AATGGTGATCTTCTTGCTGGT
CCL2	ACCAGCAGCAAGTGTCCTAA	GTCCAGGTGGCTTATGGAGTC
GSTA2	CTCTGCTGAAGGCCCTGAAA	AGTGGGAGGCTTCCTCTGG
EXPH5	TCAAAGGGGCGGACTTATCG	GAGTGCCGTGTCTGTTCTCA
MCOLN3	GGGAACCACGCTTACGAGAA	AGCTTCATCCGGCTCAACAA
CKM	GACAGGTGTGGACAACCCAG	CCGTGTCGGTCTTGGATGAT
PDK4	CACATTGGCAGCATTGACCC	TCGACTGTAGCCCTCATTGC
CDO1	GAAGCCTATGAGAGCGACCC	CAAAGGCATGGCACGTATCG
FOS	TCAGAGCATTGGCAGAAGGG	GTGTGTCAGTCAGCTCCCTC
HIST1H2AB	GCAGCTTGCCATCCGTAATG	GTCTTCTTTGGCAGCAGCAC
CYP2C42	ATCCCCAAGGGCACAACAAT	GGCCAGGGTCAAACACTTCT
FUT2	CCGAGTGGATTGGGATCGAG	CTCCCTGTGCCTTGGAAGTG
ARG2	CCGAAGAAGTCCGTCCACTC	AGTCTTTAAGGTGGCAGCCC
PLK3	CGTGTGGCTGTGCTTTTCAA	GTAGGAGGCGAAATACCGCA
VAV3	CGGGCGCACTCTATTAACCT	CACCTTTCCGAAGTCACGGA
JUN	ACGACCTTCTACGACGATGC	GTTCAGGGTCATGCTCTGCT
EGR1	TTACCCCAGCCAAACCACTC	GCTGTCGTTGGATTGCACTG
KLF4	CATCAACGATGTGAGCCCCT	GGCATGAGCTCTTGGTAATGG
HSP70	CCGGTGCCGATATCTCGAT	GCGACCAGGTACGCAGAGTAG
